# Chemico-Biological Characterization of Torpedino Di Fondi^®^ Tomato Fruits: A Comparison with San Marzano Cultivar at Two Ripeness Stages

**DOI:** 10.3390/antiox9101027

**Published:** 2020-10-21

**Authors:** Cinzia Ingallina, Alessandro Maccelli, Mattia Spano, Giacomo Di Matteo, Antonella Di Sotto, Anna Maria Giusti, Giuliana Vinci, Silvia Di Giacomo, Mattia Rapa, Salvatore Ciano, Caterina Fraschetti, Antonello Filippi, Giovanna Simonetti, Carlos Cordeiro, Marta Sousa Silva, Maria Elisa Crestoni, Anatoly P. Sobolev, Simonetta Fornarini, Luisa Mannina

**Affiliations:** 1 Dipartimento di Chimica e Tecnologie del Farmaco, Sapienza Università di Roma, P. le Aldo Moro 5, 00185 Rome, Italy; cinzia.ingallina@uniroma1.it (C.I.); alessandro.maccelli@uniroma1.it (A.M.); mattia.spano@uniroma1.it (M.S.); giacomo.dimatteo@uniroma1.it (G.D.M.); caterina.fraschetti@uniroma1.it (C.F.); antonello.filippi@uniroma1.it (A.F.); simonetta.fornarini@uniroma1.it (S.F.); luisa.mannina@uniroma1.it (L.M.); 2 Dipartimento di Fisiologia e Farmacologia “V. Ersparmer”, Sapienza Università di Roma, P. le Aldo Moro 5, 00185 Rome, Italy; antonella.disotto@uniroma1.it (A.D.S.); silvia.digiacomo@uniroma1.it (S.D.G.); 3 Dipartimento di Medicina Sperimentale Sapienza, Università di Roma, P. le Aldo Moro 5, 00185 Rome, Italy; annamaria.giusti@uniroma1.it; 4 Dipartimento di Management, Laboratorio di Merceologia, Sapienza Università di Roma, Via del Castro Laurenziano 9, 00161 Rome, Italy; giuliana.vinci@uniroma1.it (G.V.); mattia.rapa@uniroma1.it (M.R.); salvatore.ciano@uniroma1.it (S.C.); 5 Dipartimento di Biologia Ambientale, Sapienza Università di Roma, P. le Aldo Moro 5, 00185 Rome, Italy; giovanna.simonetti@uniroma1.it; 6 Laboratório de FT-ICR e Espectrometria de Massa Estrutural, Faculdade de Ciências da Universidade de Lisboa, Campo-Grande, 1749-016 Lisboa, Portugal; cacordeiro@fc.ul.pt (C.C.); mfsilva@fc.ul.pt (M.S.S.); 7 Istituto per i Sistemi Biologici, Laboratorio di Risonanza Magnetica “Annalaura Segre”, CNR, 00015 Monterotondo (Rome), Italy

**Keywords:** tomatoes, NMR spectroscopy, FT-ICR mass spectrometry, ripening stage, phenolics, antioxidant activity, metabolomics, phytochemicals

## Abstract

Torpedino di Fondi (TF) is a hybrid tomato landrace developed in Sicily and recently introduced in the south Lazio area along with the classical San Marzano (SM) cultivar. The present study aimed at characterizing TF tomatoes at both pink and red ripening stages, and at comparing them with traditional SM tomatoes. A multidisciplinary approach consisting of morphological, chemical (FT-ICR MS, NMR, HPLC, and spectrophotometric methods), and biological (antioxidant and antifungal in vitro activity) analyses was applied. Morphological analysis confirmed the mini-San Marzano nature and the peculiar crunchy and solid consistency of TF fruits. Pink TF tomatoes displayed the highest content of hydrophilic antioxidants, like total polyphenols (0.192 mg/g), tannins (0.013 mg/g), flavonoids (0.204 mg/g), and chlorophylls a (0.344 mg/g) and b (0.161 mg/g), whereas red TF fruits were characterized by the highest levels of fructose (3000 mg/100 g), glucose (2000 mg/100 g), tryptophan (2.7 mg/100 g), phenylalanine (13 mg/100 g), alanine (25 mg/100 g), and total tri-unsaturated fatty acids (13% mol). Red SM fruits revealed the greatest content of lipophilic antioxidants, with 1234 mg/g of total carotenoids. In agreement with phenolics content, TF cultivar showed the greatest antioxidant activity. Lastly, red TF inhibited *Candida* species (*albicans*, *glabrata* and *krusei*) growth.

## 1. Introduction

San Marzano (SM) is a traditional tomato landrace grown in south Italy suitable for both fresh consumption and processing. The distinct SM organoleptic properties made this variety a worldwide model for tomato quality traits, although the scarcity of genetic resistance against pathogens represents a critical SM weakness [1]. Throughout the years, both natural and in vitro selections have led to new SM tomatoes with peculiar accessions; however, several ecotypes not suitable for local environments gradually disappeared. The term “San Marzano” refers to a population of tomatoes with a wide range of characteristics [2]. In this context, a new SM cultivar, namely, Torpedino di Fondi (TF) has been recently introduced in the south Lazio area. Developed in Sicily (Licata and Vittoria), TF is characterized by a peculiar sweetness and palatability and, due to its smaller size and weight compared to SM, it is defined as mini-San Marzano.

Different analytical methodologies, such as NMR, MS, GC-MS, and HPLC, have been applied to characterize different SM cultivars from chemical [2,3,4,5,6,7], sensorial [4,5], and genomic [1,4,8] points of view. However, to the best of our knowledge, TF tomatoes have not been characterized yet.

It is well established that plants and vegetable foodstuffs represent a unique reservoir of nutrients and phytochemicals with health implications [9]. Tomatoes and tomato-based food have proven to possess a wide variety of bioactive compounds that are beneficial for human well-being; among these, dietary antioxidants like carotenoids, polyphenols, and vitamins are the most abundant in tomato fruits. Indeed, carotenoids have shown to play an important role in reducing the incidence of some chronic diseases, like cancer and cardiovascular diseases [10], whereas polyphenols in tomatoes have proved to prevent the oxidative damage [11,12,13,14] in human cells. Recently, the influence of the cultivation system on the polyphenols content has been found to depend mostly on variety and year than the cultivation and drying methods [15,16].

Moreover, a recent study provided evidence on the activity of ethanolic extracts of *Solanum lycopersicum* at concentration of 6.25 mg/mL against *C. albicans*, *C. guilliermondii*, and *C. lusitaniae* isolated from HIV positive patients [17]. Conversely, extracts from tomato crop remains at the end of the cultivation cycle displayed a low antifungal activity against the microfungi *Aspergillus* and *Penicillium species* [18]. Interestingly, an antimicrobial snaking peptide (SN2) obtained from *Solanum lycopersicum* tested as a recombinant peptide in *E. coli* exhibited strong fungicidal bioactivity ascribed to biomembrane perforation [19]. *Candida* spp. is present in the gut, but an overproduction may lead to serious health problems. Some diseases, such as Crohn’s disease and ulcerative colitis, are associated with an overgrowth of *Candida* in the gastrointestinal tract [20]. *Candida* overgrowth can be prevented by healthy foods.

The aim of the present study was to fully characterize for the first time TF tomatoes at pink and red ripeness stages, both considered ideal for fresh consumption, through the investigation of morphological characteristics, metabolite profile (carbohydrates, amino acids, organic acids, polyphenols, pigments, sterols, fatty acids), and the evaluation of antioxidant and antifungal (towards *Candida* spp.) properties of tomato extracts. In this study, a comparison with traditional SM tomatoes was also carried out at the same experimental conditions.

The present multidisciplinary analytical approach here employed was already successfully applied to other matrices such as sweet pepper [21], celery [22], extra-virgin olive oil [23], hemp inflorescences [24], but never to tomato fruits. In this study, the powerful combination of high-resolution NMR spectroscopy and FT-ICR MS, not yet widely exploited and largely complementing each other, allowed to obtain a broad untargeted chemical profile, whereas HPLC and spectrophotometric targeted methodologies enabled the content of biogenic amines, polyphenols, and pigments, respectively, to be quantified.

Herein, a biological evaluation of the extracts was finally carried out in terms of antioxidant properties, antifungal activity, and enzyme inhibition. The combined results of radical-scavenging activity, formation of advanced glycation final product (AGE), and the cytoprotective activity towards the oxidative damage induced by tert-butyl hydroperoxide solution (tBuOOH) has allowed to estimate the TF and SM tomatoes antioxidant properties. Moreover, in vitro antifungal activity of the tomatoes extracts towards four *C. albicans*, three *C. glabrata*, and two *C. krusei* strains was assayed.

## 2. Materials and Methods

### 2.1. Plant Material

Fresh fruits of *Solanum lycopersicum* L. TF variety were grown and collected by Mafalda SRL (41.342622, 13.420856), whereas SM fresh fruits were grown and collected by San Leone Agricultural Cooperative (41.293929, 13.397638) sited both in Fondi (Latina, Italy). Fondi is characterized by a Mediterranean climate with an average air temperature (T) = 19.5 °C and humidity = 54.1% during the growing season. Irrigation and plant protection, as well as the weed control were carried out following local practices. Samples were harvested at two different ripening stages according to market demand, namely pink (P) stage (from 30% to 60% of not green tomato skin) and red stage (R) (about 90% of not green tomato skin) showing a red colour (Figure S1). Peduncles were removed, some fresh fruits were subjected to morphological analysis and the extraction procedure, while other samples were instantly stored at −80 °C.

### 2.2. Chemicals

Rutin, quercetin, 2,2-diphenyl-1-picrylhydrazyl (DPPH), 2,2′-azino-bis(3-thylbenzothiazoline-6-sulfonic acid) diammonium salt (ABTS), 2,2′-azobis(2-methylpropionamidine) dihydrochloride (AAPH), trolox, polyvinylpyrrolidone (PVP), tert-butyl hydroperoxide solution (tBuOOH; 900 mg mL^−1^), ferrozine, hydroxylamine hydrochloride, iron(III) chloride (FeCl_3_ × 6H_2_O), iron(II) sulfate heptahydrate (FeSO_4_ × 7H_2_O), potassium hexacyanoferrate(III), iron(II) chloride (FeCl_2_×4H_2_O), magnesium oxide, doxorubicin, 3-(4,5-dimethylthiazol-2-yl)-2,5-diphenyltetrazolium bromide (MTT), 2,7-dichlorofluorescein diacetate (DCFH-DA), and the solvents (HPLC-MS purity grade) were purchased from Sigma-Aldrich (Milan, Italy). Methanol (HPLC-grade), formic acid (99%), perchloric acid (70%), acetone (analytical-grade), chloroform, acetonitrile (HPLC-grade) were obtained from Carlo Erba Reagenti (Milan, Italy). Double-distilled water was obtained using a Millipore Milli-Q Plus water treatment system (Millipore Bedford Corp., Bedford, MA). Sodium carbonate (Na_2_CO_3_; 99.999% purity), Folin–Ciocalteu’s phenol reagent, tannic acid (Ph. Eur. purity) and aluminum chloride hexahydrate (AlCl_3_ × 6H_2_O; Ph. Eur. purity) were purchased from Merck (Darmstadt, Germany). Deuterated water (D_2_O) 99.97 atom% of deuterium, methanol-D4 99.80 atom% of deuterium, chloroform-D 99.80 atom% of deuterium + 0.03% tetramethylsilane (TMS), and 3-(trimethylsilyl)-propionic-2,2,3,3-d_4_ acid sodium salt (TSP) were purchased from Euriso-Top (Saclay, France).

### 2.3. Morphological Analysis

Ten fresh fruits for both tomato varieties and redness stages were subjected to morphological analysis, in order to describe their size (length and diameter), weight, and shape. For each fruit, a careful separation of different components, including peel (i.e., exocarp of the fruit), pulp (i.e., mesocarp of the fruit), seeds (removed with the internal juice), and juice, was performed. The peel was gently separated from the pulp by using a scalpel. The detached components were examined and weighted, and their amount in the whole fruit was determined.

#### Pigments Characterization

The total carotenoids and chlorophylls analysis in peel and pulp of SM and TF tomatoes samples were performed according to Solovchenko and co-workers [25] with some modifications. The peel was cut from the surface of the fruits, carefully freed from the pulp, and successively weighted. Both peel and pulp from each sample were twice washed with distilled water for 1 min and dried with filter paper. To remove the cuticular lipids, peel fraction was washed with 2 mL portion of chloroform for 1 min. Pigments were extracted according to the Folch method [26]. The samples were homogenized with mortar and pestle in 6 mL of chloroform-methanol (2:1, *v/v*) and, to prevent chlorophyll pheophytinization, 30 mg of MgO were added before the homogenization. The homogenate was passed through a paper filter and after an amount of distilled water equal to 1/5 of the extract volume was added. Finally, this mixture was centrifuged in a glass tube test for 20 min at 2469× *g* for 20 min at 10 °C to complete separation of chloroform fraction from hydroalcoholic one. Absorption spectra of the chloroform phase were recorded with a Beckman Coulter DU 800 instruments, in the range of 350–800 nm with a spectral resolution of 0.5 nm, at a temperature of 20 °C. The concentrations of chlorophyll a and b as well as total carotenoids (*mg/g* of sample) were determined according to Wellburn [27].

### 2.4. Extraction Procedures

Fifteen fresh whole fruits from pink TF (TF_P_), red TF (TF_R_), pink SM (SM_P_), and red SM (SM_R_) were frozen and ground in liquid nitrogen to obtain a homogeneous pool and subjected to the Bligh–Dyer extraction method, which allows to extract both water-soluble and liposoluble metabolites in a quantitative manner.

In details, about 1.0 g of samples (peel, pulp, and seeds) of each variety was added sequentially with 3 mL methanol/chloroform (2:1 *v/v*) mixture, 1 mL of chloroform, and 1.2 mL of distilled water. After each addition the sample was carefully shacked. The emulsion was maintained at 4 °C for 40 min. The sample was then centrifuged (4200× *g* for 15 min at 4 °C) and the upper (hydroalcoholic) and lower (organic) phases were carefully separated. The pellets were re-extracted using half of the solvent volumes (in the same conditions described above) and the separated fractions were pooled. Both hydroalcoholic and organic fractions were filtered with Whatman paper filters and dried under a gentle N_2_ flow at room temperature until the solvent was completely evaporated [22]. The dried phases were stored at −20 °C until further analyses. The values of drug to extract ratio (DER) are reported in Table 1.

### 2.5. Metabolite Profile

#### 2.5.1. NMR Analysis

The dried organic fraction of each sample was dissolved in 0.7 mL of a CDCl_3_/CD_3_OD mixture (2:1 *v*/*v*) and then placed into a 5 mm NMR tube. Finally, the NMR tube was flame sealed. Conversely, the dried hydroalcoholic phase of each sample was solubilized in 0.7 mL of 400 mM phosphate buffer/D_2_O containing 1 mM solution of TSP as internal standard and then transferred into a 5 mm NMR tube. NMR spectra of all hydroalcoholic and organic extracts were recorded at 27 °C on a Bruker AVANCE 600 spectrometer operating at the proton frequency of 600.13 MHz and equipped with a Bruker multinuclear z-gradient 5 mm probe head. ^1^H spectra were referenced to methyl group signals of TSP (δ = 0.00 ppm) in D_2_O and to the residual CHD_2_ signal of methanol (set to 3.31 ppm) in CD_3_OD/CDCl_3_ mixture. ^1^H spectra of hydroalcoholic extracts were acquired with 256 transients with a recycle delay of 5 s. The residual HDO signal was suppressed using a pre-saturation. The experiment was carried out by using 45° pulse of 6.5–7.5 μs, 32 K data points. ^1^H spectra of extracts in CD_3_OD/CDCl_3_ were acquired with 256 transients, recycle delay of 5 s, and 90° pulse of 9–11 μs, 32 K data points. The two-dimensional (2D) NMR experiments, such as ^1^H-^1^H TOCSY, ^1^H-^13^C HSQC, and ^1^H-^13^C HMBC, were carried out under the same experimental conditions previously reported [28]. The integrals of 26 selected signals in hydroalcoholic extract (Table 2) were measured using the Bruker TOPSPIN software and normalized with respect to the resonance at 0.00 ppm, due to methyl group signal of TSP, set to 100. Results were expressed in mg/100 g fresh weight (FW). The quantification of components in organic extracts was described in a previous work [28].

#### 2.5.2. FT-ICR MS Analysis

A portion (1 mg) of each dried Bligh–Dyer hydroalcoholic (H) and organic (O) fraction of TF and SM cultivars was dissolved in 1 mL (1:1) methanol/water and CH_2_Cl_2_, respectively. These stock solutions were then vortexed for 3 min, filtered through a 0.45 μm polypropylene Acrodisc (Sigma–Aldrich) syringe filter to remove debris and subsequently diluted in methanol so as to obtain a final concentration of 100 μg L^−1^, a value chosen to limit ion suppression effects. For each extract, three distinct solutions prepared according to the above procedure were submitted to analysis. In positive mode MS, formic acid (1% *v/v*) was used to assist protonation, while leucine enkephalin (YGGFL, C_28_H_37_N_5_O_7_) was added to all samples at a final concentration of 0.5 µg L^−1^ as an internal reference (revealed as [M+H]^+^ at m/z 556.27657 in positive mode and as [M−H]^−^ at *m/z* 554.26202 in negative mode) to calibrate the spectra by means of the on-line calibration tool (Data Analysis 5.0, Bruker Daltonics). Further internal calibration was achieved by referring to a list of ubiquitous metabolites, including hexose/monosaccharides, citric and palmitic acids, reaching a routine mass accuracy lower than 0.2 ppm. Preliminary mass spectrometric surveys were carried out by using a Bruker BioApex Fourier transform ion cyclotron resonance (FT-ICR) [29] mass spectrometer (Bruker Daltonics GmbH, Bremen, Germany) equipped with an Apollo I electrospray ionization (ESI) source and a 4.7 T superconducting magnet (FT-ICR lab, Sapienza Università di Roma). Ultrahigh-resolution mass spectra were acquired on a Bruker SolariX XR FT-ICR MS endowed with a 7 T superconducting magnet (Magnex Scientific Inc., Yarnton, UK), a ParaCell (Bruker Daltonics GmbH, Bremen, Germany), and an APOLLO II electrospray ionization (ESI) source operated in either the positive (ESI+) or negative ionization mode (ESI-), at Universidade de Lisboa. Samples were directly infused in the ESI source at a flow rate of 120 µL h^−1^. The nebulizer gas pressure was set at 1.0 bar, the drying gas flow rate at 4.0 L min^−1^ at a temperature of 200 °C, and the capillary exit voltage at 200 V.

All MS spectra were acquired in absorption mode, over a mass range between *m/z* 100 and 3000 (resolution of 650,000 at *m/z* 400), with an acquisition size of 4 mega words, resulting in a free induction decay (FID) of 1.973 s. For each sample, two hundred scans were coadded, corresponding to a run time of 10 min.

Overall, 20 μL of dilute sample solution (corresponding to 2 ng of original dry sample) were used for acquiring one mass spectrum, which not only makes the FT-ICR MS analysis compatible for high sample throughput but also uses relatively small sample amounts.

The list of *m/z* values was exported with a cut-off signal-to-noise ratio (S/N) of 4 and submitted to the free tool MassTRIX [30], taking into account protonated, sodiated, and potassiated (ESI(+)), and deprotonated and chlorinated (ESI(−)) ions, with a maximum deviation range set to ± 1 ppm. An accurate check of the isotopic pattern based on the natural abundances of 13C, 15N, 18O, 34S, and 37Cl isotopes, was also performed to minimize false positive results. Only singly charged species were revealed, in both polarity modes. In analyzing each cultivar, peaks with a reproducibility lower than 67% were removed. A large number of unambiguous molecular formulas, for which several isomers are possible, admitting the presence of the elements C, H, O, N, P, and S, could be assigned by both ESI(+) and ESI(−) analyses and were further filtered by application of several chemical constraints as indicated by Kind et al. [31]. Additional information was obtained by acquisition of collision induced dissociation (CID) spectra, though limited to components of adequate abundance, further verified against fragmentation patterns of reference compounds or data inserted into a specialized database. The formulas generated from each sample were then transposed to two-dimensional van Krevelen diagrams, known as elemental ratio analysis, constructed by plotting the molar hydrogen to carbon ratio (H/C) vs. the molar ratio of oxygen to carbon (O/C) for each data point. According to their own characteristic H/C and O/C ratios, main classes of compounds are specifically localized as areas in the plot, thus allowing a depiction of a sample’s composition [32].

#### 2.5.3. Phenolic Compounds (Polyphenols, Tannins, and Flavonoids)

Total polyphenols, tannins, and flavonoids per milligram of fresh fruit were determined by spectrophotometric methods according to previous published methods [24]. The total amount of both polyphenols and tannins was expressed as tannic acid equivalents (TAE), while flavonoids were expressed as quercetin equivalents (QE).

#### 2.5.4. Biogenic Amines (BAs) Determination

The BAs determination was carried out as previously described [33]. Briefly, 8 g of tomato extract were added 15 mL 0.6 M HClO_4_ aqueous solution and 0.5 mL of 1,7diaminoheptane 100 mg mL^−1^ (Internal Standard), then homogenized for 3 min with an Ultra-Turrax and centrifuged at 3000 RPM for 10 min. Supernatant was filtered through a 0.20 µm membrane Millipore filter and sediment was added with 8 mL of HClO_4_ 0.6 M, mixed, and centrifuged again for 3 min. The second extract was then filtered and added to the first. The final volume was adjusted to 25 mL with HClO_4_ 0.6M. An aliquot of 1 mL of the final extract was then derivatized by adding 200 µL of NaOH 2 M, 300 µL of saturated NaHCO_3_ solution, and 2 mL of dansyl chloride solution (10 mg mL^−1^ in acetone). After shaking, samples were left in the dark at 45 °C for 60 min. The final volume was adjusted to 5 mL by adding acetonitrile. The dansylated extract was filtered using 0.22 µm (Polypro Acrodisc, PallGelman Laboratory, USA) filter, injected into the chromatograph, and analyzed with a previous standard method [34]. The determination was carried out twice on 8 samples of each examined tomato cultivar.

### 2.6. Screening of Biological Activities

#### 2.6.1. Antioxidant Activities

All tests were performed in 96-multiwell microplates away from direct light. To perform the assays, the extracts were assayed at the concentrations of 1, 10, 25, 50, 100, 250, 500, 1000, 1500, 2000, and 5000 µg mL^−1^ in order to achieve a concentration-response curve. The samples were dissolved in 50% or 100% *v/v* EtOH (organic and hydroalcoholic extracts, respectively). The experiments were repeated at least twice, and in every experiment, each concentration was tested in triplicate. Data obtained from at least two experiments were pooled for the statistical analysis.

In each experiment, the vehicle (negative control) and standard antioxidants (positive controls), i.e., trolox (assayed concentrations 0.1, 0.25, 1, 5, 10, 50, and 100 µg mL^−1^) for the radical scavenger and reducing activity, and quercetin (assayed concentrations 1, 5, 10, 50, 100, and 200 µg mL^−1^) for the chelating activity, were included too. The absorbance was measured by a microplate reader (Epoch Microplate Spectrophotometer, BioTeK^®^ Instruments Inc., Winooski, VT, USA). Some wells containing only the test samples were also included to determine its possible absorbance.

Scavenging activity towards DPPH and ABTS radicals was determined according to the methods of Di Sotto et al. [35]. Furthermore, the ability of the extracts to indirectly interfere with the ROS-generation through blocking the Fenton reaction was evaluated by testing the iron chelating and reducing activities in the ferrozine assay [35]. Chelation ability was evaluated against both ferrous and ferric ions. The ability of the samples to inhibit the ROS-induced lipid peroxidation was assessed by the ferric thiocyanate method [36].

#### 2.6.2. Advanced Glycation End-Product (AGE) Inhibition

The ability of the tested samples to inhibit the AGE formation was measured through the method of Di Sotto et al. [37]. The phenolics naringenin and rutin were included as standard inhibitors, while the vehicle (50% or 100% *v/v* EtOH for organic and hydroalcoholic extracts respectively) represented the lack of inhibition. The inhibitory activity was calculated as percentage of the control, as follow:

(A_control_ − A_sample_/A_control_) × 100 (1)

where A_control_ is the fluorescence of the control, whereas A_sample_ is the fluorescence of the sample. Data from at least three replicated experiments (including six replicates for experiment) were pooled for the statistical analysis.

#### 2.6.3. Cytoprotection Towards the Oxidative Stress Induced by tBuOOH

Cytoprotective activity of the tested extracts was evaluated towards the oxidative damage induced by tert-butyl hydroperoxide solution (tBuOOH) in HepG2 liver cancer cells (American Type Culture Collection, Milan, Italy). The cells were grown at 37 °C in 5% CO_2_ in Dulbecco’s modified Eagle’s medium, supplemented with fetal bovine serum (10% *v/v*), glutamine (2 mM), streptomycin (100 µg mL^−1^), and penicillin (100 U mL^−1^) [38]. All experiments were performed when cells reached the logarithmic growth phase.

Preliminarily, the extracts (1–1000 µg mL^−1^ concentration range) were tested for the mitochondrial cytotoxicity by the 3-(4,5-dimethylthiazol-2-yl)-2,5-diphenyl tetrazolium bromide (MTT) assay [39], in order to define the proper concentrations to be used in the subsequent experiments. Then, 50% or 100% *v/v* EtOH were used as vehicle for organic and hydroalcoholic extracts respectively; the vehicle was nontoxic at final concentration of 1% *v/v* in the medium.

The ability of the tested samples to counteract the oxidative stress induced by tBuOOH was evaluated by measuring the levels of intracellular ROS (reactive oxygen species) through the 2,7-dichlorofluorescein diacetate assay (DCFH-DA) [40]. To this end, 5 × 10^5^ cells were grown into 6-well plates for 24 h, then treated with a nontoxic concentration of the extracts (100 μg mL^−1^) for 24 h. At the end of incubation, the cells were treated with a low-toxic concentration (about 40% cytotoxicity as found in preliminary experiments) of the pro-oxidant agent tBuOOH (5 mM) for 2 h, then washed twice with Hank’s Balanced Salt Solution (HBSS) (1×) and added with DCFH-DA (10 µM; 6 μL). Fluorescence of DCF, obtained by DCFH-DA oxidation, was measured though a BD Accuri™ C6 flow cytometer at an excitation wavelength of 485 nm and emission wavelength of 528 nm. In each experiment, proper treatment with the vehicle control (corresponding to the basal ROS level) and the pro-oxidant agent tBuOOH were included too; furthermore, the extracts alone were assayed to evaluate their effect on the basal ROS levels, released as a consequence of cell metabolism. The oxidation index was obtained by the ratio between the DCF fluorescence of the sample and vehicle control.

#### 2.6.4. In Vitro Metabolic Enzyme Inhibition

The ability of the tested extracts to inhibit in vitro the α-amylase and α-glucosidase enzymes was measured by dinitrosalicilic acid (DNSA) and *p*-nitrophenyl-α-D-glucopiranoside (PNGP) methods described by Di Sotto et al. [37]. Acarbose was included in all the experiments as standard enzyme inhibitor (100% enzyme inhibition), while the vehicle (50% or 100% *v/v* EtOH for organic and hydroalcoholic extracts respectively) represented the maximum enzyme activity. Additional treatments, in which enzyme solution was replaced by buffer solution, were included to evaluate a possible interfering absorbance of the samples. The experiments were performed at least in triplicate and in each experiment about six replicates were prepared. Data obtained from at least two experiments were pooled in the statistical analysis. The inhibitory activity was calculated as percentage of inhibition with respect to the vehicle control.

#### 2.6.5. Antifungal Susceptibility Test

To evaluate the minimal inhibitory concentration (MIC) of the extracts, the broth microdilution method was performed according to a standardized method for yeasts [41].

The assay was carried out with four *C. albicans* strains (ATCC10231, ATCC24433, 3153A, PMC1033), three *C. glabrata s*trains (PMC0822, PMC0851, PMC0807), and two *C. krusei* strains (PMC0631, PMC0624). *Candida* spp. strains were grown on Sabouraud dextrose agar at 37 °C for 24 h. Then, cell suspensions of the strains were prepared in RPMI 1640 medium buffered to pH 7.0 with 0.165 mM MOPS. The final concentration of the inoculum was 1 × 10^3^–5 × 10^3^ cells mL^−1^. The extracts were dissolved in DMSO and diluted 100 times in RPMI-1640 broth. Ten concentrations ranging from 1000 to 1.9 μg mL^−1^ were tested against *Candida* spp. strains in 96-well round-bottom microtitration plates. The antifungal activity is the result of four independent experiments. The MIC_50_, MIC_90_, and MIC_100_, the lowest concentrations of extracts that caused growth inhibitions ≥50%, ≥90%, and 100% respectively, were evaluated. Data were reported as range and geometric mean (GM) of MIC.

### 2.7. Statistical Analysis

All values are expressed as mean ± standard error (SE). Statistical analysis was performed by GraphPad Prism™ (Version 4.00) software (GraphPad Software, Inc., San Diego, CA, USA). The one-way analysis of variance (one-way ANOVA), followed by a suitable multiple comparison post hoc test (i.e., Bonferroni post-test for comparison among means, while Dunnett’s post-test for estimating a difference compared to the control), was used to analyze the difference between treatments. The concentration–response curves were constructed using the “Hill equation”:

E = Emax/(1 + 10^(LogEC_50_/A) × HillSlope) (2)

where E is the effect at a given concentration of agonist, Emax is the maximum activity, EC_50_ is the concentration that produces a 50% of the inhibitory response (namely IC_50_), A is the agonist concentration in molarity, HillSlope is the slope of the agonist curve. *p* values < 0.05 were considered as significant. Correlation between two variables was evaluated by the Pearson correlation coefficient and the statistical significance was measured by the two-tailed *t*-test.

## 3. Results and Discussion

### 3.1. Morphological and Pigments Analyses

The sampled TF tomatoes showed a shape similar to SM fruits, but were smaller in length and circumference (about two-fold lower) and in weight (about five folds lower), thus supporting their nature of “Mini-San Marzano tomato” (Table 3). Despite a smaller size, peel amount in pink and red TF fruits was two- and ten-fold higher than those of SM at the same ripening stages, respectively (Table S1). Furthermore, at least a doubled peel amount was found in the red TF tomatoes compared to the pink ones, whereas an opposite trend was observed in SM fruits (Table S1).

Ripeness also increased the pulp amount in both varieties. The TF pulp content per gram of fruit was at least four-fold higher with respect to SM tomatoes (Table S1). In spite of a lower pulp amount, both pink and red SM (SM_P_, SM_R_) tomatoes contained high amounts of juice (pH 4.0–4.2). Conversely, the juice content was at least two to six-fold lower in pink and red TF (TF_P_, TF_R_) tomatoes (Table S1). Altogether, these features support the claimed crunchy and solid consistency of TF tomatoes, likely ascribable to a high peel and pulp content, despite a significant low amount of juice.

A significant difference in the seed number and weight per gram of fruit was observed, which was about four-fold higher in TF compared to SM tomatoes at both ripening stages (Table S1). Conversely, the size and weight of each seed were similar in both varieties, with a slight increase in the SM_R_ fruits (Table S1). It is widely accepted that seed size and number are strictly linked to the plant reproductive potential. Small seeds were reported to possess lower reproductive capacity, due to a lower endosperm amount, which limits seedling survivorship and competitive ability [42]. On the other hand, a high seed number improves the competitive ability of the plant, due to the increased probability of seedling survivorship. In this context, TF tomatoes seem to possess a higher reproductive competition related to SM, along with similar plant reproductive capacity and survival.

The color of SM tomatoes varied from pale green in the pink fruits to light red in the red ones (Table 3 and Figure S1). The analysis of pigments i.e., chlorophyll a, chlorophyll b, and total carotenoids, carried out on the TF and SM organic extracts from peel and pulp justified these features. The dark-green colour of TF_P_ tomatoes can be ascribed to the higher content in chlorophylls a and b in peel (+70% and +52% higher amount, respectively) (*p* < 0.01) and in pulp (+10% and +82% higher amount, respectively) (*p* < 0.01) with respect to TF_R_ fruits (Table 4). On the contrary, carotenoids content (bright red color) was found about five-fold higher in TF_R_ peel and pulp than in pink ones (*p* < 0.001) (Figure S1).

The difference in chlorophyll a content between pink and red fruits was particularly marked in SM cultivar, where chlorophyll a amount was 90% higher in peel and pulp of SM_P_ compared to SM_R_ fruits (*p* < 0.01). SM_P_ tomatoes displayed both chlorophyll a and b content to be double in peel with respect to pulp, while in SM_R_ fruits, this difference was not evident (Table 4). The opposite trend was observed with regard to carotenoids content. SM_R_ peel showed a carotenoid amount 28 times higher with respect to SM_P_ peel (*p* < 0.001), while in SM_R_ pulp, a 40 times higher level of total carotenoids with respect to SM_P_ pulp (*p* < 0.001) was found.

TF fruits were characterized by higher levels of chlorophylls compared to SM ones at both ripening stages (*p* < 0.01). In particular, chlorophyll a and b content was found to be 60% and 80% higher in TF_P_ peel and pulp fruits than in SM_P_ peel and pulp, respectively (*p* < 0.01). A similar trend was observed in terms of total carotenoids content, which was 68% higher in TF_P_ fruits with respect to SM_P_ (*p* < 0.01). These findings agree with the different shade of green color observed in the TF_P_ (dark green) compared to SM_P_ fruits (pale green) (Figure S1). In addition, red fruits of both cultivars were characterized by different pigments proportion. In TF_R_ peel, chlorophyll levels were 90% higher compared to SM_R_ peel (+90%, *p* < 0.001), the latter showed 40% higher content of carotenoids than TF_R_ peel (*p* < 0.001; Table 4).

Carotenoids are mainly responsible for the red color of tomatoes and are involved in the fruit protection against excessive sun irradiation and harmful UV rays. Total carotenoids amount in fruits depends on the ripening stage and other factors, such as cultivar, climate, sun exposure, agronomic practices, irrigation. The ratio of chlorophyll a and b to total carotenoids (a + b/total carotenoids) can be considered as an indicator of tomato ripening stage. During chromoplast development in fruit maturation, the ratio a + b/total carotenoids tends to decrease continuously, thus reaching a value below 1.0 [43]. Our findings confirmed the differences in ripening stages selected for the study, revealing a ratio value of 3.16 in TF_P_ tomatoes and 0.18 in TF_R_ ones. In SM fruits, this trend was even wider, in fact, pink fruits showed a ratio value of 3.9, whereas in red fruits, this ratio was 0.02 (far below of 1).

### 3.2. Metabolite Profiling

FT-ICR MS and NMR untargeted analyses were carried out for a thorough metabolite profile characterization of TF and SM in relation to their pink and red states. The high mass accuracy typically achieved with FT-MS implies that elemental formulas can be determined, pertaining to a large number of metabolites, based on their accurate mass, whereas the NMR capacity of structural determination allows the unambiguous compound identification and quantification.

The ESI FT-ICR MS analysis of both Bligh–Dyer hydroalcoholic and organic fractions of TF_P_, TF_R_, SM_P_, and SM_R_ fruits has allowed to detect both polar and non-polar metabolites. Each sample was analyzed in both positive and negative ionization mode (Figures S2–S5), detecting up to 1138 molecular formulas; however, a larger number of compounds were detected in positive ionization mode (Table 5). Overall, the TF cultivar was characterized by a smaller number of compounds with respect to SM and the ripeness process promotes a general increase in the number of putatively identified metabolites (Table 5). An overview of all the recorded plausible compounds is available in Tables S2 and S3.

**Figure 1:**
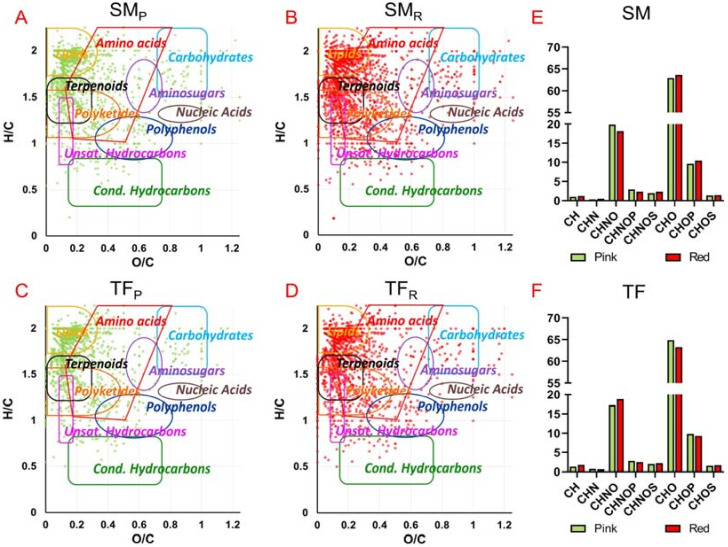
Van Krevelen plot (elemental plot) obtained from the molecular formulas obtained by ESI FT-ICR MS analysis of total hydroalcoholic and organic fractions of: (**A**) SM_P_; (**B**) SM_R_; (**C**) TF_P_; (**D**) TF_R_. Histograms of the relative frequency of CH, CHN, CHNO, CHNOP, CHNOS, CHO, CHOP, CHOS compounds: (**E**) in SM_P_ (green), SM_R_ (red); (**F**) TF_P_ (green) and TF_R_ (red).

Specific data analysis allows to organize the vast amount and complexity of detected formulas to uncover interesting information. Among the detected molecular formulas, the relative frequency distribution was investigated (Figure 1E,F) showing that all tomato extracts contain a majority of CHO species followed by CHON, CHOP and, in smaller amount, CHNOP and CHNOS. In particular, CHO components correspond mainly to polyphenols (more hits in SM_R_ extracts), steroids (more hits in TF), and fatty acids (more entries in TF), followed by di- and tri-glycerides (more entries in SM_P_), terpenoids, organic acids, and arachidonic derivatives (Supplementary Figure S6A). When considering CHON components, they can be ascribed mainly to amino fatty acids, amino-sugars, amines (more hits in red extracts), N-acylamines (more hits in TF_P_), followed by amino acids (more entries in pink extracts), solanidines, nucleosides (more hits in SM_P_), and vitamins (more hits in TF_R_) as shown in Figure S6B.

Van Krevelen diagrams were used to classify the detected molecular formulas in different classes of natural compounds such as lipids, terpenoids, carbohydrates, amino acids, aminosugars, nucleic acids, polyphenols, polyketides, unsaturated hydrocarbons and condensed hydrocarbons (Figure 1A–D). TF and SM tomato extracts showed marked similarities, covering several classes of metabolite families. A relatively higher compound density is present in the area of lipids, terpenoids, and polyketides, followed by components in the areas of amino acids, unsaturated hydrocarbons, polyphenols and (relatively less) in the regions of carbohydrates, aminosugars, nucleic acids, and condensed hydrocarbons (Figure 1A–D).

Moreover, Venn diagrams (Figure S7) pointed out possible similarities and differences in the metabolic profile of the sampled TF and SM fruits. The combined pattern of hydroalcoholic and organic fractions of pink and red extracts of the two cultivars showed that overall only 19% of the molecular formulas were found to be common, thus suggesting a noticeable extent of chemical diversity, whereas more than 40% of molecular formulas were shared between pink and red samples of each variety.

This wide metabolomics survey supported the untargeted and targeted analyses driving the identification of selected classes of metabolites. In particular, 1D NMR spectra assignment of the TF and SM hydroalcoholic extracts solubilized in D_2_O phosphate buffer and organic extracts solubilized in CDCl_3_/CD_3_OH (Table 2) were obtained by means of literature data [28,44,45,46]. Furthermore, targeted analytical approaches provided the identification and quantification of total polyphenols, tannins, and flavonoids content and BAs.

Results will be presented and discussed according to compound classes.

#### 3.2.1. Amino Acids and Derivatives

NMR spectra of both red and pink TF and SM hydroalcoholic extracts showed signals of sixteen amino acids, namely leucine, valine, isoleucine, threonine, alanine, GABA, glutamic acid, glutamine, aspartic acid, asparagine, lysine, arginine, tyrosine, phenylalanine, tryptophan, and histidine, as also confirmed by ESI FT-ICR MS. All of them were quantifiable, except arginine. In addition, ESI FT-ICR MS revealed the presence of proline, serine, the non-essential amino acid citrulline, and other amino-acids-related metabolites, like hydroxyproline and phosphoserine. Some peptides were also found. In particular, glutathione was detected in all hydroalcoholic extracts, S-nitrosoglutathione in red fruits, glutathione disulfide in pink fruits.

Alanyl-alanine (in hydroalcoholic SM_P_), glycyl-proline (in organic SM_R_), glycyl-leucine (in organic TF_R_), glutamyl-valine and glutamyl-glutamine (in hydroalcoholic SM_R_) were also revealed.

**Figure 2:**
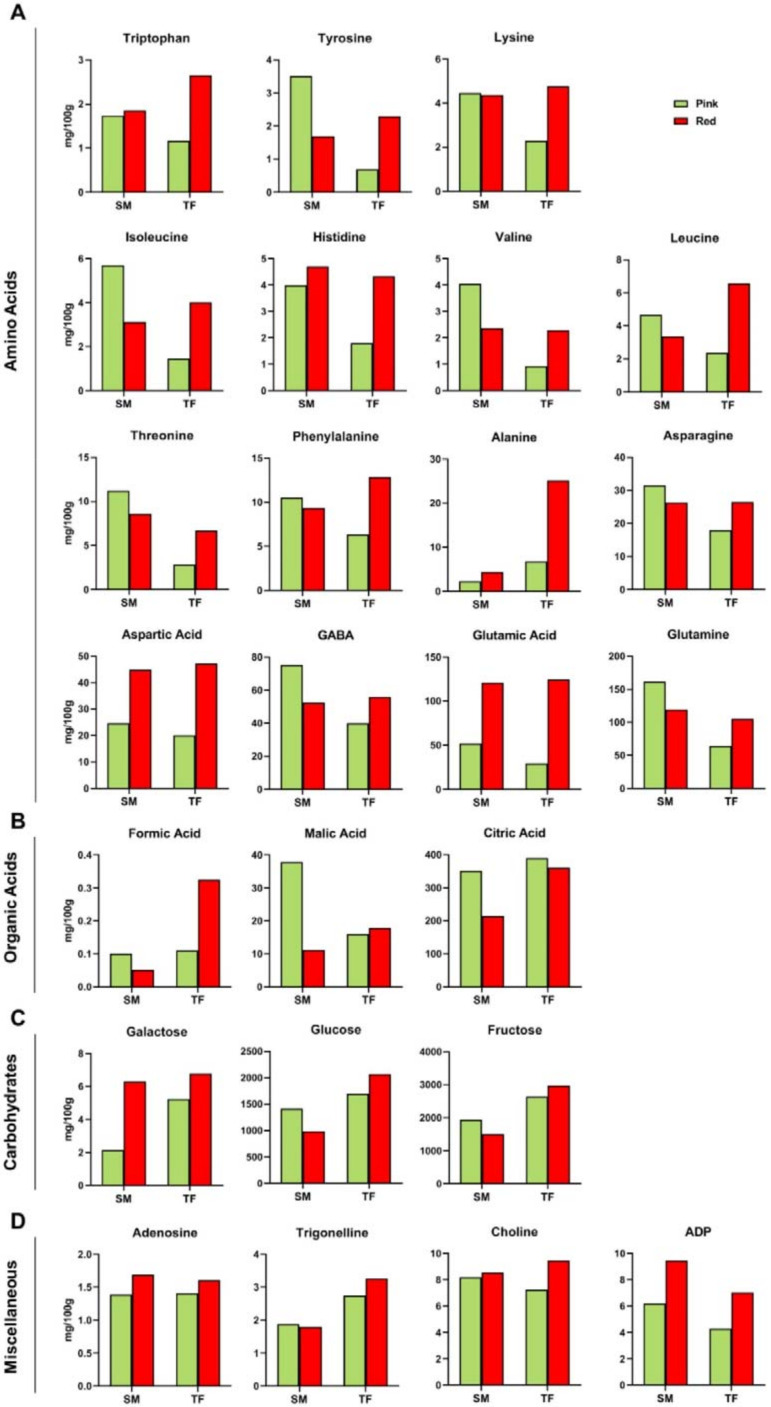
Histograms relative to metabolites identified by H^1^-NMR spectroscopy in tomato hydroalcoholic extracts from pink (green) and red (red) SM and TF cultivars: (**A**) Amino acids; (**B**) Organic acids; (**C**) Carbohydrates; (**D**) Other compounds. Data are expressed as mg/100 g FW.

According to NMR scrutiny, TF and SM samples showed some/several similarities (Figure 2A): glutamine was found to be the most abundant amino acid in both cultivars at the pink stage, followed by GABA and glutamic acid, whereas, at the red stage, glutamic acid increased, becoming the most abundant amino acid. The pattern of SM developmental changes in free amino acid content was in agreement with literature data, being glutamic acid characterized by a remarkable increase in all ripe fruits [2,3,4,45,47,48,49,50,51,52]. Glutamic acid, aspartate, tryptophan, and alanine rose upon the fruit ripening; asparagine and phenylalanine turned out to be constant; tyrosine, isoleucine, valine, threonine, GABA, and glutamine content decreased from pink to red SM fruits. These findings reflect data reported in literature about the analyses of SM [2,4] and other cultivars [3,45,47,48,49,50,51,52].

Interestingly, the TF fruit ripening showed a peculiar trend in the amino acid profile, being characterized by an increase in the content of all the amino acids from the pink to red stage. In particular, except for asparagine, GABA, and glutamine, the content of the remaining twelve amino acids rose more than two-fold.

#### 3.2.2. Organic Acids

Ascorbic, citric, chlorogenic, malic and formic acids were detected by NMR analysis. Chlorogenic acid (5-caffeoylquinic acid) was identified only in hydroalcoholic TF_P_ extracts. The ESI FT-ICR mass spectra in negative mode provided additional peaks corresponding to deprotonated organic acids identified in one or a few samples, like succinic and glutaric (in organic SM_R_ and hydroalcoholic TF_P_ extracts), maleic (in hydroalcoholic TF_P_ extracts), quinic and shikimic (in hydroalcoholic TF_R_ and TF_P_ extracts), and lactic (absent only in red hydroalcoholic samples) acids, as the most prominent signals. In addition, sugar esters of caffeic and ferulic acids, caffeoyl- and feruloyl-hexose, were revealed, with both metabolites being present in hydroalcoholic extracts. Although the present method does not allow to recognize which constitutional isomer is formed, glycosylated forms of phenolic acids have been previously identified in methanol extracts of tomato fruit by a HPLC/DAD/MS approach [53].

Histograms reporting NMR data (Figure 2B) showed that formic acid was always present in a minor amount, whereas citric acid represented the main organic acid, contributing to sourness [5] and confirming literature data [2,3,4,45,47,48]. During the developmental process, both malic and citric acids contents stayed constant in TF fruits and decreased in SM tomatoes. Previous results concerning the organic acid trends are contradictory: Mounet et al. [48] and Jezequel et al. [54] described an increasing citric acid content from the pink to red stage, whereas Perez et al. [45] found out a decrease in citric and malic acids content. These differences might be ascribed to the combination of genetic, pedoclimatic, seasonal, and agronomic factors.

In terms of cultivar, the three organic acids were comparably abundant at the pink stage, except for malic acid which was significantly higher in SM than in TF fruits. At the red stage, formic, malic and citric acids contents were found nearly doubled in TF compared to SM fruits.

#### 3.2.3. Sugars

Fructose, glucose and galactose were the monosaccharides identified in the ^1^H NMR spectrum of hydroalcoholic extracts. Besides, confirming the widespread incidence of mono and disaccharides, ESI FT-ICR results reported an Amadori compound, fructosyl lysine, in all hydroalcoholic extracts, providing a sensitive marker of early modifications in food nutrients previously described also in unprocessed tomato extracts [55].

Fructose was the most abundant sugar in both cultivars at both ripeness stages, followed by glucose; however, higher levels of both carbohydrates were found in TF compared to SM fruits (Figure 2C). Fructose, glucose, and galactose levels increased in TF cultivar over the ripening period, whereas the opposite trend was observed in SM fruits. Loiudice et al. [2] reported glucose and fructose contents of eleven SM tomato cultivars at three harvesting years: fructose was always found the most abundant sugar in all samples characterized by a mean content of 1.4g/100 g, whereas glucose mean content was 1.2 g/100 g. These data are consistent with the values reported in Figure 2C: fructose was the main sugar in SM with a mean value of 1.7 g/100 g and glucose content was 1.2 g/100 g, with a total amount of 2.9 g/100 g. However, sugars content in hybrid cultivars tended to increase [2], confirming the higher amount of both fructose and glucose in TF, which reached 5 g/100 g as mean value.

#### 3.2.4. Other Compounds

NMR signals of choline, trigonelline, uridine, adenosine, and ADP were identified and, except for uridine, quantified (Figure 2D). A rich variety of miscellaneous compounds were detected by ESI FT-ICR analysis, comprising small amines (serotonin), nucleosides (adenosine, methylthioadenosine, orotidine), and nucleotides (guanosine-, cytidine-, and uridine-monophosphate), sugar alcohols (sorbitol, mannitol), sugar acids (galactonic and glucuronic acids), aminosugars (glucosamine, lactosamine), terpenes (*p*-cymene, caryophyllene, limonene), terpenoid (apiole, oxo-campholide), vitamins and derivatives (ascorbic acid, retinol, α-tocopherol). As expected, both cultivars contained several key secondary metabolites characteristic of tomato fruits, including alkaloids (trigonelline, narciclasine, and catharanthine, only in hydroalcoholic extracts, nicotine and sauroxine, spread in all samples), polyketides such as lycoflexine, mostly in pink fruits, and glycoalkaloids as tomatine and tomatidine, only in pink hydroalcoholic extracts [56,57]. Several phytohormones, recognized as key signaling molecules, were observed in most samples, like derivatives of abscisic acid, only in hydroalcoholic extracts, jasmonic acid, only in red hydroalcoholic fractions, and salicylic acid, mostly in TF_P_ and SM_R_, and several gibberellins, mainly detected in red organic samples [58].

The concentration of choline and adenosine were comparable in SM and TF fruits, slightly increasing at the red stage, whereas trigonelline content was found to be lower in SM with respect to TF at both developmental stages. ADP content severely increased in both cultivars at the red stage, almost doubling its level.

The presence of hydroxyl-substituted fatty acids, metabolites with strong anti-inflammatory and antioxidative effects, confirms the nutraceutical potential of tomato. Hydroxy-stearic acid was observed in all samples except for organic SM_R_, hydroxy-linoleic acid, in all extract excluding hydroalcoholic SM_P_, and hydroxylinolenic acid, in organic SM_R_ and all TF extracts [59].

#### 3.2.5. Phenolic Compounds (Polyphenols, Tannins, and Flavonoids)

Spectrophotometric targeted analyses provided the total content of phenolic compounds expressed as polyphenols, tannins, and flavonoids. Highest levels of total polyphenols were found in hydroalcoholic extracts from pink fruits of both cultivars, being TF_P_ the most enriched sample (triple content compared SM_P_); conversely, their levels were reduced of about 14- and 6-fold with ripeness (Table 6). Similarly, a 1.6-fold reduction in total polyphenols was found in TF_R_ organic extracts with respect to those from TF_P_, whereas an opposite trend occurred in the SM variety (Table 6).

TF_P_ and SM_P_ hydroalcoholic extracts contained similar levels of tannins, which slightly increased with ripening (Table 6). Comparing the ripening stages, both SM_P_ and SM_R_ organic extracts contained an analogue content of tannins, whereas a marked 8-fold reduction in their levels occurred in TF_R_ organic extracts compared to TF_P_ (Table 6).

Flavonoids were mainly concentrated in the hydroalcoholic extracts of both TF and SM tomatoes at both the ripening stages, although high levels were also found in the organic extracts of TF_P_ and SM_R_ fruits (Table 6). TF_P_ hydroalcoholic extract resulted in the most enriched sample in flavonoids, being three times more concentrated than SM_P_; similarly, the same trend was observed in pink organic fractions, being TF the cultivar characterized by an almost 30-fold higher flavonoid content compared to SM (Table 6). At the red stage, TF fruits showed a flavonoid reduction of about 1.5- and 4-fold in organic and hydroalcoholic extracts respectively, whereas an opposite trend was registered for SM tomatoes. Indeed, a 2- and 66-fold flavonoid increase was found in the hydroalcoholic and organic SM_R_ with respect to SM_P_ (Table 6). This evidence revealed that the highest levels of total polyphenols, tannins, and flavonoids were concentrated in the TF_P_ tomatoes, although a high flavonoid content was retained in TF_R_ and SM_R_ fruits. Our data agree with previous evidence that highlighted a total flavonoid content of 200 μg/g (calculated as quercetin equivalents) in SM_R_ tomatoes [60]. Conversely, to the best of our knowledge, no comparison data are available in the literature regarding TF landrace.

In addition, ESI FT-ICR MS experiments have revealed flavanols (dihydroxy-methoxy-isoflavanol), flavan-3-ols (epigallocatechin sulfate), flavonoids (apigeniflavan, tetrahydroxyflavanone glucoside), and polyphenol derivatives like catechin-O-glucoside, catechin-O-rutinoside, dihydrokaempferol, trihydroxy-prenyldihydrochalcone glucosyl-coumarate, quercetin glucoside-glucuronide [61].

#### 3.2.6. Sterols

β-Sitosterol and stigmasterol were detected and quantified by NMR analysis in the organic extracts of both cultivars at pink and red developmental stages.

**Figure 3:**
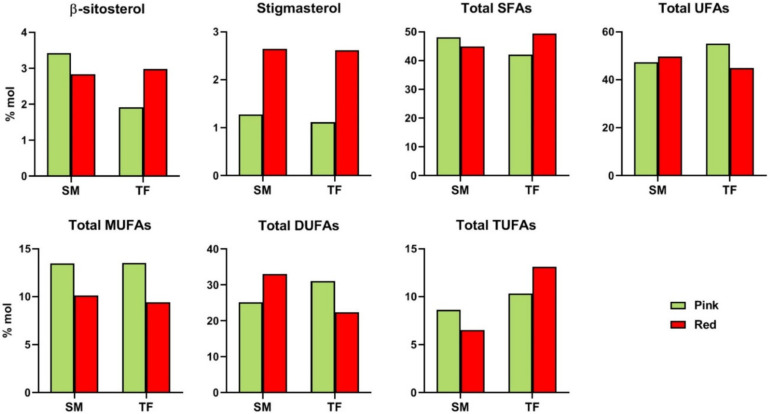
Histograms comparing the concentration (% molar) of metabolites present in tomato organic extracts from pink and red SM and TF. SFA: saturated fatty acids; UFA: unsaturated fatty acids; MUFA: mono-unsaturated fatty acids; DUFA: di-unsaturated.

β-Sitosterol content showed a decreasing trend in SM_R_ fruits, opposite to TF fruits. Conversely, stigmasterol content significantly increased (3-fold higher) over the ripening stage in both cultivars (Figure 3). According to ESI FT-ICR results, cholesterol- and hydroxycholesterol-sulfate were found in all samples, whereas methylstigmasterol was detected in the hydroalcoholic TF_R_ sample.

#### 3.2.7. Fatty Acid Chains

NMR analysis allowed the identification and quantification of the total saturated (SFA) and unsaturated (UFA) fatty acid chains, the latter ones including mono- (MUFA), di- (DUFA) and tri-unsaturated (TUFA) fatty acid chains. The amount of the selected metabolites was comparable in SM and TF cultivars at both ripening stages, except for the case of TUFA, more abundant in TF fruits (Figure 3). Regarding the ripening stages, total UFA content was found to slightly increase in SM fruits and decrease in TF cultivar, conversely total SFA showed the opposite trend.

A drop from pink to red fruits was noticed in MUFA (both SM and TF), DUFA (TF), and TUFA (SM) content, whereas an opposite trend/increase with the ripening stage emerged DUFA (SM) and TUFA (TF) amount.

**Figure 4:**
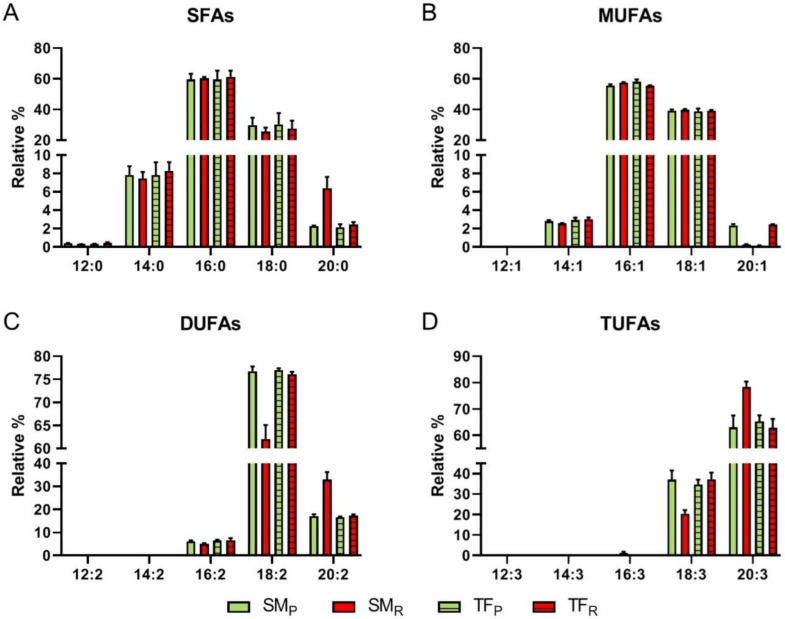
Histograms of the relative abundance distribution within specific classes of FA: saturated (**A**); mono-unsaturated (**B**); di-unsaturated (**C**); tri-unsaturated (**D**) obtained by ESI(−) FT-ICR MS analyses of organic SM_R_ (red), SM_P_ (green), TF_R_ (red), TF_P_ (green) extracts.

Despite untargeted investigation, direct infusion ESI FT-ICR MS analysis delivers consistent solution composition and maximum metabolome coverage, ion-suppression effects and differences of signal response are a concern in view of an accurate quantitation. However, careful tuning of experimental conditions has recently allowed a successful quantification of numerous isomeric groups of intact wax esters, where relative ionization efficiency was found to be influenced only by lipid class and saturation degree, while independent on carbon chain length [62]. On this basis, the abundances of molecular formulas classified as free fatty acids (FA) and presenting the expected (CH_2_)_2_ increments were obtained from the lists of organic extracts in the negative ionization mode, relatively richer in lipids (Table S4). Then, these values were summed up within each specific class, namely saturated SFA, containing the series 12:0-20:0, MUFA, with the series 14:1–20:1, DUFA and TUFA, including compounds 18:2, 20:2, 18:3, 20:3, 18:4, 20:4, respectively, to evaluate their relative abundance. Notably, four FAs at *m/z* 227, 255, 277, and 279 have been assigned to myristic (14:0), palmitic (16:0), linolenic (18:3), and linoleic (18:2) acids, respectively, on the basis of their characteristic fragmentation in CID experiments. As shown in Figure 4, an overall similar composition is highlighted in both organic SM_R_ and TF_R_ extracts, with the highest percentages found for: (i) 16:0 (ca. 60%) and 18:0 (ca. 28%) among SFAs; (ii) 16:1 (ca. 58%) and 18:1 (ca. 39%) among MUFAs; (iii) 18:2 (ca. 77%) and 20:2 (ca. 17%) among DUFAs; (iv) 18:3 (ca. 37%) and 20:3 (ca. 64%), among TUFAs.

The presence and chemical diversity of long chain fatty acids pointed out in both cultivars is intriguing, since these compounds are known to have a wide range of biological properties, including the promotion of type 2 immune responses [63].

Noteworthy, SM_R_ organic fraction contains both the lowest percentage of relatively shorter chain di- and tri-unsaturated FA, including 18:2 (ca. 62% vs. the above reported value of 77%) and 18:3 (ca. 21% vs. 37% above reported), and the highest amount of longer chain FA, including 20:2 (ca. 33% vs. 17%) and 20:3 (ca. 78% vs. 64%).

The availability of appropriate unsaturated fatty acids is reported as a significant factor responsible for specific fruit flavor and aroma development, due to the action of lipase enzymes that may release a rich milieu of metabolites from acyl lipids during the ripeness processes.

#### 3.2.8. Biogenic Amines

High performance liquid chromatography allowed to detect seven BAs (Table 7), namely putrescine (PUT), cadaverine (CAD), histidine (HIS), serotonin (SER), spermidine (SPD), and spermine (SPM). All BAs investigated are found in tomato samples, except for tyramine (TYM), an amine with negative health effect, which was not detected in any samples. By contrast, HIS, another BA with negative health effect, was detected in all samples. Histamine presence is regulated in some food, but none in tomatoes. The tolerance presence of histamine in wine and fish is up to 100 μg/g [64]. The maximum HIS level was found in TFR at 1.463 ± 0.015 μg/g, far less than the regulation limits (Commission Regulation (EU) No 1019/2013 of 23 October 2013 amending Annex I to Regulation (EC) No 2073/2005 as regards to histamine in fishery products.) For these reasons, these tomato cultivars appear to be safe for human health. The rest of BAs are usually related to cultivar and storage condition of samples. Indeed, a great variability of BAs concentration was found between SM and TF. In all the tomatoes, SER, an important neurotransmitter, was also detected in high concentration. This BA has positive effects on human health; SER plays an important role in regulating mood, sleep, body temperature, sexuality, and appetite. SER is involved in many neuropsychiatric disorders such as migraine, bipolar disorder; serotonin deficiency causes obsessive-compulsive disorder, repetition, and mania. So, its assumption by diet is highly recommended [65]. About the different level of the BAs in pink with respect to red tomatoes, is possible to highlight that in almost all samples, the highest concentration was found in red fruits (Table 7). This is in accordance with previously studies which demonstrated the accumulation of BAs during ripening of meat [66] or dairy products [67]. Finally, based on the obtained data, BAs could be also used as ripening markers of tomatoes.

### 3.3. Screening of Biological Activities

#### 3.3.1. Antioxidant Activities

The radical scavenging properties of the tested extracts were evaluated against the synthetic chromogenic DPPH and ABTS radicals. Under our experimental conditions, all extracts (1–5000 μg mL^−1^) were able to counteract the ABTS radical, despite a weak radical scavenger activity against DPPH, which achieved a lower than 40% inhibition at the highest tested concentration, thus hindering the IC_50_ evaluation (Figure S8). As expected, the positive control trolox (concentration range of 1–100 µg mL^−1^) was found to be a potent scavenger of both DPPH and ABTS (Figure S8). The measurable IC_50_ values for the extracts and Trolox were displayed in Table 8.

Comparing the tomato varieties at the pink developmental stage, TF_P_ organic extract displayed the most potent ABTS scavenging activity, being the IC_50_ value about 2-, 3-, and 4-fold less than that of TF_P_ hydroalcoholic, and both SM_P_ organic and hydroalcoholic fractions, respectively (Table 8). Similarly, TF_R_ organic extract was the most effective scavenging sample from red fruits, followed by TF_R_ hydroalcoholic extract, although with a 3-fold lower potency compared to the TF_P_ sample (Table 8). Conversely, SM_R_ organic and hydroalcoholic extracts produced a lower than 50% ABTS inhibition at the highest tested concentrations, thus hindering the evaluation the IC_50_ value (Table 8).

ABTS and DPPH radicals are scavenged by electron- or hydrogen-transfer mechanisms, although with a different specificity and kinetic profile [68]. ABTS usually reacts with both lipophilic and hydrophilic compounds and possesses a poor selectivity in the reaction with hydrogen-atom donors; conversely, DPPH is more selective for small molecules, likely due to the limited steric accessibility of the radical site to larger compounds [68]. ABTS assay has been also reported to better estimate the antioxidant power of fruits and vegetables rich in hydrophilic, lipophilic, and high-pigmented antioxidant compounds compared to DPPH assay [69]. Particularly, carotenoids seem to not react with DPPH, while being able to bleach ABTS [70].

On the base of this evidence, the scavenging abilities of TF and SM extracts towards ABTS radical can be ascribed to the presence of both hydrophilic and lipophilic antioxidant phytochemicals. Among them, the antioxidant contribution of polyphenols, carotenoids, tocopherols, and vitamins C and E to the ABTS scavenging properties of tomato fruits has been previously hypothesized [71]. Further studies are required to clarify their involvement in the radical scavenging activity of TF and SM extracts.

When assessed in ferrozine assay, all samples exhibited a weak chelating activity of ferrous ion; conversely, the hydroalcoholic extracts of TF and SM fruits were able to chelate ferric ions (Table 8), being hydroalcoholic TF_P_ and SM_P_ extracts the most potent (IC_50_ values about two-fold lower than that of the corresponding red extracts). The positive control quercetin resulted to be about two- and four-fold more potent than the tested extracts (Table 8).

Despite a marked ferric chelating activity, the hydroalcoholic samples were ineffective as reducing agents; conversely, the organic extracts significantly reduced ferric ions, being that from TF_P_ the most potent (Table 8). According to the Pearson analysis, a significant correlation occurs between the ABTS scavenger power of TF hydroalcoholic and organic extract and the respective chelating and reducing activities (correlation coefficient r of 0.81 and 0.95, respectively).

**Figure 5:**
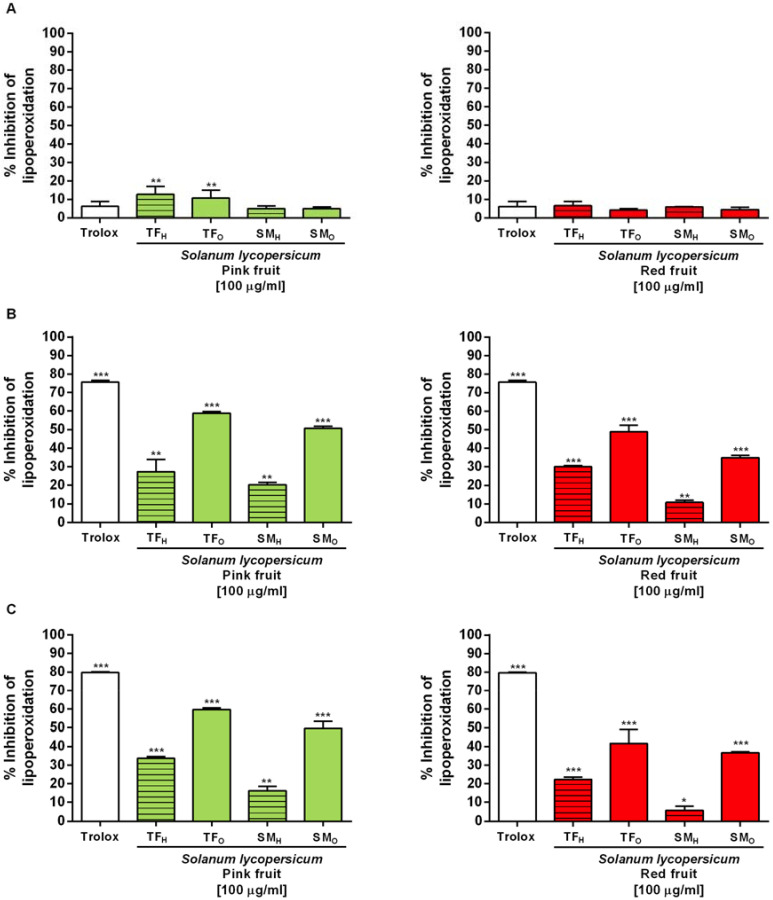
Inhibitory effects of the organic and hydroalcoholic extracts from *Solanum lycopersicum* var. TF and SM tomatoes, at pink (left) and red (right) stages, on linoleic acid peroxidation after different time exposure (**A**) t = 0, (**B**) t = 24 h, and(**C**) t = 48 h. TF organic (TF_O_), TF hydroalcoholic (TF_H_), SM organic (SM_O_), SM hydroalcoholic (SM_H_), extracts 100 µg mL^−1^. * *p* < 0.05, ** *p* < 0.01, and *** *p* < 0.01, represent a statistically significant lipoperoxidation inhibition respect to the basal effect at t = 0 (Anova + Dunnett’s multiple comparison post test).

Regarding the ferric thiocyanate assay, all the extracts showed an inhibitory activity of linoleic acid peroxidation, being the organic samples from both pink and red tomatoes the most effective ones (Figure 5). Among the tested extracts, TF_P_ and TF_R_ organic fractions induced about a 60 and 50% inhibition of lipid peroxidation already after 24 h incubation (Figure 5B), followed by a 50% of SM_P_ organic fraction and a lower than 40% inhibition of the other samples (Figure 5B).

#### 3.3.2. Advanced Glycation End-Product (AGE) Inhibition

Growing evidence highlighted that phenolic compounds are able to prevent the production of advance glycation end products (AGEs), toxic metabolites accumulated under different pathologies, and responsible for the inflammatory and oxidative stress [37]. According to these data, the ability to interfere with AGE formation was assessed as a possible mechanism correlated to the antioxidant and cytoprotective power of TF and SM extracts. Therefore, treatment with AGEs inhibitors is believed to be a potential strategy for preventing diabetes complications.

**Figure 6:**
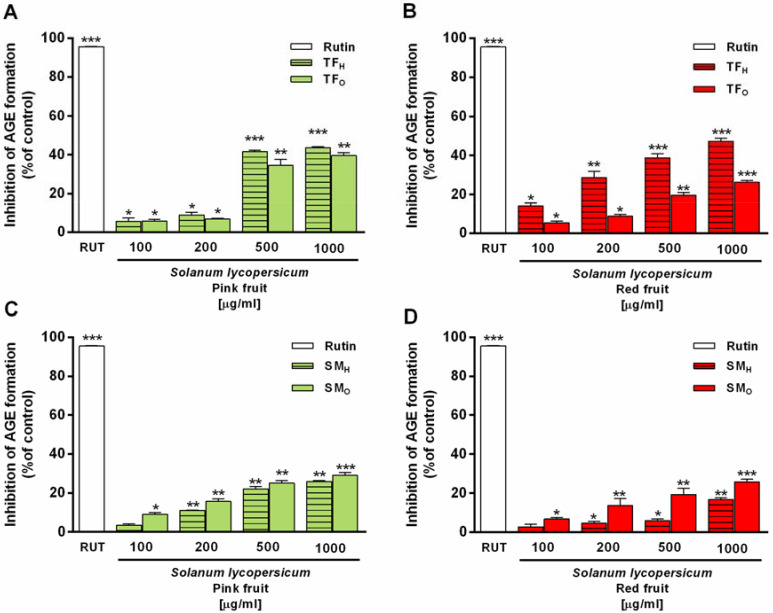
Inhibition of formation of advanced glycation end-products (AGE) induced by organic (O) and hydroalcoholic (H) extracts from *Solanum lycopersicum* var. (**A**) TF_P_, (**B**) TF_R_, (**C**) SM_P_, (**D**) SM_R_, and the positive control rutin [200 μg mL^−1^]. RUT, rutin, TF_H_, TF_O_, SM_H_, SM_O_. Each value represents mean ± SEM (*n* = 6). * *p* < 0.05, ** *p* < 0.01, and *** *p* < 0.01, represent a statistically significant AGE inhibition compared to the basal level (Anova + Dunnett’s multiple comparison post test).

Under the experimental conditions, despite a null activity of SM samples, both TF_P_ and TF_R_ hydroalcoholic fractions produced a concentration-dependent and statistically significant inhibition of the AGE production, although with a potency about 3-fold lower compared to rutin (positive control). The maximum 47% inhibition was achieved at the concentration of 1000 µg mL^−1^ of TF_R_ hydroalcoholic extract (Figure 6B). Furthermore, that from TF_P_ produced a maximum 44% inhibition at the highest tested concentration. According to literature [37], phenolic compounds could contribute to the observed effects.

#### 3.3.3. In Vitro Metabolic Enzyme Inhibition

Taking into account that dietary phenolics are known to decrease the activity of α-amylase and α-glucosidase, thus lowering carbohydrate digestion and absorption [37], the ability of the tested samples was also evaluated to affect the function of both enzymes. Under our experimental conditions, the extracts resulted ineffective towards α-amylase enzyme, whereas a partial α-glucosidase inhibition (maximum 50% inhibition at the highest concentration of 1000 μg mL^−1^) was found in the presence of the hydroalcoholic extracts of both pink and red TF tomatoes (data not shown), likely ascribable to the highest phenolic content.

#### 3.3.4. Cytoprotection towards the Oxidative Stress Induced by tBuOOH

Preliminarily, the cytotoxicity of selected tomatoes samples on HepG2 cells was evaluated by MTT assay, thus highlighting that the extracts did not affect significantly the cell viability up to the concentration of 100 µg mL^−1^ after 24 h exposure, with early toxicity signs at higher concentrations (data not shown). On the basis of this evidence, the concentration of 100 µg mL^−1^ was used to study the ability of the extracts to inhibit the intracellular oxidative stress induced by tBuOOH after 2 h exposure.

**Figure 7:**
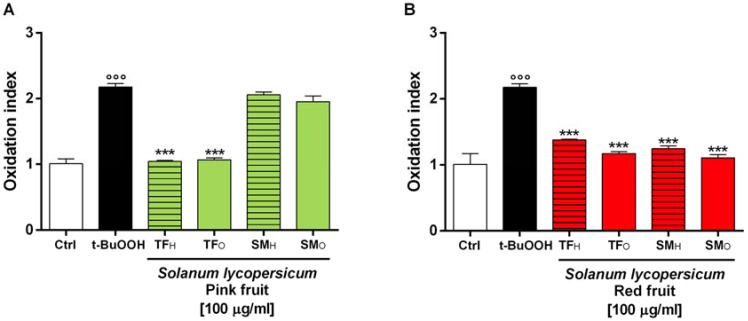
Effect of the organic and hydroalcoholic extracts from the fruits of *Solanum lycopersicum* var. Torpedino di Fondi (TF) and San Marzano (SM) at (**A**) pink and (**B**) red ripening stages on the *t*BuOOH-induced intracellular ROS levels by the DCFH-DA (2,7-dichlorofluorescein diacetate) assay. *t*BuOOH (5 mM) TF_H_, TF_O_, SM_H_, SM_O_. The oxidation index was obtained by the ration between the DCF fluorescence of the sample and that of the vehicle control (i.e., EtOH 1% *v/v*). *** *p* < 0.001, represent a statistically significant difference of the treatment with respect to *t*BuOOH (Anova + Dunnett’s multiple comparison post test). °°° *p* < 0.001, represent a statistically significant difference of *t*BuOOH vs. Ctrl. *p* < 0.001, represent a statistically significant difference of *t*BuOOH vs. Ctrl.

Under our experimental conditions, tBuOOH produced a statistically significant increase of the intracellular ROS-level with respect to the vehicle control, reaching an oxidation index of 2.17 ± 0.04 (Figure 7), while the extracts alone did not affect the ROS levels (data not shown).

When the cells were pre-treated overnight with the tested extracts, the pro-oxidant effect of tBuOOH resulted significantly reduced, although with different potency. Among pink tomato samples, both TF hydroalcoholic and organic extracts were able to halve the tBuOOH-induced oxidation (with 48% and 54% inhibition index respectively), thus exhibiting a strong antioxidant activity (Figure 7A). Conversely, the organic and hydroalcoholic extracts from SM_P_ fruits resulted ineffective, being the oxidation index similar to that of tBuOOH (Figure 7A). All the extracts from red tomatoes displayed marked antioxidant activity, with the oxidation index of tBuOOH reduced from 1.7- to 2-fold. For both varieties, the organic extracts were the most potent samples, achieving the inhibition levels of 46.0 and 49% for TF and SM, respectively (Figure 7B). Analogously, the hydroalcoholic extracts produced antioxidant effects against tBuOOH, although with lower potency, being the inhibition values of 36 and 43% for TF and SM, respectively (Figure 7B).

#### 3.3.5. Antifungal Activity of SM and TF Hydroalcoholic and Organic Extracts

Irving and colleagues have shown antifungal activity of tomato plant extracts against *Candida albicans* ATCC 2091 [72]. In the present study, for the first time has been analyzed the anti-*Candida* activity of different extracts of tomato fruits from TF and SM cultivars against different *Candida* species such as *C. albicans*, *glabrata*, and *krusei*.

*Candida* is a human commensal in several anatomically distinct sites such as in the gastrointestinal tract. In specific environmental condition, *Candida* can switch to pathogen and can be responsible of some diseases. In the gut, patients with intestinal inflammation have high levels of *Candida* species when compared to healthy individuals [73]. The main *Candida* species isolated from the human gastrointestinal tract are *C. albicans*, *C. glabrata*, and *C. krusei* [74]. Antifungal activity of plants extracts was demonstrated on these species by the broth microdilution method. Between the two varieties tested, TF showed a better activity, against all *Candida* strains (Table S5). Moreover, organic fractions showed the best antifungal activity compared to the hydroalcoholic ones. In particular, organic TF_R_ and TF_P_ and hydroalcoholic TF_R_ and TF_P_ extracts showed a geometric (GM) MIC_50_ of 707 µg mL^−1^, 841 µg mL^−1^, 1361 µg mL^−1^, 1101 µg mL^−1^, respectively. Organic SM_R_ and SM_P_, hydroalcoholic SM_R_ and SM_P_ fractions showed a GM MIC_50_ of 1236 µg mL^−1^, 891 µg mL^−1^, 1442 µg mL^−1^, 1414 µg mL^−1^, respectively (Table S5) In particular, TF_R_ organic extracts showed a GM MIC_50_ of 707 µg mL^−1^, while the TF_R_ hydroalcoholic showed a GM MIC_50_ 1442 µg mL^−1^ against all *Candida* strains (Table S5).

Antibacterial and antifungal activity of several plant secondary metabolites and their derivatives such as alkaloids and polyphenols has been reported [75]. The alkaloid such as glycoalkaloid α-tomatine depicted antifungal effects against a variety of fungi [76]. In our results, tomatine was found only in TF_P_ and not in TF_R_, indicating that probably, as hypothesized by some authors, the synergy of several compounds is responsible for the antifungal activity shown.

In conclusion, TF reduces *Candida* cells in the intestinal tract intake of TF, which have a growth inhibiting activity against different *Candida* species, and could be a strategy to restore the intestinal microbiota present in the healthy individual.

## 4. Conclusions

The combined application of both targeted and untargeted methodologies allowed to outline the chemical profile of both TF, a new hybrid cultivar recently introduced in south Lazio (Italy), and SM tomatoes at two ripening stages. We wish to itemize here in some detail both single important molecules and chemical classes to stimulate an active consideration of these highly complex natural mixtures, rich in compounds that may reveal novel important, hopefully beneficial roles in forthcoming studies. Some metabolites were shared by all extracts, though at different concentration, such as macronutrients like sugars and derivatives (hexose, sorbitol, mannosylglycerate), and amino acids (tryptophan and citrulline), the Amadori adduct fructoselysine, relevant biochemical intermediates (ornithine, chorismic acid, and GABA), terpenes (caryophyllene), nucleobase (adenosine), vitamin precursors and metabolites (diapophytoene, diapolycopene, α-tocopheronolactone), fatty alcohol (panaxytriol), organic acids (citric, chlorogenic, and azelaic acid) and conjugates (caffeic acid 3-glucoside, O-feruloylquinate), sterols (solagenin), free fatty acids (myristic, myristoleic, lauric, palmitic, oleic, linoleic, linolenic, eicosenoic acids). Differently, other metabolites might be considered marker compounds, being detected only in one or a few extracts, like glycyphyllin, maleic and tartaric acids (TF_P_), cinnamoyl glucoside (TF_R_), the vitamins dihydroretinol, dehydroretinal, retinoic and tocopheronic acids, and the antifungal terpenoid phytuberin (SM_R_), the solanine derivative, tomatidinol (SM_P_), the vitamin-E precursors, phytol (SM_R_, TF_P_), and γ-tocotrienol (SM_P_, SM_R_), the glycoalkaloids tomatine and tomatidine, suberic and ascorbic acids (SM_P_, TF_P_), quinic, phosphogluconic and shikimic acids (TF_R_, TF_P_), the polyketide lycoflexine (SM_R_, SM_P_, TF_P_). These characteristic chemical features may concur to the excellent organoleptic properties as well as to antioxidant, antiglycative, and antifungal activities of Torpedino di Fondi, an emerging south Lazio tomato belonging to the mini-San Marzano type. This study may contribute to the unceasing buildup of reliable reference databases useful to guarantee food authenticity and freshness, and to support consumers and further nutraceutical evaluations.

## Figures and Tables

**Table 1:** Drug to extract ratio of Bligh–Dyer (hydroalcoholic and organic) extracts of both pink and red fruits from *Solanum lycopersicum* var. Torpedino di Fondi (TF) and San Marzano (SM) ^a^.

Sample	Drug/Extract Ratio (DER)	
	Hydroalcoholic	Organic
TF_P_	20	274
TF_R_	9	320
SM_P_	20	447

^a^ TF_P_ = pink TF; TF_R_ = red TF; SM_P_ = pink SM; SM_R_ = red SM.

**Table 2:** Compounds and relative signals (δ(^1^H), ppm) selected for quantitative analysis in the hydroalcoholic and organic extracts.

Ppm	Compounds	Ppm	Compounds
**Hydroalcoholic**			
0.96	Leucine	3.25	β-Glucose
0.99	Valine	4.04	Fructose
1.01	Isoleucine	4.31	Malic Acid
1.34	Threonine	4.59	β-Galactose
1.49	Alanine	5.25	α-Glucose
2.30	γ-amino butyric acid (GABA)	6.91	Tyrosine
2.35	Glutamic acid	7.34	Phenylalanine
2.46	Glutamine	7.74	Tryptophan
2.55	Citric Acid	8.14–8.17	Histidine
2.81	Aspartic acid	8.36	Adenosine
2.90	Asparagine	8.46	Formic Acid
3.04	Lysine	8.586	ADP
3.21	Choline	9.13	Trigonelline
**Organic**			
0.66	β-Sitosterol	2.73	Di-unsaturated fatty acids (DUFAs)
0.68	Stigmasterol	2.77	Tri-unsaturated fatty acids (TUFAs)
2.30	Total fatty acids (FAs)	5.31	Total unsaturated fatty acids (UFAs)

**Table 3:** Color, weight, and size (length and diameter) of both pink and red fruits from Solanum lycopersicum TF and SM varieties. Data are displayed as mean ± SE (n = 10) ^a^.

Sample	Color	Weight (g)	Size (cm)	
			Length	Diameter
**TF_P_**	dark-green	21.7 ± 0.1	6.3 ± 0.1	2.6 ± 0.1
**TF_R_**	bright red	20.6 ± 0.1	5.6 ± 0.1	2.7 ± 0.1
**SM_P_**	pale green	106.1 ± 0.9 **	10.1 ± 0.1 **	4.5 ± 0.1 *
**SM_R_**	light red	110.7 ± 0.6 **^,§^	11.4 ± 0.1 **	4.4 ± 0.1 *

^a^ * *p* < 0.05 and ** *p* < 0.01 denote a statistically significant difference compared to TF tomato at the same ripening stage (t-Student Test). ^§^ *p* < 0.05 denotes a statistically significant difference with respect to pink stage within the same cultivar (t-Student Test).

**Table 4:** Amounts of chlorophyll a, chlorophyll b, and total carotenoids in the organic extracts of both pink and red fruits from Solanum lycopersicum var. Torpedino di Fondi (TF) compared San Marzano (SM) tomatoes ^a^.

Sample	Fruit Part	Chlorophyll a	Chlorophyll b	Total Carotenoids	Ratio (a + b)/Total Carotenoids
	μg/g FW				
**TF_P_**	peel	166 ± 32	67 ± 3	79 ± 7	3.16
	pulp	178 ± 30	94 ± 4	82 ± 9	
**TF_R_**	peel	49 ± 5 ^§^	32 ± 3 ^§^	404 ± 22 ^§§^	0.18
	pulp	18 ± 4 ^§§^	17 ± 1 ^§§^	455 ± 10 ^§§^	
**SM_P_**	peel	63 ± 3 **	33 ± 4 *	26 ± 5 **	3.9
	pulp	31 ± 3 ***	13 ± 3 **	13 ± 3 ***	
**SM_R_**	peel	5 ± 2 *** ^§^	9 ± 1 ^§^	723 ± 4 ***^,§§^	0.02
	pulp	4 ± 2 ^§^	8 ± 1	511 ± 13 ***^,§§^	

^a^ * *p* < 0.05, ** *p* < 0.01 and *** *p* < 0.001 denote a statistically significant difference compared to TF tomato at the same ripening stage (ANOVA followed by Bonferroni multiple comparison post test). ^§§^ *p* < 0.01 denotes a statistically significant difference with respect to pink stage within the same variety (ANOVA followed by Bonferroni multiple comparison post test).

**Table 5:** Number of chemical formulas detected in hydroalcoholic and organic extracts of pink and red SM and TF by ESI FT-ICR MS.

Sample		Ion Mode	Detected Molecular Formulas	
**San Marzano**				
Hydroalcoholic	Pink	ESI(+)	824	935
		ESI(−)	132	
	Red	ESI(+)	808	1031
		ESI(−)	240	
Organic	Pink	ESI(+)	401	508
		ESI(−)	113	
	Red	ESI(+)	865	1138
		ESI(−)	286	
**Torpedino di Fondi**				
Hydroalcoholic	Pink	ESI(+)	488	652
		ESI(−)	261	
	Red	ESI(+)	549	751
		ESI(−)	204	
Organic	Pink	ESI(+)	381	586
		ESI(−)	208	
	Red	ESI(+)	800	948
		ESI(−)	151	

**Table 6:** Amounts of total polyphenols, tannins and flavonoids in Bligh–Dyer hydroalcoholic and organic extracts of both pink and red fruits from *Solanum lycopersicum* var. TF and SM ^a^.

Sample	ADD HEADING	Polyphenols	Tannins	Flavonoids
		[µg TAEs/g Fruit] ^b^		[µg QEs/g Fruit] ^c^
TF_P_	Hydroalcoholic	155.0 ± 0.1	5.0 ± 0.4	149.7 ± 0.6
	Organic	36.9 ± 0.3	7.7 ± 0.3	54.4 ± 0.4
TF_R_	Hydroalcoholic	11.1 ± 0.3 *^§§§^*	6.4 ± 0.1 *^§^*	101.3 ± 0.8 *^§§^*
	Organic	22.9 ± 0.5 *^§§^*	0.9 ± 0.1 *^§§§^*	12.8 ± 0.5 *^§§§^*
SM_P_	Hydroalcoholic	55.0 ± 0.4 ***	5.0 ± 0.6	50.0 ± 0.7 ***
	Organic	13.7 ± 0.1 ***	4.7 ± 0.2 **	2.2 ± 0.1 ***
SM_R_	Hydroalcoholic	9.1 ± 0.6 *^§§§^*	5.8 ± 0.2	91.0 ± 0.3 * *^§§^*
	Organic	27.8 ± 0.7 *^§§§^*	4.3 ± 0.3 ***	132.2 ± 1.2 *** *^§§§^*

^a^ * *p* < 0.05, ** *p* < 0.01 and *** *p* < 0.001 denote a statistically significant difference compared to TF tomato at the same stage of ripening (ANOVA followed by Bonferroni multiple comparison post test). ^§^ *p* < 0.05, ^§§^ *p* < 0.01 and ^§§§^ *p* < 0.001 denotes a statistically significant difference with respect to pink stage within the same variety (t-Student Test). ^b^ TAEs, tannic acid equivalents. ^c^ QEs, quercetin equivalents.

**Table 7:** Biogenic amines determined by HPLC in SM and TF samples, at red and pink ripening stages ± Std. Dev. (μg/g) ^a^.

ADD HEADING	BPEA	PUT	CAD	HIS	SER	TYM	SPD	SPM
SM_P_	0.170 ± 0.001	2.777 ± 0.137	1.127 ± 0.055	0.448 ± 0.027	277.760 ± 5.226	n.d.	0.235 ± 0.010	0.253 ± 0.013
SM_R_	0.168 ± 0.001	7.564 ± 0.289	1.888 ± 0.025	1.363 ± 0.037	394.054 ± 12.725	n.d.	0.122 ± 0.006	0.346 ± 0.019
TF_P_	0.165 ± 0.001	3.289 ± 0.006	1.287 ± 0.006	0.589 ± 0.032	258.679 ± 7.360	n.d.	0.116 ± 0.001	0.191 ± 0.002
TF_R_	0.272 ± 0.050	6.293 ± 0.113	1.533 ± 0.033	1.463 ± 0.015	326.848 ± 8.850	n.d.	0.249 ± 0.010	0.477 ± 0.031

^a^ BPEA: β-phenylethylamine, PUT: putrescine; CAD: cadaverine; HIS: histidine; SER: serotonin; TYM: tyramine; SPD: spermidine; SPM: spermine; n.d.: not detected.

**Table 8:** Effects of hydroalcoholic and organic extracts from both pink (*p*) and red (R) fruits of *Solanum lycopersicum* var. TF and SM, and standard antioxidant agents in the antioxidant assays ^a^.

Sample	IC_50_ (CL) (μg mL^−1^) ^a^			
	ADD HEADING	ABTS Radical Scavenging Activity	Ferric Ion Chelating Activity	Ferric Ion Reducing Activity
TF_P_	Hydroalcoholic	371.2 (296.6–494.8)	106.6 (69.3–163.6)	-
	Organic	174.9 (119.6–255.8)	-	184.6 (109.3–433.6)
TF_R_	Hydroalcoholic	-	208.5 (143.1–303.1)	*-*
	Organic	573.1 (463.3–708.9)	-	250.0 (152.1–410.5)
SM_P_	Hydroalcoholic	629.9 (475.9–720.5)	93.5 (63.9–116.6)	-
	Organic	479.6 (358.7–663.3)	-	397.8 (319.3–438.7)
SM_R_	Hydroalcoholic	-	242.7 (233.9–276.6)	-
	Organic	-	-	368.5 (289.3–468.6)
Positive control		2.5 (1.3–5.6) ^b^	45.2 (13.1–75.5) ^c^	1.5 (1.1–2.0) ^b^

^a^ CL, confidence limits; - not evaluable since a lower than 40% effect was achieved. ^b^ trolox; ^c^ quercetin.

## Supplementary Materials

Supplementary materials can be found at https://www.mdpi.com/2076-3921/9/10/1027/s1, Figures S1–S5: TF and SM tomato fruits from Fondi area (Lazio region), Figure S6: Comparison between TFP and TFR and SMP and SMR their ripening stage for the number of possible CHO (A) and CHNO (B) metabolites, Figure S7: Common and uncommon features (in%) in the combined pattern of hydroalcoholic and organic extracts, Table S1: Amount of peel, pulp, seeds and juice in both pink and red Solanum lycopersicum var. TF and SM fruits; Table S2–3: Comprehensive list of metabolites detected in hydroalcoholic and organic fractions of pink and red San Marzano (SM) and Torpedino di Fondi (TF) extracts, Table S4: Overview of the relative abundancies of specific classes of saturated, mono-, di- and poly-unsaturated fatty acids (FA), Table S5: Antifungal activity of extracts against 4 *C.albicans* strains, 3 *C. glabrata* strains and 2 *C. krusei*.
