# Supplementary material for: Chemico-Biological Characterization of Torpedino Di Fondi® Tomato Fruits: A Comparison with San Marzano Cultivar at Two Ripeness Stages
Source: Antioxidants (Basel). 2020 Oct 21;9(10):1027. doi: 10.3390/antiox9101027 (PMC7590105; doi:10.3390/antiox9101027)
Supplement: Supplementary file 1 [file antioxidants-09-01027-s001.pdf]

Supplementary material

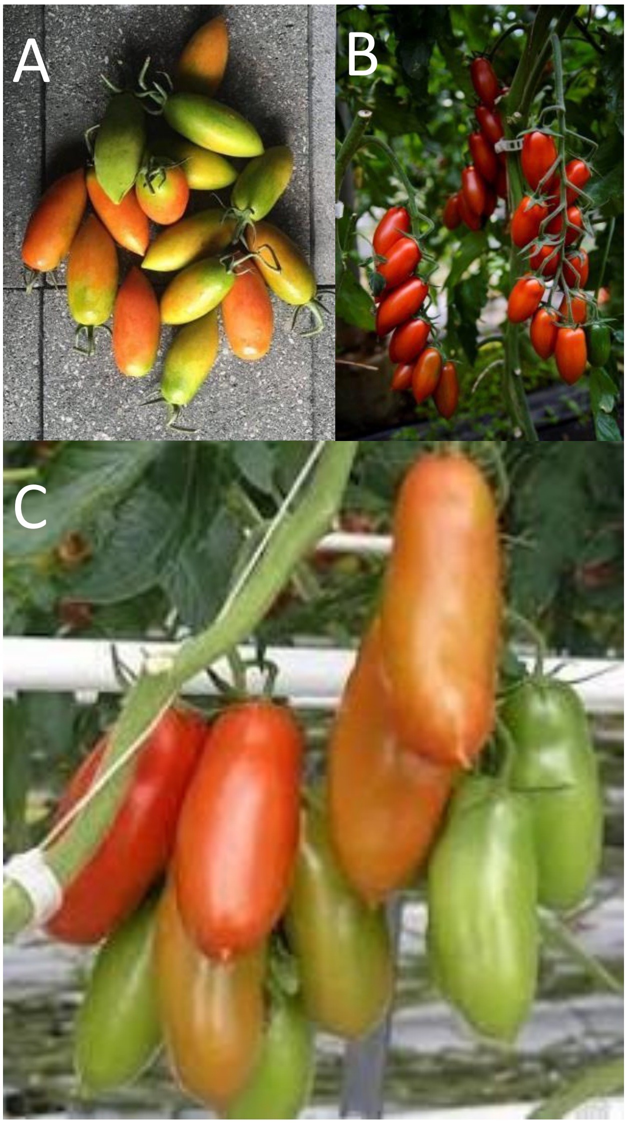

Figure 1. TF and SM tomato fruits from Fondi area (Lazio region): **A**) TF at pink ripening stage; **B**) TF at red ripening stage; **C**) SM at both pink and red ripening stages.

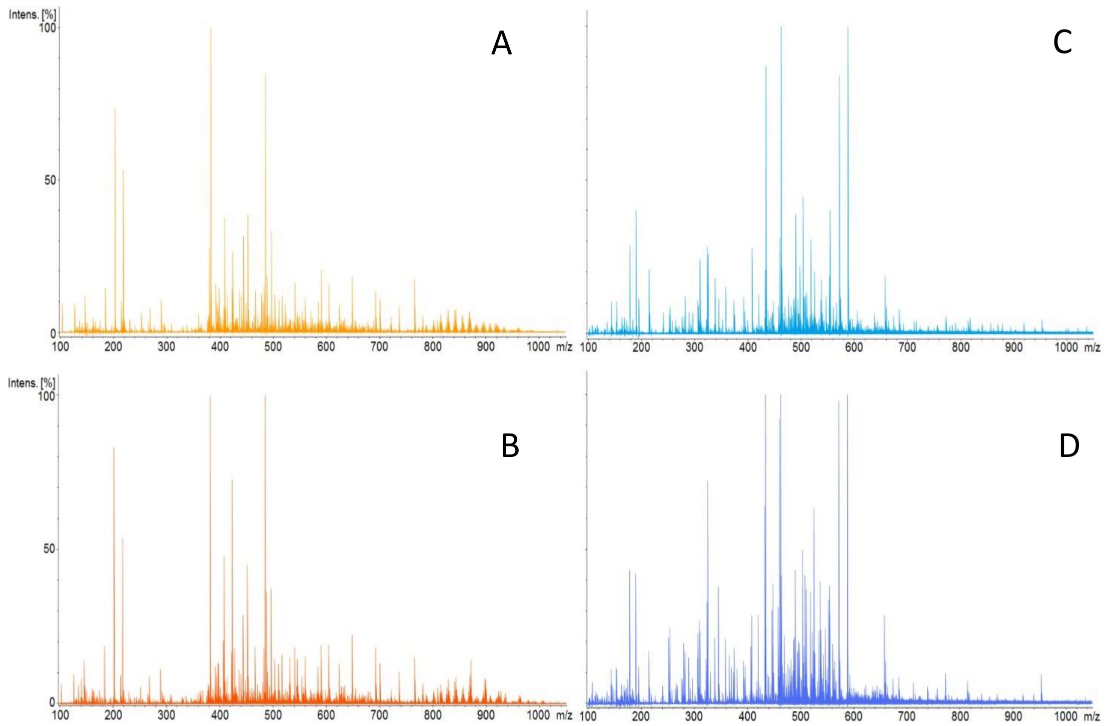

Figure 2. Raw ESI(+) FT-ICR MS spectra of hydroalcoholic extracts of **A**) SM$_P$ (yellow profile) and **B**) SM$_R$ (orange profile); raw ESI(-) FT-ICR MS spectra of hydroalcoholic extracts of **C**) SM$_P$ (light blue profile) and **D**) SM$_R$ (blue profile).

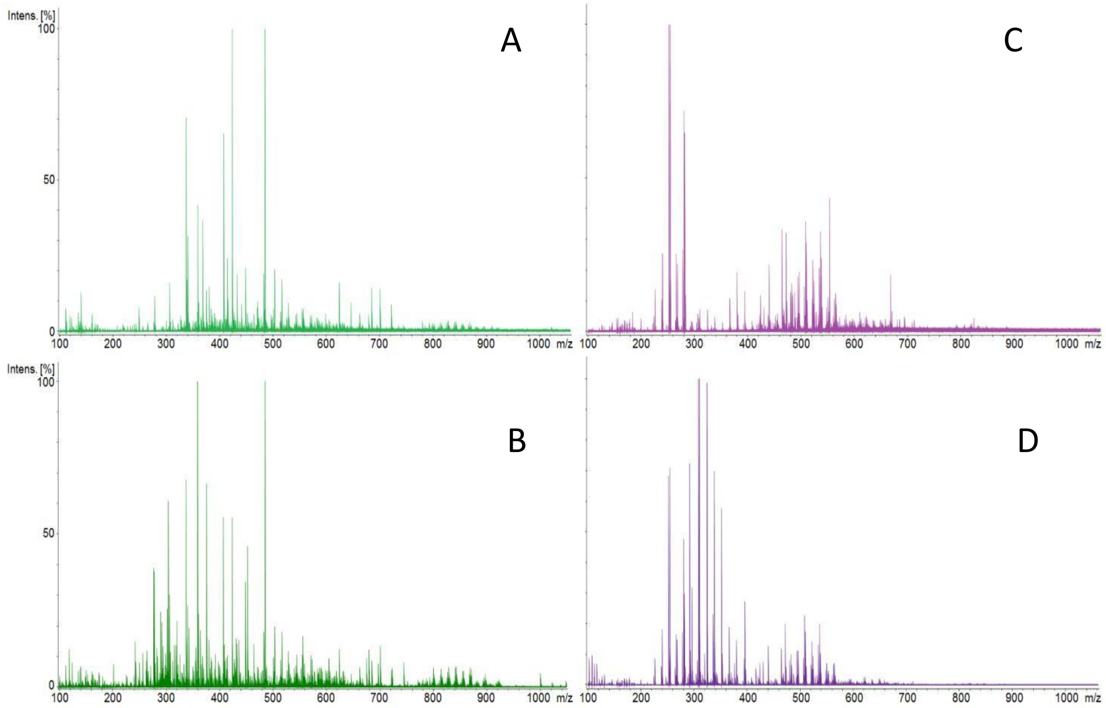

Figure 3. Raw ESI(+) FT-ICR MS spectra of organic extracts of **A**) SM$_P$ (green profile), **B**) SM$_R$ (dark green profile); raw ESI(-) FT-ICR MS spectra of organic extracts of **C**) SM$_P$ (pink profile) and **D)** SM$_R$ (violet profile).

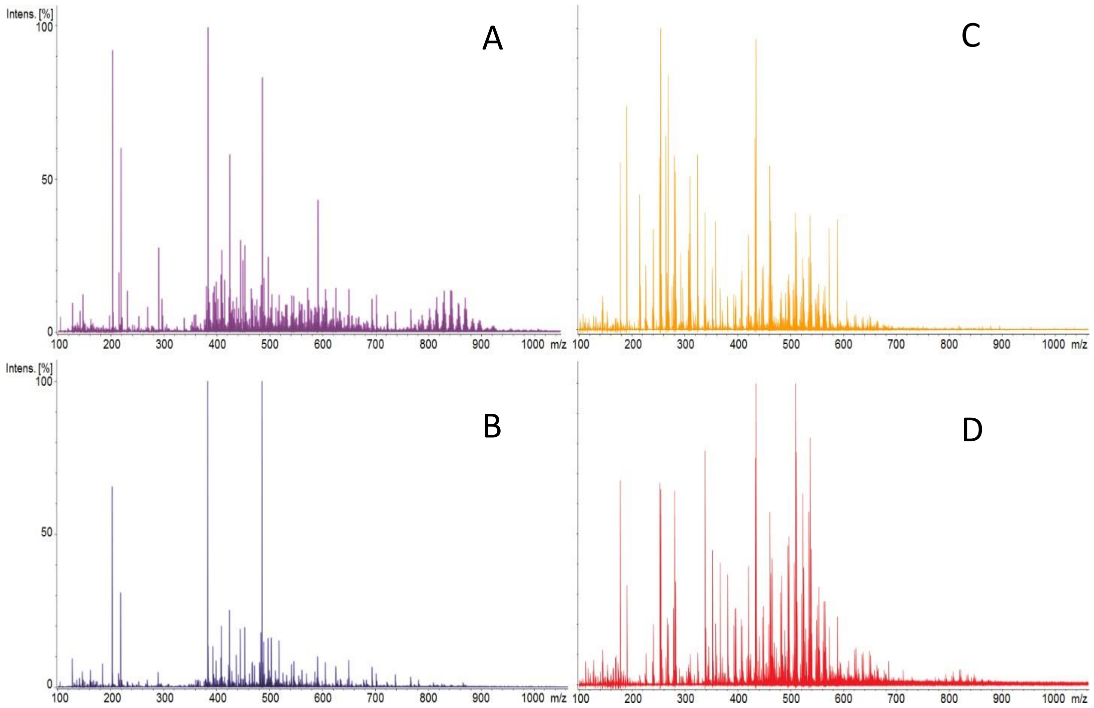

Figure 4. Raw ESI(+) FT-ICR MS spectra of hydroalcoholic extracts **A**) TF_P (violet profile), **B**) TF_R (blue profile); raw ESI(-) FT-ICR MS spectra of hydroalcoholic extracts **C**) TF_P (orange profile) and **D**) TF_R (red profile).

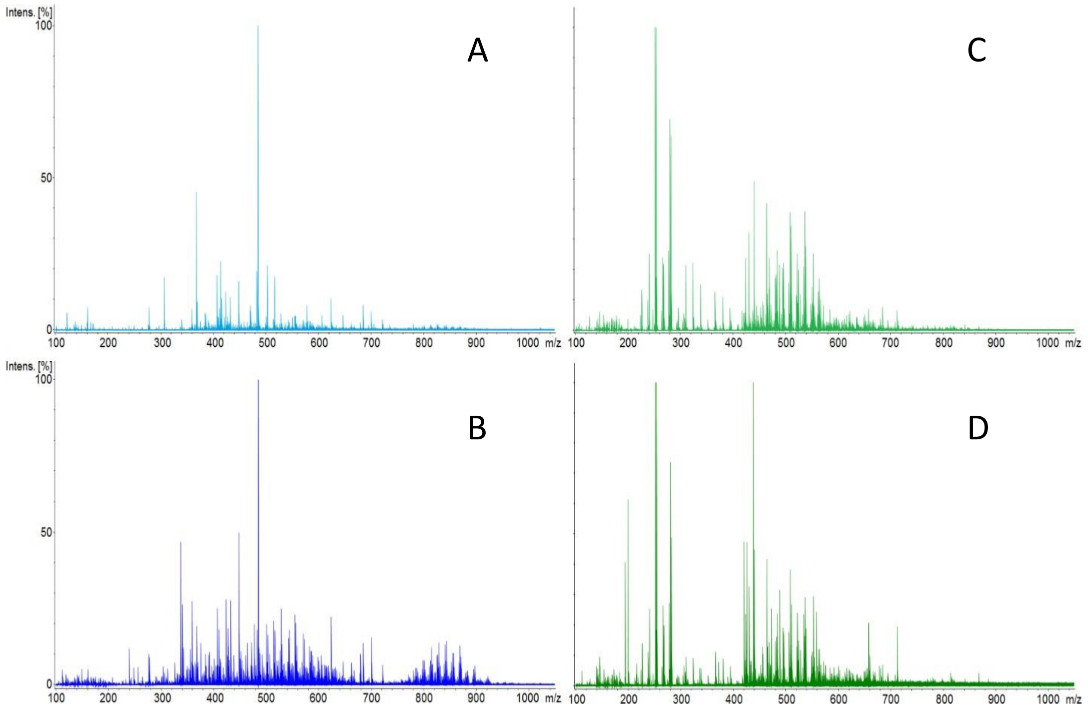

Figure 5. Raw ESI(+) FT-ICR MS spectra of organic extracts **A**) TF_P (light blue profile) and **B**) TF_R (blue profile); raw ESI(-) FT-ICR MS spectra of **C**) TF_P (light green profile) and **D**) TF_R (dark green profile).

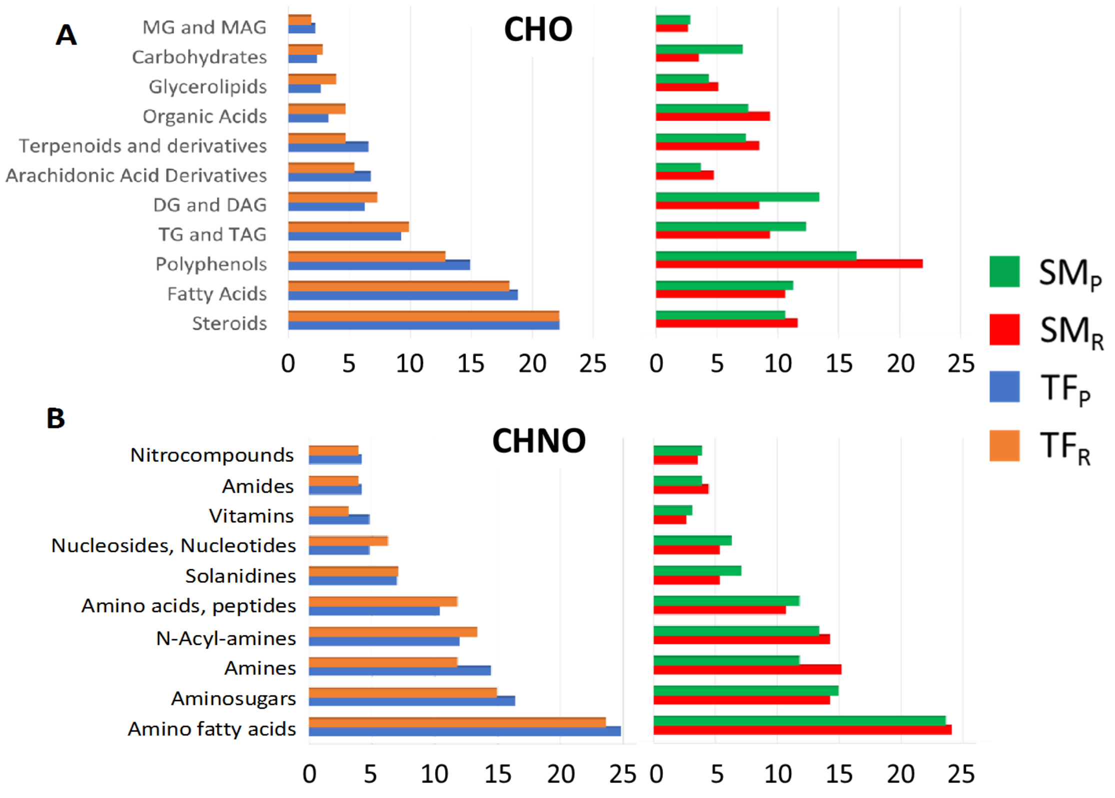

Figure 6. Comparison between TF_P and TF_R and SM_P and SM_R of number of possible **A**) CHO and **B**) CHNO metabolites assigned in the various metabolic pathways of *Solanum lycopersicum* var. Torpedino di Fondi and San Marzano.

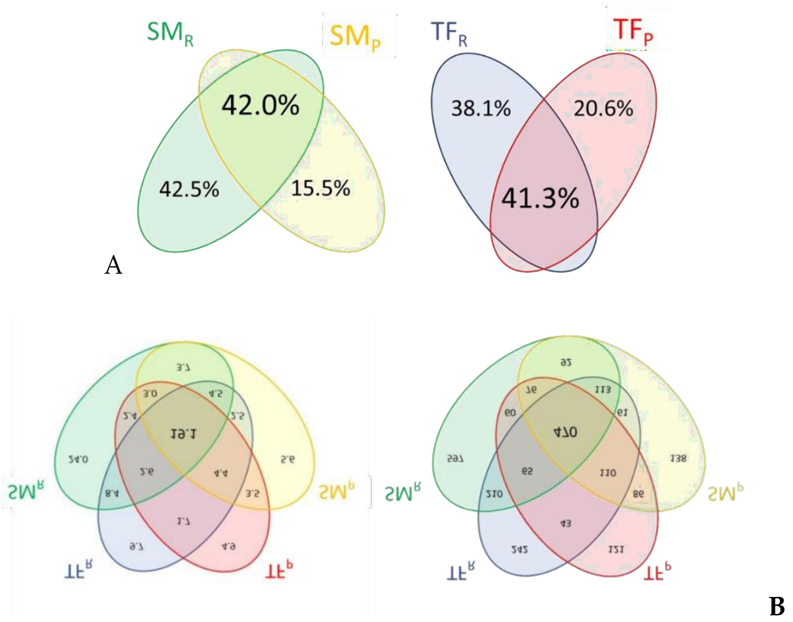

Figure 7. Common and uncommon features (in %) in the combined pattern of hydroalcoholic and organic extracts of **A**) SM_R and SM_P and TF_R and TF_P , shown in a two-way Venn diagram, and **B**) SM_R and SM_P and TF_R nd TF_P , shown in a four-way Venn diagram. The results are reported as relative percentages (**A** and left **B**) or absolute numbers (right **B**) of total hits.

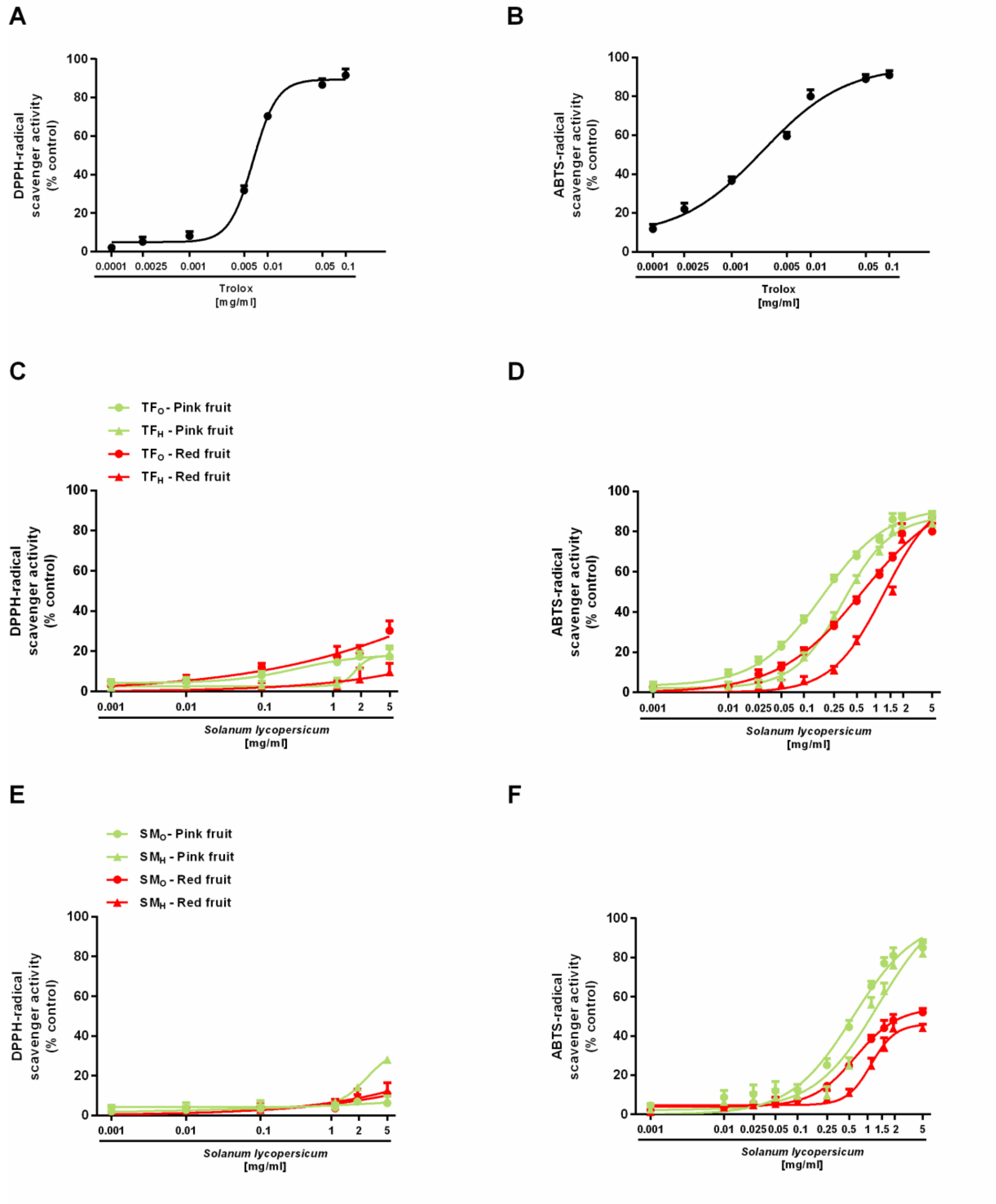

Figure 8. Concentration-response curves of the scavenging activity of TF and SM extracts and the positive control trolox towards DPPH and ABTS radicals. **A-B**) Scavenging activity of trolox. **C-D)** Scavenging activity of TF_P and TF_R hydroalcoholic (H) and organic (O) extracts. **E-F)** Scavenging activity of SM_P and SM_R hydroalcoholic (H) and organic (O) extracts. Each value represents the mean ⊚ SE of at least two experiments and at least three replicates for each experiment (n = 6).

Table 1. Amount of peel, pulp, seeds and juice in both pink and red *Solanum lycopersicum* var. TF and SM fruits.[a]

Sample	Peel	Pulp	Seeds		Juice
	mg/g fruit<br>*% fruit w/w*	mg/g fruit<br>*% fruit w/w*	mg/g fruit<br>(mg/seed)<br>*% fruit w/w*	Number/<br>g fruit	g/g fruit<br>(pH)<br>*% fruit w/w*
**TF$_P$**	15.1 ± 0.2<br>*1.5*	630.0 ± 10.1<br>*62.6*	17.0 ± 0.6<br>(5.6 ± 0.6)<br>*1.7*	3.1 ± 0.2	0.34 ± 0.02<br>(4.1 ± 0.1)<br>*34.3*
**TF$_R$**	37.9 ± 0.2§§<br>*3.7*	820.0 ± 20.3§§<br>*81.7*	15.7 ± 0.8§<br>(4.4 ± 0.1) §<br>*1.6*	3.6 ± 0.1§	0.13 ± 0.01§§<br>(4.0 ± 0.1)<br>*13.0*
**SM$_P$**	8.2 ± 0.6**<br>*0.4*	161.0 ± 11.0**<br>*16.1*	3.9 ± 0.01**<br>(7.5 ± 0.5)<br>*0.5*	0.6 ± 0.02**	0.83 ± 0.02**<br>(4.2 ± 0.1)<br>*83.0*
**SM$_R$**	4.0 ± 0.4§§**<br>*0.7*	178.0 ± 10.4§§**<br>*15.7*	4.4 ± 0.02**<br>(5.9 ± 0.01) §<br>*0.4*	0.7 ± 0.02§**	0.83 ± 0.03**<br>(4.0 ± 0.1)<br>*83.1*

[a)] **$p < 0.01$ denotes a statistically significant difference respect to TF tomato at the same stage of ripening (ANOVA followed by Bonferroni Multiple Comparison Post Test). § $p < 0.05$ and §§ $p < 0.01$ denote a statistically significant difference respect to unripe stage within the same variety (ANOVA followed by Bonferroni Multiple Comparison Post Test).

Table S2: Comprehensive list of metabolites detected in hydroalcoholic and organic fractions of pink and red San Marzano (SM) and Torpedino di Fondi (TF) extracts using ESI(+) FT-ICR MS.

							ESI(+) FT-ICR MS							
**No.**	**Plausible Compound[a]**	**Molecular formula (M)**	**Ion**	**Theor m/z[b]**	**Exp. m/z[c]**	**Δppm[d]**	**SM_RH^e**	**SM_RO^e**	**SM_PH^e**	**SM_PO^e**	**TF_RH^e**	**TF_RO^e**	**TF_PH^e**	**TF_PO^e**
1	Gamma-Aminobutyric acid	$C_4H_9NO_2$	[M+H]+	104.07060	104.07065	-0.41	7.0E+07		1.1E+08		3.6E+07		3.8E+07	
2	L-Serine	$C_3H_7NO_3$	[M+H]+	106.04987	106.04992	-0.46	1.7E+06	7.9E+06	2.6E+06	1.7E+06		6.2E+06	2.4E+06	1.2E+06
3	Aminobutyraldehyde	C_4H_9NO	[M+Na]+	110.05763	110.05768	-0.41		1.8E+06		8.8E+05		1.5E+06		1.3E+06
4	Lactic acid	$C_3H_6O_3$	[M+Na]+	113.02091	113.02097	-0.47		1.6E+07	9.6E+05	3.8E+06		8.9E+06	2.1E+06	2.6E+06
5	Butanediol	$C_4H_{10}O_2$	[M+Na]+	113.05730	113.05735	-0.44		4.1E+06		1.2E+06		1.6E+06		1.7E+06
6	Glycerol	$C_3H_8O_3$	[M+Na]+	115.03656	115.03662	-0.44		2.9E+06		1.1E+06		2.2E+06		2.7E+06
7	Amino-piperidone	$C_5H_{10}N_2O$	[M+H]+	115.08659	115.08664	-0.44		2.6E+06					1.4E+06	
8	Proline	$C_5H_9NO_2$	[M+H]+	116.07060	116.07065	-0.40	1.0E+06	3.3E+06	1.4E+06			1.5E+06	1.7E+06	
9	L-Valine	$C_5H_{11}NO_2$	[M+H]+	118.08626	118.08631	-0.45	1.4E+06	4.8E+06	2.0E+06			1.9E+06	1.2E+06	
10	L-Threonine	$C_4H_9NO_3$	[M+H]+	120.06552	120.06558	-0.48	1.9E+06	3.4E+06	2.3E+06		1.4E+06	1.8E+06	1.2E+06	
11	Tromethamine	$C_4H_{11}NO_3$	[M+H]+	122.08117	122.08123	-0.49							1.1E+06	
12	Diethylene glycol	$C_4H_{10}O_3$	[M+Na]+	129.05221	129.05225	-0.27								1.0E+06
13	Pyroglutamic acid	$C_5H_7NO_3$	[M+H]+	130.04987	130.04993	-0.45	3.1E+06	4.0E+06	2.4E+06		2.0E+06	1.6E+06	1.6E+06	
14	Octylamine	$C_8H_{19}N$	[M+H]+	130.15903	130.15909	-0.49								1.9E+06
15	Leucine	$C_6H_{13}NO_2$	[M+H]+	132.10191	132.10195	-0.37	2.7E+06	1.6E+07	4.3E+06		1.3E+06	3.4E+06	2.1E+06	1.1E+06
16	Hydroxy-hexanoic acid	$C_6H_{12}O_3$	[M+H]+	133.08592	133.08593	-0.07	1.0E+06							
17	Ornithine	$C_5H_{12}N_2O_2$	[M+H]+	133.09715	133.09721	-0.38	7.4E+06	1.1E+07	1.0E+07	5.6E+06	2.9E+06	8.1E+06	1.2E+07	2.1E+06
18	Aspartic acid	$C_4H_7NO_4$	[M+H]+	134.04478	134.04484	-0.42	5.6E+06	3.6E+06	3.2E+06	9.2E+05	3.0E+06	3.0E+06	3.5E+06	
19	p-Cymene	$C_{10}H_{14}$	[M+H]+	135.11683	135.11688	-0.39						1.1E+06		
20	Limonene	$C_{10}H_{16}$	[M+H]+	137.13248	137.13254	-0.42		1.4E+06				1.5E+06		
21	Trigonelline	$C_7H_7NO_2$	[M+H]+	138.05495	138.05500	-0.34	3.0E+06		3.1E+06		2.0E+06		2.6E+06	
22	Nicotinamide N-oxide	$C_6H_6N_2O_2$	[M+H]+	139.05020	139.05026	-0.38	3.3E+06	5.6E+07	6.2E+06	3.7E+06	1.1E+06	8.6E+06	7.5E+06	1.3E+06
23	Succinic acid	$C_4H_6O_4$	[M+Na]+	141.01583	141.01588	-0.36				4.6E+05		1.0E+06		
24	L-Histidinol	$C_6H_{11}N_3O$	[M+H]+	142.09749	142.09743	0.41	1.1E+06							
25	Hydroxyisobutyric acid	$C_4H_8O_3$	[M+H]+	143.01050	143.01052	-0.08			1.3E+06				1.4E+06	
26	Ectoine	$C_6H_{10}N_2O_2$	[M+H]+	143.08150	143.08156	-0.39		1.4E+06						

						ESI(+) FT-ICR MS								
**No.**	**Plausible Compound[a]**	**Molecular formula (M)**	**Ion**	**Theor m/z[b]**	**Exp. m/z[c]**	**Δppm[d]**	**SM$_R$H[e]**	**SM$_R$O[e]**	**SM$_P$H[e]**	**SM$_P$O[e]**	**TF$_R$H[e]**	**TF$_R$O[e]**	**TF$_P$H[e]**	**TF$_P$O[e]**
27	Hexenyl acetate	$C_8H_{14}O_2$	[M+H]+	143.10666	143.10671	-0.38		1.4E+06						
28	Solerol	$C_6H_{10}O_4$	[M+H]+	147.06519	147.06511	0.55	1.1E+06					1.1E+06		
29	Glutamine	$C_5H_{10}N_2O_3$	[M+H]+	147.07642	147.07648	-0.42	2.0E+08	7.1E+06	1.5E+08		1.1E+08	1.5E+06	7.8E+07	
30	Lysine	$C_6H_{14}N_2O_2$	[M+H]+	147.11280	147.11286	-0.39	7.2E+06	1.1E+07	6.8E+06	2.1E+06	5.5E+06	4.5E+06	5.1E+06	1.5E+06
31	Glutamic acid	$C_5H_9NO_4$	[M+H]+	148.06043	148.06049	-0.35	8.4E+07	1.0E+07	2.5E+07	8.3E+05	4.8E+07	3.2E+06	2.3E+07	7.8E+05
32	Thymine	$C_5H_6N_2O_2$	[M+Na]+	149.03215	149.03220	-0.31	1.2E+06				1.7E+06			
33	Cinnamic acid	$C_9H_8O_2$	[M+H]+	149.05971	149.05975	-0.30				4.5E+05				2.0E+06
34	Mevalonic acid	$C_6H_{12}O_4$	[M+H]+	149.08084	149.08072	0.75	2.1E+06		1.6E+06		1.5E+06			
35	Ectocarpen	$C_{11}H_{16}$	[M+H]+	149.13248	149.13254	-0.42		3.0E+06				1.7E+06		
36	Creatinine	$C_4H_7N_3O$	[M+K]+	152.02207	152.02213	-0.39				1.3E+06				
37	Oxoproline	$C_5H_7NO_3$	[M+Na]+	152.03181	152.03188	-0.40				2.5E+06				1.7E+06
38	Ketoleucine	$C_6H_{10}O_3$	[M+Na]+	153.05221	153.05228	-0.43		2.1E+06						
39	Histidine	$C_6H_9N_3O_2$	[M+H]+	156.07675	156.07681	-0.36	1.3E+07	2.2E+07	1.2E+07	2.6E+06	7.9E+06	4.8E+06	1.1E+07	1.2E+06
40	Dimethyl-heptenoic acid	$C_9H_{16}O_2$	[M+H]+	157.12231	157.12238	-0.47		1.2E+06						
41	Methylnicotinamide	$C_7H_8N_2O$	[M+Na]+	159.05288	159.05273	0.97								8.2E+05
42	Trigonelline	$C_7H_7NO_2$	[M+Na]+	160.03690	160.03694	-0.26			1.5E+06					
43	Valerylglycine	$C_7H_{13}NO_3$	[M+H]+	160.09682	160.09686	-0.25	1.1E+06							
44	Pargyline	$C_{11}H_{13}N$	[M+H]+	160.11208	160.11206	0.10			1.5E+06					
45	Alanyl-alanine	$C_6H_{12}N_2O_3$	[M+H]+	161.09207	161.09215	-0.50			1.0E+06					
46	Tryptamine	$C_{10}H_{12}N_2$	[M+H]+	161.10732	161.10739	-0.40							1.1E+06	
47	Dimethylmalic acid	$C_6H_{10}O_5$	[M+H]+	163.06010	163.06015	-0.29	1.9E+06		2.1E+06		1.6E+06		3.2E+06	
48	Nicotine	$C_{10}H_{14}N_2$	[M+H]+	163.12297	163.12303	-0.33	2.1E+06	1.8E+07	3.9E+06	1.1E+06	1.2E+06	4.8E+06	4.0E+06	1.3E+06
49	S-(carboxypropyl)-Cysteamine	$C_6H_{13}NO_2S$	[M+H]+	164.07398	164.07403	-0.31	1.4E+06		1.7E+06		1.2E+06		1.1E+06	
50	L-Phenylalanine	$C_9H_{11}NO_2$	[M+H]+	166.08626	166.08631	-0.34	5.9E+06	2.7E+07	5.6E+06		3.1E+06	2.5E+06	3.6E+06	
51	Dihydrothymine	$C_5H_8N_2O_2$	[M+K]+	167.02174	167.02179	-0.32	1.9E+06		2.9E+06		2.0E+06			
52	Formylsalicylic acid	$C_8H_6O_4$	[M+H]+	167.03389	167.03395	-0.36	9.9E+05	1.9E+06		9.9E+05		1.3E+06	1.3E+06	1.0E+06
53	Hydroxy-methyl-oxopentanoic acid	$C_6H_{10}O_4$	[M+Na]+	169.04713	169.04719	-0.33				1.5E+06				1.3E+06
54	Creatine	$C_4H_9N_3O_2$	[M+K]+	170.03264	170.03271	-0.44				1.1E+06				
55	Ectocarpen	$C_{11}H_{16}$	[M+Na]+	171.11442	171.11434	0.47			1.3E+06					

				ESI(+) FT-ICR MS											
**No.**	**Plausible Compound**[a]	**Molecular formula (M)**	**Ion**	**Theor m/z**[b]	**Exp. m/z**[c]	**Δppm**[d]	**SM$_R$H**[e]	**SM$_R$O**[e]	**SM$_P$H**[e]	**SM$_P$O**[e]	**TF$_R$H**[e]	**TF$_R$O**[e]	**TF$_P$H**[e]	**TF$_P$O**[e]
56	Limonene-1,2-diol	$C_{10}H_{18}O_2$	[M+H]+	171.13796	171.13802	-0.37		1.4E+06						
57	Glycylproline	$C_7H_{12}N_2O_3$	[M+H]+	173.09207	173.09214	-0.41		7.8E+06						
58	Propylglutaric acid	$C_8H_{14}O_4$	[M+H]+	175.09649	175.09657	-0.48	1.1E+06							
59	Arginine	$C_6H_{14}N_4O_2$	[M+H]+	175.11895	175.11901	-0.30	4.0E+07	4.9E+07	4.6E+07	3.5E+06	3.5E+07	8.5E+06	1.4E+07	2.7E+06
60	Dimethyl-2-aminoethylphosphonate	$C_4H_{12}NO_3P$	[M+Na]+	176.04470	176.04477	-0.41	1.5E+06		1.2E+06		1.8E+06			
61	Citrulline	$C_6H_{13}N_3O_3$	[M+H]+	176.10297	176.10302	-0.32	4.0E+06	3.5E+07	3.5E+06	1.0E+06	1.8E+06	4.1E+06	4.9E+06	9.0E+05
62	Serotonin	$C_{10}H_{12}N_2O$	[M+H]+	177.10224	177.10230	-0.34	1.8E+06		1.7E+06		1.3E+06		1.8E+06	
63	Hydroxy-methylglutamate	$C_6H_{11}NO_5$	[M+H]+	178.07100	178.07105	-0.29	2.9E+06				2.2E+06		3.2E+06	
64	Methoxycinnamic acid	$C_{10}H_{10}O_3$	[M+H]+	179.07027	179.07033	-0.33		1.2E+06						
65	Hexylphenol	$C_{12}H_{18}O$	[M+H]+	179.14304	179.14299	0.27		1.1E+06				1.2E+06		1.1E+06
66	Glucosamine	$C_6H_{13}NO_5$	[M+H]+	180.08665	180.08671	-0.32			1.3E+06		1.2E+06		1.8E+06	
67	Oxooctanoic acid	$C_8H_{14}O_3$	[M+Na]+	181.08352	181.08357	-0.30						1.3E+06		
68	Methyl-hydroxy-formylpyridine-carboxylate	$C_8H_7NO_4$	[M+H]+	182.04478	182.04485	-0.33	9.6E+05				1.6E+06			
69	Tyrosine	$C_9H_{11}NO_3$	[M+H]+	182.08117	182.08122	-0.29	2.4E+06	3.1E+07	2.7E+06	1.2E+06	1.6E+06	3.6E+06	2.0E+06	
70	Dimethylglutaric acid	$C_7H_{12}O_4$	[M+Na]+	183.06278	183.06283	-0.25				1.3E+06	1.1E+06	2.9E+06		2.0E+06
71	Oxo-campholide	$C_{10}H_{14}O_3$	[M+H]+	183.10157	183.10173	-0.87			1.5E+06		1.9E+06			
72	Indole-ethanol	$C_{10}H_{11}NO$	[M+Na]+	184.07328	184.07339	-0.57	1.1E+06							
73	Solerol	$C_6H_{10}O_4$	[M+K]+	185.02107	185.02113	-0.33		1.5E+07						
74	beta-L-Oleandropyranose;Oleandrose ([M+Na]+)	$C_7H_{14}O_4$	[M+Na]+	185.07843	185.07849	-0.33				9.4E+05		1.3E+06		
75	Citronellyl formate	$C_{11}H_{20}O_2$	[M+H]+	185.15361	185.15367	-0.34		1.8E+06						
76	D-O-Phosphoserine	$C_3H_8NO_6P$	[M+H]+	186.01620	186.01638	-0.94	2.2E+07		8.3E+06		2.0E+07		3.5E+06	
77	Hydroxycinnamic acid	$C_9H_8O_3$	[M+Na]+	187.03656	187.03656	0.01	1.4E+06		1.7E+06		1.5E+06			
78	Mevalonic acid	$C_6H_{12}O_4$	[M+K]+	187.03672	187.03659	0.71			1.7E+06		1.5E+06			
79	Methyl-decanoic acid	$C_{11}H_{22}O_2$	[M+H]+	187.16926	187.16920	0.30		9.9E+05						
80	Monomethyl-arginine	$C_7H_{16}N_4O_2$	[M+H]+	189.13460	189.13466	-0.28	1.1E+06		9.6E+05					
81	Citric acid	$C_6H_8O_7$	[M+H]+	193.03428	193.03433	-0.28	1.1E+06		1.1E+06				2.2E+06	
82	Tridecatrienal	$C_{13}H_{20}O$	[M+H]+	193.15869	193.15876	-0.35		3.0E+06						
83	Oxononanoic acid	$C_9H_{16}O_3$	[M+Na]+	195.09917	195.09924	-0.36		3.4E+06				1.6E+06		
84	Allantoin	$C_4H_6N_4O_3$	[M+K]+	197.00715	197.00722	-0.36			1.4E+06		1.2E+06			

No.	Plausible Compound[a]	Molecular formula (M)	Ion	Theor m/z[b]	Exp. m/z[c]	Δppm[d]	SM$_R$H[e]	SM$_R$O[e]	SM$_P$H[e]	SM$_P$O[e]	TF$_R$H[e]	TF$_R$O[e]	TF$_P$H[e]	TF$_P$O[e]
				ESI(+) FT-ICR MS										
85	Suberic acid	$C_8H_{14}O_4$	[M+Na]+	197.07843	197.07849	-0.31				1.2E+06		3.2E+06		1.7E+06
86	N-Acetylornithine	$C_7H_{14}N_2O_3$	[M+Na]+	197.08966	197.08975	-0.42				9.4E+05				1.0E+06
87	Hydroxy pelargonic acid	$C_9H_{18}O_3$	[M+Na]+	197.11482	197.11488	-0.33						1.2E+06		
88	Terpinyl acetate	$C_{12}H_{20}O_2$	[M+H]+	197.15361	197.15367	-0.32		2.0E+06						
89	Ascorbic acid	$C_6H_8O_6$	[M+Na]+	199.02131	199.02137	-0.28				7.0E+05				3.0E+06
90	Lauroleic acid	$C_{12}H_{22}O_2$	[M+H]+	199.16926	199.16931	-0.27		2.2E+06						
91	D-galacto-Hexodialdose	$C_6H_{10}O_6$	[M+Na]+	201.03696	201.03704	-0.40					1.1E+06			
92	Sulfobenzoate	$C_7H_6O_5S$	[M+H]+	203.00087	203.00087	0.00	1.8E+06				2.7E+06		2.4E+06	
93	Aldohexose	$C_6H_{12}O_6$	[M+Na]+	203.05261	203.05267	-0.32	8.3E+08	1.3E+07	1.2E+09	1.6E+06	1.2E+09	2.4E+06	1.1E+09	3.8E+06
94	Metamitron	$C_{10}H_{10}N_4O$	[M+H]+	203.09274	203.09278	-0.21							3.0E+06	
95	alpha-Curcumene	$C_{15}H_{22}$	[M+H]+	203.17943	203.17946	-0.18	1.1E+06	1.7E+06	1.7E+06		1.5E+06	2.3E+06	1.5E+06	
96	Hydroxyfluorene	$C_{13}H_{10}O$	[M+Na]+	205.06239	205.06246	-0.36				7.6E+05				2.6E+06
97	Sorbitol	$C_6H_{14}O_6$	[M+Na]+	205.06826	205.06832	-0.30	1.5E+06	4.6E+06	2.8E+06	2.3E+06	1.8E+06	1.4E+06	3.4E+06	
98	Tryptophan	$C_{11}H_{12}N_2O_2$	[M+H]+	205.09715	205.09721	-0.29	1.5E+07	1.8E+07	1.3E+07	8.4E+05	1.1E+07	2.3E+06	9.8E+06	
99	Caryophyllene	$C_{15}H_{24}$	[M+H]+	205.19508	205.19513	-0.26	1.2E+06	2.4E+06	1.4E+06	6.5E+05		2.9E+06	1.2E+06	1.1E+06
100	Oxo-decenoic acid	$C_{10}H_{16}O_3$	[M+Na]+	207.09917	207.09922	-0.27						1.2E+06		
101	Actinamine	$C_8H_{18}N_2O_4$	[M+H]+	207.13393	207.13399	-0.27			1.2E+06		1.2E+06			
102	Hydroxybiphenyl	$C_{12}H_{10}O$	[M+K]+	209.03632	209.03638	-0.27					1.3E+06			
103	Carboxycyclohexyl-acetic acid	$C_9H_{14}O_4$	[M+Na]+	209.07843	209.07850	-0.35				5.0E+05	1.0E+06	1.3E+06	1.0E+06	1.1E+06
104	Oxodecanoate	$C_{10}H_{18}O_3$	[M+Na]+	209.11482	209.11487	-0.26				8.0E+05		1.0E+06		
105	(Acetamidomethylene)succinate	$C_7H_9NO_5$	[M+Na]+	210.03729	210.03736	-0.29	1.4E+06				1.3E+06			
106	Butenyl-methyl-threonine	$C_9H_{17}NO_3$	[M+Na]+	210.11006	210.11014	-0.36				7.9E+05	3.2E+06	1.8E+06	1.6E+06	1.3E+06
107	Azelaic acid	$C_9H_{16}O_4$	[M+Na]+	211.09408	211.09414	-0.31	1.5E+06		1.4E+06	6.2E+05	1.9E+06	2.4E+06	1.6E+06	1.2E+06
108	Glycyl-L-leucine	$C_8H_{16}N_2O_3$	[M+Na]+	211.10531	211.10537	-0.27						1.8E+06		
109	Tridecynoic acid	$C_{13}H_{22}O_2$	[M+H]+	211.16926	211.16934	-0.40		3.7E+06						
110	Propylglutaric acid	$C_8H_{14}O_4$	[M+K]+	213.05237	213.05242	-0.24			1.1E+06		1.2E+06			
111	Citrulline	$C_6H_{13}N_3O_3$	[M+K]+	214.05885	214.05891	-0.28	5.9E+06		1.1E+07	7.9E+05	5.5E+06	2.4E+06	4.4E+06	
112	Quinic acid	$C_7H_{12}O_6$	[M+Na]+	215.05261	215.05266	-0.22	1.1E+06				1.4E+06		1.2E+06	
113	Methyl lauric acid	$C_{13}H_{26}O_2$	[M+H]+	215.20056	215.20063	-0.32		2.0E+06				1.1E+06		

				ESI(+) FT-ICR MS										
**No.**	**Plausible Compound**[a]	**Molecular formula (M)**	**Ion**	**Theor m/z**[b]	**Exp. m/z**[c]	**Δppm**[d]	**SM_RH**[e]	**SM_RO**[e]	**SM_PH**[e]	**SM_PO**[e]	**TF_RH**[e]	**TF_RO**[e]	**TF_PH**[e]	**TF_PO**[e]
114	Dihydroxydibenzothiophene	$C_{12}H_8O_2S$	[M+H]+	217.03178	217.03195	-0.80	1.2E+06				1.4E+06			
115	Methyl-myo-inositol	$C_7H_{14}O_6$	[M+Na]+	217.06826	217.06833	-0.30	1.3E+07		5.5E+07		2.9E+07		1.7E+07	
116	Oxidized Latia luciferin	$C_{13}H_{22}O$	[M+Na]+	217.15629	217.15636	-0.32				1.2E+06		2.3E+06		
117	Vulgraon B	$C_{16}H_{24}$	[M+H]+	217.19508	217.19514	-0.30	1.1E+06	2.9E+06	1.4E+06	4.8E+05		2.9E+06	1.3E+06	1.2E+06
118	Salicyluric acid	$C_9H_9NO_4$	[M+Na]+	218.04238	218.04259	-0.97			1.3E+06					
119	Aldohexose	$C_6H_{12}O_6$	[M+K]+	219.02655	219.02661	-0.28	5.1E+08	1.0E+07	7.4E+08		5.9E+08		6.3E+08	2.4E+06
120	N-Acetylserotonin	$C_{12}H_{14}N_2O_2$	[M+H]+	219.11280	219.11281	-0.03					1.0E+06			
121	Amino-dinitrotoluene	$C_7H_7N_3O_4$	[M+Na]+	220.03288	220.03287	0.01	1.1E+06		1.6E+06		1.4E+06			
122	Dihydroxy-dihydro-p-cumate	$C_{10}H_{14}O_4$	[M+Na]+	221.07843	221.07852	-0.41		1.7E+06						
123	Prilocaine	$C_{13}H_{20}N_2O$	[M+H]+	221.16484	221.16488	-0.18						1.0E+06		
124	Dodecanamide	$C_{12}H_{25}NO$	[M+Na]+	222.18284	222.18289	-0.26	2.3E+06			3.6E+06	2.4E+06	3.4E+06	2.1E+06	2.5E+06
125	cis-5-Decenedioic acid	$C_{10}H_{16}O_4$	[M+Na]+	223.09408	223.09414	-0.28	1.0E+06			5.3E+05	1.3E+06	2.0E+06	1.0E+06	1.3E+06
126	Amylmercaptopurine	$C_{10}H_{14}N_4S$	[M+H]+	223.10119	223.10119	0.02	1.6E+06							
127	Oxo-undecanoic acid	$C_{11}H_{20}O_3$	[M+Na]+	223.13047	223.13052	-0.26				8.5E+05		1.4E+06		1.6E+06
128	Carboxycyclohexyl-acetic acid	$C_9H_{14}O_4$	[M+K]+	225.05237	225.05243	-0.25	1.3E+06		1.2E+06					
129	Sebacic acid	$C_{10}H_{18}O_4$	[M+Na]+	225.10973	225.10979	-0.28	1.1E+06			7.0E+05	1.5E+06	2.3E+06		1.4E+06
130	Tetradecadienoic acid	$C_{14}H_{24}O_2$	[M+H]+	225.18491	225.18499	-0.37		1.8E+06						
131	N-Heptanoylglycine	$C_9H_{17}NO_3$	[M+K]+	226.08400	226.08407	-0.27	1.4E+06				1.7E+06			
132	Oxosuberate	$C_8H_{12}O_5$	[M+K]+	227.03163	227.03169	-0.25					1.2E+06			
133	D-Tryptophan	$C_{11}H_{12}N_2O_2$	[M+Na]+	227.07910	227.07921	-0.51			1.9E+06		1.6E+06		1.4E+06	
134	Acetyl-hydroxy-lysine	$C_8H_{16}N_2O_4$	[M+Na]+	227.10023	227.10028	-0.24	1.3E+06			1.5E+06		1.0E+06		
135	Diazacyclotetradecanedione	$C_{12}H_{22}N_2O_2$	[M+H]+	227.17540	227.17549	-0.35				1.6E+06		2.5E+06		
136	Myristoleic acid	$C_{14}H_{26}O_2$	[M+H]+	227.20056	227.20062	-0.30	2.3E+06	3.9E+06	1.4E+06		1.5E+06		1.5E+06	
137	Methylcitric acid	$C_7H_{10}O_7$	[M+Na]+	229.03187	229.03194	-0.29	3.1E+06		5.8E+06		3.3E+06		5.1E+06	
138	Serine-phosphoethanolamine	$C_5H_{13}N_2O_6P$	[M+H]+	229.05840	229.05859	-0.83	1.1E+06							
139	Acetyleugenol	$C_{12}H_{14}O_3$	[M+Na]+	229.08352	229.08374	-0.96		3.0E+06	1.1E+06					
140	Myristic acid	$C_{14}H_{28}O_2$	[M+H]+	229.21621	229.21627	-0.29	1.4E+06	5.8E+06	1.4E+06	9.6E+05		2.4E+06	1.3E+06	1.1E+06
141	Xestoaminol C	$C_{14}H_{31}NO$	[M+H]+	230.24784	230.24792	-0.33	2.4E+06	7.2E+06	1.4E+06			2.1E+06		
142	Citric acid	$C_6H_8O_7$	[M+K]+	230.99016	230.99023	-0.27	4.1E+07		5.2E+07		1.4E+07		7.6E+07	

				ESI(+) FT-ICR MS										
**No.**	**Plausible Compound**[a]	**Molecular formula (M)**	**Ion**	**Theor m/z**[b]	**Exp. m/z**[c]	**Δppm**[d]	**SM$_R$H**[e]	**SM$_R$O**[e]	**SM$_P$H**[e]	**SM$_P$O**[e]	**TF$_R$H**[e]	**TF$_R$O**[e]	**TF$_P$H**[e]	**TF$_P$O**[e]
143	Heptyloxyphenol	$C_{13}H_{20}O_2$	[M+Na]+	231.13555	231.13563	-0.33				5.1E+05		2.2E+06		2.0E+06
144	L-Arabinitol phosphate	$C_5H_{13}O_8P$	[M+H]+	233.04208	233.04226	-0.76	4.2E+06		1.4E+07		7.2E+06		4.3E+06	
145	Sinapyl alcohol	$C_{11}H_{14}O_4$	[M+Na]+	233.07843	233.07864	-0.90	1.2E+06							
146	Aplotaxene	$C_{17}H_{28}$	[M+H]+	233.22638	233.22644	-0.28		2.3E+06		5.1E+05		2.5E+06		
147	Perseitol	$C_7H_{16}O_7$	[M+Na]+	235.07882	235.07889	-0.28	5.3E+06		7.7E+06		7.6E+06		6.6E+06	
148	Hexadecatrienal	$C_{16}H_{26}O$	[M+H]+	235.20564	235.20570	-0.25		3.1E+06						
149	Carboxin	$C_{12}H_{13}NO_2S$	[M+H]+	236.07398	236.07403	-0.23						3.4E+06		
150	Methyl lauric acid	$C_{13}H_{26}O_2$	[M+Na]+	237.18250	237.18258	-0.31				5.0E+05				1.4E+06
151	Hexadecadienal	$C_{16}H_{28}O$	[M+H]+	237.22129	237.22136	-0.29		5.5E+06						
152	Acetyl-arginine	$C_8H_{16}N_4O_3$	[M+Na]+	239.11146	239.11151	-0.20				1.4E+06				
153	Undecanedioic acid	$C_{11}H_{20}O_4$	[M+Na]+	239.12538	239.12546	-0.35	1.2E+06			1.1E+06	1.6E+06	1.5E+06		1.1E+06
154	Campherene-diol	$C_{15}H_{26}O_2$	[M+H]+	239.20056	239.20064	-0.35		1.6E+06						
155	Hexadecenal	$C_{16}H_{30}O$	[M+H]+	239.23694	239.23702	-0.31		2.0E+06				1.8E+06		1.1E+06
156	Anatalline	$C_{15}H_{17}N_3$	[M+H]+	240.14952	240.14960	-0.32		5.0E+06	2.0E+06	1.2E+06		2.2E+06	1.1E+06	
157	Dihydroxy-dihydrodibenzothiophene	$C_{12}H_{10}O_2S$	[M+Na]+	241.02937	241.02944	-0.29				6.8E+06		3.6E+07		1.0E+07
158	Tridecenyl acetate	$C_{15}H_{28}O_2$	[M+H]+	241.21621	241.21628	-0.28		4.0E+06	1.1E+06					
159	(4E,6E,d14:2) sphingosine	$C_{14}H_{27}NO_2$	[M+H]+	242.21146	242.21153	-0.31			1.2E+06		1.6E+06			
160	Canthin-one	$C_{14}H_8N_2O$	[M+Na]+	243.05288	243.05309	-0.86	2.6E+06		2.5E+06		2.2E+06			
161	Hydroxy-tryptophan	$C_{11}H_{12}N_2O_3$	[M+Na]+	243.07401	243.07423	-0.88	2.3E+06	2.1E+06	1.7E+06	1.6E+06			1.3E+06	
162	Methyl myristic acid	$C_{15}H_{30}O_2$	[M+H]+	243.23186	243.23191	-0.22	1.7E+06	8.7E+06	1.7E+06			2.8E+06	1.4E+06	9.6E+05
163	Acetyl-glucosamine	$C_8H_{15}NO_6$	[M+Na]+	244.07916	244.07923	-0.27	1.7E+06				2.0E+06			
164	Cytidine	$C_9H_{13}N_3O_5$	[M+H]+	244.09280	244.09287	-0.28	1.5E+07		5.5E+06		8.5E+06		7.4E+06	
165	Undecanoylglycine	$C_{13}H_{25}NO_3$	[M+H]+	244.19072	244.19079	-0.28	1.8E+07	7.4E+07	9.3E+06	9.0E+06	5.8E+06	8.7E+06	1.9E+07	3.8E+06
166	Amino-tetradecanoic acid	$C_{14}H_{29}NO_2$	[M+H]+	244.22711	244.22719	-0.35		1.9E+07						
167	Apiole	$C_{12}H_{14}O_4$	[M+Na]+	245.07843	245.07850	-0.30				5.0E+05		1.5E+06		1.1E+06
168	Hydroxy-oxo-ionone	$C_{13}H_{18}O_3$	[M+Na]+	245.11482	245.11488	-0.26				1.7E+06	1.3E+06	4.0E+06	1.0E+06	2.7E+06
169	Glutamyl-valine	$C_{10}H_{18}N_2O_5$	[M+H]+	247.12885	247.12892	-0.29	1.1E+06							
170	Propylideneisolongifolane	$C_{18}H_{30}$	[M+H]+	247.24203	247.24210	-0.29		2.5E+06				1.5E+06		
171	Linamarin	$C_{10}H_{17}NO_6$	[M+H]+	248.11286	248.11294	-0.31			1.2E+06					

		ESI(+) FT-ICR MS												
**No.**	**Plausible Compound[a]**	**Molecular formula (M)**	**Ion**	**Theor m/z[b]**	**Exp. m/z[c]**	**Δppm[d]**	**SM_RH^e**	**SM_RO^e**	**SM_PH^e**	**SM_PO^e**	**TF_RH^e**	**TF_RO^e**	**TF_PH^e**	**TF_PO^e**
172	Chorismic acid	$C_{10}H_{10}O_6$	[M+Na]+	249.03696	249.03718	-0.88	1.4E+07		3.0E+06		1.4E+07		3.3E+06	
173	Furyl-nitro-furylacrylamide	$C_{11}H_8N_2O_5$	[M+H]+	249.05060	249.05073	-0.53		2.7E+06						
174	Diazacyclotetradecanedione	$C_{12}H_{22}N_2O_2$	[M+Na]+	249.15735	249.15742	-0.30			1.1E+06		1.8E+06			4.4E+06
175	Myristoleic acid	$C_{14}H_{26}O_2$	[M+Na]+	249.18250	249.18258	-0.32				8.8E+05				1.8E+06
176	Dubamine	$C_{16}H_{11}NO_2$	[M+H]+	250.08626	250.08633	-0.30		4.5E+06		7.2E+05		3.7E+06		
177	Hydroxy-carboxy-oxo-methylocta-dienoate	$C_{10}H_{12}O_6$	[M+Na]+	251.05261	251.05282	-0.84	2.2E+06		3.0E+06		2.1E+06		2.0E+06	
178	Deoxyindenestrol	$C_{18}H_{18}O$	[M+H]+	251.14304	251.14317	-0.51	1.3E+06		1.3E+06					
179	Oxo-tridecanoic acid	$C_{13}H_{24}O_3$	[M+Na]+	251.16177	251.16181	-0.18						1.5E+06		
180	Muramic acid	$C_9H_{17}NO_7$	[M+H]+	252.10778	252.10784	-0.24	2.6E+06		2.0E+06		1.6E+06		1.8E+06	
181	N-Decanoylglycine	$C_{12}H_{23}NO_3$	[M+Na]+	252.15701	252.15711	-0.38				1.4E+06				
182	Dodecanedioic acid	$C_{12}H_{22}O_4$	[M+Na]+	253.14103	253.14109	-0.25	1.2E+06			1.0E+06	1.7E+06	2.2E+06		1.5E+06
183	Tetramethyl-undecandiol	$C_{14}H_{30}O_2$	[M+Na]+	253.21380	253.21386	-0.21						2.6E+06		2.0E+06
184	Palmitolinoleic acid	$C_{16}H_{28}O_2$	[M+H]+	253.21621	253.21627	-0.23		6.5E+06				1.5E+06		
185	Goniothalenol	$C_{13}H_{12}O_4$	[M+Na]+	255.06278	255.06299	-0.81	1.4E+06		1.5E+06		1.2E+06			
186	Hexadecenoic acid	$C_{16}H_{30}O_2$	[M+H]+	255.23186	255.23192	-0.24	2.0E+06	2.2E+07	2.3E+06	7.6E+05		3.7E+06	2.4E+06	1.4E+06
187	Palmitic acid	$C_{16}H_{32}O_2$	[M+H]+	257.24751	257.24758	-0.28	2.2E+06	4.7E+07	1.9E+06	1.0E+06		3.7E+06	2.0E+06	1.3E+06
188	Lophophorine	$C_{13}H_{17}NO_3$	[M+Na]+	258.11006	258.11015	-0.33			1.3E+06					
189	Galactosamine phosphate	$C_6H_{14}NO_8P$	[M+H]+	260.05298	260.05316	-0.69	4.0E+06		2.6E+06		3.3E+06		2.3E+06	
190	Acetyl-glucosaminate	$C_8H_{15}NO_7$	[M+Na]+	260.07407	260.07414	-0.26			1.3E+06					
191	Ketose-phosphate	$C_6H_{13}O_9P$	[M+H]+	261.03700	261.03711	-0.43	1.4E+06		1.1E+06				1.9E+06	
192	Deoxy-manno-octulosonate	$C_8H_{14}O_8$	[M+Na]+	261.05809	261.05815	-0.25	1.7E+06				2.1E+06		3.1E+06	
193	Methoxychalcone	$C_{16}H_{14}O_2$	[M+Na]+	261.08860	261.08882	-0.83	1.5E+06	1.5E+06	1.2E+06	1.4E+06	2.0E+06			1.0E+06
194	Androstane	$C_{19}H_{32}$	[M+H]+	261.25768	261.25775	-0.27		3.2E+06	1.8E+06	5.9E+05		3.5E+06	1.3E+06	1.3E+06
195	Tridecenyl acetate	$C_{15}H_{28}O_2$	[M+Na]+	263.19815	263.19822	-0.26				1.7E+06				2.7E+06
196	Dodecylphenol	$C_{18}H_{30}O$	[M+H]+	263.23694	263.23700	-0.22		7.3E+06				1.2E+06		
197	Selagine	$C_{15}H_{18}N_2O$	[M+Na]+	265.13113	265.13133	-0.73				3.0E+06		5.3E+06		1.1E+06
198	Keto myristic acid	$C_{14}H_{26}O_3$	[M+Na]+	265.17742	265.17749	-0.28				6.1E+05				1.7E+06
199	Norlinolenic acid	$C_{17}H_{28}O_2$	[M+H]+	265.21621	265.21627	-0.24		2.3E+06						
200	Octadecadienal	$C_{18}H_{32}O$	[M+H]+	265.25259	265.25267	-0.29		9.8E+06						

				ESI(+) FT-ICR MS										
No.	Plausible Compound[a]	Molecular formula (M)	Ion	Theor m/z[b]	Exp. m/z[c]	Δppm[d]	SM$_R$H[e]	SM$_R$O[e]	SM$_P$H[e]	SM$_P$O[e]	TF$_R$H[e]	TF$_R$O[e]	TF$_P$H[e]	TF$_P$O[e]
201	Isobutylphendienamide	$C_{16}H_{21}NO$	[M+Na]+	266.15153	266.15175	-0.82	9.4E+06	6.6E+06		6.8E+06	4.8E+06	6.1E+06	9.3E+06	3.3E+06
202	Amino-tetradecanoic acid	$C_{14}H_{29}NO_2$	[M+Na]+	266.20905	266.20912	-0.26	1.5E+06		1.2E+06					
203	Methyl-dodecanedioic acid	$C_{13}H_{24}O_4$	[M+Na]+	267.15668	267.15673	-0.17			1.2E+06			1.4E+06		1.3E+06
204	Epoxy-trimethyltrideca-dienoicacid	$C_{16}H_{26}O_3$	[M+H]+	267.19547	267.19553	-0.22		2.1E+06						
205	Pentadecadienyl acetate	$C_{17}H_{30}O_2$	[M+H]+	267.23186	267.23194	-0.31		2.2E+06						
206	Octadecenal	$C_{18}H_{34}O$	[M+H]+	267.26824	267.26830	-0.22								1.4E+06
207	Neuraminic acid	$C_9H_{17}NO_8$	[M+H]+	268.10269	268.10279	-0.36	1.8E+06							
208	Adenosine	$C_{10}H_{13}N_5O_4$	[M+H]+	268.10403	268.10410	-0.26	2.3E+07		1.3E+07		1.5E+07		6.8E+06	
209	Oxo-pentyl-cyclopentanehexanoic acid	$C_{16}H_{28}O_3$	[M+H]+	269.21112	269.21121	-0.33		5.0E+06						
210	Methyl-hexadecenoic acid	$C_{17}H_{32}O_2$	[M+H]+	269.24751	269.24759	-0.30		1.1E+07	1.2E+06			1.8E+06		
211	Meperidine	$C_{15}H_{21}NO_2$	[M+Na]+	270.14645	270.14667	-0.82	2.5E+06						2.0E+06	
212	Keto palmitic acid	$C_{16}H_{30}O_3$	[M+H]+	271.22677	271.22683	-0.22		7.6E+06				2.6E+06		
213	Erogorgiaene	$C_{20}H_{30}$	[M+H]+	271.24203	271.24211	-0.31		4.6E+06	1.3E+06			5.2E+06	1.8E+06	2.1E+06
214	Methyl palmitic acid	$C_{17}H_{34}O_2$	[M+H]+	271.26316	271.26324	-0.29		2.3E+07				1.6E+06		
215	Amino-hexadecanoic acid	$C_{16}H_{33}NO_2$	[M+H]+	272.25841	272.25849	-0.29		9.2E+07				1.7E+06		
216	Citrinin	$C_{13}H_{14}O_5$	[M+Na]+	273.07334	273.07335	-0.02	1.1E+06							
217	Glycinol	$C_{15}H_{12}O_5$	[M+H]+	273.07575	273.07582	-0.26							3.1E+06	
218	Methylripariochromene A ([M+Na]+)	$C_{14}H_{18}O_4$	[M+Na]+	273.10973	273.10981	-0.29	2.5E+06	1.8E+06		9.2E+05	2.4E+06	3.3E+06	2.6E+06	2.3E+06
219	Hydroxy palmitic acid	$C_{16}H_{32}O_3$	[M+H]+	273.24242	273.24250	-0.29		2.1E+06						
220	Pimaradiene	$C_{20}H_{32}$	[M+H]+	273.25768	273.25774	-0.21	1.9E+06	6.3E+06	2.2E+06	9.2E+05		5.0E+06	2.2E+06	1.5E+06
221	Glutamyl-glutamine	$C_{10}H_{17}N_3O_6$	[M+H]+	276.11901	276.11907	-0.21	1.4E+06							
222	Phosphogluconic acid	$C_6H_{13}O_{10}P$	[M+H]+	277.03191	277.03209	-0.65	1.5E+06				1.4E+06		1.6E+06	
223	Gamma Glutamylglutamic acid	$C_{10}H_{16}N_2O_7$	[M+H]+	277.10303	277.10309	-0.21	1.4E+06				1.6E+06			
224	methyl-[(formyl-vinyl)-hydroxy-oxo-cyclopentyl]-butanoate	$C_{13}H_{18}O_5$	[M+Na]+	277.10464	277.10487	-0.83	2.2E+06	1.0E+07					1.9E+06	
225	Chrysanthetriol	$C_{15}H_{26}O_3$	[M+H]+	277.17742	277.17748	-0.23						1.3E+06		
226	Hydroxybutanyl-isolongifol-ene	$C_{19}H_{32}O$	[M+H]+	277.25259	277.25266	-0.24		3.5E+06						
227	Dihydroxyflavanone	$C_{15}H_{12}O_4$	[M+Na]+	279.06278	279.06302	-0.86					1.3E+06			
228	alhpa-tocopheronolactone	$C_{16}H_{22}O_4$	[M+H]+	279.15909	279.15915	-0.24	8.8E+06	2.5E+07		4.2E+06	4.1E+06	1.5E+07	9.9E+06	4.5E+06

				ESI(+) FT-ICR MS											
No.	Plausible Compound[a]	Molecular formula (M)	Ion	Theor m/z[b]	Exp. m/z[c]	Δppm[d]	SM$_R$H[e]	SM$_R$O[e]	SM$_P$H[e]	SM$_P$O[e]	TF$_R$H[e]	TF$_R$O[e]	TF$_P$H[e]	TF$_P$O[e]
229	keto pentadecanoic acid	$C_{15}H_{28}O_3$	[M+Na]+	279.19307	279.19312	-0.20						1.6E+06	1.1E+06	
230	Linolenic acid	$C_{18}H_{30}O_2$	[M+H]+	279.23186	279.23193	-0.26		4.9E+06						
231	Serratine	$C_{16}H_{25}NO_3$	[M+H]+	280.19072	280.19083	-0.39		1.6E+06						
232	Linoleamide	$C_{18}H_{33}NO$	[M+H]+	280.26349	280.26358	-0.32		6.9E+06						
233	Apigeniflavan	$C_{15}H_{14}O_4$	[M+Na]+	281.07843	281.07867	-0.86		4.5E+06						
234	Glycero-Phosphocholine	$C_8H_{21}NO_6P$	[M+Na]+	281.09987	281.09966	0.75		2.7E+06						
235	Methylbatatasin III	$C_{16}H_{18}O_3$	[M+Na]+	281.11482	281.11504	-0.80		4.3E+06						
236	Keto myristic acid	$C_{14}H_{26}O_3$	[M+K]+	281.15135	281.15144	-0.31		6.7E+06						
237	Tetradecanedioic acid	$C_{14}H_{26}O_4$	[M+Na]+	281.17233	281.17238	-0.19	1.6E+06		1.5E+06	9.1E+05	1.7E+06		1.5E+06	
238	Hydroxy-pentadecanoic acid	$C_{15}H_{30}O_3$	[M+Na]+	281.20872	281.20881	-0.32		4.4E+06				2.8E+06		1.7E+06
239	Hydroxy-heptadecatrienoic acid	$C_{17}H_{28}O_3$	[M+H]+	281.21112	281.21120	-0.28		2.1E+06						
240	Linoleic acid	$C_{18}H_{32}O_2$	[M+H]+	281.24751	281.24760	-0.33		1.9E+07				2.1E+06		
241	Heptyl-hydroxy-quinolone	$C_{16}H_{21}NO_2$	[M+Na]+	282.14645	282.14667	-0.80	5.1E+06	1.7E+07	4.5E+06	3.1E+06	3.0E+06	1.9E+06	3.7E+06	
242	Oleamide	$C_{18}H_{35}NO$	[M+H]+	282.27914	282.27924	-0.35		2.2E+07						
243	Hemigossypol	$C_{15}H_{16}O_4$	[M+Na]+	283.09408	283.09431	-0.81		4.3E+06						
244	Falcarindiol	$C_{17}H_{24}O_2$	[M+Na]+	283.16685	283.16706	-0.74		1.7E+06						
245	Oleic acid	$C_{18}H_{34}O_2$	[M+H]+	283.26316	283.26322	-0.21	1.6E+06	4.2E+07	1.6E+06	7.4E+05		2.8E+06	1.5E+06	
246	Nonadecanal	$C_{19}H_{38}O$	[M+H]+	283.29954	283.29961	-0.24		1.6E+06						
247	Guanosine	$C_{10}H_{13}N_5O_5$	[M+H]+	284.09895	284.09901	-0.22	1.0E+07				7.3E+06		3.9E+06	
248	Acrifoline	$C_{16}H_{23}NO_2$	[M+Na]+	284.16210	284.16237	-0.95		1.5E+06						
249	Stearamide	$C_{18}H_{37}NO$	[M+H]+	284.29479	284.29487	-0.28		4.8E+06			1.6E+06			
250	Coformycin	$C_{11}H_{16}N_4O_5$	[M+H]+	285.11935	285.11943	-0.28		8.8E+06				1.4E+06		
251	Dihexyl maleic acid	$C_{16}H_{28}O_4$	[M+H]+	285.20604	285.20613	-0.33		2.6E+06						
252	Methoxy-hexadecenoic acid	$C_{17}H_{32}O_3$	[M+H]+	285.24242	285.24249	-0.23	2.6E+06	4.7E+06				5.3E+06	1.9E+06	1.3E+06
253	Stearic acid	$C_{18}H_{36}O_2$	[M+H]+	285.27881	285.27889	-0.27		4.4E+07				1.7E+06		
254	Buchananine	$C_{12}H_{15}NO_7$	[M+H]+	286.09213	286.09220	-0.26	1.7E+06		1.2E+06		1.5E+06			
255	Octenoylcarnitine	$C_{15}H_{27}NO_4$	[M+H]+	286.20128	286.20137	-0.30		1.3E+07						
256	Myristoylglycine	$C_{16}H_{31}NO_3$	[M+H]+	286.23767	286.23776	-0.31		2.9E+06						
257	C17 Sphingosine	$C_{17}H_{35}NO_2$	[M+H]+	286.27406	286.27414	-0.29		3.0E+07						

				ESI(+) FT-ICR MS										
**No.**	**Plausible Compound[a]**	**Molecular formula (M)**	**Ion**	**Theor m/z[b]**	**Exp. m/z[c]**	**Δppm[d]**	**SM$_R$H[e]**	**SM$_R$O[e]**	**SM$_P$H[e]**	**SM$_P$O[e]**	**TF$_R$H[e]**	**TF$_R$O[e]**	**TF$_P$H[e]**	**TF$_P$O[e]**
258	Spisulosine	$C_{18}H_{39}NO$	[M+H]+	286.31044	286.31051	-0.24		2.0E+06						
259	Dihydroxy-methoxyisoflavanone	$C_{16}H_{14}O_5$	[M+H]+	287.09140	287.09147	-0.24		2.0E+06						
260	Abscisic acid	$C_{15}H_{20}O_4$	[M+Na]+	287.12538	287.12556	-0.63		1.8E+06						
261	Hydroxy-oxo-hexadecanoic acid	$C_{16}H_{30}O_4$	[M+H]+	287.22169	287.22176	-0.26		2.2E+06						
262	Retinol	$C_{20}H_{30}O$	[M+H]+	287.23694	287.23701	-0.24		1.3E+06						
263	Methoxyhexadecanoic acid	$C_{17}H_{34}O_3$	[M+H]+	287.25807	287.25817	-0.34		2.4E+06						
264	Prosopinine	$C_{16}H_{33}NO_3$	[M+H]+	288.25332	288.25338	-0.22	1.8E+06	1.8E+07				2.9E+06	1.3E+06	
265	C17 Sphinganine	$C_{17}H_{37}NO_2$	[M+H]+	288.28971	288.28979	-0.29		1.4E+07						
266	Methoxy-methylisoflavone	$C_{17}H_{14}O_3$	[M+Na]+	289.08352	289.08373	-0.75	2.2E+06	3.6E+06			2.3E+06		2.0E+06	
267	Tributyl phosphate	$C_{12}H_{27}O_4P$	[M+Na]+	289.15392	289.15409	-0.60						3.8E+06		
268	Heptadecynoic acid	$C_{17}H_{30}O_2$	[M+Na]+	289.21380	289.21387	-0.24						1.4E+06		
269	Androstanedione	$C_{19}H_{28}O_2$	[M+H]+	289.21621	289.21627	-0.22						1.1E+06		
270	Flabellidine	$C_{18}H_{28}N_2O$	[M+H]+	289.22744	289.22751	-0.24		1.3E+06						
271	Dihydroretinol	$C_{20}H_{32}O$	[M+H]+	289.25259	289.25268	-0.30		2.7E+06						
272	Mannosylglycerate	$C_9H_{16}O_9$	[M+Na]+	291.06865	291.06873	-0.27	1.6E+06		2.0E+06		2.3E+06		1.4E+06	
273	Saphenic acid	$C_{15}H_{12}N_2O_3$	[M+Na]+	291.07401	291.07391	0.35							1.7E+06	
274	Hydroxybenzopyrene	$C_{20}H_{12}O$	[M+Na]+	291.07804	291.07801	0.08	4.7E+06				4.5E+06		7.7E+06	
275	N-Succinyl-diaminopimelate	$C_{11}H_{18}N_2O_7$	[M+H]+	291.11868	291.11872	-0.15	1.4E+06							
276	Argininosuccinic acid	$C_{10}H_{18}N_4O_6$	[M+H]+	291.12991	291.12999	-0.27		1.4E+06						
277	Palmitolinoleic acid	$C_{16}H_{28}O_2$	[M+K]+	291.17209	291.17217	-0.28		1.3E+07						
278	Oxo-octadecadiynoic acid	$C_{18}H_{26}O_3$	[M+H]+	291.19547	291.19557	-0.34		4.1E+06						
279	Methyl-hexadecenoic acid	$C_{17}H_{32}O_2$	[M+Na]+	291.22945	291.22950	-0.18	1.5E+06			3.4E+06			6.4E+06	4.0E+06
280	Hydroxy-androstanone	$C_{19}H_{30}O_2$	[M+H]+	291.23186	291.23195	-0.32		1.3E+06						
281	Anhydro-N-acetylneuraminic acid	$C_{11}H_{17}NO_8$	[M+H]+	292.10269	292.10274	-0.17	3.7E+06				2.0E+06		1.4E+06	
282	Glucosyl-sn-glycerol	$C_9H_{18}O_8$	[M+K]+	293.06333	293.06339	-0.20	1.3E+06				1.2E+06			
283	Thiamylal	$C_{12}H_{18}N_2O_2S$	[M+K]+	293.07206	293.07183	0.78			1.1E+06					
284	Hydroxy-methoxy-methylflavan	$C_{17}H_{18}O_3$	[M+Na]+	293.11482	293.11504	-0.77	2.5E+06	5.6E+06	2.0E+06		1.6E+06		2.4E+06	
285	Estrone	$C_{18}H_{22}O_2$	[M+Na]+	293.15120	293.15142	-0.75		3.4E+06						
286	Methyl-norandrosta-dienone	$C_{19}H_{26}O$	[M+Na]+	293.18759	293.18782	-0.79				1.5E+06		3.5E+06		1.4E+06

					ESI(+) FT-ICR MS									
**No.**	**Plausible Compound**[a]	**Molecular formula (M)**	**Ion**	**Theor m/z**[b]	**Exp. m/z**[c]	**Δppm**[d]	**SM_RH^e**	**SM_RO^e**	**SM_PH^e**	**SM_PO^e**	**TF_RH^e**	**TF_RO^e**	**TF_PH^e**	**TF_PO^e**
287	Keto palmitic acid	$C_{16}H_{30}O_3$	[M+Na]+	293.20872	293.20878	-0.23	2.7E+06		3.8E+06	1.4E+06	2.3E+06		3.7E+06	2.1E+06
288	Methyl palmitic acid	$C_{17}H_{34}O_2$	[M+Na]+	293.24510	293.24517	-0.22	3.1E+06		2.5E+06	4.8E+06	1.8E+06		3.4E+06	9.0E+06
289	Stearyl alcohol	$C_{18}H_{38}O$	[M+Na]+	293.28149	293.28158	-0.32		2.4E+06						
290	N-Acetylmuramic acid	$C_{11}H_{19}NO_8$	[M+H]+	294.11834	294.11836	-0.06		1.5E+06						
291	2-(Carboxy-(methylammonio)propyl)-histidine	$C_{11}H_{19}N_4O_4$	[M+Na]+	294.12985	294.12963	0.74	2.0E+07				1.3E+07		1.6E+06	
292	Melosatin B	$C_{19}H_{19}NO_2$	[M+H]+	294.14886	294.14898	-0.42	3.5E+06		3.3E+06		2.3E+06			
293	Dihydrobunolol	$C_{17}H_{27}NO_3$	[M+H]+	294.20637	294.20645	-0.27		3.7E+06						
294	Ribose diphosphate	$C_5H_{12}O_{10}P_2$	[M+H]+	294.99785	294.99783	0.06						2.4E+06		
295	Dihydroxy-methoxyflavan	$C_{16}H_{16}O_4$	[M+Na]+	295.09408	295.09432	-0.81		3.0E+06						
296	Tuliposide B	$C_{11}H_{18}O_9$	[M+H]+	295.10236	295.10241	-0.17			3.0E+06				2.8E+06	
297	Aspartame	$C_{14}H_{18}N_2O_5$	[M+H]+	295.12885	295.12895	-0.35		2.4E+06						
298	Methallenestrilphenol	$C_{17}H_{20}O_3$	[M+Na]+	295.13047	295.13070	-0.80		3.6E+06						
299	(Butylbenzofuranyl)(hydroxyphenyl)ketone	$C_{19}H_{18}O_3$	[M+H]+	295.13287	295.13298	-0.37	2.6E+06		1.2E+06		2.0E+06			
300	Methyl-tetradecanedioic acid	$C_{15}H_{28}O_4$	[M+Na]+	295.18798	295.18806	-0.27						1.6E+06		
301	Palmitic acid	$C_{16}H_{32}O_2$	[M+K]+	295.20339	295.20346	-0.23				2.0E+06		3.2E+06		3.0E+06
302	Hydroxy palmitic acid	$C_{16}H_{32}O_3$	[M+Na]+	295.22437	295.22444	-0.24				8.8E+05		2.9E+06		
303	Hydroxylinolenic acid	$C_{18}H_{30}O_3$	[M+H]+	295.22677	295.22686	-0.30		4.2E+06				1.2E+06		
304	Methyl-octadecadienoic acid	$C_{19}H_{34}O_2$	[M+H]+	295.26316	295.26325	-0.32		1.8E+06						
305	Eicosenal	$C_{20}H_{38}O$	[M+H]+	295.29954	295.29963	-0.30		1.7E+06						
306	Hydroxytetrahydrobiopterin	$C_9H_{15}N_5O_4$	[M+K]+	296.07556	296.07584	-0.93	1.4E+06				1.3E+06			
307	(d18:3)sphingosine	$C_{18}H_{33}NO_2$	[M+H]+	296.25841	296.25847	-0.22		4.8E+06						
308	Tetrahydroxyflavan	$C_{15}H_{14}O_5$	[M+Na]+	297.07334	297.07359	-0.83		2.4E+06						
309	Dihydroxy-dimethoxydihydrostilbene	$C_{16}H_{18}O_4$	[M+Na]+	297.10973	297.10997	-0.81		4.0E+06						
310	Hydroxytetradecanedioic acid	$C_{14}H_{26}O_5$	[M+Na]+	297.16724	297.16732	-0.25		5.1E+06				1.5E+06		
311	Dodecanoyl-sn-glycerol	$C_{15}H_{30}O_4$	[M+Na]+	297.20363	297.20373	-0.34		3.1E+06						
312	Hydroxy-octadecadienoic acid	$C_{18}H_{32}O_3$	[M+H]+	297.24242	297.24250	-0.26		7.4E+06				1.9E+06		
313	Methyl-octadecenoic acid	$C_{19}H_{36}O_2$	[M+H]+	297.27881	297.27887	-0.21		4.5E+06				1.2E+06		
314	Phytol	$C_{20}H_{40}O$	[M+H]+	297.31519	297.31527	-0.26		2.9E+06						
315	Methylthioadenosine	$C_{11}H_{15}N_5O_3S$	[M+H]+	298.09684	298.09690	-0.20	4.0E+06		4.9E+06		2.4E+06		2.8E+06	

				ESI(+) FT-ICR MS										
**No.**	**Plausible Compound**[a]	**Molecular formula (M)**	**Ion**	**Theor m/z**[b]	**Exp. m/z**[c]	**Δppm**[d]	**SM$_R$H**[e]	**SM$_R$O**[e]	**SM$_P$H**[e]	**SM$_P$O**[e]	**TF$_R$H**[e]	**TF$_R$O**[e]	**TF$_P$H**[e]	**TF$_P$O**[e]
316	Lycoflexine	$C_{17}H_{25}NO_2$	[M+Na]+	298.17775	298.17795	-0.68		1.4E+07	1.5E+06	1.7E+06			1.5E+06	
317	N-(oxododecanoyl) homoserine lactone	$C_{16}H_{27}NO_4$	[M+H]+	298.20128	298.20138	-0.32		3.9E+06						
318	Palmitoleoyl Ethanolamide	$C_{18}H_{35}NO_2$	[M+H]+	298.27406	298.27414	-0.28		3.7E+07						
319	Ranunculin	$C_{11}H_{16}O_8$	[M+Na]+	299.07374	299.07378	-0.14					1.6E+06			
320	Dihydroxy myristoic acid	$C_{14}H_{28}O_4$	[M+K]+	299.16192	299.16186	0.20						1.2E+06		1.3E+06
321	Heptadecenedioic acid	$C_{17}H_{30}O_4$	[M+H]+	299.22169	299.22180	-0.38		1.7E+06						
322	Oxooctadecanoic acid	$C_{18}H_{34}O_3$	[M+H]+	299.25807	299.25813	-0.19	2.8E+06	9.3E+06	2.4E+06	8.6E+05		5.4E+06	2.2E+06	1.4E+06
323	Methyl stearic acid	$C_{19}H_{38}O_2$	[M+H]+	299.29446	299.29452	-0.21		9.0E+06				1.3E+06		
324	Lycopsamine	$C_{15}H_{25}NO_5$	[M+H]+	300.18055	300.18064	-0.30		4.5E+06						
325	Coriamyrtin	$C_{15}H_{18}O_5$	[M+Na]+	301.10464	301.10486	-0.72		3.9E+06						
326	Panaxytriol	$C_{17}H_{26}O_3$	[M+Na]+	301.17742	301.17748	-0.21		4.6E+06		5.7E+05		2.9E+06		1.7E+06
327	Tetranor-trihydroxy-prostenoic acid	$C_{16}H_{28}O_5$	[M+H]+	301.20095	301.20098	-0.10		3.5E+06						
328	Methyl-hexadecanedioic acid	$C_{17}H_{32}O_4$	[M+H]+	301.23734	301.23742	-0.28		1.5E+06						
329	Hydroxystearic acid	$C_{18}H_{36}O_3$	[M+H]+	301.27372	301.27379	-0.23		2.0E+06						
330	Nocodazole	$C_{14}H_{11}N_3O_3S$	[M+H]+	302.05939	302.05920	0.62					1.4E+06			
331	N-Acetyl-Glucosamine Phosphate	$C_8H_{16}NO_9P$	[M+H]+	302.06354	302.06372	-0.57			1.6E+06		1.2E+06		1.2E+06	
332	Sphinganine	$C_{18}H_{39}NO_2$	[M+H]+	302.30536	302.30544	-0.28		2.6E+07						
333	Glyceryl tributyrate	$C_{15}H_{26}O_6$	[M+H]+	303.18022	303.18035	-0.45		2.0E+06						
334	Hydroxy-heptadecatrienoic acid	$C_{17}H_{28}O_3$	[M+Na]+	303.19307	303.19312	-0.18				7.7E+05		2.4E+06		1.5E+06
335	Linoleic acid	$C_{18}H_{32}O_2$	[M+Na]+	303.22945	303.22953	-0.25	1.8E+06		2.6E+06	2.6E+06			4.1E+06	2.9E+06
336	Retinyl ester	$C_{20}H_{30}O_2$	[M+H]+	303.23186	303.23192	-0.21		2.1E+06						
337	Hydroxyphenylacetylglutamic acid	$C_{13}H_{15}NO_6$	[M+Na]+	304.07916	304.07937	-0.71	6.7E+06		1.3E+07		5.9E+06		2.5E+06	
338	Vasconine	$C_{17}H_{16}NO_2$	[M+K]+	305.08126	305.08118	0.27					3.4E+06			
339	Randainol	$C_{18}H_{18}O_3$	[M+Na]+	305.11482	305.11500	-0.61		1.3E+06						
340	Dihydrophaseic acid	$C_{15}H_{22}O_5$	[M+Na]+	305.13594	305.13608	-0.44		3.0E+06						
341	Nopaline	$C_{11}H_{20}N_4O_6$	[M+H]+	305.14556	305.14566	-0.32		2.4E+06						
342	Epoxy-trimethyltrideca-dienoic acid	$C_{16}H_{26}O_3$	[M+K]+	305.15135	305.15144	-0.28		2.6E+06						
343	Dehydroretinal	$C_{20}H_{26}O$	[M+Na]+	305.18759	305.18784	-0.83		4.1E+06						
344	Arachidonic acid	$C_{20}H_{32}O_2$	[M+H]+	305.24751	305.24758	-0.24		3.4E+06						

				ESI(+) FT-ICR MS									
**No.**	**Plausible Compound[a]**	**Molecular formula (M)**	**Ion**	**Theor m/z[b]**	**Exp. m/z[c]**	**Δppm[d]**	**SM$_R$H[e]**	**SM$_R$O[e]**	**SM$_P$H[e]**	**SM$_P$O[e]**	**TF$_R$H[e]**	**TF$_R$O[e]**	**TF$_P$H[e]**	**TF$_P$O[e]**
345	Assoanine	$C_{17}H_{17}NO_2$	[M+K]+	306.08909	306.08900	0.29							1.7E+06	
346	Dimethoxyflavanone	$C_{17}H_{16}O_4$	[M+Na]+	307.09408	307.09429	-0.68		2.3E+06						
347	Allogibberic acid	$C_{18}H_{20}O_3$	[M+Na]+	307.13047	307.13070	-0.76		2.7E+06						
348	Methylestra-tetraenediol	$C_{19}H_{24}O_2$	[M+Na]+	307.16685	307.16710	-0.81		1.2E+07						
349	Retinal	$C_{20}H_{28}O$	[M+Na]+	307.20324	307.20341	-0.57						1.7E+06		
350	Stearic acid	$C_{18}H_{36}O_2$	[M+Na]+	307.26075	307.26082	-0.21	8.6E+06		5.7E+06		9.0E+06		8.9E+06	
351	Glutathione	$C_{10}H_{17}N_3O_6S$	[M+H]+	308.09108	308.09114	-0.19	2.1E+06		1.8E+06		1.8E+06		1.7E+06	
352	Hydroxy-dimethoxyflavan	$C_{17}H_{18}O_4$	[M+Na]+	309.10973	309.10997	-0.78		6.8E+06						
353	Hydroxyestrone	$C_{18}H_{22}O_3$	[M+Na]+	309.14612	309.14636	-0.79		4.4E+06						
354	Fructoselysine	$C_{12}H_{24}N_2O_7$	[M+H]+	309.16563	309.16569	-0.19	2.8E+06		3.2E+06		2.4E+06		2.0E+06	
355	Boldenone	$C_{19}H_{26}O_2$	[M+Na]+	309.18250	309.18272	-0.72	2.1E+06	2.8E+07	2.9E+06		1.9E+06		2.3E+06	
356	Trifluoro-tetradecenyl acetate	$C_{16}H_{27}F_3O_2$	[M+H]+	309.20359	309.20370	-0.35		1.3E+07		1.1E+06	1.3E+06	2.4E+06	1.5E+06	1.5E+06
357	Methylembelin	$C_{18}H_{28}O_4$	[M+H]+	309.20604	309.20611	-0.24		2.8E+06						
358	Hydroxy-palmitic acid methyl ester	$C_{17}H_{34}O_3$	[M+Na]+	309.24002	309.24007	-0.18						2.4E+06		1.6E+06
359	Eicosadienoic acid	$C_{20}H_{36}O_2$	[M+H]+	309.27881	309.27891	-0.33		3.9E+06						
360	N-Acetyl-neuraminic acid	$C_{11}H_{19}NO_9$	[M+H]+	310.11326	310.11332	-0.20	2.7E+07	3.0E+06	9.1E+06		1.6E+07		5.1E+06	
361	Galanthamine	$C_{17}H_{21}NO_3$	[M+Na]+	310.14136	310.14160	-0.77	1.8E+06		1.4E+06	2.2E+06				
362	Procyclidine	$C_{19}H_{29}NO$	[M+Na]+	310.21414	310.21438	-0.79		6.6E+06						
363	Prosopinine	$C_{16}H_{33}NO_3$	[M+Na]+	310.23526	310.23531	-0.15								1.7E+06
364	Dihydroxy-methoxy-isoflavanol	$C_{16}H_{16}O_5$	[M+Na]+	311.08899	311.08923	-0.76		4.2E+06						
365	Glutamyltyrosine	$C_{14}H_{18}N_2O_6$	[M+H]+	311.12376	311.12384	-0.25		3.2E+06						
366	Karwinaphthol B	$C_{17}H_{20}O_4$	[M+Na]+	311.12538	311.12565	-0.87		3.1E+06						
367	Ovalitenin B	$C_{19}H_{18}O_4$	[M+H]+	311.12779	311.12788	-0.30				9.4E+05				
368	Hydroxyestradiol	$C_{18}H_{24}O_3$	[M+Na]+	311.16177	311.16200	-0.75		2.7E+06						
369	Dimethyl-(methoxyphenyl)-ethyl-benzopyranol	$C_{20}H_{22}O_3$	[M+H]+	311.16417	311.16428	-0.35		4.9E+06						
370	Botrydial	$C_{17}H_{26}O_5$	[M+H]+	311.18530	311.18536	-0.19	2.1E+06				1.9E+06		1.6E+06	
371	Dihydroxy-palmitic acid	$C_{16}H_{32}O_4$	[M+Na]+	311.21928	311.21937	-0.29		5.4E+06						
372	Methoprene	$C_{19}H_{34}O_3$	[M+H]+	311.25807	311.25813	-0.20	1.7E+06	3.8E+06	1.6E+06			4.1E+06	1.9E+06	1.3E+06
373	Eicosenoic acid	$C_{20}H_{38}O_2$	[M+H]+	311.29446	311.29452	-0.20		5.5E+06				1.6E+06		

				ESI(+) FT-ICR MS									
**No.**	**Plausible Compound[a]**	**Molecular formula (M)**	**Ion**	**Theor m/z[b]**	**Exp. m/z[c]**	**Δppm[d]**	**SM$_R$H[e]**	**SM$_R$O[e]**	**SM$_P$H[e]**	**SM$_P$O[e]**	**TF$_R$H[e]**	**TF$_R$O[e]**	**TF$_P$H[e]**	**TF$_P$O[e]**
374	Dyclonine	$C_{18}H_{27}NO_2$	[M+Na]+	312.19340	312.19362	-0.71			1.5E+06					
375	Methylvisamminol	$C_{16}H_{18}O_5$	[M+Na]+	313.10464	313.10487	-0.72		4.6E+06						
376	epi-Tulipinolide	$C_{17}H_{22}O_4$	[M+Na]+	313.14103	313.14126	-0.73		4.5E+06						
377	Sterculynic acid	$C_{19}H_{30}O_2$	[M+Na]+	313.21380	313.21397	-0.53	1.7E+06		1.8E+06			2.9E+06	1.8E+06	1.2E+06
378	Oxo-nonadecanoic acid	$C_{19}H_{36}O_3$	[M+H]+	313.27372	313.27379	-0.22	4.2E+06	8.0E+06	3.6E+06	1.5E+06	1.4E+06	1.2E+07	3.6E+06	1.8E+06
379	Arachidic acid	$C_{20}H_{40}O_2$	[M+H]+	313.31011	313.31018	-0.22		4.1E+06		7.4E+05		2.1E+06		
380	Heliotrine	$C_{16}H_{27}NO_5$	[M+H]+	314.19620	314.19629	-0.29		4.1E+06						
381	N-palmitoyl glycine	$C_{18}H_{35}NO_3$	[M+H]+	314.26897	314.26906	-0.28		3.3E+06						
382	Margaroyl-ethanolamine	$C_{19}H_{39}NO_2$	[M+H]+	314.30536	314.30544	-0.27		7.3E+06						
383	Amino-dimethyloctadecanol	$C_{20}H_{43}NO$	[M+H]+	314.34174	314.34186	-0.38		2.5E+06						
384	Picrotoxinin	$C_{15}H_{16}O_6$	[M+Na]+	315.08391	315.08414	-0.73		2.2E+06						
385	Sugeonyl acetate	$C_{17}H_{24}O_3$	[M+K]+	315.13570	315.13580	-0.30	1.5E+06							
386	Gingerdione	$C_{17}H_{24}O_4$	[M+Na]+	315.15668	315.15678	-0.32		2.3E+06						1.5E+06
387	Hydroxysteroid	$C_{19}H_{32}O_2$	[M+Na]+	315.22945	315.22954	-0.28		2.5E+06						
388	Octadecanedioic acid	$C_{18}H_{34}O_4$	[M+H]+	315.25299	315.25307	-0.25		4.3E+06				2.2E+06		
389	Hydroxy-nonadecanoic acid	$C_{19}H_{38}O_3$	[M+H]+	315.28937	315.28944	-0.22		2.9E+06						
390	Eicosanediol	$C_{20}H_{42}O_2$	[M+H]+	315.32576	315.32584	-0.25		3.4E+06				1.8E+06		
391	Butoctamide hydrogen succinate	$C_{16}H_{29}NO_5$	[M+H]+	316.21185	316.21194	-0.29		1.8E+08						
392	Decanoylcarnitine	$C_{17}H_{33}NO_4$	[M+H]+	316.24824	316.24833	-0.30		1.7E+06						
393	Dehydrophytosphingosine	$C_{18}H_{37}NO_3$	[M+H]+	316.28462	316.28471	-0.30	1.2E+06	2.3E+07	2.5E+06			2.1E+06	1.6E+06	
394	(Butylbenzofuranyl)(hydroxyphenyl)ketone	$C_{19}H_{18}O_3$	[M+Na]+	317.11482	317.11503	-0.68		2.0E+07		2.0E+06		3.5E+06	4.1E+06	1.6E+06
395	[Ethyl-(methoxyphenyl)-pyrazolyl]phenol	$C_{18}H_{18}N_2O_2$	[M+Na]+	317.12605	317.12582	0.72						1.4E+06		
396	Docosahexaynoic acid	$C_{22}H_{20}O_2$	[M+H]+	317.15361	317.15370	-0.30						3.1E+06		
397	Methylptelefolonium	$C_{18}H_{22}NO_4$	[M+H]+	317.16216	317.16219	-0.08		4.4E+06				5.0E+06		
398	Phytuberin	$C_{17}H_{26}O_4$	[M+Na]+	317.17233	317.17241	-0.25		3.9E+06						
399	Methyl-octadecadienoic acid	$C_{19}H_{34}O_2$	[M+Na]+	317.24510	317.24513	-0.09						2.0E+06		
400	Propyl-androstenol	$C_{22}H_{36}O$	[M+H]+	317.28389	317.28397	-0.25		9.4E+06				1.6E+06		
401	Nilutamide	$C_{12}H_{10}F_3N_3O_4$	[M+H]+	318.06962	318.06987	-0.80	3.5E+06				3.4E+06			
402	(R)-Prunasin	$C_{14}H_{17}NO_6$	[M+Na]+	318.09481	318.09499	-0.57			1.4E+06					

				ESI(+) FT-ICR MS											
**No.**	**Plausible Compound[a]**	**Molecular formula (M)**	**Ion**	**Theor m/z[b]**	**Exp. m/z[c]**	**Δppm[d]**	**SM_RH[e]**	**SM_RO[e]**	**SM_PH[e]**	**SM_PO[e]**	**TF_RH[e]**	**TF_RO[e]**	**TF_PH[e]**	**TF_PO[e]**
403	Phytosphingosine	$C_{18}H_{39}NO_3$	[M+H]+	318.30027	318.30038	-0.34		3.3E+06						
404	Dimethoxy-methylflavone	$C_{18}H_{16}O_4$	[M+Na]+	319.09408	319.09432	-0.75		2.0E+06						
405	Deoxynivalenol	$C_{15}H_{20}O_6$	[M+Na]+	319.11521	319.11539	-0.57		2.9E+06						
406	Tocopheronic acid	$C_{16}H_{24}O_5$	[M+Na]+	319.15159	319.15181	-0.68		2.5E+06				1.5E+06		
407	Diethylstilbestrol dimethyl ether	$C_{20}H_{24}O_2$	[M+Na]+	319.16685	319.16709	-0.75		6.1E+06						
408	Methano-retinal	$C_{21}H_{28}O$	[M+Na]+	319.20324	319.20350	-0.81		7.9E+07				2.4E+06		
409	Keto-octadecenoic acid	$C_{18}H_{32}O_3$	[M+Na]+	319.22437	319.22443	-0.21	1.6E+06		2.2E+06	1.9E+06	1.4E+06		4.0E+06	1.7E+06
410	Phenthoate	$C_{12}H_{17}O_4PS_2$	[M+H]+	321.03786	321.03803	-0.52					1.4E+06			
411	Doisynoestrol	$C_{19}H_{22}O_3$	[M+Na]+	321.14612	321.14636	-0.76		1.9E+06						
412	Tetranor-oxo-dihydroxy-prostenoic acid	$C_{16}H_{26}O_5$	[M+Na]+	321.16724	321.16735	-0.33		4.1E+06						
413	Methyl-nor-pregnatrienol	$C_{21}H_{30}O$	[M+Na]+	321.21889	321.21912	-0.72		1.2E+08				3.5E+06		1.3E+06
414	Keto stearic acid	$C_{18}H_{34}O_3$	[M+Na]+	321.24002	321.24008	-0.20					3.3E+06			
415	Methyl stearic acid	$C_{19}H_{38}O_2$	[M+Na]+	321.27640	321.27640	0.00			1.2E+06	1.6E+06				2.7E+06
416	Phytanol	$C_{20}H_{42}O$	[M+Na]+	321.31279	321.31291	-0.38		1.6E+06						
417	Dihydroxy-methoxy-methylflavanone	$C_{17}H_{16}O_5$	[M+Na]+	323.08899	323.08922	-0.70		2.3E+06						
418	Trimethoxyflavan	$C_{18}H_{20}O_4$	[M+Na]+	323.12538	323.12564	-0.81		4.0E+06						
419	Methoxyestrone	$C_{19}H_{24}O_3$	[M+Na]+	323.16177	323.16202	-0.79		5.3E+06						
420	Retinoic acid	$C_{20}H_{28}O_2$	[M+Na]+	323.19815	323.19838	-0.71		8.9E+06						
421	MG(0:0/14:1/0:0)	$C_{17}H_{32}O_4$	[M+Na]+	323.21928	323.21935	-0.23	2.4E+06		2.7E+06	1.5E+06	2.8E+06	3.4E+06	3.3E+06	1.8E+06
422	Allylestrenol	$C_{21}H_{32}O$	[M+Na]+	323.23454	323.23476	-0.68		4.4E+07		2.7E+06				3.6E+06
423	Hydroxystearic acid	$C_{18}H_{36}O_3$	[M+Na]+	323.25567	323.25575	-0.26				6.2E+05		2.1E+06		
424	Linoleoyl ethanolamide	$C_{20}H_{37}NO_2$	[M+H]+	324.28971	324.28978	-0.23		5.3E+06						
425	N-Glucosylnicotinate	$C_{12}H_{16}NO_7$	[M+K]+	325.05584	325.05554	0.91							4.5E+06	
426	Dihydroxy-dimethoxyflavan	$C_{17}H_{18}O_5$	[M+Na]+	325.10464	325.10488	-0.72		7.8E+06						
427	Fructofuranose dianhydride	$C_{12}H_{20}O_{10}$	[M+H]+	325.11292	325.11299	-0.22	2.4E+06	4.6E+06	4.1E+06		2.0E+06	1.4E+06	6.6E+06	
428	Ketoestriol	$C_{18}H_{22}O_4$	[M+Na]+	325.14103	325.14127	-0.74		3.6E+06						
429	Allethrin	$C_{19}H_{26}O_3$	[M+Na]+	325.17742	325.17765	-0.72		1.2E+07						
430	Hydroxy-hexadecandioic acid	$C_{16}H_{30}O_5$	[M+Na]+	325.19854	325.19860	-0.18		1.7E+07	1.4E+06	1.9E+06		3.7E+06		1.3E+06
431	3-Hydroxy-palmitic acid methyl ester	$C_{17}H_{34}O_3$	[M+K]+	325.21395	325.21390	0.17						1.9E+06		

				ESI(+) FT-ICR MS									
**No.**	**Plausible Compound**[a]	**Molecular formula (M)**	**Ion**	**Theor m/z**[b]	**Exp. m/z**[c]	**Δppm**[d]	**SM$_{R}$H**[e]	**SM$_{R}$O**[e]	**SM$_{P}$H**[e]	**SM$_{P}$O**[e]	**TF$_{R}$H**[e]	**TF$_{R}$O**[e]	**TF$_{P}$H**[e]	**TF$_{P}$O**[e]
432	MG(14:0/0:0/0:0)	$C_{17}H_{34}O_4$	[M+Na]+	325.23493	325.23499	-0.19	2.0E+06			1.3E+06	2.0E+06	4.3E+06		2.8E+06
433	Methyl-eicosenoic acid	$C_{21}H_{40}O_2$	[M+H]+	325.31011	325.31017	-0.19		1.6E+06						
434	Nitrolinoleic acid	$C_{18}H_{31}NO_4$	[M+H]+	326.23258	326.23266	-0.23		3.1E+06						
435	N-oleoyl ethanolamine	$C_{20}H_{39}NO_2$	[M+H]+	326.30536	326.30545	-0.29		1.1E+07						
436	Dihydroxy-prenyloxydihydrochalcone	$C_{20}H_{22}O_4$	[M+H]+	327.15909	327.15913	-0.14		1.3E+06						
437	Hydroxytestosterone	$C_{19}H_{28}O_3$	[M+Na]+	327.19307	327.19329	-0.69		4.8E+06						
438	Isoacitretin	$C_{21}H_{26}O_3$	[M+H]+	327.19547	327.19559	-0.35		2.9E+06				1.7E+06		
439	Arachidonic acid	$C_{20}H_{32}O_2$	[M+Na]+	327.22945	327.22954	-0.27						2.1E+06		
440	Methoxy-hydroxy-octadecadienoic acid	$C_{19}H_{34}O_4$	[M+H]+	327.25299	327.25312	-0.41		1.6E+06						
441	Oxo-eicosanoic acid	$C_{20}H_{38}O_3$	[M+H]+	327.28937	327.28945	-0.25	1.6E+06	3.4E+06	1.5E+06			3.2E+06	1.4E+06	
442	Methyl-eicosanoic acid	$C_{21}H_{42}O_2$	[M+H]+	327.32576	327.32585	-0.28		1.8E+06						
443	Carboxy-chromano	$C_{18}H_{25}O_4$	[M+Na]+	328.16450	328.16430	0.63	3.3E+06		2.6E+06		2.3E+06		3.3E+06	
444	Nitro-octadecenoic acid	$C_{18}H_{33}NO_4$	[M+H]+	328.24824	328.24834	-0.32		2.4E+06						
445	N-palmitoyl alanine	$C_{19}H_{37}NO_3$	[M+H]+	328.28462	328.28475	-0.39		1.8E+06						
446	Dimethylsphingosine	$C_{20}H_{41}NO_2$	[M+H]+	328.32101	328.32109	-0.26		1.4E+07						
447	Confertiflorin	$C_{17}H_{22}O_5$	[M+Na]+	329.13594	329.13618	-0.72		2.8E+06						
448	Dehydroreticuline	$C_{19}H_{22}NO_4$	[M+H]+	329.16216	329.16223	-0.21						1.7E+06		
449	Hydroxyandrosterone	$C_{19}H_{30}O_3$	[M+Na]+	329.20872	329.20885	-0.41		4.0E+06						
450	Icosatrienoic acid	$C_{20}H_{34}O_2$	[M+Na]+	329.24510	329.24518	-0.24		1.3E+07				2.0E+06		
451	MG(16:1/0:0/0:0)	$C_{19}H_{36}O_4$	[M+H]+	329.26864	329.26871	-0.23		8.4E+06		9.1E+05		4.9E+06		
452	Narciclasine	$C_{14}H_{13}NO_7$	[M+Na]+	330.05842	330.05864	-0.67	1.2E+07		7.9E+06		6.1E+06		3.4E+06	
453	Europine	$C_{16}H_{27}NO_6$	[M+H]+	330.19111	330.19124	-0.38		2.7E+06						
454	Keto-decanoylcarnitine	$C_{17}H_{31}NO_5$	[M+H]+	330.22750	330.22755	-0.15		6.8E+06						
455	Dimethylnonanoyl carnitine	$C_{18}H_{35}NO_4$	[M+H]+	330.26389	330.26398	-0.27		6.2E+06				2.3E+06		
456	Palmitoyl Serinol	$C_{19}H_{39}NO_3$	[M+H]+	330.30027	330.30036	-0.27		3.8E+06				1.7E+06		
457	N,N-dimethyl-Safingol	$C_{20}H_{43}NO_2$	[M+H]+	330.33666	330.33674	-0.25		1.3E+07		7.1E+05		4.0E+06		
458	Dihydroxy-(dimethyl-propenyl)chalcone	$C_{20}H_{20}O_3$	[M+Na]+	331.13047	331.13069	-0.68		2.0E+06				3.2E+06		
459	Gambirtannine	$C_{21}H_{18}N_2O_2$	[M+H]+	331.14410	331.14379	0.95			1.5E+06					
460	Gestrinone	$C_{21}H_{24}O_2$	[M+Na]+	331.16685	331.16706	-0.62		4.1E+06				1.7E+06		

No.	Plausible Compound[a]	Molecular formula (M)	Ion	Theor m/z[b]	Exp. m/z[c]	Δppm[d]	SM_RH[e]	SM_RO[e]	SM_PH[e]	SM_PO[e]	TF_RH[e]	TF_RO[e]	TF_PH[e]	TF_PO[e]
				ESI(+) FT-ICR MS										
461	Tetraphyllicine	$C_{20}H_{24}N_2O$	[M+Na]+	331.17808	331.17783	0.77		5.0E+06				7.2E+06		
462	Methylembelin	$C_{18}H_{28}O_4$	[M+Na]+	331.18798	331.18803	-0.14						2.4E+06		1.7E+06
463	Methyl-epoxy-octadecadienoate	$C_{19}H_{32}O_3$	[M+Na]+	331.22437	331.22448	-0.33		2.7E+06				2.0E+06		
464	Eicosadienoic acid	$C_{20}H_{36}O_2$	[M+Na]+	331.26075	331.26082	-0.22				2.6E+06		2.5E+07		1.5E+06
465	Hydroxy-pentanorvitamin D3	$C_{22}H_{34}O_2$	[M+H]+	331.26316	331.26324	-0.24		5.4E+06				3.5E+06		
466	MG(16:0/0:0/0:0)	$C_{19}H_{38}O_4$	[M+H]+	331.28429	331.28437	-0.25		7.8E+06		9.6E+05		8.1E+06	1.5E+06	
467	Geranylgeranylacetone	$C_{23}H_{38}O$	[M+H]+	331.29954	331.29959	-0.14						2.0E+06		
468	Melochinone	$C_{22}H_{21}NO_2$	[M+H]+	332.16451	332.16449	0.05						1.4E+06		
469	Pentahydroxy-methoxyflavone	$C_{16}H_{12}O_8$	[M+H]+	333.06049	333.06062	-0.38	2.3E+06				2.7E+06			
470	Cinnamoyl glucoside	$C_{15}H_{18}O_7$	[M+Na]+	333.09447	333.09470	-0.68						1.5E+06		
471	Sanguinarine	$C_{20}H_{14}NO_4$	[M+H]+	333.09956	333.09964	-0.24			1.9E+06					
472	Dihydrocordoin	$C_{20}H_{22}O_3$	[M+Na]+	333.14612	333.14624	-0.37				6.2E+05		2.8E+06	1.2E+06	
473	Dihydro-oxo-phytoenoic acid	$C_{18}H_{30}O_3$	[M+K]+	333.18265	333.18272	-0.20		6.3E+06				2.6E+06		
474	apo-Carotenal	$C_{22}H_{30}O$	[M+Na]+	333.21889	333.21912	-0.70		5.7E+06				7.5E+06		
475	Eicosenoic acid	$C_{20}H_{38}O_2$	[M+Na]+	333.27640	333.27650	-0.28				2.7E+06			2.8E+06	
476	Docosatetraenoic acid	$C_{22}H_{36}O_2$	[M+H]+	333.27881	333.27887	-0.20		4.9E+06		9.3E+05		4.1E+06		
477	Xylopine	$C_{18}H_{17}NO_3$	[M+K]+	334.08400	334.08372	0.85					1.4E+06			
478	Hydroxy-methoxyestratriene-carbonitrile	$C_{20}H_{25}NO_2$	[M+Na]+	334.17775	334.17803	-0.84		2.0E+07						
479	Hydroxycinnamyl alcohol glucoside	$C_{15}H_{20}O_7$	[M+Na]+	335.11012	335.11032	-0.59		3.0E+06				2.2E+06		
480	Prenyloxyresveratrol	$C_{19}H_{20}O_4$	[M+Na]+	335.12538	335.12550	-0.34		2.8E+06				1.9E+06		
481	Phenylalanylphenylalanine	$C_{18}H_{20}N_2O_3$	[M+Na]+	335.13661	335.13636	0.76						1.6E+06		
482	Triphenyl-hexene	$C_{24}H_{24}$	[M+Na]+	335.17702	335.17710	-0.22				1.4E+06		1.8E+06		
483	Pregna-dienedione	$C_{21}H_{28}O_2$	[M+Na]+	335.19815	335.19838	-0.68		1.7E+07	1.7E+06	8.5E+05		9.9E+06	1.9E+06	
484	Dihydroxyoctadecadienoic acid	$C_{18}H_{32}O_4$	[M+Na]+	335.21928	335.21935	-0.21				1.2E+06		7.2E+06	1.5E+06	
485	Phytol	$C_{20}H_{40}O$	[M+K]+	335.27108	335.27113	-0.16						1.5E+06		
486	Arachidic acid	$C_{20}H_{40}O_2$	[M+Na]+	335.29205	335.29211	-0.18								2.6E+06
487	Dimethyl-eicosatrienoic acid	$C_{22}H_{38}O_2$	[M+H]+	335.29446	335.29452	-0.19						1.9E+06		
488	N-palmitoyl glycine	$C_{18}H_{35}NO_3$	[M+Na]+	336.25091	336.25097	-0.16						1.8E+06		
489	Trimethoxyflavanone	$C_{18}H_{18}O_5$	[M+Na]+	337.10464	337.10483	-0.55		3.2E+06				1.6E+06		

				ESI(+) FT-ICR MS										
**No.**	**Plausible Compound**[a]	**Molecular formula (M)**	**Ion**	**Theor m/z**[b]	**Exp. m/z**[c]	**Δppm**[d]	SM_RH^e	SM_RO^e	SM_PH^e	SM_PO^e	TF_RH^e	TF_RO^e	TF_PH^e	TF_PO^e
490	Dehydro-gibberellin A9	$C_{19}H_{22}O_4$	[M+Na]+	337.14103	337.14126	-0.68		2.4E+06				1.5E+06		
491	oxo-Retinoic acid	$C_{20}H_{26}O_3$	[M+Na]+	337.17742	337.17763	-0.62		3.0E+06				1.7E+06		
492	Methyl-retro-retinoic acid	$C_{21}H_{30}O_2$	[M+Na]+	337.21380	337.21402	-0.65	2.8E+06	2.9E+07	3.2E+06	1.5E+06	2.4E+06	1.5E+07	2.2E+06	
493	Dihydroxyoctadecenoic acid	$C_{18}H_{34}O_4$	[M+Na]+	337.23493	337.23500	-0.21	1.7E+06		2.0E+06	2.0E+06	1.5E+06		2.8E+06	1.8E+06
494	Hydroxy-nonadecanoic acid	$C_{19}H_{38}O_3$	[M+Na]+	337.27132	337.27141	-0.28				6.0E+05		2.9E+06		
495	Eicosanediol	$C_{20}H_{42}O_2$	[M+Na]+	337.30770	337.30773	-0.09								1.7E+06
496	Eicosadienyl acetate	$C_{22}H_{40}O_2$	[M+H]+	337.31011	337.31016	-0.16						1.8E+06		
497	Rosinidin	$C_{17}H_{15}O_6$	[M+Na]+	338.07608	338.07592	0.48			1.5E+06					
498	Ficine	$C_{20}H_{19}NO_4$	[M+H]+	338.13868	338.13865	0.10						1.8E+06		
499	Pentadecanoylglycine	$C_{17}H_{33}NO_3$	[M+K]+	338.20920	338.20922	-0.05						1.6E+06		
500	Dehydrophytosphingosine	$C_{18}H_{37}NO_3$	[M+Na]+	338.26656	338.26666	-0.28				8.5E+05				
501	Hydroxy-trimethoxyflavan	$C_{18}H_{20}O_5$	[M+Na]+	339.12029	339.12051	-0.64						2.1E+06		
502	Gibberellin A9	$C_{19}H_{24}O_4$	[M+Na]+	339.15668	339.15692	-0.69		5.8E+06				3.4E+06		
503	Dihydroxy-methylandrostadienone	$C_{20}H_{28}O_3$	[M+Na]+	339.19307	339.19328	-0.63		8.1E+06	1.5E+06	8.7E+05	1.6E+06	8.3E+06		
504	Hydroxypregnenone	$C_{21}H_{32}O_2$	[M+Na]+	339.22945	339.22966	-0.62		5.5E+06				2.9E+06		
505	Dihydroxy stearic acid	$C_{18}H_{36}O_4$	[M+Na]+	339.25058	339.25064	-0.19	1.8E+06		2.8E+06	2.5E+06	1.6E+06		5.4E+06	1.8E+06
506	Propylandrostenol	$C_{22}H_{36}O$	[M+Na]+	339.26584	339.26591	-0.23		1.1E+07		3.8E+06		9.4E+06		
507	Hexadecyl-sn-glycerol	$C_{19}H_{40}O_3$	[M+Na]+	339.28697	339.28705	-0.25		2.7E+06				3.0E+06		
508	Docosenoic acid	$C_{22}H_{42}O_2$	[M+H]+	339.32576	339.32581	-0.16		2.2E+06				2.9E+06		
509	S-Sulfanylglutathione	$C_{10}H_{17}N_3O_6S_2$	[M+H]+	340.06315	340.06328	-0.36	1.6E+06		1.9E+06		2.0E+06		1.6E+06	
510	N-oleoyl glycine	$C_{20}H_{37}NO_3$	[M+H]+	340.28462	340.28467	-0.15		3.6E+06				1.6E+06		
511	Docosanamide	$C_{22}H_{45}NO$	[M+H]+	340.35739	340.35746	-0.19		9.9E+06		3.4E+06		6.5E+06		
512	Trihydroxy-dimethoxyisoflavan	$C_{17}H_{18}O_6$	[M+Na]+	341.09956	341.09981	-0.72		2.5E+06				1.6E+06		
513	Zearalenone	$C_{18}H_{22}O_5$	[M+Na]+	341.13594	341.13618	-0.69		6.5E+06				3.1E+06		
514	Ubiquinone Q2	$C_{19}H_{26}O_4$	[M+Na]+	341.17233	341.17242	-0.28	2.3E+06	2.3E+07	1.8E+06			1.4E+07	2.1E+06	
515	Menthyl O-beta-D-glucoside	$C_{16}H_{30}O_6$	[M+Na]+	341.19346	341.19354	-0.24		4.2E+06						
516	Methantheline	$C_{21}H_{26}NO_3$	[M+H]+	341.19855	341.19858	-0.11	1.9E+06				2.1E+06		1.5E+06	4.4E+06
517	Hydroxy-sacculatadienedial	$C_{20}H_{30}O_3$	[M+Na]+	341.20872	341.20892	-0.60		1.3E+07		1.3E+06		9.0E+06		
518	Canrenone	$C_{22}H_{28}O_3$	[M+H]+	341.21112	341.21120	-0.23		3.8E+06				2.3E+06		

				ESI(+) FT-ICR MS										
**No.**	**Plausible Compound**[a]	**Molecular formula (M)**	**Ion**	**Theor m/z**[b]	**Exp. m/z**[c]	**Δppm**[d]	**SM$_R$H**[e]	**SM$_R$O**[e]	**SM$_P$H**[e]	**SM$_P$O**[e]	**TF$_R$H**[e]	**TF$_R$O**[e]	**TF$_P$H**[e]	**TF$_P$O**[e]
519	Pregnanolone	$C_{21}H_{34}O_2$	[M+Na]+	341.24510	341.24527	-0.48				1.3E+06		5.1E+06		
520	Oxo-heneicosanoic acid	$C_{21}H_{40}O_3$	[M+H]+	341.30502	341.30509	-0.20		2.8E+06				3.7E+06		
521	Methyl-heneicosanoic acid	$C_{22}H_{44}O_2$	[M+H]+	341.34141	341.34147	-0.17		3.8E+06		1.3E+06		6.0E+06		
522	Lactosamine	$C_{12}H_{23}NO_{10}$	[M+H]+	342.13947	342.13954	-0.20		1.8E+06						
523	Carboxy-chromanol	$C_{19}H_{27}O_4$	[M+Na]+	342.18016	342.17996	0.57	3.8E+06		2.4E+06		3.8E+06		4.0E+06	
524	Acetyllycopsamine	$C_{17}H_{27}NO_6$	[M+H]+	342.19111	342.19116	-0.13		3.0E+06						
525	Dodecenoylcarnitine	$C_{19}H_{35}NO_4$	[M+H]+	342.26389	342.26395	-0.18		2.4E+06				1.9E+06		
526	N-stearoyl glycine	$C_{20}H_{39}NO_3$	[M+H]+	342.30027	342.30033	-0.17		2.0E+06				1.7E+06		
527	Arctolide	$C_{17}H_{20}O_6$	[M+Na]+	343.11521	343.11542	-0.60		4.1E+06				2.4E+06		
528	Lactose	$C_{12}H_{22}O_{11}$	[M+H]+	343.12349	343.12355	-0.17	2.2E+06	3.8E+06	2.3E+06			1.7E+06	4.6E+06	
529	alpha-Zearalenol	$C_{18}H_{24}O_5$	[M+Na]+	343.15159	343.15183	-0.69		2.4E+06						
530	Phaseollidin hydrate	$C_{20}H_{22}O_5$	[M+H]+	343.15400	343.15406	-0.17		1.6E+06						
531	Hydroxyarachidonic acid	$C_{20}H_{32}O_3$	[M+Na]+	343.22437	343.22447	-0.30		3.0E+06				2.1E+06		
532	Dimethyl-androstanediol	$C_{21}H_{36}O_2$	[M+Na]+	343.26075	343.26084	-0.26		3.4E+06				2.5E+06		
533	Tetrahydroxy-prostaene	$C_{20}H_{38}O_4$	[M+H]+	343.28429	343.28432	-0.10		2.5E+06				4.5E+06		
534	MG(P-18:0e/0:0/0:0)	$C_{21}H_{42}O_3$	[M+H]+	343.32067	343.32071	-0.11						2.2E+06		
535	N-palmitoyl serine	$C_{19}H_{37}NO_4$	[M+H]+	344.27954	344.27959	-0.16		3.9E+06				2.8E+06		
536	epi-Tulipinolide diepoxide	$C_{17}H_{22}O_6$	[M+Na]+	345.13086	345.13106	-0.57		3.4E+06				2.1E+06		
537	Quinidinone	$C_{20}H_{22}N_2O_2$	[M+Na]+	345.15735	345.15711	0.69	1.3E+06	4.7E+06	1.7E+06	7.2E+05		7.2E+06	1.7E+06	1.5E+06
538	Tetracosahexaynoic acid	$C_{24}H_{24}O_2$	[M+H]+	345.18491	345.18499	-0.24						2.2E+06		
539	Gingerol	$C_{19}H_{30}O_4$	[M+Na]+	345.20363	345.20369	-0.17						1.4E+06		
540	Lamtidine	$C_{18}H_{28}N_6O$	[M+H]+	345.23974	345.24005	-0.89		5.7E+06				3.1E+06		
541	Heneicosadienoic acid	$C_{21}H_{38}O_2$	[M+Na]+	345.27640	345.27647	-0.18		2.5E+06				2.2E+06		
542	Dihydroxy arachidic acid	$C_{20}H_{40}O_4$	[M+H]+	345.29994	345.29994	-0.01		3.5E+06				2.6E+06		
543	Portulacaxanthin I	$C_{14}H_{16}N_2O_7$	[M+Na]+	347.08497	347.08519	-0.62	1.2E+07		2.1E+07		1.1E+07		2.9E+06	
544	Oxo-dihydroxy-octadecatrienoic acid	$C_{18}H_{28}O_5$	[M+Na]+	347.18289	347.18309	-0.55		3.3E+06				2.5E+06		
545	Docosatetraynoic acid	$C_{22}H_{28}O_2$	[M+Na]+	347.19815	347.19839	-0.69		2.5E+06						
546	Methyl hydroperoxy-octadecatrienoate	$C_{19}H_{32}O_4$	[M+Na]+	347.21928	347.21933	-0.14						1.7E+06		
547	Propano-retinal	$C_{23}H_{32}O$	[M+Na]+	347.23454	347.23475	-0.62		2.1E+07		6.3E+05		1.2E+07		

				ESI(+) FT-ICR MS											
**No.**	**Plausible Compound**[a]	**Molecular formula (M)**	**Ion**	**Theor m/z**[b]	**Exp. m/z**[c]	**Δppm**[d]	**SM$_R$H**[e]	**SM$_R$O**[e]	**SM$_P$H**[e]	**SM$_P$O**[e]	**TF$_R$H**[e]	**TF$_R$O**[e]	**TF$_P$H**[e]	**TF$_P$O**[e]
548	Heneicosenoic acid	$C_{21}H_{40}O_2$	[M+Na]+	347.29205	347.29212	-0.20				7.6E+05				
549	Pancratistatin	$C_{14}H_{15}NO_8$	[M+Na]+	348.06899	348.06918	-0.56	1.1E+07		6.1E+06		7.9E+06		1.7E+06	
550	Deoxyguanosine monophosphate	$C_{10}H_{14}N_5O_7P$	[M+H]+	348.07036	348.07041	-0.14	8.5E+06		4.2E+06		5.7E+06	1.6E+06	3.6E+06	
551	O-beta-D-Glucosyl-hydroxycinnamate	$C_{15}H_{18}O_8$	[M+Na]+	349.08939	349.08946	-0.21			1.6E+06		1.7E+06			
552	Trihydroxy-prenyldihydrochalcone	$C_{20}H_{22}O_4$	[M+Na]+	349.14103	349.14123	-0.57	4.0E+06		3.1E+06	1.7E+06	3.3E+06	1.6E+07	2.7E+06	
553	Caribine	$C_{19}H_{22}N_2O_3$	[M+Na]+	349.15226	349.15200	0.75						2.2E+06		
554	Dinor-iso prostaglandin F2	$C_{18}H_{30}O_5$	[M+Na]+	349.19854	349.19864	-0.26		3.7E+06				2.0E+06		
555	Methyl-eicosanoic acid	$C_{21}H_{42}O_2$	[M+Na]+	349.30770	349.30776	-0.15				1.7E+06		8.7E+06		
556	Biperiden	$C_{21}H_{29}NO$	[M+K]+	350.18807	350.18775	0.93								1.6E+06
557	Dihomo-gamma-Linolenoyl ethanolamide	$C_{22}H_{39}NO_2$	[M+H]+	350.30536	350.30539	-0.10		3.9E+06				2.8E+06		
558	Succinyl-enolpyruvyl-hydroxy-cyclohexene-carboxylate	$C_{14}H_{16}O_9$	[M+Na]+	351.06865	351.06886	-0.59			1.5E+06		1.6E+06		2.3E+06	
559	Hydroxy-dioxotetranorprostanedioic acid	$C_{16}H_{24}O_7$	[M+Na]+	351.14142	351.14163	-0.59		2.1E+06						
560	Triphenyl-hexene	$C_{24}H_{24}$	[M+K]+	351.15096	351.15084	0.34								1.8E+06
561	Crocetin	$C_{20}H_{24}O_4$	[M+Na]+	351.15668	351.15688	-0.57		3.1E+06				2.1E+06		
562	Dihydroxypregnatrienone	$C_{21}H_{28}O_3$	[M+Na]+	351.19307	351.19328	-0.60		9.7E+06				4.1E+06		
563	Hydroperoxy-epoxy-octadecenoic acid	$C_{18}H_{32}O_5$	[M+Na]+	351.21419	351.21427	-0.21		7.5E+06		1.3E+06				
564	Retinol Acetate	$C_{22}H_{32}O_2$	[M+Na]+	351.22945	351.22963	-0.50		4.1E+06				3.1E+06		
565	MG(16:1/0:0/0:0)	$C_{19}H_{36}O_4$	[M+Na]+	351.25058	351.25065	-0.19	7.1E+06		8.1E+06		5.8E+06		1.2E+07	4.0E+06
566	Hydroxy-eicosanoic acid	$C_{20}H_{40}O_3$	[M+Na]+	351.28697	351.28700	-0.10						2.7E+06		
567	Ochotensine	$C_{21}H_{21}NO_4$	[M+H]+	352.15433	352.15431	0.07				1.1E+06		3.0E+06		
568	Anandamide (20:2, n-6)	$C_{22}H_{41}NO_2$	[M+H]+	352.32101	352.32106	-0.16		8.8E+06		9.8E+05		3.9E+06		
569	Gibberellin A5	$C_{19}H_{22}O_5$	[M+Na]+	353.13594	353.13615	-0.57		2.7E+06				1.5E+06		
570	Epiiridodial glucoside	$C_{16}H_{26}O_7$	[M+Na]+	353.15707	353.15717	-0.27						1.9E+06		
571	Dicyclohexyl phthalate	$C_{20}H_{26}O_4$	[M+Na]+	353.17233	353.17256	-0.64		3.1E+06				2.3E+06		
572	Octadecanedioic acid	$C_{18}H_{34}O_4$	[M+K]+	353.20887	353.20893	-0.17		1.4E+07		6.9E+05		7.4E+06		
573	Trihydroxy-octadecenoic acid	$C_{18}H_{34}O_5$	[M+Na]+	353.22984	353.22990	-0.17	2.0E+06	1.7E+07		3.4E+06		1.7E+07	2.2E+06	
574	MG(16:0/0:0/0:0)	$C_{19}H_{38}O_4$	[M+Na]+	353.26623	353.26629	-0.17	1.3E+07		1.7E+07		1.5E+07			5.4E+06
575	Eicosanediol	$C_{20}H_{42}O_2$	[M+K]+	353.28164	353.28170	-0.17				1.0E+06				

No.	Plausible Compound[a]	Molecular formula (M)	Ion	Theor m/z[b]	Exp. m/z[c]	Δppm[d]	$SM_{R}H$[e]	$SM_{R}O$[e]	$SM_{P}H$[e]	$SM_{P}O$[e]	$TF_{R}H$[e]	$TF_{R}O$[e]	$TF_{P}H$[e]	$TF_{P}O$[e]
				ESI(+) FT-ICR MS										
576	Tetracosanal	$C_{24}H_{48}O$	[M+H]+	353.37779	353.37779	0.01						1.8E+06		
577	C16 Sphinganine-phosphate	$C_{16}H_{36}NO_5P$	[M+H]+	354.24039	354.24055	-0.46		4.8E+06				5.9E+06		
578	N-oleoyl alanine	$C_{21}H_{39}NO_3$	[M+H]+	354.30027	354.30035	-0.21		7.8E+06				1.9E+06		
579	Anandamide (20:l, n-9)	$C_{22}H_{43}NO_2$	[M+H]+	354.33666	354.33670	-0.12		1.0E+07				3.3E+06		
580	Dihydroxy-trimethoxydihydrochalcone	$C_{18}H_{20}O_6$	[M+Na]+	355.11521	355.11542	-0.59		2.5E+06				1.6E+06		
581	Dehydro-fluoro-oxotestololactone	$C_{19}H_{21}FO_4$	[M+Na]+	355.13161	355.13165	-0.13		3.5E+06		8.9E+05		3.3E+06		
582	Gibberellin A20	$C_{19}H_{24}O_5$	[M+Na]+	355.15159	355.15182	-0.62		9.9E+06				7.1E+06		
583	Hydroxytridecane-tricarboxylate	$C_{16}H_{28}O_7$	[M+Na]+	355.17272	355.17277	-0.13						1.8E+06		
584	Hydroxyprogesterone	$C_{20}H_{28}O_4$	[M+Na]+	355.18798	355.18818	-0.57		8.7E+06		8.2E+05		5.7E+06		
585	Dihydroxy stearic acid	$C_{18}H_{36}O_4$	[M+K]+	355.22452	355.22455	-0.09	1.5E+06	1.0E+07		1.0E+06		9.9E+06	2.2E+06	
586	Etretinate	$C_{23}H_{30}O_3$	[M+H]+	355.22677	355.22686	-0.25		8.2E+06		3.5E+06		5.5E+06	2.5E+06	4.1E+06
587	Hydroxy-dihydroxy-stearic acid	$C_{18}H_{36}O_5$	[M+Na]+	355.24549	355.24556	-0.19		2.8E+06		7.4E+05		2.2E+06		
588	MG(18:2/0:0/0:0)	$C_{21}H_{38}O_4$	[M+H]+	355.28429	355.28435	-0.17		2.1E+06				2.2E+06		
589	Oxo-docosanoic acid	$C_{22}H_{42}O_3$	[M+H]+	355.32067	355.32074	-0.20		2.4E+06		8.9E+05		2.7E+06		
590	Methyl-docosanoic acid	$C_{23}H_{46}O_2$	[M+H]+	355.35706	355.35713	-0.19				7.3E+05		3.8E+06		
591	N-palmitoyl valine	$C_{21}H_{41}NO_3$	[M+H]+	356.31592	356.31599	-0.19						2.0E+06		
592	Eicosanoyl-ethanolamine	$C_{22}H_{45}NO_2$	[M+H]+	356.35231	356.35238	-0.19		6.1E+06				2.9E+06		
593	Melanin	$C_{18}H_{10}N_2O_4$	[M+K]+	357.02722	357.02713	0.24					1.9E+06			
594	Glutathione episulfonium ion	$C_{12}H_{20}N_3O_6S$	[M+Na]+	357.09650	357.09633	0.50	3.8E+06		2.4E+06		3.9E+06		3.9E+06	
595	Vinorine	$C_{21}H_{22}N_2O_2$	[M+Na]+	357.15735	357.15707	0.78						1.0E+07		
596	Fluoro-dihydroxy-methylandrosta-dienone	$C_{20}H_{27}FO_3$	[M+Na]+	357.18364	357.18357	0.21		2.5E+06						
597	Asterogenol	$C_{21}H_{34}O_3$	[M+Na]+	357.24002	357.24024	-0.62	1.7E+06	1.8E+07		1.4E+06		1.4E+07	1.7E+06	
598	Docosatrienoic acid	$C_{22}H_{38}O_2$	[M+Na]+	357.27640	357.27648	-0.22				7.4E+05				
599	MG(0:0/18:1/0:0)	$C_{21}H_{40}O_4$	[M+H]+	357.29994	357.30001	-0.19		3.8E+06				4.5E+06		
600	N-palmitoyl threonine	$C_{20}H_{39}NO_4$	[M+H]+	358.29519	358.29526	-0.21		8.0E+06				3.5E+06	1.5E+06	
601	S-Nitrosoglutathione	$C_{10}H_{16}N_4O_7S$	[M+Na]+	359.06319	359.06299	0.57	3.2E+06				2.9E+06			
602	Catharanthine	$C_{21}H_{24}N_2O_2$	[M+Na]+	359.17300	359.17273	0.75	6.2E+06	1.4E+07	6.5E+06	2.8E+06	4.8E+06	2.1E+07	6.5E+06	1.1E+07
603	Dihydroxy-eicosatetraenoic acid	$C_{20}H_{32}O_4$	[M+Na]+	359.21928	359.21932	-0.11						1.7E+06		
604	Docosadienoic acid	$C_{22}H_{40}O_2$	[M+Na]+	359.29205	359.29212	-0.19		6.7E+06						

				ESI(+) FT-ICR MS											
**No.**	**Plausible Compound**[a]	**Molecular formula (M)**	**Ion**	**Theor m/z**[b]	**Exp. m/z**[c]	**Δppm**[d]	**SM_RH**[e]	**SM_RO**[e]	**SM_PH**[e]	**SM_PO**[e]	**TF_RH**[e]	**TF_RO**[e]	**TF_PH**[e]	**TF_PO**[e]
605	MG(0:0/18:0/0:0)	$C_{21}H_{42}O_4$	[M+H]+	359.31559	359.31564	-0.15		2.2E+06				4.0E+06		
606	Benzyl viologen	$C_{24}H_{22}N_2$	[M+Na]+	361.16752	361.16723	0.80		1.0E+07				1.1E+07		
607	Dimethoxy-prenylflavan	$C_{22}H_{26}O_3$	[M+Na]+	361.17742	361.17763	-0.59		2.5E+06						
608	Tetrahydroxyandrostanone	$C_{19}H_{30}O_5$	[M+Na]+	361.19854	361.19864	-0.26						2.1E+06		
609	Dihydroxy-eicosatrienoic acid	$C_{20}H_{34}O_4$	[M+Na]+	361.23493	361.23498	-0.12		3.0E+06				2.9E+06		
610	Cyclopentadienylidene-androstanol	$C_{24}H_{34}O$	[M+Na]+	361.25019	361.25036	-0.48		2.4E+06				1.7E+06		
611	Clofilium	$C_{21}H_{37}ClN$	[M+Na]+	361.25067	361.25041	0.73		2.4E+06						
612	Docosenoic acid	$C_{22}H_{42}O_2$	[M+Na]+	361.30770	361.30775	-0.13				1.1E+06				
613	Docosanamide	$C_{22}H_{45}NO$	[M+Na]+	362.33934	362.33937	-0.09								1.4E+06
614	Uridine monophosphate	$C_9H_{13}N_2O_9P$	[M+K]+	362.99903	362.99908	-0.15	1.8E+06							
615	Neobanol	$C_{18}H_{12}O_6$	[M+Na]+	363.02655	363.02645	0.28	4.9E+06		2.1E+06		2.9E+06		2.1E+06	
616	Chelirubine	$C_{21}H_{16}NO_5$	[M+H]+	363.11012	363.11045	-0.90	1.2E+07		1.7E+07		1.4E+07		8.1E+06	
617	Dihydroxy-methoxy-prenyldihydrochalcone	$C_{21}H_{24}O_4$	[M+Na]+	363.15668	363.15688	-0.55		1.7E+06						
618	Hydroxyquinine	$C_{20}H_{24}N_2O_3$	[M+Na]+	363.16791	363.16765	0.74		4.5E+06				5.1E+06	1.5E+06	1.8E+06
619	Epoxy-fluoro-hydroxypregnenedione	$C_{21}H_{27}FO_4$	[M+H]+	363.19661	363.19649	0.34				2.4E+06		3.2E+06		2.4E+06
620	Hydroxy-eicosadienoic acid	$C_{20}H_{36}O_3$	[M+K]+	363.22960	363.22964	-0.08		1.8E+06				1.5E+06		
621	Dihydroxy-prostaenoic acid	$C_{20}H_{36}O_4$	[M+Na]+	363.25058	363.25061	-0.08				9.0E+05				
622	Hexadecenoylcholine	$C_{21}H_{42}NO_2$	[M+Na]+	363.31078	363.31057	0.57		5.3E+06	1.4E+06			6.7E+06	2.0E+06	
623	Methyl-heneicosanoic acid	$C_{22}H_{44}O_2$	[M+Na]+	363.32335	363.32337	-0.05								2.1E+06
624	Dioncophylline C	$C_{23}H_{25}NO_3$	[M+H]+	364.19072	364.19074	-0.04		2.8E+06				2.6E+06		
625	Hydroxy-dimethoxy-methylenedioxyflavone	$C_{18}H_{14}O_7$	[M+Na]+	365.06317	365.06338	-0.57	3.3E+06		3.7E+06		3.0E+06		1.9E+06	
626	Lactose	$C_{12}H_{22}O_{11}$	[M+Na]+	365.10543	365.10549	-0.14				2.2E+07				5.8E+06
627	Brosimacutin C	$C_{20}H_{22}O_5$	[M+Na]+	365.13594	365.13602	-0.21						2.8E+06		
628	Dinor-iso prostaglandin F2alpha	$C_{18}H_{30}O_5$	[M+K]+	365.17248	365.17253	-0.13		3.0E+06						
629	Dinor-keto-prostaglandin F1 a	$C_{18}H_{30}O_6$	[M+Na]+	365.19346	365.19355	-0.25		2.2E+06				2.4E+06		
630	Epoxy-docosahexaenoic acid	$C_{22}H_{30}O_3$	[M+Na]+	365.20872	365.20893	-0.57		3.8E+06				2.6E+06		
631	Methyl dihydroxy-oxo-octadecenoate	$C_{19}H_{34}O_5$	[M+Na]+	365.22984	365.22990	-0.16		3.4E+06		6.7E+05		3.5E+06		
632	Tricosahexaenoic acid	$C_{23}H_{34}O_2$	[M+Na]+	365.24510	365.24530	-0.54		5.0E+06				3.1E+06		
633	Eicosanedioic acid	$C_{20}H_{38}O_4$	[M+Na]+	365.26623	365.26629	-0.16	2.1E+06		2.2E+06	2.4E+06	1.8E+06		3.5E+06	2.1E+06

				ESI(+) FT-ICR MS											
**No.**	**Plausible Compound**[a]	**Molecular formula (M)**	**Ion**	**Theor m/z**[b]	**Exp. m/z**[c]	**Δppm**[d]	**SM$_R$H**[e]	**SM$_R$O**[e]	**SM$_P$H**[e]	**SM$_P$O**[e]	**TF$_R$H**[e]	**TF$_R$O**[e]	**TF$_P$H**[e]	**TF$_P$O**[e]
634	Hydroxy-heneicosanoic acid	$C_{21}H_{42}O_3$	[M+Na]+	365.30262	365.30272	-0.28				6.6E+05				
635	Methyl-heneicosanediol	$C_{22}H_{46}O_2$	[M+Na]+	365.33900	365.33904	-0.11		1.9E+06		7.2E+05				
636	Dihydroxybenzoxazinone glucoside	$C_{14}H_{17}NO_9$	[M+Na]+	366.07955	366.07976	-0.57	5.0E+06		2.2E+06		5.0E+06			
637	Oxo-dihydroxy-leukotriene B4	$C_{20}H_{29}O_6$	[M+H]+	366.20369	366.20334	0.96						2.4E+06		
638	Sanaganone	$C_{22}H_{16}O_4$	[M+Na]+	367.09408	367.09383	0.69			1.8E+06		2.0E+06			
639	Epiiridotrial glucoside	$C_{16}H_{24}O_8$	[M+Na]+	367.13634	367.13635	-0.03						1.5E+06		
640	Trifluoro-methoxy-norpregna-tetraenol	$C_{21}H_{25}F_3O_2$	[M+H]+	367.18794	367.18820	-0.69		6.6E+06				4.2E+06		
641	Hydroxydocosahexaenoic acid	$C_{22}H_{32}O_3$	[M+Na]+	367.22437	367.22457	-0.57	4.7E+06	2.4E+07	4.4E+06	2.9E+06	3.2E+06	2.7E+07	5.4E+06	
642	Dihydroxy-methoxy-octadecenoic acid	$C_{19}H_{36}O_5$	[M+Na]+	367.24549	367.24553	-0.10		8.1E+06		1.3E+06		6.4E+06		
643	Hydroxy-tetranorvitamin D3	$C_{23}H_{36}O_2$	[M+Na]+	367.26075	367.26089	-0.38						1.8E+06		
644	Dihydroxy arachidic acid	$C_{20}H_{40}O_4$	[M+Na]+	367.28188	367.28193	-0.15	1.6E+06		2.1E+06	2.7E+06			2.9E+06	1.8E+06
645	Docosenyl acetate	$C_{24}H_{46}O_2$	[M+H]+	367.35706	367.35714	-0.23		3.1E+06				3.7E+06		
646	N-oleoyl GABA	$C_{22}H_{41}NO_3$	[M+H]+	368.31592	368.31597	-0.13		7.8E+06				4.2E+06		
647	Gibberellin A24	$C_{20}H_{26}O_5$	[M+Na]+	369.16724	369.16747	-0.60		4.3E+06				2.6E+06		
648	Fluoro-hydroxypregnadienedione	$C_{21}H_{27}FO_3$	[M+Na]+	369.18364	369.18359	0.15		3.8E+06						
649	Hydroxytetradecane-tricarboxylate	$C_{17}H_{30}O_7$	[M+Na]+	369.18837	369.18844	-0.18		2.2E+06				2.7E+06		
650	3-Oxo-hydroxysteroid	$C_{21}H_{30}O_4$	[M+Na]+	369.20363	369.20385	-0.60	1.9E+06	2.3E+07		1.9E+06		1.3E+07		
651	(Methylcrotonoyl)oxylupanine	$C_{20}H_{30}N_2O_3$	[M+Na]+	369.21486	369.21455	0.85						2.2E+06		
652	MG(0:0/16:0/0:0)	$C_{19}H_{38}O_4$	[M+K]+	369.24017	369.24022	-0.12	7.5E+06		8.1E+06	3.4E+06	4.8E+06		8.2E+06	2.4E+06
653	Oxocholatrienoic Acid	$C_{24}H_{32}O_3$	[M+H]+	369.24242	369.24252	-0.27		2.0E+06						
654	Norcholenediol	$C_{23}H_{38}O_2$	[M+K]+	369.27640	369.27652	-0.31		2.6E+06				3.0E+06		
655	Oxo-tricosanoic acid	$C_{23}H_{44}O_3$	[M+H]+	369.33632	369.33638	-0.14		2.1E+06				2.7E+06		
656	Deoxyvitamin D3	$C_{27}H_{44}$	[M+H]+	369.35158	369.35162	-0.10	5.0E+06	4.3E+07	6.2E+06	8.4E+06	1.9E+06	1.2E+08	6.7E+06	7.9E+06
657	Dimethyl-docosanoic acid	$C_{24}H_{48}O_2$	[M+H]+	369.37271	369.37273	-0.07	2.2E+06	5.9E+06	2.2E+06	1.9E+06		1.4E+07	1.9E+06	1.9E+06
658	Prostaglandin G1	$C_{20}H_{33}O_6$	[M+H]+	370.23499	370.23464	0.95		7.0E+06	2.6E+06	1.7E+07	5.8E+06	1.4E+07		7.3E+07
659	Tetradecenoylcarnitine	$C_{21}H_{39}NO_4$	[M+H]+	370.29519	370.29525	-0.17		4.6E+06				1.7E+06		
660	N-palmitoyl leucine	$C_{22}H_{43}NO_3$	[M+H]+	370.33157	370.33164	-0.17		6.3E+06				1.1E+07		
661	Dehydro-fluoro-oxotestololactone	$C_{19}H_{21}FO_4$	[M+K]+	371.10555	371.10554	0.02	1.6E+06							
662	Leonuridine	$C_{15}H_{24}O_9$	[M+Na]+	371.13125	371.13134	-0.23		4.9E+06				1.7E+06		

No.	Plausible Compound[a]	Molecular formula (M)	Ion	Theor m/z[b]	Exp. m/z[c]	Δppm[d]	SM_RH^e	SM_RO^e	SM_PH^e	SM_PO^e	TF_RH^e	TF_RO^e	TF_PH^e	TF_PO^e
				ESI(+) FT-ICR MS										
663	Bis(dimethoxyphenyl)propanediol	$C_{19}H_{24}O_6$	[M+Na]+	371.14651	371.14673	-0.59		1.6E+06						
664	Fluoro-hydroxypregnenedione	$C_{21}H_{29}FO_3$	[M+Na]+	371.19929	371.19922	0.20		6.6E+06						
665	Deoxy-methylene-keto-PGD2	$C_{21}H_{32}O_4$	[M+Na]+	371.21928	371.21945	-0.46		1.7E+06						
666	Trimethy eicosatrienoic acid	$C_{23}H_{40}O_2$	[M+Na]+	371.29205	371.29212	-0.18		3.6E+07		6.6E+06		3.8E+07		
667	Docosanedioic acid	$C_{22}H_{42}O_4$	[M+H]+	371.31559	371.31564	-0.16	1.8E+06	3.3E+07	3.4E+06	2.4E+06		1.8E+07	2.8E+06	2.1E+06
668	Cholestene	$C_{27}H_{46}$	[M+H]+	371.36723	371.36719	0.10						1.4E+06		
669	N-(fluro-ethyl) arachidonoyl amine	$C_{22}H_{36}FNO$	[M+Na]+	372.26731	372.26761	-0.80								1.7E+06
670	N-stearoyl serine	$C_{21}H_{41}NO_4$	[M+H]+	372.31084	372.31091	-0.19		4.7E+06				2.5E+06		
671	Fluoro-hydroxyandrostenetrione	$C_{19}H_{23}FO_4$	[M+K]+	373.12120	373.12107	0.34					1.6E+06			
672	Deoxyoleandolide	$C_{20}H_{36}O_6$	[M+H]+	373.25847	373.25853	-0.17						3.0E+06		
673	Hydroxy-N-desmethyltamoxifen	$C_{25}H_{27}NO_2$	[M+H]+	374.21146	374.21145	0.02	2.8E+06	9.2E+06	2.6E+06	3.4E+06	2.0E+06	7.3E+06	2.9E+06	8.3E+06
674	MG(18:3/0:0/0:0)	$C_{21}H_{36}O_4$	[M+Na]+	375.25058	375.25066	-0.22		5.7E+06		7.0E+05		3.1E+06		
675	Methyl-(cyclopentadienylidene)-androstanol	$C_{25}H_{36}O$	[M+Na]+	375.26584	375.26604	-0.53		4.7E+06				2.5E+06		
676	Prostaglandin H1	$C_{20}H_{33}O_5$	[M+Na]+	376.22202	376.22237	-0.93				3.1E+06		4.5E+06		3.6E+06
677	C16 Sphinganine-phosphate	$C_{16}H_{36}NO_5P$	[M+Na]+	376.22233	376.22239	-0.15				3.1E+06		4.5E+06	1.7E+06	3.6E+06
678	Anandamide (20:l, n-9)	$C_{22}H_{43}NO_2$	[M+Na]+	376.31860	376.31872	-0.32								1.5E+06
679	Chlorogenic acid	$C_{16}H_{18}O_9$	[M+Na]+	377.08430	377.08421	0.24	1.5E+06		3.5E+06		2.2E+06			
680	Dimethoxyestra-pentaenecarboxylic acid methyl ester	$C_{22}H_{26}O_4$	[M+Na]+	377.17233	377.17254	-0.56		3.2E+06						
681	Yohimbine	$C_{21}H_{26}N_2O_3$	[M+Na]+	377.18356	377.18329	0.73	2.2E+06	5.6E+06	2.1E+06	6.7E+05	1.9E+06	7.0E+06	2.5E+06	2.9E+06
682	Hydroperoxy-[epidioxy-(octenyl)-cyclopentyl]-heptenoic acid	$C_{19}H_{30}O_6$	[M+Na]+	377.19346	377.19354	-0.20		2.6E+06				1.6E+06		
683	Hydroxy-mercaptoandrostenone propionate	$C_{22}H_{32}O_3S$	[M+H]+	377.21449	377.21467	-0.47						2.0E+06		
684	Aspidospermine	$C_{22}H_{30}N_2O_2$	[M+Na]+	377.21995	377.21987	0.21		3.2E+06				3.1E+06		1.5E+06
685	trihydroxy-prostadienoic acid	$C_{20}H_{34}O_5$	[M+Na]+	377.22984	377.22995	-0.28		3.4E+06				2.1E+06		
686	MG(18:2/0:0/0:0)	$C_{21}H_{38}O_4$	[M+Na]+	377.26623	377.26628	-0.12				1.9E+06			1.9E+06	1.3E+06
687	Dinor-cholestenynol	$C_{25}H_{38}O$	[M+Na]+	377.28149	377.28171	-0.58		3.8E+06				3.0E+06		
688	Lignoceryl alcohol	$C_{24}H_{50}O$	[M+Na]+	377.37539	377.37551	-0.31		1.9E+06				2.1E+06		
689	Dioncophylline A	$C_{24}H_{27}NO_3$	[M+H]+	378.20637	378.20639	-0.04		3.7E+06				2.8E+06		
690	Evocarpine	$C_{23}H_{33}NO$	[M+K]+	378.21937	378.21904	0.90						2.9E+06		2.8E+06

No.	Plausible Compound[a]	Molecular formula (M)	Ion	Theor m/z[b]	Exp. m/z[c]	Δppm[d]	SM$_R$H[e]	SM$_R$O[e]	SM$_P$H[e]	SM$_P$O[e]	TF$_R$H[e]	TF$_R$O[e]	TF$_P$H[e]	TF$_P$O[e]
				ESI(+) FT-ICR MS										
691	O-Feruloyl-D-glucose	$C_{16}H_{20}O_9$	[M+Na]+	379.09995	379.10004	-0.22	2.0E+06		2.2E+06		2.4E+06		1.8E+06	
692	Dihydroxy-methoxy-(hydroxybenzyl)dihydrochalcone	$C_{23}H_{22}O_5$	[M+H]+	379.15400	379.15428	-0.74		2.2E+06						
693	Dioxopregna-dienoic acid methyl ester	$C_{22}H_{28}O_4$	[M+Na]+	379.18798	379.18818	-0.51	1.9E+06	2.2E+06						
694	methyl-dihydroperoxy-octadecatrienoate	$C_{19}H_{32}O_6$	[M+Na]+	379.20911	379.20925	-0.37						1.9E+06		
695	Dehydropregnenolone acetate	$C_{23}H_{32}O_3$	[M+Na]+	379.22437	379.22457	-0.53	1.8E+06	3.2E+06	2.0E+06			2.5E+06	1.9E+06	
696	Hydroxyestrenone benzoate	$C_{25}H_{30}O_3$	[M+H]+	379.22677	379.22685	-0.21		2.0E+06						
697	Dihydro PGE1	$C_{20}H_{36}O_5$	[M+Na]+	379.24549	379.24558	-0.21		2.2E+06				2.9E+06		
698	Docosahexaenoic acid ethyl ester	$C_{24}H_{36}O_2$	[M+Na]+	379.26075	379.26095	-0.52		2.8E+06	2.1E+06			2.2E+06		
699	MG(0:0/18:1/0:0)	$C_{21}H_{40}O_4$	[M+Na]+	379.28188	379.28195	-0.17	4.3E+06		5.5E+06	4.6E+06	2.5E+06		7.9E+06	5.8E+06
700	Bisnor-dehydro-cholesterol	$C_{25}H_{40}O$	[M+Na]+	379.29714	379.29734	-0.55	2.9E+06	7.8E+06	4.4E+06	2.1E+06	1.8E+06	6.9E+06	4.0E+06	
701	Hydroxydocosanoic acid	$C_{22}H_{44}O_3$	[M+Na]+	379.31827	379.31835	-0.21		3.3E+06		1.2E+06		3.2E+06		1.8E+06
702	cyclo-Dopa glucoside	$C_{15}H_{19}NO_9$	[M+Na]+	380.09520	380.09539	-0.49			3.7E+06					
703	Hydroxydihydrochelirubine	$C_{21}H_{17}NO_6$	[M+H]+	380.11286	380.11291	-0.12			1.5E+06					
704	Propafenone	$C_{21}H_{27}NO_3$	[M+K]+	380.16225	380.16192	0.88						1.9E+06		
705	N-palmitoyl threonine	$C_{20}H_{39}NO_4$	[M+Na]+	380.27713	380.27718	-0.14			2.8E+06		4.3E+06			3.7E+06
706	Tetrahydroxy-methoxyflavone acetate	$C_{18}H_{14}O_8$	[M+Na]+	381.05809	381.05821	-0.31	6.4E+06		5.7E+06		7.2E+06		4.3E+06	
707	Phaseollidin hydrate	$C_{20}H_{22}O_5$	[M+K]+	381.10988	381.10979	0.24	3.6E+06				3.4E+06			
708	Prednisone	$C_{21}H_{26}O_5$	[M+Na]+	381.16724	381.16740	-0.42	1.7E+06	2.3E+06				2.1E+06		
709	PA(14:1/0:0)	$C_{17}H_{33}O_7P$	[M+H]+	381.20367	381.20384	-0.45	2.1E+06	2.8E+06	2.5E+06		2.3E+06	2.2E+06	2.1E+06	
710	Hydroxy-oxo-tetranorcalciol	$C_{23}H_{34}O_3$	[M+Na]+	381.24002	381.24023	-0.55	1.0E+07	1.2E+07	1.1E+07	3.1E+06	5.5E+06	9.4E+06	1.1E+07	2.1E+06
711	Oxopregnene carboxaldehyde dioxime	$C_{22}H_{34}N_2O_2$	[M+Na]+	381.25125	381.25146	-0.56	1.6E+07	2.4E+07	1.6E+07	1.2E+07	4.7E+06	5.7E+06	1.6E+07	2.1E+06
712	Dimethoxy-hydroxy-octadecenoic acid	$C_{20}H_{38}O_5$	[M+Na]+	381.26115	381.26121	-0.17		3.7E+06		1.5E+06				2.6E+06
713	Tetracosapentaenoic acid	$C_{24}H_{38}O_2$	[M+Na]+	381.27640	381.27650	-0.27		3.2E+06		1.0E+06		3.2E+06		
714	MG(0:0/18:0/0:0)	$C_{21}H_{42}O_4$	[M+Na]+	381.29753	381.29760	-0.17	2.2E+07		2.5E+07	5.3E+06	1.9E+07			1.9E+07
715	Pentamethyleicosa-pentaenol	$C_{25}H_{42}O$	[M+Na]+	381.31279	381.31299	-0.53		1.7E+06						
716	Oxo-tetracosenoic acid	$C_{24}H_{44}O_3$	[M+H]+	381.33632	381.33632	0.00		1.9E+06				1.8E+06		
717	Trimethyl-docosenoic acid	$C_{25}H_{48}O_2$	[M+H]+	381.37271	381.37271	-0.01						2.4E+06		
718	Symlandine	$C_{20}H_{31}NO_6$	[M+H]+	382.22241	382.22248	-0.18	2.7E+06		4.6E+06		3.4E+06			

				ESI(+) FT-ICR MS									
**No.**	**Plausible Compound**[a]	**Molecular formula (M)**	**Ion**	**Theor m/z**[b]	**Exp. m/z**[c]	**Δppm**[d]	**SM$_{R}$H**[e]	**SM$_{R}$O**[e]	**SM$_{P}$H**[e]	**SM$_{P}$O**[e]	**TF$_{R}$H**[e]	**TF$_{R}$O**[e]	**TF$_{P}$H**[e]	**TF$_{P}$O**[e]
719	Sphinganine 1-phosphate	$C_{18}H_{40}NO_5P$	[M+H]+	382.27169	382.27189	-0.52		2.3E+06				3.1E+06		
720	N-dihydroxy-eicosatrienoyl)-ethanolamine	$C_{22}H_{39}NO_4$	[M+H]+	382.29519	382.29524	-0.13		2.0E+06	1.9E+06					
721	N-stearoyl proline	$C_{23}H_{43}NO_3$	[M+H]+	382.33157	382.33166	-0.23	2.1E+06					2.0E+06		
722	Trihydroxy-trimethoxyflavone	$C_{18}H_{16}O_8$	[M+Na]+	383.07374	383.07397	-0.61	9.2E+06							
723	Fisetinidolol-pentamethyl ether	$C_{20}H_{24}O_6$	[M+Na]+	383.14651	383.14671	-0.53	1.7E+07	2.9E+06	2.4E+07		2.3E+07		1.9E+07	
724	Aldosterone	$C_{21}H_{28}O_5$	[M+Na]+	383.18289	383.18312	-0.60	6.0E+06	6.4E+06	5.8E+06		4.4E+06	4.6E+06	5.2E+06	1.8E+06
725	PA(14:0/0:0)	$C_{17}H_{35}O_7P$	[M+H]+	383.21932	383.21948	-0.44	2.5E+06	4.8E+06	3.6E+06	2.5E+06		3.5E+06	2.8E+06	
726	Dihydroxy-didehydro-dihydro-apo-carotenal	$C_{25}H_{34}O_3$	[M+H]+	383.25807	383.25817	-0.26		2.8E+06		4.2E+06		2.8E+06		6.3E+06
727	MG(18:0/0:0/0:0)	$C_{21}H_{44}O_3$	[M+K]+	383.29220	383.29219	0.05		1.9E+06				2.2E+06		
728	Didehydrovitamin D3	$C_{27}H_{42}O$	[M+H]+	383.33084	383.33089	-0.13	3.3E+06	4.6E+06	4.8E+06	1.4E+06	4.2E+06	5.5E+06	4.0E+06	1.9E+06
729	Hydroxy-tetracosenoic acid	$C_{24}H_{46}O_3$	[M+H]+	383.35197	383.35203	-0.15		3.3E+06		1.4E+06		3.6E+06		1.4E+06
730	Mycocerosic acid	$C_{25}H_{50}O_2$	[M+H]+	383.38836	383.38841	-0.13	4.7E+06	4.6E+06	4.9E+06	2.4E+06	3.6E+06	9.9E+06	4.4E+06	3.2E+06
731	Hydroxybenzyldesulphoglucosinolate	$C_{14}H_{19}NO_7S$	[M+K]+	384.05138	384.05139	-0.02			3.0E+06					
732	Mannose-acetylglucosamine	$C_{14}H_{25}NO_{11}$	[M+H]+	384.15004	384.15014	-0.27	2.6E+06		2.8E+06		4.5E+06			
733	Trihydroxy-leukotriene-B4	$C_{20}H_{31}O_7$	[M+H]+	384.21425	384.21391	0.89				3.4E+06		6.6E+06		2.2E+06
734	Hydroxy-tetradecadiencarnitine	$C_{21}H_{37}NO_5$	[M+H]+	384.27445	384.27453	-0.21		2.2E+06						
735	N-oleoyl threonine	$C_{22}H_{41}NO_4$	[M+H]+	384.31084	384.31090	-0.18		3.6E+06	1.7E+06			1.7E+06		
736	N-(Docosanoyl)-ethanolamine	$C_{24}H_{49}NO_2$	[M+H]+	384.38361	384.38368	-0.20		3.7E+06	2.0E+06			3.1E+06		
737	Chelirubine	$C_{21}H_{16}NO_5$	[M+Na]+	385.09207	385.09215	-0.21					1.8E+06			
738	Dihydroxy-tetramethoxyisoflavan	$C_{19}H_{22}O_7$	[M+Na]+	385.12577	385.12596	-0.47	3.8E+06	3.7E+06	2.8E+06		2.4E+06		3.4E+06	
739	Acetyl-maltose	$C_{14}H_{24}O_{12}$	[M+H]+	385.13405	385.13419	-0.36		1.9E+06						
740	Oxo-carboxy-leukotriene B4	$C_{20}H_{26}O_6$	[M+Na]+	385.16216	385.16236	-0.52	6.7E+06	2.4E+06	5.1E+06		5.0E+06		3.7E+06	
741	Epoxy-fluoro-hydroxypregnendione	$C_{21}H_{27}FO_4$	[M+Na]+	385.17856	385.17845	0.28		2.7E+06						
742	Cortisol	$C_{21}H_{30}O_5$	[M+H]+	385.19854	385.19875	-0.52	1.7E+06		2.1E+06					
743	Di-n-heptyl phthalate	$C_{22}H_{34}O_4$	[M+Na]+	385.23493	385.23511	-0.47	5.0E+06		4.3E+06		5.0E+06	3.5E+06	4.0E+06	1.5E+06
744	Norcholenetriol	$C_{23}H_{38}O_3$	[M+Na]+	385.27132	385.27152	-0.52	4.9E+06	9.7E+06	5.4E+06		2.6E+06	6.9E+06	5.7E+06	1.5E+06
745	Tetracosatrienoic acid	$C_{24}H_{42}O_2$	[M+Na]+	385.30770	385.30779	-0.22		5.3E+06	5.9E+06	7.2E+05		1.6E+07	2.8E+06	7.5E+06
746	MG(20:1/0:0/0:0)	$C_{23}H_{44}O_4$	[M+H]+	385.33124	385.33131	-0.19		3.6E+06		8.0E+05		5.3E+06	1.7E+06	
747	Cholecalciferol	$C_{27}H_{44}O$	[M+H]+	385.34649	385.34655	-0.15	4.0E+06	8.8E+06	6.8E+06	3.7E+06	2.9E+06	1.2E+07	6.4E+06	3.9E+06

				ESI(+) FT-ICR MS									
**No.**	**Plausible Compound**[a]	**Molecular formula (M)**	**Ion**	**Theor m/z**[b]	**Exp. m/z**[c]	**Δppm**[d]	**SM$_R$H**[e]	**SM$_R$O**[e]	**SM$_P$H**[e]	**SM$_P$O**[e]	**TF$_R$H**[e]	**TF$_R$O**[e]	**TF$_P$H**[e]	**TF$_P$O**[e]
748	Dihydro-trihydroxy-leukotriene B4	$C_{20}H_{33}O_7$	[M+H]+	386.22990	386.22956	0.89		8.4E+06		5.9E+06		9.4E+06	3.6E+06	8.8E+06
749	N-palmitoyl glutamic acid	$C_{21}H_{39}NO_5$	[M+H]+	386.29010	386.29014	-0.10		3.2E+06	1.7E+06					
750	Leonuridine	$C_{15}H_{24}O_9$	[M+K]+	387.10519	387.10525	-0.16	2.3E+06		2.7E+06		2.1E+06		2.1E+06	
751	Gibberellin A8	$C_{19}H_{24}O_7$	[M+Na]+	387.14142	387.14164	-0.55	2.2E+06				2.3E+06			
752	Pinoresinol dimethyl ether	$C_{22}H_{26}O_6$	[M+H]+	387.18022	387.18028	-0.18	2.2E+06		2.0E+06	1.5E+06	2.0E+06		2.2E+06	
753	Dihydrocortisol	$C_{21}H_{32}O_5$	[M+Na]+	387.21419	387.21441	-0.56	2.5E+06	1.8E+06	2.2E+06		1.7E+06			
754	Robustadial A	$C_{23}H_{30}O_5$	[M+H]+	387.21660	387.21667	-0.18		2.4E+06						
755	Anacardic acid	$C_{22}H_{36}O_3$	[M+K]+	387.22960	387.22967	-0.17	2.8E+06	4.7E+06	3.3E+06			3.5E+06	3.4E+06	
756	Oxo-hydroxy-dimethyl-prostadienoic acid	$C_{22}H_{36}O_4$	[M+Na]+	387.25058	387.25072	-0.37	4.2E+06	4.0E+06			3.2E+06	2.3E+06		2.1E+06
757	(Ladderane-octanyl)-sn-glycerol	$C_{23}H_{40}O_3$	[M+Na]+	387.28697	387.28702	-0.15	1.7E+06	1.0E+07	3.3E+06	3.2E+06		1.1E+07	4.6E+06	5.4E+06
758	Tetracosadienoic acid	$C_{24}H_{44}O_2$	[M+Na]+	387.32335	387.32345	-0.25		1.9E+06						
759	Ulifloxacin	$C_{16}H_{16}FN_3O_3S$	[M+K]+	388.05280	388.05277	0.08					5.1E+06			
760	Hydroxymyristoylcarnitine	$C_{21}H_{41}NO_5$	[M+H]+	388.30575	388.30580	-0.13	1.7E+06	3.0E+06	2.4E+06			1.9E+06	1.6E+06	
761	Diacetoxyscirpenol	$C_{19}H_{26}O_7$	[M+Na]+	389.15707	389.15728	-0.52	1.5E+07		1.4E+07		8.9E+06		1.2E+07	
762	Cortolone	$C_{21}H_{34}O_5$	[M+Na]+	389.22984	389.23017	-0.84								1.3E+06
763	Tetracosenoic acid	$C_{24}H_{46}O_2$	[M+Na]+	389.33900	389.33907	-0.18	1.8E+06		2.8E+06				4.6E+06	3.8E+06
764	Amino-tetrahydroxyeicosenoic acid	$C_{20}H_{39}NO_6$	[M+H]+	390.28501	390.28505	-0.08	2.2E+06	2.9E+06	2.8E+06		2.0E+06	2.3E+06	1.9E+06	
765	N-oleoyl GABA	$C_{22}H_{41}NO_3$	[M+Na]+	390.29786	390.29798	-0.29								1.6E+06
766	Phosphatidyl-D-myo-inositol	$C_{11}H_{19}O_{13}P$	[M+H]+	391.06360	391.06378	-0.44	2.3E+06				2.1E+06			
767	N-[(Methylenedioxyphenyl)-(mercaptomethyl)-oxoprolyl]-alanine	$C_{16}H_{20}N_2O_6S$	[M+Na]+	391.09343	391.09319	0.61	2.1E+06		1.9E+06		2.2E+06			
768	O-Feruloylquinate	$C_{17}H_{20}O_9$	[M+Na]+	391.09995	391.10017	-0.55	2.7E+06		2.2E+06		2.5E+06		1.9E+06	
769	Dihydroxy-methoxy-prenyloxyflavone	$C_{21}H_{20}O_6$	[M+Na]+	391.11521	391.11542	-0.54	1.6E+06							
770	O-Acetylajmaline	$C_{22}H_{28}N_2O_3$	[M+Na]+	391.19921	391.19894	0.71	4.3E+06	1.1E+07	4.4E+06	2.0E+06	3.7E+06	1.4E+07	4.5E+06	8.2E+06
771	Trifluoro-LTB4	$C_{20}H_{29}F_3O_4$	[M+H]+	391.20907	391.20918	-0.27		2.9E+06				1.9E+06		1.5E+06
772	MG(18:3/0:0/0:0)	$C_{21}H_{36}O_4$	[M+K]+	391.22452	391.22457	-0.14	2.0E+06		1.9E+06			2.5E+06	1.9E+06	
773	Cortol	$C_{21}H_{36}O_5$	[M+Na]+	391.24549	391.24556	-0.15		2.6E+06				2.1E+06		
774	Didesmethyl tocotrienol	$C_{25}H_{36}O_2$	[M+Na]+	391.26075	391.26093	-0.46			1.8E+06					
775	Diisooctyl phthalate	$C_{24}H_{38}O_4$	[M+H]+	391.28429	391.28434	-0.13	3.6E+07	4.0E+07	1.2E+07	5.0E+06	2.4E+07	2.5E+07	1.4E+07	7.1E+06

					ESI(+) FT-ICR MS									
No.	Plausible Compound[a]	Molecular formula (M)	Ion	Theor m/z[b]	Exp. m/z[c]	Δppm[d]	SM_RH^e	SM_RO^e	SM_PH^e	SM_PO^e	TF_RH^e	TF_RO^e	TF_PH^e	TF_PO^e
776	Tricosenoic acid	$C_{23}H_{44}O_2$	[M+K]+	391.29729	391.29730	-0.03			1.8E+06					
777	Oxo-tricosanoic acid	$C_{23}H_{44}O_3$	[M+Na]+	391.31827	391.31833	-0.16								3.4E+06
778	Flavoxate	$C_{24}H_{25}NO_4$	[M+H]+	392.18563	392.18555	0.22						3.2E+06		
779	N-oleoyl alanine	$C_{21}H_{39}NO_3$	[M+K]+	392.25615	392.25626	-0.27	2.0E+06							
780	N-palmitoyl isoleucine	$C_{22}H_{43}NO_3$	[M+Na]+	392.31351	392.31358	-0.16	1.6E+06			2.7E+06			2.5E+06	4.8E+06
781	Stemonone	$C_{19}H_{14}O_8$	[M+Na]+	393.05809	393.05829	-0.51	3.9E+06		1.1E+07		3.6E+06		2.3E+07	
782	Pentahydroxy-trimethoxyflavone	$C_{18}H_{16}O_{10}$	[M+H]+	393.08162	393.08169	-0.17			1.7E+06					
783	C-p-Hydroxybenzylkaempferol	$C_{22}H_{16}O_7$	[M+H]+	393.09688	393.09721	-0.84			1.7E+06					
784	Unanisoflavan [Isoflavans [PK1208]] ([M+Na]+)	$C_{22}H_{26}O_5$	[M+Na]+	393.16724	393.16744	-0.50		2.6E+06			2.6E+06			
785	Oxo-pentanorvitamin D3 6,19-sulfur dioxide adduct	$C_{22}H_{32}O_4S$	[M+H]+	393.20941	393.20956	-0.40	1.4E+08	9.9E+06	1.3E+08	1.1E+07	1.5E+08	1.9E+07	1.0E+08	1.3E+07
786	Hydroxy-PGE1	$C_{20}H_{34}O_6$	[M+Na]+	393.22476	393.22493	-0.42		2.7E+06		1.3E+06		2.4E+06		2.2E+06
787	Hydroxychola-trienoic Acid	$C_{24}H_{34}O_3$	[M+Na]+	393.24002	393.24022	-0.52	7.5E+06	9.7E+06	1.1E+07	4.5E+06	3.7E+06	6.6E+06	1.1E+07	2.0E+06
788	Oxo-docosanoic acid	$C_{22}H_{42}O_3$	[M+K]+	393.27655	393.27660	-0.12	2.1E+06		3.3E+06		1.8E+06		3.0E+06	
789	Docosanedioic acid	$C_{22}H_{42}O_4$	[M+Na]+	393.29753	393.29761	-0.20					5.6E+06			
790	Norcholestenone	$C_{26}H_{42}O$	[M+Na]+	393.31279	393.31299	-0.51	4.2E+06	6.4E+06	4.6E+06	3.9E+06	1.8E+06	6.7E+06	3.7E+06	
791	Hydroxy-tricosanoic acid	$C_{23}H_{46}O_3$	[M+Na]+	393.33392	393.33399	-0.18		3.3E+06		1.5E+06		3.7E+06		1.8E+06
792	(Triaza-indenoanthracenyl)-acetic acid ethylester	$C_{22}H_{17}N_3O_2$	[M+K]+	394.09524	394.09560	-0.91			2.1E+06				1.9E+06	
793	Tylophorine	$C_{24}H_{27}NO_4$	[M+H]+	394.20128	394.20117	0.30						3.2E+06		4.6E+06
794	N-palmitoyl histidine	$C_{22}H_{39}N_3O_3$	[M+H]+	394.30642	394.30648	-0.15	3.0E+06		5.1E+06				1.9E+06	
795	Hydroxy-trimethoxy-methylenedioxyflavone	$C_{19}H_{16}O_8$	[M+Na]+	395.07374	395.07394	-0.51	5.2E+06		7.6E+06		4.4E+06		3.4E+06	
796	Veraguensin	$C_{22}H_{28}O_5$	[M+Na]+	395.18289	395.18284	0.15	2.6E+06				2.6E+06			
797	Hydroxy-pregnenedione acetate	$C_{23}H_{32}O_4$	[M+Na]+	395.21928	395.21947	-0.48	2.4E+06	3.2E+06	2.6E+06		1.8E+06	2.4E+06	2.8E+06	
798	Deoxyoleandolide	$C_{20}H_{36}O_6$	[M+Na]+	395.24041	395.24046	-0.13	2.8E+06		2.8E+06	2.5E+06	3.9E+06		3.0E+06	5.6E+06
799	Hydroxycholadienoic Acid	$C_{24}H_{36}O_3$	[M+Na]+	395.25567	395.25587	-0.52	1.5E+07	1.7E+07	1.7E+07	5.0E+06	5.1E+06	1.2E+07	1.9E+07	2.7E+06
800	Dysideapalaunic acid	$C_{25}H_{40}O_2$	[M+Na]+	395.29205	395.29223	-0.46	2.1E+06	2.8E+06	2.4E+06			2.3E+06	2.4E+06	
801	Dihydroxy-docosanoic acid	$C_{22}H_{44}O_4$	[M+Na]+	395.31318	395.31323	-0.12		3.7E+06	2.0E+06	1.6E+06		4.6E+06		2.6E+06
802	Hexacosenoic acid	$C_{26}H_{50}O_2$	[M+H]+	395.38836	395.38839	-0.08	1.8E+06	2.5E+06	2.0E+06			3.4E+06	1.8E+06	
803	Deacetylcolchicine	$C_{20}H_{23}NO_5$	[M+K]+	396.12078	396.12061	0.43								2.6E+06
804	PC(O-10:1/0:0)	$C_{18}H_{38}NO_6P$	[M+H]+	396.25095	396.25110	-0.37	1.7E+07	2.0E+07	8.6E+06	1.3E+07	1.0E+07	4.9E+06	1.6E+07	3.0E+06

					ESI(+) FT-ICR MS									
**No.**	**Plausible Compound**[a]	**Molecular formula (M)**	**Ion**	**Theor m/z**[b]	**Exp. m/z**[c]	**Δppm**[d]	**SM_RH**[e]	**SM_RO**[e]	**SM_PH**[e]	**SM_PO**[e]	**TF_RH**[e]	**TF_RO**[e]	**TF_PH**[e]	**TF_PO**[e]
805	Prostaglandin E2 ethanolamide	$C_{22}H_{37}NO_5$	[M+H]+	396.27445	396.27450	-0.11	1.9E+06		2.1E+06					
806	Dihydroxy-tetramethoxyflavone	$C_{19}H_{18}O_8$	[M+Na]+	397.08939	397.08955	-0.41	2.3E+06				2.4E+06			
807	Hydroxy-dioxopregn-enoic acid	$C_{22}H_{30}O_5$	[M+Na]+	397.19854	397.19876	-0.54	2.8E+06	1.2E+07	2.6E+06	3.0E+06	2.5E+06	4.5E+06	2.6E+06	1.8E+06
808	Hydroxy-tetranorvitamin D3 carboxylic acid	$C_{23}H_{34}O_4$	[M+Na]+	397.23493	397.23512	-0.48	4.1E+06	3.5E+06	4.3E+06		2.9E+06	3.6E+06	3.7E+06	
809	Hydroxycholenoic Acid	$C_{24}H_{38}O_3$	[M+Na]+	397.27132	397.27150	-0.46	1.0E+08	1.3E+07	9.9E+07	6.9E+06	5.3E+07	1.2E+07	9.4E+07	9.6E+06
810	Pentacosadiynoic acid	$C_{25}H_{42}O_2$	[M+Na]+	397.30770	397.30788	-0.46	2.2E+06	1.8E+06					1.9E+06	
811	Hydroxynervonic acid	$C_{25}H_{48}O_3$	[M+H]+	397.36762	397.36769	-0.18		2.7E+06	1.9E+06	2.1E+06		3.5E+06	2.1E+06	1.9E+06
812	Trimethyl-tricosanoic acid	$C_{26}H_{52}O_2$	[M+H]+	397.40401	397.40405	-0.10	5.0E+06	5.7E+06	4.6E+06	2.4E+06	2.5E+06	1.0E+07	4.2E+06	3.3E+06
813	PC(4:0/4:0)	$C_{16}H_{32}NO_8P$	[M+H]+	398.19383	398.19400	-0.43							2.0E+06	
814	N-(trihydroxy-prostadienoyl)-ethanolamine	$C_{22}H_{39}NO_5$	[M+H]+	398.29010	398.29017	-0.18			2.0E+06					
815	Hexadecenoyl carnitine	$C_{23}H_{43}NO_4$	[M+H]+	398.32649	398.32659	-0.26		2.9E+06				2.1E+06		
816	Dihydroxy-tetramethoxypterocarpan	$C_{19}H_{20}O_8$	[M+Na]+	399.10504	399.10524	-0.51	2.8E+06		2.1E+06		2.7E+06			
817	Hydroxy-pentamethoxyisoflavan	$C_{20}H_{24}O_7$	[M+Na]+	399.14142	399.14163	-0.52	6.8E+06	5.4E+06	4.7E+06		4.5E+06		4.4E+06	
818	S-Adenosyl-L-methionine	$C_{15}H_{22}N_6O_5S$	[M+H]+	399.14452	399.14455	-0.09	2.8E+06		5.2E+06		3.6E+06		3.2E+06	
819	Oxocortisol	$C_{21}H_{28}O_6$	[M+Na]+	399.17781	399.17799	-0.44	2.0E+06		2.7E+06		2.5E+06			
820	di-O-hexanoyl-glucopyranose	$C_{18}H_{32}O_8$	[M+Na]+	399.19894	399.19898	-0.10				1.3E+06	2.6E+06	2.9E+06		
821	MG(0:0/20:5/0:0)	$C_{23}H_{36}O_4$	[M+Na]+	399.25058	399.25069	-0.26	7.5E+06	5.3E+06	5.9E+06	1.7E+06	4.8E+06	4.5E+06	4.2E+06	2.1E+06
822	Oxoprevitamin D3	$C_{27}H_{42}O_2$	[M+H]+	399.32576	399.32578	-0.05	1.7E+06	2.4E+06	1.8E+06		1.8E+06	3.3E+06		
823	Axillarenic acid	$C_{24}H_{46}O_4$	[M+H]+	399.34689	399.34693	-0.10	2.2E+06	4.3E+06	1.8E+06			7.1E+06		2.1E+06
824	Methylvitamin D3	$C_{28}H_{46}O$	[M+H]+	399.36214	399.36221	-0.16		1.7E+06	1.9E+06			3.4E+06		
825	L-Palmitoylcarnitine	$C_{23}H_{45}NO_4$	[M+H]+	400.34214	400.34219	-0.14	4.9E+06	7.3E+06	5.9E+06	1.7E+06	3.0E+06	4.9E+06	6.3E+06	2.3E+06
826	Hydroxyvernolide	$C_{19}H_{22}O_8$	[M+Na]+	401.12069	401.12089	-0.50	3.0E+06		2.4E+06		2.1E+06		2.1E+06	
827	Dihydroxy-methoxy-(hydroxybenzyl)dihydrochalcone	$C_{23}H_{22}O_5$	[M+H]+	401.13594	401.13614	-0.49	2.5E+06							
828	Epoxy-fluoro-hydroxypregnendione	$C_{21}H_{27}FO_4$	[M+K]+	401.15250	401.15238	0.30	2.6E+06		2.9E+06		3.7E+06			
829	Gibberellin A17	$C_{20}H_{26}O_7$	[M+Na]+	401.15707	401.15726	-0.45	2.0E+06				2.1E+06			
830	Trioxo-cholenoic Acid	$C_{24}H_{32}O_5$	[M+H]+	401.23225	401.23233	-0.20		6.1E+06		2.4E+06		3.5E+06		2.9E+06
831	MG(20:4/0:0/0:0)	$C_{23}H_{38}O_4$	[M+Na]+	401.26623	401.26642	-0.48	1.7E+07	3.0E+07	1.7E+07	4.4E+06	6.7E+06	1.9E+07	2.0E+07	2.4E+06
832	Cholesta-tetraenone	$C_{27}H_{38}O$	[M+Na]+	401.28149	401.28169	-0.49						8.4E+06	4.0E+06	

				ESI(+) FT-ICR MS										
**No.**	**Plausible Compound[a]**	**Molecular formula (M)**	**Ion**	**Theor m/z[b]**	**Exp. m/z[c]**	**Δppm[d]**	**SM_RH^e**	**SM_RO^e**	**SM_PH^e**	**SM_PO^e**	**TF_RH^e**	**TF_RO^e**	**TF_PH^e**	**TF_PO^e**
833	Cholantriol	$C_{24}H_{42}O_3$	[M+Na]+	401.30262	401.30267	-0.14		2.0E+06				2.9E+06		1.8E+06
834	Hydroxyvitamin D3	$C_{27}H_{44}O_2$	[M+H]+	401.34141	401.34146	-0.13	2.5E+06	5.0E+06	2.4E+06	1.8E+06	1.8E+06	7.4E+06	2.7E+06	2.4E+06
835	Leukotriene B4 ethanolamide	$C_{22}H_{37}NO_4$	[M+Na]+	402.26148	402.26168	-0.50								1.7E+06
836	Sphingofungin F	$C_{21}H_{39}NO_6$	[M+H]+	402.28501	402.28504	-0.06		2.2E+06	4.5E+06			3.5E+06	2.6E+06	
837	N-propyl-dimethyl-docosatetraenoyl amine	$C_{27}H_{47}NO$	[M+H]+	402.37304	402.37310	-0.13		3.2E+06	2.5E+06			4.1E+06	2.2E+06	
838	Tetracenomycin D3	$C_{20}H_{12}O_8$	[M+Na]+	403.04244	403.04266	-0.54	2.5E+06		3.2E+06		3.3E+06			
839	Chaparrin	$C_{20}H_{28}O_7$	[M+Na]+	403.17272	403.17288	-0.39	3.1E+06		2.8E+06		2.4E+06		2.6E+06	
840	Oxo-hydroxy-dimethyl-prostadienoic acid	$C_{22}H_{36}O_4$	[M+K]+	403.22452	403.22454	-0.05			2.2E+06					
841	Dihydroxy-diperoxy-eicosadienoic acid	$C_{20}H_{34}O_8$	[M+H]+	403.23264	403.23268	-0.08		4.5E+06	2.6E+06			3.0E+06		
842	Dihydroxy-oxo-dimethyl-prostadienoic acid	$C_{22}H_{36}O_5$	[M+Na]+	403.24549	403.24566	-0.42		2.7E+06		1.9E+06		2.1E+06		
843	Arachidonyl Glycerol ether	$C_{23}H_{40}O_3$	[M+K]+	403.26090	403.26091	-0.01					1.9E+06			
844	MG(0:0/20:3/0:0)	$C_{23}H_{40}O_4$	[M+Na]+	403.28188	403.28192	-0.10		4.5E+06		2.3E+06		4.5E+06	3.3E+06	2.8E+06
845	Oxo-tetracosenoic acid	$C_{24}H_{44}O_3$	[M+Na]+	403.31827	403.31830	-0.08								2.0E+06
846	Pentacosenoic acid	$C_{25}H_{48}O_2$	[M+Na]+	403.35465	403.35473	-0.19				1.3E+06				2.3E+06
847	N-palmitoyl phenylalanine	$C_{25}H_{41}NO_3$	[M+H]+	404.31592	404.31594	-0.05	2.0E+06						2.1E+06	
848	Dihydroxy-dimethoxy-prenylisoflavone	$C_{22}H_{22}O_6$	[M+Na]+	405.13086	405.13105	-0.46	3.9E+06		3.3E+06					
849	(Methyl-(methylpropyl)benzeneacetate)-Glucopyranuronic acid	$C_{19}H_{26}O_8$	[M+Na]+	405.15199	405.15217	-0.44	1.0E+07		7.8E+06		8.9E+06		6.2E+06	
850	Dioxocholatrienoic Acid	$C_{24}H_{30}O_4$	[M+Na]+	405.20363	405.20370	-0.17						2.1E+06		
851	Hydroxy-mercapto-pregnendione acetate	$C_{23}H_{32}O_4S$	[M+H]+	405.20941	405.20959	-0.45			2.7E+06					
852	Sarcostin	$C_{21}H_{34}O_6$	[M+Na]+	405.22476	405.22499	-0.56	1.7E+06	3.3E+06		2.3E+06		2.2E+06		2.2E+06
853	Dihydroxy-didehydro-dihydro-apo-beta-carotenal	$C_{25}H_{34}O_3$	[M+Na]+	405.24002	405.23993	0.20	2.1E+06		3.2E+06				2.8E+06	
854	Methyl trihydroxy-methyl-prostadienoate	$C_{22}H_{38}O_5$	[M+Na]+	405.26115	405.26121	-0.16		1.9E+06						
855	MG(0:0/20:2/0:0)	$C_{23}H_{42}O_4$	[M+Na]+	405.29753	405.29756	-0.06		3.0E+06						2.1E+06
856	Dehydroprevitamin D3	$C_{27}H_{42}O$	[M+Na]+	405.31279	405.31294	-0.38					1.6E+06			
857	O-fucopyranosyl-acetamido-deoxyglucopyranose	$C_{14}H_{25}NO_{10}$	[M+Na]+	406.11101	406.11106	-0.13	2.0E+06							
858	Hydroxy-tetradecadiencarnitine	$C_{21}H_{37}NO_5$	[M+Na]+	406.25639	406.25643	-0.09						2.7E+06		
859	N-oleoyl threonine	$C_{22}H_{41}NO_4$	[M+Na]+	406.29278	406.29284	-0.14				4.1E+06				3.2E+06
860	Indolylmethyl-desulfoglucosinolate	$C_{16}H_{20}N_2O_6S$	[M+K]+	407.06737	407.06719	0.43							1.7E+07	

				ESI(+) FT-ICR MS										
No.	Plausible Compound[a]	Molecular formula (M)	Ion	Theor m/z[b]	Exp. m/z[c]	Δppm[d]	SM_RH[e]	SM_RO[e]	SM_PH[e]	SM_PO[e]	TF_RH[e]	TF_RO[e]	TF_PH[e]	TF_PO[e]
861	Dihydroxy-dimethoxy-prenylflavanone	$C_{22}H_{24}O_6$	[M+Na]+	407.14651	407.14668	-0.42	1.1E+07	2.8E+06	9.6E+06		5.5E+06		6.7E+06	
862	Argentine	$C_{23}H_{26}N_4O_3$	[M+H]+	407.20777	407.20812	-0.87				1.2E+06				3.1E+06
863	Dihydroxy-(p-menthenyl)-methoxychalcone	$C_{26}H_{30}O_4$	[M+H]+	407.22169	407.22179	-0.26								1.8E+06
864	Hexacosahexaenoic acid	$C_{26}H_{40}O_2$	[M+Na]+	407.29205	407.29225	-0.50	2.2E+06	2.8E+06	2.9E+06		1.6E+06	3.7E+06	2.9E+06	1.7E+06
865	Diapophytofluene	$C_{30}H_{46}$	[M+H]+	407.36723	407.36727	-0.09		3.4E+06				2.6E+06		
866	Hydroxy-(morpholinylmethyl)estratrienone	$C_{23}H_{31}NO_3$	[M+K]+	408.19355	408.19317	0.94						4.6E+06		
867	N-palmitoyl glutamic acid	$C_{21}H_{39}NO_5$	[M+Na]+	408.27204	408.27220	-0.38								2.9E+06
868	N-palmitoyl leucine	$C_{22}H_{43}NO_3$	[M+K]+	408.28745	408.28748	-0.06	2.8E+06		2.6E+06			3.7E+06		
869	Hydroxychalcone-glucoside	$C_{21}H_{22}O_7$	[M+Na]+	409.12577	409.12583	-0.14		1.0E+07						
870	Di-O-methyl-prenylafzelechinol	$C_{22}H_{26}O_6$	[M+Na]+	409.16216	409.16228	-0.30		3.2E+06						2.9E+06
871	Epoxy-dioxopregnenyl acetate	$C_{23}H_{30}O_5$	[M+Na]+	409.19854	409.19869	-0.35							3.4E+06	
872	Oleandolide	$C_{20}H_{34}O_7$	[M+Na]+	409.21967	409.21979	-0.28		3.0E+06			4.5E+06	2.1E+06		
873	Dioxocholenoic Acid	$C_{24}H_{34}O_4$	[M+Na]+	409.23493	409.23511	-0.44	2.2E+06	2.7E+06	2.6E+06			2.6E+06	3.3E+06	
874	Deoxyerythronolide B	$C_{21}H_{38}O_6$	[M+Na]+	409.25606	409.25612	-0.14		6.2E+06		1.3E+06		3.7E+06		3.2E+06
875	Testosterone isocaproate	$C_{25}H_{38}O_3$	[M+Na]+	409.27132	409.27152	-0.49	2.1E+07	6.4E+07	3.1E+07	1.1E+07	9.9E+06	2.9E+07	2.2E+07	5.6E+06
876	DG(18:0/2:0/0:0)	$C_{23}H_{46}O_4$	[M+Na]+	409.32883	409.32888	-0.12		4.1E+06	2.6E+06	9.8E+05		4.5E+06	2.6E+06	3.3E+06
877	Epoxycholestane	$C_{27}H_{46}O$	[M+Na]+	409.34409	409.34413	-0.11	4.5E+06	1.3E+07	6.9E+06	7.9E+06	2.5E+06	2.8E+07	6.2E+06	1.0E+07
878	Oxohexacosenoic acid	$C_{26}H_{48}O_3$	[M+H]+	409.36762	409.36768	-0.14						2.4E+06		
879	Diapophytoene	$C_{30}H_{48}$	[M+H]+	409.38288	409.38292	-0.11	6.5E+06	2.0E+07	9.0E+06	2.7E+06	2.7E+06	1.8E+07	8.6E+06	5.8E+06
880	Phthienoic acid	$C_{27}H_{52}O_2$	[M+H]+	409.40401	409.40408	-0.18		2.8E+06				2.6E+06		
881	Linustatin	$C_{16}H_{27}NO_{11}$	[M+H]+	410.16569	410.16559	0.24				1.7E+06		1.9E+06		
882	Hexamethoxychalcone	$C_{21}H_{24}O_7$	[M+Na]+	411.14142	411.14158	-0.38		2.0E+06						
883	Methyl bisepidioxy-hydroperoxy-octadecenoate	$C_{19}H_{32}O_8$	[M+Na]+	411.19894	411.19906	-0.30		2.3E+06						
884	Dihydroxyandrostenone diacetate	$C_{23}H_{32}O_5$	[M+Na]+	411.21419	411.21441	-0.52	8.2E+06	5.1E+06	6.6E+06		6.3E+06	4.5E+06	6.4E+06	
885	Hydroxy-oxo-cholenoic Acid	$C_{24}H_{36}O_4$	[M+Na]+	411.25058	411.25077	-0.47	3.9E+06	4.0E+06	4.0E+06	2.3E+06	2.3E+06	3.4E+06	4.2E+06	2.1E+06
886	Desoxyscalarin	$C_{25}H_{40}O_3$	[M+Na]+	411.28697	411.28714	-0.43	2.5E+06	3.4E+06	3.7E+06	1.4E+06	1.9E+06	2.8E+06	2.8E+06	
887	Gama-Tocotrienol	$C_{28}H_{42}O_2$	[M+H]+	411.32576	411.32579	-0.08		3.5E+06						
888	Serratene	$C_{30}H_{50}$	[M+H]+	411.39853	411.39857	-0.09	5.3E+07	5.7E+07	5.1E+07	1.4E+07	1.4E+07	9.2E+07	3.8E+07	3.4E+07
889	Methyl-hexacosanoic acid	$C_{27}H_{54}O_2$	[M+H]+	411.41966	411.41972	-0.14	2.5E+06	3.0E+06	2.6E+06			4.9E+06	2.1E+06	2.1E+06

					ESI(+) FT-ICR MS								
**No.**	**Plausible Compound[a]**	**Molecular formula (M)**	**Ion**	**Theor m/z[b]**	**Exp. m/z[c]**	**Δppm[d]**	**SM$_R$H[e]**	**SM$_R$O[e]**	**SM$_P$H[e]**	**SM$_P$O[e]**	**TF$_R$H[e]**	**TF$_R$O[e]**	**TF$_P$H[e]**	**TF$_P$O[e]**
890	N-oleoyl glutamic acid	$C_{23}H_{41}NO_5$	[M+H]+	412.30575	412.30583	-0.19		1.2E+07						
891	Lignoceroyl-ethanolamine	$C_{26}H_{53}NO_2$	[M+H]+	412.41491	412.41495	-0.09		4.4E+06	2.9E+06			3.4E+06	1.9E+06	
892	Erioflorin acetate	$C_{21}H_{26}O_7$	[M+Na]+	413.15707	413.15727	-0.48	3.0E+06		2.7E+06		2.5E+06		2.6E+06	
893	Hydroxy-dihydrojasmonic acid glucoside	$C_{18}H_{30}O_9$	[M+Na]+	413.17820	413.17831	-0.26					2.4E+06			
894	Rehmaionoside B	$C_{19}H_{34}O_8$	[M+Na]+	413.21459	413.21465	-0.16	6.8E+06		6.3E+06		8.0E+06	1.9E+06	6.4E+06	
895	Dihydroxy-dinor-secovitamin D3	$C_{25}H_{42}O_3$	[M+Na]+	413.30262	413.30280	-0.43	1.8E+06	2.6E+06	2.0E+06			2.8E+06		
896	MG(0:0/22:1/0:0)	$C_{25}H_{48}O_4$	[M+H]+	413.36254	413.36259	-0.12	2.1E+06	2.8E+06				4.7E+06		
897	Icaceine	$C_{22}H_{33}NO_4$	[M+K]+	414.20412	414.20434	-0.54					2.4E+06			
898	Tetrahydroxy-trimethoxyflavone	$C_{18}H_{16}O_9$	[M+K]+	415.04259	415.04264	-0.12				8.7E+07				1.8E+08
899	Epi-Eupatoroxin	$C_{20}H_{24}O_8$	[M+Na]+	415.13634	415.13653	-0.45	2.6E+06				2.0E+06			
900	Picrasin G	$C_{21}H_{28}O_7$	[M+Na]+	415.17272	415.17294	-0.51	2.4E+06		2.7E+06		2.6E+06			
901	Isodomedin	$C_{22}H_{32}O_6$	[M+Na]+	415.20911	415.20932	-0.51	2.4E+06							
902	EstraTriene-3,6alpha,17beta-triol triacetate	$C_{24}H_{30}O_6$	[M+H]+	415.21152	415.21157	-0.14		9.6E+07		4.1E+06		6.6E+06		
903	Dihydroxycholanoic acid	$C_{24}H_{40}O_4$	[M+Na]+	415.28188	415.28210	-0.54	9.7E+06	8.1E+06	1.0E+07		4.6E+06	3.9E+06	1.2E+07	
904	Alpinine	$C_{23}H_{29}NO_6$	[M+H]+	416.20676	416.20684	-0.18	1.8E+06							
905	Hydroxyhexadecanoylcarnitine	$C_{23}H_{45}NO_5$	[M+H]+	416.33705	416.33711	-0.14	2.2E+06		2.9E+06					
906	Tomatidine	$C_{27}H_{45}NO_2$	[M+H]+	416.35231	416.35237	-0.14			2.5E+06				2.4E+06	
907	Gibberellin A28	$C_{20}H_{26}O_8$	[M+Na]+	417.15199	417.15217	-0.44	3.3E+06							
908	Briantheine W	$C_{24}H_{32}O_6$	[M+H]+	417.22717	417.22724	-0.18		3.5E+06						
909	Hydroxyeicosatetraenoate glyceryl ester	$C_{23}H_{38}O_5$	[M+H]+	417.26115	417.26135	-0.48	3.6E+07	3.4E+06	2.6E+07		1.5E+07	3.4E+06	2.0E+07	
910	Dihydroxyvitamin D3	$C_{27}H_{44}O_3$	[M+H]+	417.33632	417.33639	-0.16		2.6E+06				3.0E+06		
911	Tetramethyl-docosenoic acid	$C_{26}H_{50}O_2$	[M+Na]+	417.37030	417.37034	-0.09				1.9E+06				
912	Cyclovirobuxine C	$C_{27}H_{48}N_2O$	[M+H]+	417.38394	417.38400	-0.13	3.0E+06				2.8E+06		2.4E+06	
913	Sphingofungin E	$C_{21}H_{39}NO_7$	[M+H]+	418.27993	418.27999	-0.15	2.2E+06							

914	N-Oleoyl Dopamine	$C_{26}H_{43}NO_3$	[M+H]+	418.33157	418.33164	-0.16	7.2E+06	2.1E+06	4.1E+06	2.4E+06		1.3E+07	5.3E+06
915	N-(hydroxy-methyl-ethyl)-dimethyl-docosatetraenoyl amine	$C_{27}H_{47}NO_2$	[M+H]+	418.36796	418.36802	-0.15						2.4E+06	
916	Melengestrol acetate	$C_{25}H_{32}O_4$	[M+Na]+	419.21928	419.21953	-0.60		2.5E+06					
917	methyl-[epidioxy-(hydroperoxy-pentenyl)-cyclopentyl]-octanoate	$C_{22}H_{36}O_6$	[M+Na]+	419.24041	419.24049	-0.18		2.5E+06				2.0E+06	

				ESI(+) FT-ICR MS										
No.	Plausible Compound[a]	Molecular formula (M)	Ion	Theor m/z[b]	Exp. m/z[c]	Δppm[d]	SM$_R$H[e]	SM$_R$O[e]	SM$_P$H[e]	SM$_P$O[e]	TF$_R$H[e]	TF$_R$O[e]	TF$_P$H[e]	TF$_P$O[e]
918	Estradiol cyclopentylpropionate	$C_{26}H_{36}O_3$	[M+Na]+	419.25567	419.25589	-0.54	2.9E+06	3.5E+06	3.4E+06			2.7E+06	3.5E+06	
919	Norcholanpentol	$C_{23}H_{40}O_5$	[M+Na]+	419.27680	419.27688	-0.20				2.6E+06	2.6E+06	4.7E+06		2.6E+06
920	N-oleoyl asparagine	$C_{22}H_{40}N_2O_4$	[M+Na]+	419.28803	419.28809	-0.15		2.0E+06		1.4E+06		3.0E+06		
921	Palmitoyl glucuronide	$C_{22}H_{42}O_7$	[M+H]+	419.30033	419.30038	-0.12	3.4E+06				2.7E+06		3.8E+06	
922	Trihydroxy-norvitamin D3	$C_{26}H_{42}O_4$	[M+H]+	419.31559	419.31566	-0.17	9.7E+06	1.1E+07	4.3E+06	1.2E+06	2.4E+06	5.9E+06	5.3E+06	2.4E+06
923	Isovitamin D2	$C_{28}H_{44}O$	[M+Na]+	419.32844	419.32862	-0.44		2.9E+06				2.7E+06		
924	Ethamoxytriphetol	$C_{27}H_{33}NO_3$	[M+H]+	420.25332	420.25331	0.02				1.6E+06		2.4E+06		5.5E+06
925	Trihydroxy-dimethoxy-prenylflavone	$C_{22}H_{22}O_7$	[M+Na]+	421.12577	421.12599	-0.51	3.8E+06							
926	C17 Sphingosine-1-phosphate	$C_{17}H_{39}N_2O_5P$	[M+K]+	421.22282	421.22314	-0.76		8.5E+06		1.4E+06		7.7E+06		
927	Pregna-triene-diol diacetate	$C_{25}H_{34}O_4$	[M+Na]+	421.23493	421.23508	-0.36		2.3E+06						
928	Dihydroxy-tetradehydro-norvitamin D3	$C_{26}H_{38}O_3$	[M+Na]+	421.27132	421.27151	-0.46	2.2E+06	2.7E+06	2.9E+06			2.8E+06	3.3E+06	
929	Hydroxy-didehydrovitamin D3	$C_{27}H_{42}O_2$	[M+Na]+	421.30770	421.30791	-0.50					2.1E+06			
930	Tetracosanedioic acid	$C_{24}H_{46}O_4$	[M+Na]+	421.32883	421.32891	-0.19					2.5E+06		6.8E+06	
931	Methylvitamin D3	$C_{28}H_{46}O$	[M+Na]+	421.34409	421.34428	-0.45	1.1E+07			6.0E+06	3.9E+06			3.5E+06
932	Fenpyroximate	$C_{24}H_{27}N_3O_4$	[M+H]+	422.20743	422.20783	-0.94							3.2E+06	
933	N-acetylsphingosine phosphate	$C_{20}H_{40}NO_6P$	[M+H]+	422.26660	422.26681	-0.50	3.7E+06		3.0E+06	3.9E+06	3.6E+06	2.0E+06	4.7E+06	
934	Ticarcillin	$C_{15}H_{16}N_2O_6S_2$	[M+K]+	423.00814	423.00829	-0.37	5.2E+06		6.2E+06		4.8E+06		6.7E+06	
935	Tetracenomycin F2	$C_{20}H_{16}O_8$	[M+K]+	423.04768	423.04758	0.23	4.3E+07		1.7E+07				1.4E+07	
936	Lophirone J	$C_{25}H_{20}O_5$	[M+Na]+	423.12029	423.12051	-0.51			1.1E+07					
937	Isopropylidenedioxy-methylprogesterone	$C_{25}H_{36}O_4$	[M+Na]+	423.25058	423.25077	-0.45			3.6E+06			2.5E+06	3.3E+06	
938	Dihydroxy-didehydro-norvitamin D3	$C_{26}H_{40}O_3$	[M+Na]+	423.28697	423.28717	-0.48	1.1E+07	9.1E+06	8.0E+06	2.4E+06	4.8E+06	8.0E+06	8.2E+06	2.2E+06
939	Diapolycopene	$C_{30}H_{40}$	[M+K]+	423.30222	423.30244	-0.52	5.5E+06	9.8E+06	1.8E+07	4.5E+06	2.7E+06	1.2E+07	1.5E+07	3.7E+06
940	Didecanoylglycerol	$C_{23}H_{44}O_5$	[M+Na]+	423.30810	423.30816	-0.16		3.4E+06				2.6E+06		2.4E+06
941	Hydroxyvitamin D3	$C_{27}H_{44}O_2$	[M+Na]+	423.32335	423.32349	-0.34	3.7E+06		4.8E+06		2.2E+06			
942	Theonellasterol B	$C_{30}H_{46}O$	[M+H]+	423.36214	423.36217	-0.06						2.1E+06		
943	Octacosenoic acid	$C_{28}H_{54}O_2$	[M+H]+	423.41966	423.41970	-0.09	6.0E+06	8.0E+06	6.9E+06	2.1E+06	2.5E+06	1.2E+07	6.8E+06	2.9E+06
944	O-Methylandrocymbine	$C_{22}H_{27}NO_5$	[M+K]+	424.15208	424.15192	0.38		2.6E+06		2.4E+06		4.0E+06		8.5E+06
945	Myxalamid B	$C_{25}H_{39}NO_3$	[M+Na]+	424.28221	424.28241	-0.46	5.1E+08	5.1E+08	3.1E+08	3.4E+08	3.0E+08	1.2E+08	4.8E+08	9.5E+07
946	Deoxythymidine 5'-diphosphate	$C_{10}H_{16}N_2O_{11}P_2$	[M+Na]+	425.01215	425.01222	-0.16							2.0E+06	

				ESI(+) FT-ICR MS												
**No.**	**Plausible Compound[a]**	**Molecular formula (M)**	**Ion**	**Theor m/z[b]**	**Exp. m/z[c]**	**Δppm[d]**	**SM_RH[e]**	**SM_RO[e]**	**SM_PH[e]**	**SM_PO[e]**	**TF_RH[e]**	**TF_RO[e]**	**TF_PH[e]**	**TF_PO[e]**
947	Hydroxy-(glucosyloxy)-anthraquinone	$C_{20}H_{18}O_9$	[M+Na]+	425.08430	425.08447	-0.40	2.2E+06		2.1E+06		2.6E+06		3.3E+06	
948	p-Coumaroyl-dihydro-dihydroxy-phenylcoumarin	$C_{24}H_{18}O_6$	[M+Na]+	425.09956	425.09976	-0.47		1.2E+07						
949	Tomentolide A	$C_{25}H_{22}O_5$	[M+Na]+	425.13594	425.13615	-0.48	2.9E+07	3.4E+06	2.3E+07	1.5E+07	2.1E+07	1.5E+07	2.1E+07	3.2E+06
950	Sphenostylin D	$C_{22}H_{26}O_7$	[M+Na]+	425.15707	425.15725	-0.41	3.3E+06		2.8E+06		3.4E+06		2.3E+06	
951	Dihydroxy-diperoxy-eicosadienoic acid	$C_{20}H_{34}O_8$	[M+Na]+	425.21459	425.21466	-0.16					1.9E+06			6.1E+06
952	Trioxo-cholan-oic Acid	$C_{24}H_{34}O_5$	[M+Na]+	425.22984	425.23006	-0.49	3.9E+06	6.7E+06	3.5E+06		3.9E+06	3.1E+06	3.9E+06	
953	Erythronolide B	$C_{21}H_{38}O_7$	[M+Na]+	425.25097	425.25107	-0.23						2.1E+06		
954	MG(0:0/22:6/0:0)	$C_{25}H_{38}O_4$	[M+Na]+	425.26623	425.26643	-0.47	4.0E+06	9.9E+06	5.3E+07	2.2E+06	5.1E+06	6.3E+06	2.3E+07	2.7E+06
955	Dihydroxy-norprevitamin D3	$C_{26}H_{42}O_3$	[M+Na]+	425.30262	425.30281	-0.46	3.7E+06	3.5E+06	4.7E+06		2.8E+06	3.8E+06	4.1E+06	
956	Diaponeurosporene	$C_{30}H_{42}$	[M+Na]+	425.31787	425.31804	-0.40	3.0E+06		2.2E+06			3.3E+06	3.0E+06	
957	Hydroxycholesterol	$C_{27}H_{46}O_2$	[M+Na]+	425.33900	425.33906	-0.13		2.9E+06				5.0E+06		3.6E+06
958	Glutinone	$C_{30}H_{48}O$	[M+H]+	425.37779	425.37784	-0.11	3.2E+06	8.9E+06	4.3E+06			7.4E+06	4.2E+06	3.4E+06
959	Octacosanoic acid	$C_{28}H_{56}O_2$	[M+H]+	425.43531	425.43535	-0.09	3.4E+06	4.3E+06	4.1E+06	1.9E+06		6.6E+06	3.5E+06	2.1E+06
960	Oleoylcarnitine	$C_{25}H_{47}NO_4$	[M+H]+	426.35779	426.35783	-0.11	3.6E+06	3.8E+06	4.8E+06		4.3E+06	2.9E+06	5.8E+06	
961	S-Glutathionyl-L-cysteine	$C_{13}H_{22}N_4O_8S_2$	[M+H]+	427.09518	427.09522	-0.10	3.2E+06		2.7E+06		3.2E+06			
962	Hexamethoxyflavanone	$C_{21}H_{24}O_8$	[M+Na]+	427.13634	427.13653	-0.44	2.9E+06		3.4E+06		3.6E+06		4.8E+06	
963	Chromolaenide	$C_{22}H_{28}O_7$	[M+Na]+	427.17272	427.17294	-0.51	3.0E+06		2.6E+06		2.7E+06			
964	Hydroxy-dioxo-cholanoic Acid	$C_{24}H_{36}O_5$	[M+Na]+	427.24549	427.24563	-0.30		2.4E+06				2.2E+06		
965	Hydroxy-didehydrovitamin D3 lactone	$C_{27}H_{38}O_4$	[M+H]+	427.28429	427.28395	0.79	9.4E+06	9.8E+06	5.7E+06	1.2E+07	9.1E+06	3.5E+06	1.3E+07	3.1E+06
966	Leupeptin	$C_{20}H_{38}N_6O_4$	[M+H]+	427.30273	427.30307	-0.80	2.7E+06	3.4E+06	3.3E+06		3.0E+06	3.6E+06	4.3E+06	
967	Dihydroxy-norsecovitamin D3	$C_{26}H_{44}O_3$	[M+Na]+	427.31827	427.31842	-0.35		3.0E+06				2.9E+06		
968	Hexacosanedioic acid	$C_{26}H_{50}O_4$	[M+H]+	427.37819	427.37821	-0.05	2.6E+06	3.2E+06				5.8E+06		1.8E+06
969	Neophoenol	$C_{30}H_{50}O$	[M+H]+	427.39344	427.39348	-0.09	7.0E+06	1.6E+07	9.4E+06	2.8E+06	3.4E+06	2.0E+07	7.7E+06	5.4E+06
970	Stearoylcarnitine	$C_{25}H_{49}NO_4$	[M+H]+	428.37344	428.37348	-0.10	2.9E+07	2.5E+07	3.7E+07	5.7E+06	1.9E+07	1.8E+07	4.4E+07	9.6E+06
971	Dopaxanthin	$C_{18}H_{18}N_2O_8$	[M+K]+	429.06947	429.06928	0.45	2.8E+06		3.5E+06		4.3E+06			
972	Hydroxy-dihydrojasmonic acid glucoside	$C_{18}H_{30}O_9$	[M+K]+	429.15214	429.15218	-0.09	2.5E+06							
973	Isomontanolide	$C_{22}H_{30}O_7$	[M+Na]+	429.18837	429.18857	-0.46	1.2E+07		8.3E+06		6.1E+06		5.5E+06	
974	Ketodeoxycholic acid	$C_{24}H_{38}O_4$	[M+K]+	429.24017	429.24021	-0.10	1.4E+08	5.6E+07	4.6E+07	1.4E+07	9.9E+07	3.7E+07	4.3E+07	1.0E+07
975	Trihydroxy-choenoic Acid	$C_{24}H_{38}O_5$	[M+Na]+	429.26115	429.26132	-0.41	1.0E+07	3.9E+06	8.0E+06		6.4E+06	3.9E+06	6.4E+06	2.6E+06

				ESI(+) FT-ICR MS										
**No.**	**Plausible Compound**[a]	**Molecular formula (M)**	**Ion**	**Theor m/z**[b]	**Exp. m/z**[c]	**Δppm**[d]	**SM_RH^e**	**SM_RO^e**	**SM_PH^e**	**SM_PO^e**	**TF_RH^e**	**TF_RO^e**	**TF_PH^e**	**TF_PO^e**
976	Dihydroxynoroxavitamin D3	$C_{25}H_{42}O_4$	[M+Na]+	429.29753	429.29772	-0.44	7.7E+06	1.2E+07	8.3E+06	4.0E+06	3.6E+06	8.6E+06	7.5E+06	
977	Diapophytofluene	$C_{30}H_{46}$	[M+Na]+	429.34917	429.34895	0.52								2.6E+06
978	Fucosterol epoxide	$C_{29}H_{48}O_2$	[M+H]+	429.37271	429.37276	-0.13	2.2E+06	3.3E+06	2.6E+06	1.3E+06		4.6E+06		1.9E+06
979	CMP-aminoethylphosphonate	$C_{11}H_{20}N_4O_{10}P_2$	[M+H]+	431.07274	431.07235	0.91					2.7E+06			
980	Ginkgolide A	$C_{20}H_{24}O_9$	[M+Na]+	431.13125	431.13144	-0.43	2.5E+06			1.0E+06				
981	Bleekerine	$C_{23}H_{24}N_2O_5$	[M+Na]+	431.15774	431.15765	0.21								2.1E+06
982	Vernoflexuoside	$C_{21}H_{28}O_8$	[M+Na]+	431.16764	431.16778	-0.33	2.7E+06							
983	PGE2 methyl ester acetate	$C_{23}H_{36}O_6$	[M+Na]+	431.24041	431.24064	-0.53	4.9E+06		3.6E+06					
984	Hydroxypregnenedione hydrogen succinate	$C_{25}H_{34}O_6$	[M+H]+	431.24282	431.24292	-0.24		4.2E+06						
985	Trihydroxy-cholanoic Acid	$C_{24}H_{40}O_5$	[M+Na]+	431.27680	431.27697	-0.41	5.7E+06	2.7E+06			3.9E+06	2.4E+06		
986	Oxo-hexacosenoic acid	$C_{26}H_{48}O_3$	[M+Na]+	431.34957	431.34963	-0.14		2.7E+06		2.6E+06				2.9E+06
987	C27-Phthienoic acid	$C_{27}H_{52}O_2$	[M+Na]+	431.38595	431.38596	-0.01				1.4E+06				2.8E+06
988	Sphingofungin D	$C_{22}H_{41}NO_7$	[M+H]+	432.29558	432.29563	-0.11	3.5E+06		2.3E+06		2.1E+06		2.5E+06	
989	Specionin	$C_{20}H_{26}O_9$	[M+Na]+	433.14690	433.14707	-0.39	3.5E+06				2.9E+06			
990	Gama-Tocotrienol	$C_{28}H_{42}O_2$	[M+Na]+	433.30770	433.30786	-0.37			2.2E+06					
991	MG(0:0/22:2/0:0)	$C_{25}H_{46}O_4$	[M+Na]+	433.32883	433.32891	-0.19		2.6E+06	2.2E+06	1.1E+06		4.7E+06		3.4E+06
992	Hexacosenoic acid	$C_{26}H_{50}O_2$	[M+K]+	433.34424	433.34427	-0.06	2.9E+06	3.9E+06	3.7E+06			5.0E+06	3.4E+06	
993	Acetophenazine	$C_{23}H_{29}N_3O_2S$	[M+Na]+	434.18727	434.18688	0.90	4.6E+06							
994	Grayanotoxin I	$C_{22}H_{36}O_7$	[M+Na]+	435.23532	435.23543	-0.23		3.6E+06				2.4E+06		
995	Oleoylglycerone phosphate	$C_{21}H_{39}O_7P$	[M+H]+	435.25062	435.25077	-0.34	3.5E+06	6.5E+06			2.8E+06			
996	Dihydroxy-cyclo-didehydroepivitamin D3	$C_{27}H_{40}O_3$	[M+Na]+	435.28697	435.28713	-0.38	3.1E+06	3.6E+06	3.8E+06			3.8E+06	3.8E+06	
997	Ficisterol	$C_{29}H_{48}O$	[M+Na]+	435.35974	435.35992	-0.41	1.1E+07	1.4E+07	1.2E+07	5.2E+06	3.8E+06	1.6E+07	1.1E+07	3.9E+06
998	Hydroxyhexacosanoic acid	$C_{26}H_{52}O_3$	[M+Na]+	435.38087	435.38092	-0.11		2.4E+06				3.8E+06		
999	N-stearoyl glutamic acid	$C_{23}H_{43}NO_5$	[M+Na]+	436.30334	436.30335	-0.01				1.1E+06				
1000	Estra-triene-triol triacetate	$C_{24}H_{30}O_6$	[M+Na]+	437.19346	437.19351	-0.12							2.3E+06	
1001	Methyl hydroperoxy-bisepidioxy-eicosadienoate	$C_{21}H_{34}O_8$	[M+Na]+	437.21459	437.21463	-0.09						2.1E+06		
1002	Lupulone	$C_{26}H_{38}O_4$	[M+Na]+	437.26623	437.26641	-0.40		2.1E+06	2.1E+06				3.0E+06	
1003	Dihydroxy-methano-norvitamin D3	$C_{27}H_{42}O_3$	[M+Na]+	437.30262	437.30280	-0.42	1.4E+07		9.9E+06	3.6E+06	5.1E+06		9.5E+06	
1004	Hydroxy-campestenone	$C_{28}H_{46}O_2$	[M+Na]+	437.33900	437.33914	-0.32		2.6E+06	2.3E+06			4.3E+06	3.0E+06	

ESI(+) FT-ICR MS

No.	Plausible Compound[a]	Molecular formula (M)	Ion	Theor m/z[b]	Exp. m/z[c]	Δppm[d]	SM$_R$H[e]	SM$_R$O[e]	SM$_P$H[e]	SM$_P$O[e]	TF$_R$H[e]	TF$_R$O[e]	TF$_P$H[e]	TF$_P$O[e]
1005	MG(22:0/0:0/0:0)	$C_{25}H_{50}O_4$	[M+Na]+	437.36013	437.36017	-0.09	2.0E+06	4.5E+06		1.9E+06		6.4E+06		3.0E+06
1006	Tridecyl oleate	$C_{29}H_{56}O_2$	[M+H]+	437.43531	437.43533	-0.06	4.8E+06	6.9E+06	5.9E+06	1.8E+06	2.4E+06	1.1E+07	6.0E+06	3.0E+06
1007	Fluphenazine	$C_{22}H_{26}F_3N_3OS$	[M+H]+	438.18214	438.18176	0.88			6.8E+06		5.7E+06		4.7E+06	
1008	Lunarine	$C_{25}H_{31}N_3O_4$	[M+H]+	438.23873	438.23912	-0.89	2.4E+07		2.2E+07	3.8E+06	2.5E+07	5.2E+06	1.8E+07	3.9E+06
1009	Erioflorin methacrylate	$C_{23}H_{28}O_7$	[M+Na]+	439.17272	439.17294	-0.49	3.0E+06	6.6E+06	5.8E+06				5.4E+06	
1010	Alfentanil	$C_{21}H_{32}N_6O_3$	[M+Na]+	439.24281	439.24246	0.81	3.1E+06				4.2E+06			
1011	Dihydroxyandrostenone dipropionate	$C_{25}H_{36}O_5$	[M+Na]+	439.24549	439.24570	-0.46	2.7E+06	3.6E+06	2.8E+06			2.6E+06	2.3E+06	
1012	Epoxy-dihydroxy-norvitamin D3	$C_{26}H_{40}O_4$	[M+Na]+	439.28188	439.28199	-0.25	3.5E+06	2.2E+06	3.1E+06				3.4E+06	
1013	Calcitriol	$C_{27}H_{44}O_3$	[M+Na]+	439.31827	439.31839	-0.27	3.2E+06		4.5E+06	3.0E+06	2.3E+06		4.9E+06	5.2E+06
1014	Beta-tocopherol	$C_{28}H_{48}O_2$	[M+Na]+	439.35465	439.35469	-0.09	5.0E+06	3.6E+07	1.6E+07	1.2E+07	2.4E+06	5.0E+07	2.2E+07	1.8E+07
1015	Pentacosanoylglycine	$C_{27}H_{53}NO_3$	[M+H]+	440.40982	440.40987	-0.11		4.4E+07		3.6E+06		5.3E+06		
1016	Hallactone B	$C_{20}H_{24}O_9S$	[M+H]+	441.12138	441.12154	-0.36					3.1E+06		2.8E+06	
1017	Syringaresinol	$C_{22}H_{26}O_8$	[M+Na]+	441.15199	441.15212	-0.30	2.2E+06							
1018	Trihydroxy-methylpregnendione acetate	$C_{24}H_{34}O_6$	[M+Na]+	441.22476	441.22493	-0.39		2.3E+06						
1019	Dihydroxy-pregnanone diacetate	$C_{25}H_{38}O_5$	[M+Na]+	441.26115	441.26132	-0.40		2.2E+06						
1020	Trifluoro-hydroxy-norvitamin D3	$C_{26}H_{39}F_3O_2$	[M+H]+	441.29749	441.29757	-0.18	8.6E+06	1.2E+07	4.3E+06	4.0E+06	2.7E+06	9.8E+06	8.4E+06	6.5E+06
1021	apo-Carotenol	$C_{30}H_{42}O$	[M+Na]+	441.31279	441.31299	-0.45		2.5E+06				3.5E+06		2.5E+06
1022	Dihomo-hydroxy epivitamin D2	$C_{30}H_{48}O_2$	[M+H]+	441.37271	441.37279	-0.19		4.2E+06						
1023	MG(0:0/24:1/0:0)	$C_{27}H_{52}O_4$	[M+H]+	441.39384	441.39392	-0.18		2.2E+06				4.7E+06		
1024	Glycyphyllin	$C_{21}H_{24}O_9$	[M+Na]+	443.13125	443.13144	-0.42							2.5E+06	
1025	Eupacunolin	$C_{22}H_{28}O_8$	[M+Na]+	443.16764	443.16784	-0.45	4.4E+06		3.4E+06		2.7E+06			
1026	Fluoro-trihydroxypregnadiendione acetate	$C_{23}H_{29}FO_6$	[M+Na]+	443.18404	443.18411	-0.15		2.6E+06				2.4E+06		
1027	PG(13:0/0:0)	$C_{19}H_{39}O_9P$	[M+H]+	443.24045	443.24062	-0.39	2.7E+06				2.6E+06			
1028	Scillirosidin	$C_{26}H_{34}O_6$	[M+H]+	443.24282	443.24293	-0.26		5.1E+06						
1029	Testosterone phenylpropionate	$C_{28}H_{36}O_3$	[M+Na]+	443.25567	443.25582	-0.35	2.9E+06							
1030	Hydroperoxy-squalene	$C_{30}H_{50}O_2$	[M+H]+	443.38836	443.38842	-0.14		4.5E+06				3.9E+06		
1031	Bis[(phenyl-propyl)phenyl]amine	$C_{30}H_{31}N$	[M+K]+	444.20881	444.20843	0.85		2.4E+06				3.3E+06		
1032	Hydroxy-octadecanoylcarnitine	$C_{25}H_{49}NO_5$	[M+H]+	444.36835	444.36841	-0.13	2.4E+06		3.5E+06			3.2E+06	3.0E+06	
1033	Enantiomultijugin	$C_{24}H_{22}O_7$	[M+Na]+	445.12577	445.12605	-0.62	5.1E+06							

			ESI(+) FT-ICR MS										
**No.**	**Plausible Compound**[a]	**Molecular formula (M)**	**Ion**	**Theor m/z**[b]	**Exp. m/z**[c]	**Δppm**[d]	**SM$_{R}$H**[e]	**SM$_{R}$O**[e]	**SM$_{P}$H**[e]	**SM$_{P}$O**[e]	**TF$_{R}$H**[e]	**TF$_{R}$O**[e]	**TF$_{P}$H**[e]	**TF$_{P}$O**[e]
1034	Octenol-3-o-D-xylopyranosyl-glucopyranoside	$C_{19}H_{34}O_{10}$	[M+Na]+	445.20442	445.20449	-0.16					3.9E+06			
1035	Trihydroxy-oxo-cholanoic Acid	$C_{24}H_{38}O_{6}$	[M+Na]+	445.25606	445.25632	-0.58					3.0E+06			
1036	Methyl-butanoyloxy-villanovanediol	$C_{25}H_{42}O_{5}$	[M+Na]+	445.29245	445.29266	-0.48	1.7E+07	3.3E+07	1.7E+07	6.6E+06	6.9E+06	1.7E+07	2.1E+07	2.8E+06
1037	Grandidentatin	$C_{21}H_{28}O_{9}$	[M+Na]+	447.16255	447.16273	-0.39	4.8E+06				3.6E+06		3.7E+06	
1038	Didrovaltratum	$C_{22}H_{32}O_{8}$	[M+Na]+	447.19894	447.19912	-0.41	4.5E+06				3.1E+06			
1039	Difenoxin	$C_{28}H_{28}N_{2}O_{2}$	[M+Na]+	447.20430	447.20408	0.49		3.8E+06						
1040	Pravastatin	$C_{23}H_{36}O_{7}$	[M+Na]+	447.23532	447.23554	-0.49	5.9E+06		5.0E+06		4.2E+06		4.6E+06	
1041	Alpha-Tocotrienol	$C_{29}H_{44}O_{2}$	[M+Na]+	447.32335	447.32359	-0.53		2.6E+06	3.3E+06			3.6E+06	3.9E+06	
1042	Trihydroxy-homovitamin D3	$C_{28}H_{46}O_{4}$	[M+H]+	447.34689	447.34698	-0.20		5.5E+06				4.0E+06		
1043	Glutinone	$C_{30}H_{48}O$	[M+Na]+	447.35974	447.35997	-0.52	2.4E+06							
1044	Tephrorin A	$C_{24}H_{26}O_{7}$	[M+Na]+	449.15707	449.15725	-0.40	5.0E+06	3.0E+06	5.1E+06		3.1E+06			
1045	Abscisic acid glucose ester	$C_{21}H_{30}O_{9}$	[M+Na]+	449.17820	449.17842	-0.47	1.1E+07		9.0E+06		9.5E+06		6.7E+06	
1046	Dihydroxy-oxo-prostadienoic acid glyceryl ester	$C_{23}H_{38}O_{7}$	[M+Na]+	449.25097	449.25113	-0.35		2.8E+06						
1047	Glutathionylaminopropylcadaverine	$C_{18}H_{36}N_{6}O_{5}S$	[M+H]+	449.25407	449.25438	-0.70						2.6E+06		
1048	Hydroxy-gama-tocotrienol	$C_{28}H_{42}O_{3}$	[M+Na]+	449.30262	449.30286	-0.53		4.7E+06	3.1E+06			5.6E+06	3.4E+06	2.3E+06
1049	Hydroxy-methylvitamin D2	$C_{29}H_{46}O_{2}$	[M+Na]+	449.33900	449.33922	-0.49			3.3E+06					
1050	Hexacosanedioic acid	$C_{26}H_{50}O_{4}$	[M+Na]+	449.36013	449.36020	-0.15				3.2E+06				
1051	Methylene-stigmastenol	$C_{30}H_{50}O$	[M+Na]+	449.37539	449.37548	-0.21					4.6E+06			
1052	tetramethyl-hexacosadienoic acid	$C_{30}H_{56}O_{2}$	[M+H]+	449.43531	449.43528	0.06						3.0E+06		
1053	Tetrahydroxyflavanone 6-C-glucoside	$C_{21}H_{22}O_{11}$	[M+H]+	451.12349	451.12359	-0.23			2.8E+06					
1054	Furcatin	$C_{20}H_{28}O_{10}$	[M+Na]+	451.15747	451.15770	-0.51	1.3E+07		1.1E+07		1.1E+07		7.5E+06	
1055	Heteroflavanone B	$C_{24}H_{28}O_{7}$	[M+Na]+	451.17272	451.17290	-0.39	1.7E+07	3.0E+06	1.2E+07		9.8E+06		7.8E+06	
1056	Hydroxyvitamin D3 lactone	$C_{27}H_{40}O_{4}$	[M+Na]+	451.28188	451.28213	-0.55		3.0E+06						
1057	Chaksine	$C_{22}H_{38}N_{6}O_{4}$	[M+H]+	451.30273	451.30311	-0.83		4.7E+06				4.2E+06		2.8E+06
1058	Dihydroxy-methyl-didehydrovitamin D3	$C_{28}H_{44}O_{3}$	[M+Na]+	451.31827	451.31846	-0.43	8.3E+06	8.9E+06	6.7E+06	4.0E+06	3.8E+06	7.4E+06	6.6E+06	
1059	Myristyl oleate	$C_{30}H_{58}O_{2}$	[M+H]+	451.45096	451.45101	-0.12	1.2E+07	1.8E+07	1.5E+07	3.8E+06	3.8E+06	3.0E+07	1.5E+07	6.7E+06
1060	Tomatidinol	$C_{27}H_{43}NO_{2}$	[M+K]+	452.29254	452.29284	-0.67				3.8E+06				
1061	N-oleoyl phenylalanine	$C_{27}H_{43}NO_{3}$	[M+Na]+	452.31351	452.31374	-0.49	7.5E+06		4.4E+06	8.9E+06	7.9E+06	2.6E+06	9.9E+06	2.6E+06
1062	Dimethyl-bis(methoxyphenyl)benzopyranol acetate	$C_{27}H_{26}O_{5}$	[M+Na]+	453.16724	453.16746	-0.48	6.3E+06	4.6E+08	9.6E+06	3.4E+07	5.0E+06	4.8E+07	6.6E+06	1.4E+07

							ESI(+) FT-ICR MS								
**No.**	**Plausible Compound[a]**	**Molecular formula (M)**	**Ion**	**Theor m/z[b]**	**Exp. m/z[c]**	**Δppm[d]**	**SM$_R$H[e]**	**SM$_R$O[e]**	**SM$_P$H[e]**	**SM$_P$O[e]**	**TF$_R$H[e]**	**TF$_R$O[e]**	**TF$_P$H[e]**	**TF$_P$O[e]**
1063	PA(18:4/0:0)	$C_{21}H_{35}O_7P$	[M+Na]+	453.20126	453.20146	-0.44	3.8E+06							
1064	Phenyl-trinor-PGF2alpha isopropyl ester	$C_{26}H_{38}O_5$	[M+Na]+	453.26115	453.26133	-0.41	7.1E+06	4.5E+06	3.8E+06		4.5E+06	2.9E+06	5.2E+06	
1065	Solagenin	$C_{27}H_{42}O_4$	[M+Na]+	453.29753	453.29764	-0.23	2.7E+06	3.6E+06	2.4E+06			2.9E+06	3.8E+06	
1066	Difluoro-dihydroxyvitamin D3	$C_{27}H_{42}F_2O_3$	[M+H]+	453.31748	453.31714	0.75					3.9E+06			
1067	Methyl-epi-dihydroxyvitamin D3	$C_{28}H_{46}O_3$	[M+Na]+	453.33392	453.33410	-0.41	4.0E+06	4.7E+06	5.1E+06				4.4E+06	2.4E+06
1068	Alpha-Tocopherol	$C_{29}H_{50}O_2$	[M+Na]+	453.37030	453.37035	-0.10		5.7E+06	7.7E+06			1.8E+07		
1069	Tetradecyl-hexadecanoate	$C_{30}H_{60}O_2$	[M+H]+	453.46661	453.46664	-0.08	4.9E+06	5.9E+06	5.4E+06	1.6E+06		9.4E+06	4.8E+06	3.4E+06
1070	Sphingofungin D	$C_{22}H_{41}NO_7$	[M+Na]+	454.27752	454.27756	-0.08								2.4E+06
1071	Spironolactone	$C_{24}H_{32}O_4S$	[M+K]+	455.16529	455.16558	-0.64		3.4E+07						
1072	Alfentanil	$C_{21}H_{32}N_6O_3$	[M+H]+	455.21675	455.21648	0.59					4.0E+06			
1073	Diaponeurosporenic acid	$C_{30}H_{40}O_2$	[M+Na]+	455.29205	455.29226	-0.46	4.5E+06	5.6E+06	4.5E+06			3.8E+06	5.4E+06	
1074	Trifluoro-hydroxyvitamin D3	$C_{27}H_{41}F_3O_2$	[M+Na]+	455.31314	455.31323	-0.20		4.4E+06				3.9E+06		2.9E+06
1075	Hydroxy-gama-tocopherol	$C_{28}H_{48}O_3$	[M+Na]+	455.34957	455.34962	-0.13		9.2E+06	3.8E+06	3.0E+06		1.0E+07	7.0E+06	5.4E+06
1076	Pelargonidin 3-O-glucoside	$C_{21}H_{21}O_{10}$	[M+Na]+	456.10269	456.10253	0.36	4.8E+06		3.9E+06		6.4E+06			
1077	Arachidyl carnitine	$C_{27}H_{53}NO_4$	[M+H]+	456.40474	456.40480	-0.13			2.6E+06				2.6E+06	
1078	Heptamethoxyflavanone	$C_{22}H_{26}O_9$	[M+Na]+	457.14690	457.14715	-0.54	2.6E+06						2.4E+06	
1079	(Diphenylethenylidene)bis[N,N-dimethylbenzenamine]	$C_{30}H_{30}N_2$	[M+K]+	457.20406	457.20448	-0.91	2.8E+06				3.5E+06			
1080	Trihydroxy-oxavitamin D3	$C_{26}H_{42}O_5$	[M+Na]+	457.29245	457.29264	-0.43	2.5E+06							
1081	Trihydroxy-dihydrovitamin D3	$C_{27}H_{46}O_4$	[M+Na]+	457.32883	457.32892	-0.20		4.1E+06				4.4E+06		
1082	Salvisyriacolide	$C_{25}H_{40}O_6$	[M+Na]+	459.27171	459.27192	-0.45	2.4E+07		1.4E+07		1.2E+07		1.1E+07	
1083	Varanic acid	$C_{26}H_{44}O_5$	[M+Na]+	459.30810	459.30830	-0.43	1.4E+07	1.2E+07	1.4E+07	4.7E+06	6.3E+06	5.7E+06	1.6E+07	3.1E+06
1084	Cholestane-tetrol	$C_{27}H_{48}O_4$	[M+Na]+	459.34448	459.34449	-0.02						3.4E+06		
1085	Tetramethy-tetracosadienoic acid	$C_{28}H_{52}O_2$	[M+K]+	459.35989	459.35987	0.04							2.4E+06	
1086	Oxo-octacosenoic acid	$C_{28}H_{52}O_3$	[M+Na]+	459.38087	459.38091	-0.10		2.3E+06						
1087	Militarinone A	$C_{26}H_{37}NO_6$	[M+H]+	460.26936	460.26943	-0.14		5.4E+06						
1088	(Methyl-butanoyloxy)-villanovanediol	$C_{25}H_{42}O_6$	[M+Na]+	461.28736	461.28755	-0.42	5.3E+07	4.5E+06	3.5E+07		2.3E+07		2.6E+07	
1089	Nor-cholestanpentol	$C_{26}H_{46}O_5$	[M+Na]+	461.32375	461.32387	-0.27		2.7E+06						
1090	Ophirasterol	$C_{31}H_{50}O$	[M+Na]+	461.37539	461.37556	-0.38	6.9E+06	1.7E+07	1.3E+07	3.7E+06		1.6E+07	1.3E+07	4.1E+06

No.	Plausible Compound[a]	Molecular formula (M)	Ion	Theor m/z[b]	Exp. m/z[c]	Δppm[d]	SM_RH[e]	SM_RO[e]	SM_PH[e]	SM_PO[e]	TF_RH[e]	TF_RO[e]	TF_PH[e]	TF_PO[e]
				ESI(+) FT-ICR MS										
1091	Carboxy-gama-tocotrienol	$C_{28}H_{39}O_4$	[M+Na]+	462.27406	462.27381	0.53								3.0E+06
1092	D-Glucosylsphingosine	$C_{24}H_{47}NO_7$	[M+H]+	462.34253	462.34258	-0.12		3.7E+06	2.7E+06			3.1E+06	2.5E+06	
1093	Amurensisin	$C_{22}H_{16}O_{10}$	[M+Na]+	463.06357	463.06366	-0.19	1.2E+07		1.1E+07		1.1E+07		4.2E+06	
1094	Deacetyl-debenzoylbaccatin III	$C_{22}H_{32}O_9$	[M+Na]+	463.19385	463.19397	-0.24	4.4E+06				4.8E+06			
1095	Cyclopropyl-dihydroxy-didehydro-norvitamin D3	$C_{29}H_{44}O_3$	[M+Na]+	463.31827	463.31845	-0.41	8.6E+06	9.2E+06	8.1E+06	4.3E+06	2.6E+06	6.5E+06	8.1E+06	2.6E+06
1096	Dihomo-hydroxy-epivitamin D2	$C_{30}H_{48}O_2$	[M+Na]+	463.35465	463.35481	-0.34			3.7E+06					
1097	MG(24:1/0:0/0:0)	$C_{27}H_{52}O_4$	[M+Na]+	463.37578	463.37582	-0.08			3.8E+06	4.1E+06			5.0E+06	
1098	Methylcycloartaenol	$C_{31}H_{52}O$	[M+Na]+	463.39104	463.39122	-0.39	6.1E+06	1.1E+07	8.7E+06	3.3E+06	2.8E+06	1.1E+07	7.3E+06	3.3E+06
1099	Adenylsuccinic acid	$C_{14}H_{18}N_5O_{11}P$	[M+H]+	464.08132	464.08093	0.84					3.2E+06			
1100	D-Glucosyldihydrosphingosine	$C_{24}H_{49}NO_7$	[M+H]+	464.35818	464.35820	-0.03		3.1E+06	3.0E+06			2.8E+06	2.9E+06	
1101	Hydroxy-tetramethoxy-prenyloxyflavone	$C_{24}H_{26}O_8$	[M+Na]+	465.15199	465.15219	-0.42	5.2E+06		4.9E+06		4.2E+06		3.3E+06	
1102	Lusitanicoside	$C_{21}H_{30}O_{10}$	[M+Na]+	465.17312	465.17332	-0.43	8.3E+06		6.4E+06		5.9E+06		5.5E+06	
1103	Psychotrine	$C_{28}H_{36}N_2O_4$	[M+H]+	465.27478	465.27483	-0.10	4.8E+06		6.1E+06					
1104	Dihydroxy-dimethyl-didehydrovitamin D3	$C_{29}H_{46}O_3$	[M+Na]+	465.33392	465.33411	-0.41	1.3E+07	1.1E+07	9.3E+06	4.7E+06	5.0E+06	9.6E+06	8.7E+06	3.0E+06
1105	Sphingosine-1-phosphocholine	$C_{23}H_{49}N_2O_5P$	[M+H]+	465.34519	465.34535	-0.35	4.3E+06			4.2E+06				
1106	Hopendiol	$C_{30}H_{50}O_2$	[M+Na]+	465.37030	465.37034	-0.09	6.3E+06	3.9E+07	9.4E+06	7.6E+06	3.5E+06		1.6E+07	1.5E+07
1107	MG(24:0/0:0/0:0)	$C_{27}H_{54}O_4$	[M+Na]+	465.39143	465.39147	-0.08		4.9E+06		2.2E+06		7.0E+06	3.5E+06	3.8E+06
1108	Pentadecyl oleate	$C_{31}H_{60}O_2$	[M+H]+	465.46661	465.46663	-0.04	1.1E+07	1.6E+07	1.4E+07	3.6E+06	4.6E+06	2.7E+07	1.3E+07	6.2E+06
1109	Codonocarpine	$C_{26}H_{31}N_3O_5$	[M+H]+	466.23365	466.23404	-0.84					3.7E+06			
1110	Sphingosyl-phosphocholine	$C_{23}H_{50}N_2O_5P$	[M+H]+	466.35301	466.35271	0.65	1.4E+07	2.9E+07	2.3E+07	6.8E+06	4.2E+06	2.0E+07	2.7E+07	6.3E+06
1111	Dihydrophaseic acid O-beta-D-glucoside	$C_{21}H_{32}O_{10}$	[M+Na]+	467.18877	467.18896	-0.41	6.5E+07		5.0E+07		3.9E+07		3.2E+07	
1112	Dehydro-clavulone	$C_{25}H_{32}O_7$	[M+Na]+	467.20402	467.20421	-0.40		2.8E+06						
1113	Bufotalin	$C_{26}H_{36}O_6$	[M+Na]+	467.24041	467.24055	-0.30		2.5E+06						
1114	Dihydroxyvitamin D3 lactone	$C_{27}H_{40}O_5$	[M+Na]+	467.27680	467.27697	-0.37	4.4E+06	3.6E+06	4.6E+06			3.6E+06	3.9E+06	
1115	Trihydroxyvitamin D2	$C_{28}H_{44}O_4$	[M+Na]+	467.31318	467.31332	-0.30	3.0E+06							
1116	Ethyl-dihydroxyvitamin D3	$C_{29}H_{48}O_3$	[M+Na]+	467.34957	467.34972	-0.33	3.5E+06	3.3E+06	4.2E+06			5.3E+06	3.9E+06	
1117	Triacontatetraenoic acid	$C_{30}H_{52}O_2$	[M+Na]+	467.38595	467.38599	-0.09		6.6E+06	2.6E+06			5.6E+06	3.3E+06	
1118	Dynorphin A	$C_{18}H_{39}N_9O_4$	[M+Na]+	468.30172	468.30139	0.71		2.8E+06						
1119	Catechinol O-beta-D-galactopyranoside	$C_{21}H_{24}O_{12}$	[M+H]+	469.13405	469.13411	-0.12	5.0E+06		4.3E+06		6.9E+06			

				ESI(+) FT-ICR MS										
**No.**	**Plausible Compound**[a]	**Molecular formula (M)**	**Ion**	**Theor m/z**[b]	**Exp. m/z**[c]	**Δppm**[d]	**SM$_R$H**[e]	**SM$_R$O**[e]	**SM$_P$H**[e]	**SM$_P$O**[e]	**TF$_R$H**[e]	**TF$_R$O**[e]	**TF$_P$H**[e]	**TF$_P$O**[e]
1120	Ptilosteroid A	$C_{21}H_{34}O_8S$	[M+Na]+	469.18666	469.18706	-0.85	6.2E+06		5.1E+06		5.1E+06			
1121	PG(15:1(9Z)/0:0)	$C_{21}H_{41}O_9P$	[M+H]+	469.25610	469.25624	-0.30	3.2E+06	3.8E+06				2.9E+06		
1122	Trifluoro-dihydroxy-didehydrovitamin D3	$C_{27}H_{39}F_3O_3$	[M+H]+	469.29241	469.29263	-0.48		2.8E+06						
1123	Trihydroxy-homovitamin D3	$C_{28}H_{46}O_4$	[M+Na]+	469.32883	469.32891	-0.17				2.3E+06				
1124	Hydroxy-tocopherol [primary alcohol] ([M+Na]+)	$C_{29}H_{50}O_3$	[M+Na]+	469.36522	469.36526	-0.10		3.2E+06		2.2E+06		7.0E+06		3.4E+06
1125	Kalbreclasine	$C_{20}H_{23}NO_{12}$	[M+H]+	470.12930	470.12932	-0.05	3.5E+06		5.3E+06		3.2E+06			
1126	PS(14:0/0:0)	$C_{20}H_{40}NO_9P$	[M+H]+	470.25134	470.25150	-0.32			3.9E+06	7.6E+06	5.3E+06		7.1E+06	
1127	Diiodo-hydroxyphenylpyruvate	$C_9H_6I_2O_4$	[M+K]+	470.79871	470.79880	-0.18	2.9E+06				4.7E+06			4.4E+06
1128	Temocaprilat	$C_{21}H_{24}N_2O_5S_2$	[M+Na]+	471.10188	471.10218	-0.62		4.4E+06		5.3E+06				
1129	Artonin P	$C_{25}H_{20}O_8$	[M+Na]+	471.10504	471.10522	-0.38				3.6E+07				5.0E+07
1130	D-Galactosyl-3-(N-acetyl-beta-D-galactosaminyl)-L-serine	$C_{17}H_{30}N_2O_{13}$	[M+H]+	471.18207	471.18207	-0.02	4.0E+06		5.3E+06		2.7E+06			
1131	Usambarensine	$C_{29}H_{28}N_4$	[M+K]+	471.19456	471.19425	0.66				1.3E+06				6.4E+06
1132	Estradiol glucuronide	$C_{24}H_{32}O_8$	[M+Na]+	471.19894	471.19913	-0.40	5.4E+06	5.0E+06	4.2E+06		3.9E+06		5.8E+06	
1133	Stigmatellin X	$C_{28}H_{38}O_6$	[M+H]+	471.27412	471.27421	-0.20		3.7E+06						
1134	Trihydroxyvitamin D3	$C_{27}H_{44}O_4$	[M+K]+	471.28712	471.28708	0.08			4.2E+06					
1135	Trifluoro-dihydroxyvitamin D3	$C_{27}H_{41}F_3O_3$	[M+H]+	471.30806	471.30814	-0.17		4.2E+06	2.6E+06			3.2E+06		2.9E+06
1136	Hydroxy-gama-tocopherol	$C_{28}H_{48}O_3$	[M+K]+	471.32350	471.32349	0.03	3.3E+06							
1137	Chlorogenin	$C_{28}H_{48}O_4$	[M+Na]+	471.34448	471.34453	-0.10		3.8E+06				4.8E+06		
1138	Tetramethyl-hexacosadienoic acid	$C_{30}H_{56}O_2$	[M+Na]+	471.41725	471.41734	-0.18		5.6E+06		1.2E+06				2.8E+06
1139	Glucolesquerellin	$C_{14}H_{27}NO_9S_3$	[M+Na]+	472.07402	472.07360	0.88			3.7E+06					
1140	Nigakilactone E	$C_{24}H_{34}O_8$	[M+Na]+	473.21459	473.21476	-0.37	1.5E+07		1.1E+07		1.0E+07		7.2E+06	
1141	Laserpitin	$C_{25}H_{38}O_7$	[M+Na]+	473.25097	473.25118	-0.43	3.0E+06							
1142	Trihydroxy-cholestanoic acid	$C_{27}H_{46}O_5$	[M+Na]+	473.32375	473.32395	-0.44	8.9E+06	1.5E+07	9.7E+06	4.1E+06	4.2E+06	9.2E+06	9.5E+06	
1143	Dihydroxy-dimethylhexadehydro-homovitamin D3	$C_{30}H_{44}O_3$	[M+Na]+	475.31827	475.31853	-0.55	1.1E+07	1.2E+07	4.5E+06			5.7E+07	7.6E+06	1.1E+07
1144	Phylloquinol	$C_{31}H_{48}O_2$	[M+Na]+	475.35465	475.35488	-0.48			3.0E+06					
1145	Tetrahydrogeranylgeranyl diphosphate	$C_{20}H_{40}O_7P_2$	[M+Na]+	477.21415	477.21454	-0.81			2.8E+06				7.0E+06	
1146	Dihydroxy-dimethyl-tetradehydro-	$C_{30}H_{46}O_3$	[M+Na]+	477.33392	477.33409	-0.37	2.7E+06	3.6E+06				2.9E+06		

	homovitamin D3						
1147	Vitamin D3 butyrate	$C_{31}H_{50}O_2$	[M+Na]+	477.37030	477.37050	-0.42	3.3E+06

				ESI(+) FT-ICR MS										
**No.**	**Plausible Compound[a]**	**Molecular formula (M)**	**Ion**	**Theor m/z[b]**	**Exp. m/z[c]**	**Δppm[d]**	**SM$_R$H[e]**	**SM$_R$O[e]**	**SM$_P$H[e]**	**SM$_P$O[e]**	**TF$_R$H[e]**	**TF$_R$O[e]**	**TF$_P$H[e]**	**TF$_P$O[e]**
1148	Ethyl-trimethyl-hydroxycholestadiene	$C_{32}H_{54}O$	[M+Na]+	477.40669	477.40688	-0.41	4.3E+06	1.0E+07	7.3E+06	3.1E+06		9.4E+06	6.9E+06	3.0E+06
1149	PS(13:0/0:0)	$C_{19}H_{38}NO_9P$	[M+Na]+	478.21764	478.21766	-0.03	2.8E+06						2.5E+06	
1150	Abyssinoflavanone V	$C_{25}H_{28}O_7$	[M+K]+	479.14666	479.14709	-0.89								9.4E+06
1151	Minabeolide-7	$C_{28}H_{40}O_5$	[M+Na]+	479.27680	479.27698	-0.39	2.7E+06	1.2E+07	2.8E+06	2.9E+06				
1152	Trihydroxy-tetradehydrodihomoepivitamin D3	$C_{29}H_{44}O_4$	[M+Na]+	479.31318	479.31337	-0.40		3.2E+06						
1153	Butyl-dihydroxy-didehydro-norvitamin D3	$C_{30}H_{48}O_3$	[M+Na]+	479.34957	479.34975	-0.38	9.0E+06		8.6E+06	4.8E+06	3.5E+06	9.2E+06	8.9E+06	2.8E+06
1154	Palmityl oleate	$C_{32}H_{62}O_2$	[M+H]+	479.48226	479.48230	-0.09	1.6E+07	2.5E+07	2.0E+07	5.0E+06	5.3E+06	4.4E+07	1.9E+07	9.0E+06
1155	Hexahydroxyflavone 3-glucoside	$C_{21}H_{20}O_{13}$	[M+H]+	481.09767	481.09775	-0.17	3.8E+06				3.8E+06			
1156	3-Sulfodeoxycholic acid	$C_{23}H_{38}O_7S$	[M+Na]+	481.22305	481.22325	-0.43		1.1E+07	2.9E+06			6.9E+06		
1157	PA(10:0/10:0)	$C_{23}H_{45}O_8P$	[M+H]+	481.29248	481.29267	-0.40	4.1E+06	4.7E+06	3.6E+06	2.8E+06		4.7E+06	3.2E+06	
1158	Dihydroxy-trihomovitamin D3	$C_{30}H_{50}O_3$	[M+Na]+	481.36522	481.36535	-0.27	4.9E+06		7.4E+06	5.4E+06	3.1E+06	1.4E+07	1.0E+07	9.0E+06
1159	Hexadecyl hexadecanoate	$C_{32}H_{64}O_2$	[M+H]+	481.49791	481.49794	-0.07	5.2E+06	6.5E+06	5.9E+06	2.0E+06	2.9E+06	1.2E+07	5.7E+06	3.6E+06
1160	Cer(d18:1/12:0)	$C_{30}H_{59}NO_3$	[M+H]+	482.45677	482.45680	-0.06		3.9E+06				2.6E+06		
1161	6-Acetylpicropolin	$C_{24}H_{28}O_9$	[M+Na]+	483.16255	483.16277	-0.45	4.6E+06		3.4E+06		4.0E+06			
1162	Dihydroxypregntrione(hydrogensuccinate)	$C_{25}H_{32}O_8$	[M+Na]+	483.19894	483.19924	-0.62		3.4E+06						
1163	Hydrocortisone caproate	$C_{27}H_{40}O_6$	[M+Na]+	483.27171	483.27190	-0.39	4.1E+06	6.8E+06	3.8E+06		2.8E+06	4.5E+06	3.3E+06	
1164	Thiaclycobenzylnordihomosecocholestatrienetriol	$C_{30}H_{42}O_3S$	[M+H]+	483.29274	483.29287	-0.26			3.0E+06					
1165	Dihydroxyvitamin D3	$C_{29}H_{48}O_3$	[M+K]+	483.32350	483.32339	0.24								1.6E+07
1166	Ethylthornasterol	$C_{29}H_{48}O_4$	[M+Na]+	483.34448	483.34443	0.10						3.0E+06		
1167	Myrrhanol A	$C_{30}H_{52}O_3$	[M+Na]+	483.38087	483.38090	-0.08		8.2E+06		1.7E+06		4.6E+06		3.2E+06
1168	Triacontanedioic acid	$C_{30}H_{58}O_4$	[M+H]+	483.44079	483.44086	-0.15						3.5E+06		
1169	Tetrahydrofolic acid	$C_{19}H_{23}N_7O_6$	[M+K]+	484.13414	484.13383	0.64	4.6E+06		8.6E+06		6.0E+06		3.4E+06	
1170	Dynorphin A (6-8)	$C_{18}H_{39}N_9O_4$	[M+K]+	484.27566	484.27532	0.70	2.7E+06							
1171	Uridine triphosphate	$C_9H_{15}N_2O_{15}P_3$	[M+H]+	484.97580	484.97557	0.48							3.4E+06	
1172	Digalloyl-beta-D-glucose	$C_{20}H_{20}O_{14}$	[M+H]+	485.09258	485.09266	-0.15			3.4E+06		3.7E+06			
1173	Diospyrin	$C_{21}H_{24}O_{13}$	[M+H]+	485.12897	485.12896	0.02	3.1E+06				4.3E+06			
1174	Dihydroxy-dimethoxychalcone glucoside	$C_{23}H_{26}O_{10}$	[M+Na]+	485.14182	485.14201	-0.39	3.4E+06				3.5E+06			
1175	Dehydrotestosterone glucuronide	$C_{25}H_{34}O_8$	[M+Na]+	485.21459	485.21474	-0.30	4.1E+06	7.5E+06	4.4E+06				5.5E+06	
1176	Dehydroecdysone	$C_{27}H_{42}O_6$	[M+Na]+	485.28736	485.28749	-0.28	3.1E+06	4.0E+06				3.4E+06		

				ESI(+) FT-ICR MS									
**No.**	**Plausible Compound**[a]	**Molecular formula (M)**	**Ion**	**Theor m/z**[b]	**Exp. m/z**[c]	**Δppm**[d]	**SM$_R$H**[e]	**SM$_R$O**[e]	**SM$_P$H**[e]	**SM$_P$O**[e]	**TF$_R$H**[e]	**TF$_R$O**[e]	**TF$_P$H**[e]	**TF$_P$O**[e]
1177	Hydroxy-alpha-tocopherol	$C_{29}H_{50}O_3$	[M+K]+	485.33915	485.33925	-0.20			3.6E+06				3.4E+06	
1178	N-tert-Butyloxycarbonyl-deacetyl-leupeptin	$C_{23}H_{44}N_6O_5$	[M+H]+	485.34460	485.34493	-0.68					2.8E+06	4.1E+06		
1179	(Hydroxypropyl)-dihydroxy-norvitamin D3	$C_{29}H_{50}O_4$	[M+Na]+	485.36013	485.36024	-0.23	4.2E+06		4.2E+06		4.3E+06		3.6E+06	
1180	Methylthioheptyl glucosinolate	$C_{15}H_{29}NO_9S_3$	[M+Na]+	486.08967	486.08932	0.71							4.4E+06	
1181	D-Glucosyldihydrosphingosine	$C_{24}H_{49}NO_7$	[M+Na]+	486.34012	486.34032	-0.40					3.9E+06			
1182	hydroxynorandrostene-dione glucuronide	$C_{24}H_{32}O_9$	[M+Na]+	487.19385	487.19405	-0.41	5.4E+06		5.1E+06		3.7E+06			
1183	N-Acetyl-leu-leu-tyr-amide	$C_{23}H_{36}N_4O_5$	[M+K]+	487.23173	487.23127	-0.94		1.4E+07		4.7E+06			8.8E+06	
1184	Tetrahydroxy-cholestenoic acid	$C_{27}H_{44}O_6$	[M+Na]+	487.30301	487.30317	-0.32	5.4E+06	5.0E+06	4.3E+06			3.3E+06	5.2E+06	
1185	Hydroxy-[(hydroxy-methylethyl)phenyl]-pentanorvitamin D3	$C_{31}H_{44}O_3$	[M+Na]+	487.31827	487.31845	-0.37		3.1E+06				3.0E+06		
1186	Trihydroxycoprostanoic acid	$C_{28}H_{48}O_5$	[M+Na]+	487.33940	487.33957	-0.36	6.6E+06	7.6E+06	7.5E+06		3.7E+06	4.8E+06	5.8E+06	
1187	Tetramethyl-hexacosadienoic acid	$C_{30}H_{56}O_2$	[M+K]+	487.39119	487.39124	-0.10			4.0E+06					
1188	Hydroxycyanidin glucoside	$C_{21}H_{21}O_{12}$	[M+Na]+	488.09252	488.09240	0.25					3.3E+06			
1189	Eriotriochin	$C_{27}H_{30}O_7$	[M+Na]+	489.18837	489.18858	-0.42	4.7E+06							
1190	Hydroxy-androstane glucuronide	$C_{25}H_{38}O_8$	[M+Na]+	489.24589	489.24599	-0.21	3.3E+06							
1191	Difluoro-dihydroxy-homovitamin D3	$C_{28}H_{44}F_2O_3$	[M+Na]+	489.31507	489.31524	-0.34						4.3E+06		
1192	Tetrahydroxy-cholestanoic acid	$C_{27}H_{46}O_6$	[M+Na]+	489.31866	489.31886	-0.41	2.7E+07	4.8E+07	2.6E+07	1.1E+07	1.3E+07	2.5E+07	3.1E+07	4.4E+06
1193	Hydroxy-castasterone	$C_{28}H_{50}O_5$	[M+Na]+	489.35505	489.35496	0.17						3.5E+06		
1194	Paucin	$C_{23}H_{32}O_{10}$	[M+Na]+	491.18877	491.18896	-0.40	5.1E+06		6.0E+06		5.0E+06		4.7E+06	
1195	Diphenoxylate	$C_{30}H_{32}N_2O_2$	[M+K]+	491.20954	491.20995	-0.84					3.4E+06			
1196	Repaglinide	$C_{27}H_{36}N_2O_4$	[M+K]+	491.23067	491.23020	0.95	1.3E+07		9.3E+06		8.4E+06		2.1E+07	
1197	Hydroxyandrostane-3-glucuronide	$C_{25}H_{40}O_8$	[M+Na]+	491.26154	491.26173	-0.38	1.4E+07		9.9E+06		8.0E+06		7.3E+06	
1198	(Hydroxy-propynyl)-didehydrovitamin D3	$C_{30}H_{44}O_4$	[M+Na]+	491.31318	491.31336	-0.37						3.2E+06		
1199	Dihydroxy-dimethyl-tetradehydro-dihomovitamin D3	$C_{31}H_{48}O_3$	[M+Na]+	491.34957	491.34969	-0.25		3.4E+06	3.9E+06			3.8E+06	3.6E+06	
1200	Limonin	$C_{26}H_{30}O_8$	[M+Na]+	493.18329	493.18345	-0.33	8.7E+06		8.9E+06		5.6E+06		5.7E+06	
1201	Mucronine B	$C_{28}H_{36}N_4O_4$	[M+H]+	493.28093	493.28056	0.75		4.6E+06				4.2E+06		
1202	Trihydroxy-dimethyl-tetradehydro-homo-epivitamin D3	$C_{30}H_{46}O_4$	[M+Na]+	493.32883	493.32902	-0.38		3.1E+06						
1203	N-stearoyl tryptophan	$C_{29}H_{46}N_2O_3$	[M+Na]+	493.34006	493.33976	0.62								3.5E+06

				ESI(+) FT-ICR MS									
**No.**	**Plausible Compound**[a]	**Molecular formula (M)**	**Ion**	**Theor m/z**[b]	**Exp. m/z**[c]	**Δppm**[d]	**SM$_R$H**[e]	**SM$_R$O**[e]	**SM$_P$H**[e]	**SM$_P$O**[e]	**TF$_R$H**[e]	**TF$_R$O**[e]	**TF$_P$H**[e]	**TF$_P$O**[e]
1204	Trioctanoylglycerol	$C_{27}H_{50}O_6$	[M+Na]+	493.34996	493.35000	-0.08		4.9E+06				4.3E+06		3.5E+06
1205	(Carboxylpropyl)vitamin D3	$C_{31}H_{50}O_3$	[M+Na]+	493.36522	493.36537	-0.31	8.2E+06	8.2E+06	8.3E+06		3.3E+06	8.5E+06	7.2E+06	2.9E+06
1206	Heptadecyl oleate	$C_{33}H_{64}O_2$	[M+H]+	493.49791	493.49792	-0.03	1.9E+07	2.4E+07	2.3E+07	5.3E+06	7.5E+06	5.0E+07	2.2E+07	1.1E+07
1207	Pentahydroxy-dimethoxyflavone L-arabinopyranoside	$C_{22}H_{22}O_{13}$	[M+H]+	495.11332	495.11337	-0.11	9.6E+06		3.6E+06		1.0E+07			
1208	Deoxylimonate	$C_{26}H_{32}O_8$	[M+Na]+	495.19894	495.19914	-0.41	3.0E+07		2.1E+07		1.4E+07		1.4E+07	
1209	Oxolycoclavanol	$C_{30}H_{48}O_4$	[M+Na]+	495.34448	495.34459	-0.23		4.5E+06	2.9E+06			4.6E+06	4.4E+06	
1210	Dihydroxy-dimethyl-dihomovitamin D3	$C_{31}H_{52}O_3$	[M+Na]+	495.38087	495.38098	-0.24	4.1E+06	8.7E+06	6.1E+06	3.4E+06		9.5E+06	6.4E+06	6.2E+06
1211	Heptadecyl-palmitate	$C_{33}H_{66}O_2$	[M+H]+	495.51356	495.51359	-0.06	5.9E+06	6.8E+06	6.4E+06	2.3E+06		1.4E+07	6.5E+06	4.1E+06
1212	PG(17:1/0:0)	$C_{23}H_{45}O_9P$	[M+H]+	497.28740	497.28753	-0.26	1.1E+07	8.2E+06	7.3E+06	4.3E+06	6.1E+06	5.7E+06	6.3E+06	
1213	Dihydroxy-methoxy-dimethylvitamin D3	$C_{30}H_{50}O_4$	[M+Na]+	497.36013	497.36014	-0.01			4.3E+06			1.2E+07	8.9E+06	8.8E+06
1214	Deoxytubulosine	$C_{29}H_{37}N_3O_2$	[M+K]+	498.25174	498.25206	-0.65			2.7E+06					
1215	Calofloride	$C_{30}H_{36}O_5$	[M+Na]+	499.24549	499.24571	-0.43			3.5E+06					
1216	PG(17:0/0:0)	$C_{23}H_{47}O_9P$	[M+H]+	499.30305	499.30320	-0.31			3.1E+06					
1217	Cucurbitacin S	$C_{30}H_{42}O_6$	[M+H]+	499.30542	499.30552	-0.20		5.1E+06		5.2E+06				
1218	Dammarenetriol	$C_{30}H_{52}O_3$	[M+K]+	499.35480	499.35485	-0.09			3.9E+06					
1219	D-glucopyranosyl-eicosandiol	$C_{26}H_{52}O_7$	[M+Na]+	499.36052	499.36056	-0.07						3.2E+06		
1220	Methoxy-estradiol glucuronide	$C_{25}H_{34}O_9$	[M+Na]+	501.20950	501.20967	-0.33	5.0E+06				4.5E+06			
1221	O-alpha- glucopyranosyl nonadecandiol	$C_{25}H_{50}O_7$	[M+K]+	501.31881	501.31880	0.03		3.6E+06						
1222	Dihydroxy-(hydroxypropoxy)-norvitamin D3	$C_{29}H_{50}O_5$	[M+Na]+	501.35505	501.35517	-0.25		3.4E+06			3.3E+06			
1223	Hydroxyglucobrassicin	$C_{16}H_{19}N_2O_{10}S_2$	[M+K]+	502.01127	502.01121	0.12				1.2E+07				1.8E+07
1224	Acevaltrate	$C_{24}H_{32}O_{10}$	[M+K]+	503.18877	503.18891	-0.28	2.9E+06							
1225	Hydroxyecdysone	$C_{27}H_{44}O_7$	[M+Na]+	503.29792	503.29810	-0.35	3.6E+07	4.1E+06	2.3E+07		1.6E+07	3.5E+06	1.7E+07	
1226	epi-Brassinolide	$C_{28}H_{48}O_6$	[M+Na]+	503.33431	503.33450	-0.38	1.7E+07	1.4E+07	1.8E+07	4.3E+06	9.2E+06	7.1E+06	1.8E+07	4.4E+06
1227	Dihydroxy-dimethyl-hexadehydro-trihomovitamin D3	$C_{32}H_{48}O_3$	[M+Na]+	503.34957	503.34973	-0.32			3.8E+06			4.2E+06	3.7E+06	
1228	Pederin	$C_{25}H_{45}NO_9$	[M+H]+	504.31671	504.31652	0.37				4.7E+06	2.8E+06			3.4E+06
1229	Thymidine 5'-triphosphate	$C_{10}H_{17}N_2O_{14}P_3$	[M+Na]+	504.97848	504.97825	0.46					3.9E+06			
1230	Hydroxycholesterol sulfate	$C_{27}H_{46}O_5S$	[M+Na]+	505.29582	505.29616	-0.68	3.5E+06							
1231	Dihydroxy-diethyl-tetradehydro-homovitamin	$C_{32}H_{50}O_3$	[M+Na]+	505.36522	505.36540	-0.36		3.2E+06	3.3E+06				3.6E+06	

D3

		ESI(+) FT-ICR MS													
**No.**	**Plausible Compound**[a]	**Molecular formula (M)**	**Ion**	**Theor m/z**[b]	**Exp. m/z**[c]	**Δppm**[d]	**SM_RH^e**	**SM_RO^e**	**SM_PH^e**	**SM_PO^e**	**TF_RH^e**	**TF_RO^e**	**TF_PH^e**	**TF_PO^e**
1232	Triacontanedioic acid	$C_{30}H_{58}O_4$	[M+Na]+	505.42273	505.42280	-0.13				2.3E+06				4.5E+06
1233	Hydroxyandrostane-glucuronide	$C_{25}H_{40}O_9$	[M+Na]+	507.25645	507.25648	-0.05	3.9E+06				3.6E+06			
1234	Cholestan heptol	$C_{27}H_{48}O_7$	[M+Na]+	507.32922	507.32919	0.07		6.4E+06						
1235	Stearyl oleate	$C_{34}H_{66}O_2$	[M+H]+	507.51356	507.51358	-0.05	2.0E+07	2.7E+07	2.4E+07	5.1E+06	6.1E+06	5.3E+07	2.3E+07	1.1E+07
1236	Trihydroxy-prenylchalcone O-glucoside	$C_{26}H_{30}O_9$	[M+Na]+	509.17820	509.17838	-0.35	7.0E+06		5.7E+06		5.0E+06			
1237	Hydrocortisone cypionate	$C_{29}H_{42}O_6$	[M+Na]+	509.28736	509.28767	-0.61		3.4E+06						
1238	Alisol C	$C_{30}H_{46}O_5$	[M+Na]+	509.32375	509.32393	-0.37	4.1E+06	4.4E+06	4.6E+06			3.9E+06	4.8E+06	
1239	Diethyl-dihydroxy-methano-oxavitamin D3	$C_{31}H_{50}O_4$	[M+Na]+	509.36013	509.36031	-0.36		3.7E+06	3.3E+06			4.5E+06		
1240	Epoxy-ethoxyhopan-ol	$C_{32}H_{54}O_3$	[M+Na]+	509.39652	509.39670	-0.37		3.8E+06	3.9E+06			5.1E+06		
1241	Mycocerosic acid (C34)	$C_{34}H_{68}O_2$	[M+H]+	509.52921	509.52923	-0.05	5.6E+06	6.7E+06	5.9E+06	2.1E+06		1.2E+07	5.6E+06	3.7E+06
1242	Formyldihydrofolate	$C_{20}H_{21}N_7O_7$	[M+K]+	510.11341	510.11311	0.59	3.9E+06				3.5E+06			
1243	PG(18:1(9Z)/0:0)	$C_{24}H_{47}O_9P$	[M+H]+	511.30305	511.30325	-0.41	6.3E+06	7.4E+06	4.8E+06		4.6E+06	6.1E+06	4.3E+06	
1244	Acetoxy-dihydroxy-methyl-secocholestadienone	$C_{30}H_{48}O_5$	[M+Na]+	511.33940	511.33954	-0.28		5.9E+06						
1245	Cholestadienol benzoate	$C_{34}H_{48}O_2$	[M+Na]+	511.35465	511.35484	-0.37	3.1E+06	5.4E+06	4.8E+06			5.1E+06	7.2E+06	
1246	Trihydroxy-diethylvitamin D3	$C_{31}H_{52}O_4$	[M+Na]+	511.37578	511.37586	-0.15		3.3E+06				3.9E+06		
1247	Raucaffricine	$C_{27}H_{32}N_2O_8$	[M+H]+	513.22314	513.22269	0.88	7.5E+06		8.1E+06				6.6E+06	
1248	Epoxy-acetoxy-cholestentriol	$C_{29}H_{46}O_6$	[M+Na]+	513.31866	513.31881	-0.29	3.2E+06	3.7E+06					3.5E+06	
1249	Dihydroxy-(hydroxypropoxy)vitamin D3	$C_{30}H_{50}O_5$	[M+Na]+	513.35505	513.35508	-0.07		1.6E+07		3.5E+06		7.9E+06	7.1E+06	6.6E+06
1250	Aragusteroketal	$C_{31}H_{54}O_4$	[M+Na]+	513.39143	513.39151	-0.15						6.2E+06		
1251	Cyanidin acetylglucoside	$C_{23}H_{23}O_{12}$	[M+Na]+	514.10817	514.10796	0.41	6.9E+06		1.1E+07		6.5E+06		5.1E+06	
1252	Caryoptin	$C_{26}H_{36}O_9$	[M+Na]+	515.22515	515.22526	-0.21	4.7E+06							
1253	Dihydroxy-phenylvitamin D3	$C_{33}H_{48}O_3$	[M+Na]+	515.34957	515.34974	-0.34		4.3E+06						
1254	Xeniasterol-a	$C_{30}H_{52}O_5$	[M+Na]+	515.37070	515.37085	-0.30		6.0E+06						
1255	PI(12:0/0:0)	$C_{21}H_{41}O_{12}P$	[M+H]+	517.24084	517.24099	-0.28	2.6E+07		2.0E+07		1.8E+07		1.4E+07	
1256	Dehydroemetine	$C_{29}H_{38}N_2O_4$	[M+K]+	517.24632	517.24592	0.77							5.6E+06	
1257	Makisterone A	$C_{28}H_{46}O_7$	[M+Na]+	517.31357	517.31377	-0.38	7.7E+07	3.7E+06	5.1E+07		3.0E+07	5.5E+06	3.7E+07	
1258	Dihydroneopterin triphosphate	$C_9H_{16}N_5O_{13}P_3$	[M+Na]+	517.98497	517.98541	-0.86								3.8E+06
1259	Mycalamide B	$C_{25}H_{43}NO_{10}$	[M+H]+	518.29597	518.29595	0.04	6.8E+06		5.0E+06				4.9E+06	
1260	PC(O-16:0/O-1:0)	$C_{25}H_{54}NO_6P$	[M+Na]+	518.35810	518.35812	-0.05								4.1E+06

				ESI(+) FT-ICR MS										
**No.**	**Plausible Compound**[a]	**Molecular formula (M)**	**Ion**	**Theor m/z**[b]	**Exp. m/z**[c]	**Δppm**[d]	**SM$_R$H**[e]	**SM$_R$O**[e]	**SM$_P$H**[e]	**SM$_P$O**[e]	**TF$_R$H**[e]	**TF$_R$O**[e]	**TF$_P$H**[e]	**TF$_P$O**[e]
1261	Glaucarubin	$C_{25}H_{36}O_{10}$	[M+Na]+	519.22007	519.22028	-0.41	4.7E+06							
1262	Emetine	$C_{29}H_{40}N_2O_4$	[M+K]+	519.26197	519.26147	0.96	7.2E+06				7.1E+06		1.1E+07	
1263	20,26-Dihydroxyecdysone	$C_{27}H_{44}O_8$	[M+Na]+	519.29284	519.29307	-0.44	1.4E+07		1.3E+07		7.9E+06		6.9E+06	
1264	Hydroxy-epi-brassinolide	$C_{28}H_{48}O_7$	[M+Na]+	519.32922	519.32945	-0.42	6.5E+06				5.6E+06			
1265	Tetratriacontahexaenoic acid	$C_{34}H_{56}O_2$	[M+Na]+	519.41725	519.41736	-0.20			3.2E+06				3.7E+06	
1266	Ceforanide	$C_{20}H_{21}N_7O_6S_2$	[M+H]+	520.10675	520.10628	0.90					6.4E+06			
1267	PS(16:0/0:0)	$C_{22}H_{44}NO_9P$	[M+Na]+	520.26459	520.26487	-0.53					3.0E+06		3.4E+06	
1268	Epiiridodial glucoside tetraacetate	$C_{24}H_{34}O_{11}$	[M+Na]+	521.19933	521.19951	-0.34	4.1E+06							
1269	Triacontanedioic acid	$C_{30}H_{58}O_4$	[M+K]+	521.39667	521.39672	-0.09	6.9E+06				3.7E+06			
1270	Tetratriacontapentaenoic acid	$C_{34}H_{58}O_2$	[M+Na]+	521.43290	521.43299	-0.16		9.4E+06	4.6E+06	1.6E+06		1.1E+07	7.6E+06	5.5E+06
1271	Nonadecyl oleate	$C_{35}H_{68}O_2$	[M+H]+	521.52921	521.52921	0.00	1.4E+07	1.9E+07	1.7E+07	3.6E+06	5.7E+06	4.0E+07	1.6E+07	8.5E+06
1272	Hexafluoro-dihydroxy-didehydrovitamin D3	$C_{27}H_{36}F_6O_3$	[M+H]+	523.26414	523.26442	-0.52			5.5E+06				4.4E+06	
1273	Deoxocucurbitacin I	$C_{30}H_{44}O_6$	[M+Na]+	523.30301	523.30326	-0.48	3.4E+06	9.7E+06						
1274	Cholestan-octol	$C_{27}H_{48}O_8$	[M+Na]+	523.32414	523.32409	0.09					3.1E+06			
1275	Hydroxylanostane-dione acetate	$C_{32}H_{52}O_4$	[M+Na]+	523.37578	523.37592	-0.27		4.6E+06	4.0E+06			4.2E+06		
1276	Dihydroxy-hexamethylvitamin D3	$C_{33}H_{56}O_3$	[M+Na]+	523.41217	523.41235	-0.35		4.1E+06	4.8E+06			4.6E+06	4.4E+06	
1277	Tetratriacontatetraenoic acid	$C_{34}H_{60}O_2$	[M+Na]+	523.44855	523.44857	-0.03		5.6E+06	3.8E+06			6.2E+06	4.9E+06	3.8E+06
1278	Octadecyl-heptadecanoate	$C_{35}H_{70}O_2$	[M+H]+	523.54486	523.54486	0.00	5.1E+06	5.4E+06	4.8E+06		3.0E+06	9.8E+06	4.9E+06	3.5E+06
1279	Geranioloxyalatum flavone	$C_{27}H_{34}O_9$	[M+Na]+	525.20950	525.20968	-0.34	5.6E+06							
1280	Hexafluoro-Dihydroxyvitamin D3	$C_{27}H_{38}F_6O_3$	[M+H]+	525.27979	525.27999	-0.38	7.3E+06		7.2E+06		9.3E+06		7.8E+06	
1281	PG(19:1/0:0)	$C_{25}H_{49}O_9P$	[M+H]+	525.31870	525.31885	-0.30	4.4E+06	5.3E+06	3.4E+06			3.9E+06		
1282	Kestose	$C_{18}H_{32}O_{16}$	[M+Na]+	527.15826	527.15825	0.02		4.4E+06	4.7E+06	5.1E+06			9.2E+06	
1283	Eudesobovatol A	$C_{33}H_{44}O_4$	[M+Na]+	527.31318	527.31336	-0.34		3.3E+06						
1284	PG(19:0/0:0)	$C_{25}H_{51}O_9P$	[M+H]+	527.33435	527.33439	-0.07		5.7E+06				4.3E+06		
1285	PG(O-20:0/0:0)	$C_{26}H_{55}O_8P$	[M+H]+	527.37073	527.37071	0.04						4.3E+06		
1286	Peonidin 3-(6''-acetylglucoside)	$C_{24}H_{25}O_{12}$	[M+Na]+	528.12382	528.12359	0.45	4.2E+07		3.8E+07		3.9E+07		2.2E+07	
1287	(Hydroxypropoxy)vitamin D3	$C_{30}H_{50}O_5$	[M+K]+	529.32898	529.32902	-0.07					3.3E+06			
1288	Aragusteroketal	$C_{31}H_{54}O_4$	[M+K]+	529.36537	529.36538	-0.01			4.7E+06				4.5E+06	
1289	Hydroxy-norgeminivitamin D3	$C_{31}H_{54}O_5$	[M+Na]+	529.38635	529.38643	-0.16	3.6E+06	3.5E+06	3.0E+06			5.0E+06	3.8E+06	

				ESI(+) FT-ICR MS											
**No.**	**Plausible Compound[a]**	**Molecular formula (M)**	**Ion**	**Theor m/z[b]**	**Exp. m/z[c]**	**Δppm[d]**	**SM_RH^e**	**SM_RO^e**	**SM_PH^e**	**SM_PO^e**	**TF_RH^e**	**TF_RO^e**	**TF_PH^e**	**TF_PO^e**
1290	Delphinidin (acetylglucoside)	$C_{23}H_{23}O_{13}$	[M+Na]+	530.10309	530.10283	0.48	3.6E+06		4.9E+06					
1291	Mucronine B	$C_{28}H_{36}N_4O_4$	[M+K]+	531.23682	531.23646	0.67		9.2E+06				7.4E+06		
1292	Hexafluoro-hydroxyvitamin D3	$C_{27}H_{38}F_6O_2$	[M+Na]+	531.26682	531.26729	-0.88	5.4E+06		4.1E+06					
1293	Trihydroxy-carboxymethylcholestanoic acid	$C_{29}H_{48}O_7$	[M+Na]+	531.32922	531.32941	-0.34	7.7E+06		6.6E+06		4.6E+06		5.6E+06	
1294	Acetoxy-methylcholestantetrol	$C_{30}H_{52}O_6$	[M+Na]+	531.36561	531.36575	-0.26	6.5E+06	1.0E+07	8.3E+06		4.4E+06	5.1E+06	6.4E+06	
1295	DG(14:1/14:0/0:0)	$C_{31}H_{58}O_5$	[M+Na]+	533.41765	533.41771	-0.12		6.7E+06				6.5E+06		4.0E+06
1296	Gibberellin A2 O-beta-D-glucoside	$C_{25}H_{36}O_{11}$	[M+Na]+	535.21498	535.21516	-0.32	5.1E+06				4.5E+06			
1297	Trihydroxyecdysone	$C_{27}H_{44}O_9$	[M+Na]+	535.28775	535.28796	-0.39	1.4E+07		1.0E+07		8.9E+06		9.4E+06	
1298	Dimyristyl-sn-glycerol	$C_{31}H_{60}O_5$	[M+Na]+	535.43330	535.43334	-0.08		7.8E+06		2.0E+06		7.8E+06	5.7E+06	6.0E+06
1299	Eicosanyl oleate	$C_{36}H_{70}O_2$	[M+H]+	535.54486	535.54488	-0.04	1.5E+07	2.0E+07	1.7E+07	3.9E+06	4.6E+06	3.6E+07	1.6E+07	7.9E+06
1300	PC(P-20:0/0:0)	$C_{28}H_{58}NO_6P$	[M+H]+	536.40745	536.40700	0.84						3.6E+06		
1301	Nomilin	$C_{28}H_{34}O_9$	[M+Na]+	537.20950	537.20985	-0.64	9.9E+06		1.1E+07		9.3E+06		7.3E+06	
1302	Phthioceranic acid (C36)	$C_{36}H_{72}O_2$	[M+H]+	537.56051	537.56057	-0.11	3.5E+06	4.1E+06	3.8E+06			8.7E+06	4.1E+06	3.1E+06
1303	N-Palmitoylsphingosine	$C_{34}H_{67}NO_3$	[M+H]+	538.51937	538.51943	-0.11		4.7E+06						
1304	L-Olivosyl-oleandolide	$C_{26}H_{44}O_{10}$	[M+Na]+	539.28267	539.28272	-0.10	4.9E+06				6.1E+06			
1305	Fusidic acid	$C_{31}H_{48}O_6$	[M+Na]+	539.33431	539.33454	-0.42	5.5E+06	6.3E+06						
1306	Tetratriacontatetraenoic acid	$C_{34}H_{60}O_2$	[M+K]+	539.42249	539.42256	-0.13	3.6E+06							
1307	Carboxypyranocyanidin 3-O-beta-glucopyranoside	$C_{24}H_{21}O_{13}$	[M+Na]+	540.08744	540.08718	0.48	6.1E+06		4.9E+06		4.8E+06		4.6E+06	
1308	Hexacosanoyl carnitine	$C_{33}H_{65}NO_4$	[M+H]+	540.49864	540.49865	-0.03		6.1E+06						
1309	Hexafluoro-hydroxyvitamin D2	$C_{28}H_{38}F_6O_2$	[M+Na]+	543.26682	543.26641	0.76	6.1E+06							
1310	Dihydroxy-(hydroxymethylphenyl)-didehydrovitamin D3	$C_{34}H_{48}O_4$	[M+Na]+	543.34448	543.34471	-0.42			4.9E+06					
1311	Ophrysanin	$C_{23}H_{21}O_{14}$	[M+Na]+	544.08235	544.08217	0.33	1.1E+07		1.0E+07		1.0E+07		8.9E+06	
1312	Trilobolide	$C_{27}H_{38}O_{10}$	[M+Na]+	545.23572	545.23591	-0.35	6.1E+06		6.2E+06					
1313	Oleoylglycerophosphocholine	$C_{26}H_{53}NO_7P$	[M+Na]+	545.34519	545.34496	0.42	3.2E+06	9.7E+06		4.4E+06		4.9E+06		4.4E+06
1314	Westiellamide	$C_{27}H_{42}N_6O_6$	[M+H]+	547.32386	547.32433	-0.85	8.3E+07		4.1E+07		2.7E+07		3.6E+07	
1315	DG(14:1/15:0/0:0)	$C_{32}H_{60}O_5$	[M+Na]+	547.43330	547.43329	0.01		1.1E+07	4.6E+06	3.8E+06		9.8E+06	8.4E+06	5.4E+06
1316	Retinyl palmitate	$C_{36}H_{60}O_2$	[M+Na]+	547.44855	547.44865	-0.18		5.2E+06				3.9E+06		
1317	bacteriohopane-tetrol	$C_{35}H_{62}O_4$	[M+H]+	547.47209	547.47208	0.01						7.5E+06		

No.	Plausible Compound[a]	Molecular formula (M)	Ion	Theor m/z[b]	Exp. m/z[c]	Δppm[d]	SM$_{R}$H[e]	SM$_{R}$O[e]	SM$_{P}$H[e]	SM$_{P}$O[e]	TF$_{R}$H[e]	TF$_{R}$O[e]	TF$_{P}$H[e]	TF$_{P}$O[e]
				ESI(+) FT-ICR MS										
1318	DG(14:0/14:1/0:0)	$C_{31}H_{58}O_5$	[M+K]+	549.39158	549.39163	-0.08	6.6E+06							
1319	Anhydrobacteriohopantetrol	$C_{35}H_{58}O_3$	[M+Na]+	549.42782	549.42799	-0.31	5.3E+06	5.9E+06	5.6E+06				4.8E+06	
1320	DG(14:0/15:0/0:0)	$C_{32}H_{62}O_5$	[M+Na]+	549.44895	549.44897	-0.03		1.4E+07	5.8E+06	3.7E+06		1.5E+07	9.0E+06	7.3E+06
1321	Hydroxyphthioceranic acid (C33)	$C_{33}H_{66}O_3$	[M+K]+	549.46436	549.46425	0.19	6.7E+06	3.8E+07	1.5E+07	6.7E+06		3.7E+07	2.1E+07	1.3E+07
1322	Heneicosanyl oleate	$C_{37}H_{72}O_2$	[M+H]+	549.56051	549.56053	-0.04	1.0E+07	1.3E+07	1.2E+07	3.0E+06		2.7E+07	1.1E+07	6.0E+06
1323	Pachymic acid	$C_{33}H_{52}O_5$	[M+Na]+	551.37070	551.37096	-0.48		6.1E+06	2.2E+07			6.3E+06	8.5E+06	
1324	Dimyristyl-sn-glycerol	$C_{31}H_{60}O_5$	[M+K]+	551.40723	551.40712	0.21	1.2E+07				4.4E+06			
1325	Anhydrobacteriohopaneterol	$C_{35}H_{60}O_3$	[M+Na]+	551.44347	551.44360	-0.25	4.0E+06	5.3E+06	5.0E+06			5.3E+06	5.7E+06	
1326	Hexatriacontatetraenoic acid	$C_{36}H_{64}O_2$	[M+Na]+	551.47985	551.47989	-0.07		8.3E+06	5.0E+06	1.7E+06		9.5E+06	6.8E+06	5.7E+06
1327	(Tetradecenyl)-(octadecenoyl)-sn-glycerol	$C_{35}H_{66}O_4$	[M+H]+	551.50339	551.50339	-0.01	1.0E+07	9.2E+06	7.2E+06	2.5E+06	3.5E+06	2.0E+07	7.3E+06	5.5E+06
1328	Phthioceranic acid (C37)	$C_{37}H_{74}O_2$	[M+H]+	551.57616	551.57616	0.00						6.6E+06		
1329	((O-hydroxy-ethane)-hydroxy-propane)-hopane	$C_{35}H_{62}O_3$	[M+Na]+	553.45912	553.45906	0.10						9.4E+06		
1330	Calactin	$C_{29}H_{40}O_9$	[M+Na]+	555.25645	555.25664	-0.34	7.5E+06							
1331	Mayolene-16	$C_{34}H_{60}O_4$	[M+Na]+	555.43838	555.43843	-0.08		5.0E+06				5.4E+06		
1332	Cer(t18:0/16:0)	$C_{34}H_{69}NO_4$	[M+H]+	556.52994	556.52990	0.06		4.7E+06				4.0E+06		
1333	PG(20:3/0:0)	$C_{26}H_{47}O_9P$	[M+Na]+	557.28499	557.28449	0.90	4.9E+06							
1334	Cyanidin (malonylglucoside)	$C_{24}H_{23}O_{14}$	[M+Na]+	558.09800	558.09776	0.43	5.2E+07		3.1E+07		4.9E+07		1.6E+07	
1335	DG(14:1/16:1/0:0)	$C_{33}H_{60}O_5$	[M+Na]+	559.43330	559.43330	0.00		8.1E+06	4.9E+06			7.7E+06	6.9E+06	5.3E+06
1336	Hexatriacontylic acid	$C_{36}H_{72}O_2$	[M+Na]+	559.54245	559.54242	0.06				4.9E+06				
1337	PG(22:4/0:0)	$C_{28}H_{49}O_9P$	[M+H]+	561.31870	561.31889	-0.35		1.1E+07				4.4E+06		
1338	Deferoxamine	$C_{25}H_{48}N_6O_8$	[M+H]+	561.36064	561.36092	-0.49	1.2E+07		1.3E+07		1.1E+07		1.5E+07	
1339	DG(14:0/16:1/0:0)	$C_{33}H_{62}O_5$	[M+Na]+	561.44895	561.44896	-0.02	5.6E+06	2.4E+07	9.4E+06	5.0E+06			1.5E+07	1.2E+07
1340	Methyl-hexatriacontahexaenoic acid	$C_{37}H_{62}O_2$	[M+Na]+	561.46420	561.46438	-0.31	4.4E+06		4.6E+06			3.7E+06	4.6E+06	
1341	Aminobacteriohopanetetrol	$C_{35}H_{63}NO_4$	[M+H]+	562.48299	562.48300	-0.02			9.9E+06					
1342	Tetrahydroaldosterone glucuronide	$C_{27}H_{40}O_{11}$	[M+Na]+	563.24628	563.24630	-0.03	4.7E+06							
1343	DG(14:1/15:0/0:0)	$C_{32}H_{60}O_5$	[M+K]+	563.40723	563.40706	0.31					3.8E+06			
1344	DG(14:0/16:0/0:0)	$C_{33}H_{64}O_5$	[M+Na]+	563.46460	563.46463	-0.06	6.0E+06	2.1E+07	7.5E+06	6.1E+06		2.4E+07	1.2E+07	1.1E+07
1345	CE(10:0)	$C_{37}H_{64}O_2$	[M+Na]+	563.47985	563.47988	-0.05		1.1E+07	5.2E+06	2.2E+06		1.2E+07		6.7E+06
1346	Docosanyl oleate	$C_{38}H_{74}O_2$	[M+H]+	563.57616	563.57615	0.01	8.2E+06	1.0E+07	8.7E+06	3.3E+06	4.4E+06	2.0E+07	9.0E+06	5.0E+06

				ESI(+) FT-ICR MS									
**No.**	**Plausible Compound**[a]	**Molecular formula (M)**	**Ion**	**Theor m/z**[b]	**Exp. m/z**[c]	**Δppm**[d]	**SM_RH^e**	**SM_RO^e**	**SM_PH^e**	**SM_PO^e**	**TF_RH^e**	**TF_RO^e**	**TF_PH^e**	**TF_PO^e**
1347	Cortolone-3-glucuronide	$C_{27}H_{42}O_{11}$	[M+Na]+	565.26193	565.26209	-0.28	2.2E+07		2.0E+07		1.6E+07		1.8E+07	
1348	(D-ribonyl)hopane	$C_{35}H_{58}O_4$	[M+Na]+	565.42273	565.42293	-0.36	1.7E+07	1.6E+07	2.0E+07	9.4E+06	5.0E+06	1.4E+07	2.0E+07	4.7E+06
1349	Methyl-anhydrobacteriohopanetetrol	$C_{36}H_{62}O_3$	[M+Na]+	565.45912	565.45923	-0.19		5.9E+06	3.9E+06			6.7E+06		
1350	Tetramethyl-tetratriacontanoic acid	$C_{38}H_{76}O_2$	[M+H]+	565.59181	565.59182	-0.02						5.5E+06		
1351	Hemiglutaryloxy-dihydroxyvitamin D3	$C_{33}H_{52}O_6$	[M+Na]+	567.36561	567.36591	-0.53							3.7E+06	
1352	DG(14:0/18:1/0:0)	$C_{35}H_{66}O_5$	[M+H]+	567.49830	567.49829	0.02						3.5E+06		
1353	Adouetine Y	$C_{34}H_{40}N_4O_4$	[M+H]+	569.31223	569.31191	0.57			8.4E+06					
1354	Cholest-dienol O-beta-D-glucopyranoside	$C_{33}H_{54}O_6$	[M+Na]+	569.38126	569.38130	-0.07		4.1E+06				3.5E+06		
1355	Retinyl linolate	$C_{38}H_{58}O_2$	[M+Na]+	569.43290	569.43309	-0.34					8.3E+07	1.1E+07	6.1E+06	
1356	Hexaprenyl-methoxy-benzoquinol	$C_{37}H_{56}O_3$	[M+Na]+	571.41217	571.41206	0.18		4.6E+06	4.2E+06			4.1E+06	5.1E+06	
1357	Dihydrochlorobactene	$C_{40}H_{54}$	[M+K]+	573.38571	573.38557	0.25						6.3E+06		
1358	Canthiumine	$C_{33}H_{36}N_4O_4$	[M+Na]+	575.26288	575.26259	0.50						6.7E+06		
1359	Lithocholate O-glucuronide	$C_{30}H_{48}O_9$	[M+Na]+	575.31905	575.31924	-0.32	7.1E+06				5.0E+06			
1360	DG(15:0/16:1/0:0)	$C_{34}H_{64}O_5$	[M+Na]+	575.46460	575.46459	0.01	7.2E+06	2.9E+07	1.2E+07	6.4E+06	3.5E+06	2.9E+07	1.7E+07	1.3E+07
1361	Octatriacontahexaenoic acid	$C_{38}H_{64}O_2$	[M+Na]+	575.47985	575.47999	-0.24	4.1E+06	6.2E+06	4.2E+06			6.0E+06	5.0E+06	
1362	Toonacilin	$C_{31}H_{38}O_9$	[M+Na]+	577.24080	577.24100	-0.33	8.3E+06		6.7E+06		6.0E+06			
1363	6,8a-Seco-6,8a-deoxy-5-oxoavermectin "1b" aglycone	$C_{33}H_{46}O_7$	[M+Na]+	577.31357	577.31370	-0.22		7.7E+06	3.7E+06					
1364	PA(P-16:0/12:0)	$C_{31}H_{61}O_7P$	[M+H]+	577.42277	577.42289	-0.21	2.8E+07	3.2E+07	4.1E+07	7.2E+06	7.0E+06	2.5E+07	4.4E+07	7.7E+06
1365	DG(15:0/16:0/0:0)	$C_{34}H_{66}O_5$	[M+Na]+	577.48025	577.48028	-0.05	6.7E+06	2.9E+07	1.0E+07	7.6E+06		3.2E+07	1.5E+07	1.4E+07
1366	Octatriacontapentaenoic acid	$C_{38}H_{66}O_2$	[M+Na]+	577.49550	577.49549	0.02		7.6E+06	5.2E+06	2.1E+06		7.6E+06	5.5E+06	4.4E+06
1367	Tricosanyl oleate	$C_{39}H_{76}O_2$	[M+H]+	577.59181	577.59182	-0.02	6.0E+06	7.0E+06	6.1E+06			1.5E+07	5.9E+06	4.2E+06
1368	PA(O-16:0/12:0)	$C_{31}H_{63}O_7P$	[M+H]+	579.43842	579.43855	-0.23	3.1E+07	3.0E+07	4.0E+07	8.6E+06	7.7E+06	2.6E+07	4.1E+07	7.5E+06
1369	Chloroxanthin	$C_{40}H_{60}O$	[M+Na]+	579.45364	579.45381	-0.30	6.7E+06	1.1E+07	1.5E+07		3.6E+06	1.1E+07	1.4E+07	
1370	Octatriacontatetraenoic acid	$C_{38}H_{68}O_2$	[M+Na]+	579.51115	579.51118	-0.05		6.9E+06	4.1E+06			7.9E+06		
1371	Hexadecyl-tricosanoate	$C_{39}H_{78}O_2$	[M+H]+	579.60746	579.60749	-0.05						4.1E+06		
1372	Dihydroergotamine	$C_{33}H_{37}N_5O_5$	[M+H]+	584.28675	584.28661	0.23			4.9E+06					
1373	(Acetoxymethylphenyl)-dihydroxy-didehydrovitamin D3	$C_{36}H_{50}O_5$	[M+Na]+	585.35505	585.35536	-0.54		3.4E+06						
1374	Cholesterol glucuronide	$C_{33}H_{54}O_7$	[M+Na]+	585.37617	585.37634	-0.28	5.9E+06							

					ESI(+) FT-ICR MS								
**No.**	**Plausible Compound**[a]	**Molecular formula (M)**	**Ion**	**Theor m/z**[b]	**Exp. m/z**[c]	**Δppm**[d]	**SM$_R$H**[e]	**SM$_R$O**[e]	**SM$_P$H**[e]	**SM$_P$O**[e]	**TF$_R$H**[e]	**TF$_R$O**[e]	**TF$_P$H**[e]	**TF$_P$O**[e]
1375	DG(14:0/18:3/0:0)	$C_{35}H_{62}O_5$	[M+Na]+	585.44895	585.44895	0.00		5.0E+06				4.9E+06		
1376	Phycocyanobilin	$C_{33}H_{38}N_4O_6$	[M+H]+	587.28641	587.28643	-0.03	1.0E+07		8.0E+06		7.3E+06		7.3E+06	
1377	DG(14:0/18:2/0:0)	$C_{35}H_{64}O_5$	[M+Na]+	587.46460	587.46462	-0.03	7.0E+06	2.6E+07	1.2E+07	5.4E+06	3.7E+06	2.6E+07	1.8E+07	1.1E+07
1378	Antiarigenin O-antiaroside	$C_{29}H_{42}O_{11}$	[M+Na]+	589.26193	589.26206	-0.22	8.5E+06		6.3E+06		6.6E+06			
1379	Sarmentoloside	$C_{29}H_{44}O_{11}$	[M+Na]+	591.27758	591.27761	-0.05	3.9E+06							
1380	PA(P-16:0/13:0)	$C_{32}H_{63}O_7P$	[M+H]+	591.43842	591.43854	-0.20	3.8E+07	3.6E+07	5.7E+07	9.6E+06	7.5E+06	3.1E+07	5.8E+07	7.9E+06
1381	Dihydroanhydrorhodovibrin	$C_{41}H_{60}O$	[M+Na]+	591.45364	591.45383	-0.33			5.0E+06					
1382	DG(14:0/18:0/0:0)	$C_{35}H_{68}O_5$	[M+Na]+	591.49590	591.49591	-0.02	8.6E+06	2.9E+07	1.1E+07	8.3E+06	4.4E+06	3.5E+07	1.6E+07	1.7E+07
1383	Coixenolide	$C_{38}H_{70}O_4$	[M+H]+	591.53469	591.53475	-0.11						4.0E+06		
1384	Tetracosanyl oleate	$C_{40}H_{78}O_2$	[M+H]+	591.60746	591.60742	0.06	5.3E+06	6.2E+06	5.2E+06	1.7E+06		1.4E+07	5.4E+06	4.5E+06
1385	Avermectin B1b aglycone	$C_{33}H_{46}O_8$	[M+Na]+	593.30849	593.30869	-0.34		7.3E+06						
1386	PA(12:0/16:0)	$C_{31}H_{61}O_8P$	[M+H]+	593.41768	593.41795	-0.45		4.0E+06						
1387	PA(O-16:0/13:0)	$C_{32}H_{65}O_7P$	[M+H]+	593.45407	593.45418	-0.18	3.6E+07	3.2E+07	4.7E+07	1.1E+07	9.1E+06	3.0E+07	4.8E+07	7.6E+06
1388	Dihydrospheroidene	$C_{41}H_{62}O$	[M+Na]+	593.46929	593.46945	-0.28	5.0E+06	6.5E+06	1.0E+07			6.7E+06	9.7E+06	
1389	Cer(d18:0/20:0)	$C_{38}H_{77}NO_3$	[M+H]+	596.59762	596.59773	-0.18						3.6E+06		
1390	DG(15:0/18:3/0:0)	$C_{36}H_{64}O_5$	[M+Na]+	599.46460	599.46466	-0.11		4.7E+06				3.9E+06		
1391	Albanol A	$C_{34}H_{26}O_8$	[M+K]+	601.12593	601.12642	-0.82							4.2E+06	
1392	Hexaprenyl-methyl-hydroxy-methoxy-benzoquinol	$C_{38}H_{58}O_4$	[M+Na]+	601.42273	601.42293	-0.34		3.9E+06	5.4E+06				4.9E+06	
1393	DG(15:0/18:2/0:0)	$C_{36}H_{66}O_5$	[M+Na]+	601.48025	601.48028	-0.05	4.4E+06	1.3E+07	6.0E+06	3.4E+06		1.3E+07	1.1E+07	5.6E+06
1394	PA(P-16:0/14:1)	$C_{33}H_{63}O_7P$	[M+H]+	603.43842	603.43853	-0.19	2.7E+07	3.1E+07	4.5E+07	7.0E+06	5.4E+06	2.4E+07	4.5E+07	7.6E+06
1395	DG(15:0/18:1/0:0)	$C_{36}H_{68}O_5$	[M+Na]+	603.49590	603.49588	0.03	6.3E+06	2.3E+07	9.1E+06	5.7E+06	3.7E+06		1.5E+07	1.1E+07
1396	Polidocanol	$C_{30}H_{62}O_{10}$	[M+Na]+	605.42352	605.42356	-0.06		8.6E+06	4.1E+06	4.6E+06	4.2E+06	1.2E+07	8.7E+06	7.4E+06
1397	PA(O-16:0/14:1)	$C_{33}H_{65}O_7P$	[M+H]+	605.45407	605.45420	-0.21	5.6E+07	5.5E+07	8.3E+07	1.3E+07	1.1E+07	4.6E+07	8.4E+07	1.1E+07
1398	DG(15:0/18:0/0:0)	$C_{36}H_{70}O_5$	[M+Na]+	605.51155	605.51157	-0.04	4.6E+06	1.7E+07	6.0E+06	5.4E+06		2.1E+07	1.2E+07	8.6E+06
1399	Hydroxyphthioceranic acid (C37)	$C_{37}H_{74}O_3$	[M+K]+	605.52696	605.52690	0.09						4.5E+06		
1400	Pentacosanyl oleate	$C_{41}H_{80}O_2$	[M+H]+	605.62311	605.62304	0.11	3.9E+06					6.6E+06	4.0E+06	
1401	Pubescenol	$C_{32}H_{40}O_{10}$	[M+Na]+	607.25137	607.25151	-0.23	6.1E+06							
1402	Ouabain	$C_{29}H_{44}O_{12}$	[M+Na]+	607.27250	607.27257	-0.12	5.4E+06							
1403	PA(O-16:0/14:0)	$C_{33}H_{67}O_7P$	[M+H]+	607.46972	607.46985	-0.22	4.5E+07	3.5E+07	4.9E+07	1.1E+07	1.0E+07	3.2E+07	5.0E+07	8.3E+06

				ESI(+) FT-ICR MS											
**No.**	**Plausible Compound[a]**	**Molecular formula (M)**	**Ion**	**Theor m/z[b]**	**Exp. m/z[c]**	**Δppm[d]**	**SM_RH^e**	**SM_RO^e**	**SM_PH^e**	**SM_PO^e**	**TF_RH^e**	**TF_RO^e**	**TF_PH^e**	**TF_PO^e**
1404	5-Oxoavermectin "2b" aglycone	$C_{33}H_{46}O_9$	[M+Na]+	609.30340	609.30356	-0.26		5.6E+06						
1405	Niguldipine	$C_{36}H_{39}N_3O_6$	[M+H]+	610.29116	610.29160	-0.72	5.4E+06		7.5E+06		5.6E+06			
1406	Glucosylkaempferol 3-O-glucoside	$C_{27}H_{30}O_{16}$	[M+H]+	611.16066	611.16073	-0.11							6.3E+06	
1407	Cyclo[D-trp-D-asp-L-pro-D-val-L-leu]	$C_{31}H_{42}N_6O_7$	[M+H]+	611.31877	611.31922	-0.73		6.9E+06				3.7E+06		
1408	Hydroxyvitamin D2 (beta-glucuronide)	$C_{34}H_{52}O_8$	[M+Na]+	611.35544	611.35557	-0.21		1.1E+07						
1409	DG(14:0/20:4/0:0)	$C_{37}H_{64}O_5$	[M+Na]+	611.46460	611.46461	-0.01		5.2E+06				4.4E+06		
1410	Glutathione disulfide	$C_{20}H_{32}N_6O_{12}S_2$	[M+H]+	613.15924	613.15938	-0.22			3.8E+06				4.9E+06	
1411	(O-D-glucopyranosyl)-keto-hexacosanediol	$C_{32}H_{62}O_8$	[M+K]+	613.40763	613.40779	-0.26	3.7E+06							
1412	DG(14:0/20:3/0:0)	$C_{37}H_{66}O_5$	[M+Na]+	613.48025	613.48028	-0.05		9.5E+06	4.8E+06	1.9E+06		1.0E+07	8.0E+06	5.3E+06
1413	Myricetin (triacetylxyloside)	$C_{26}H_{24}O_{15}$	[M+K]+	615.07468	615.07457	0.17	1.3E+07		4.7E+06		4.9E+06		8.9E+06	
1414	Ubiquinol-6	$C_{39}H_{60}O_4$	[M+Na]+	615.43838	615.43861	-0.37		4.9E+06	5.2E+06				5.9E+06	
1415	DG(14:0/20:2/0:0)	$C_{37}H_{68}O_5$	[M+Na]+	615.49590	615.49591	-0.02	9.2E+06	2.8E+07	1.3E+07	5.9E+06		2.9E+07	1.7E+07	1.2E+07
1416	Tetracosyl-palmitate	$C_{40}H_{80}O_2$	[M+Na]+	615.60505	615.60500	0.09						6.2E+06		
1417	PA(P-16:0/15:1)	$C_{34}H_{65}O_7P$	[M+H]+	617.45407	617.45418	-0.19	1.7E+07	1.7E+07	2.8E+07	7.7E+06	4.6E+06	1.4E+07	2.6E+07	5.5E+06
1418	Hexadecanoyl-(octadecenoyl)-sn-glycerol	$C_{37}H_{70}O_5$	[M+Na]+	617.51155	617.51153	0.02	1.3E+07	2.9E+07	1.1E+07	6.7E+06		3.2E+07	1.5E+07	1.2E+07
1419	Cholesteryl ester	$C_{41}H_{70}O_2$	[M+Na]+	617.52680	617.52687	-0.10		4.7E+06	4.2E+06			6.1E+06	4.5E+06	3.3E+06
1420	CerP(d18:1/16:0)	$C_{34}H_{68}NO_6P$	[M+H]+	618.48570	618.48519	0.83						3.8E+06		
1421	O-[beta-D-glucopyranosyl]-furostantriol	$C_{33}H_{56}O_9$	[M+Na]+	619.38165	619.38184	-0.30	2.1E+07	3.7E+06	1.4E+07		1.1E+07		1.0E+07	
1422	PA(12:0/18:1)	$C_{33}H_{63}O_8P$	[M+H]+	619.43333	619.43357	-0.38		4.2E+06				4.0E+06		
1423	PA(O-16:0/15:1)	$C_{34}H_{67}O_7P$	[M+H]+	619.46972	619.46979	-0.11	3.5E+07	3.1E+07	4.9E+07	9.2E+06	6.8E+06	2.8E+07	4.9E+07	7.0E+06
1424	DG(14:0/20:0/0:0)	$C_{37}H_{72}O_5$	[M+Na]+	619.52720	619.52717	0.04	4.3E+06	9.8E+06	4.6E+06	3.6E+06		1.3E+07	7.2E+06	1.0E+07
1425	Hexacosanyl oleate	$C_{42}H_{82}O_2$	[M+H]+	619.63876	619.63883	-0.12						4.6E+06		
1426	Kurilensoside G	$C_{32}H_{54}O_{10}$	[M+Na]+	621.36092	621.36101	-0.15	7.9E+06				4.0E+06		5.9E+06	
1427	PA(12:0/18:0)	$C_{33}H_{65}O_8P$	[M+H]+	621.44898	621.44914	-0.25	5.2E+06	5.4E+06	4.1E+06			5.1E+06	4.9E+06	
1428	PA(O-16:0/15:0)	$C_{34}H_{69}O_7P$	[M+H]+	621.48537	621.48547	-0.16	2.3E+07	1.9E+07	2.8E+07	8.2E+06	6.6E+06	1.9E+07	2.7E+07	6.1E+06
1429	Cer(d18:0/22:1)	$C_{40}H_{79}NO_3$	[M+H]+	622.61327	622.61326	0.02		3.5E+06						
1430	Hydroxy-oxobaccatin III	$C_{31}H_{36}O_{12}$	[M+Na]+	623.20990	623.21001	-0.18	4.0E+06				3.5E+06		3.8E+06	
1431	Avermectin A2b aglycone	$C_{34}H_{50}O_9$	[M+Na]+	625.33470	625.33486	-0.26	4.8E+06	6.8E+06	4.0E+06		4.2E+06	4.5E+06		
1432	(O-glucopyranosyl)-keto-octacosanediol	$C_{34}H_{66}O_8$	[M+Na]+	625.46499	625.46511	-0.18	4.4E+06		4.9E+06		4.3E+06		5.0E+06	

No.	Plausible Compound[a]	Molecular formula (M)	Ion	Theor m/z[b]	Exp. m/z[c]	Δppm[d]	SM$_{R}$H[e]	SM$_{R}$O[e]	SM$_{P}$H[e]	SM$_{P}$O[e]	TF$_{R}$H[e]	TF$_{R}$O[e]	TF$_{P}$H[e]	TF$_{P}$O[e]
				ESI(+) FT-ICR MS										
1433	DG(15:0/20:4/0:0)	$C_{38}H_{66}O_5$	[M+Na]+	625.48025	625.48038	-0.21		5.0E+06	4.3E+06			5.2E+06		
1434	N-((methyl-hexadecanoyloxy)-methyl-tetradecanoyl)-L-serine	$C_{37}H_{71}NO_6$	[M+H]+	626.53542	626.53539	0.04	5.7E+06	6.3E+06	8.2E+06			7.5E+06	7.9E+06	
1435	Cerbertin	$C_{32}H_{44}O_{11}$	[M+Na]+	627.27758	627.27777	-0.30	3.4E+07		2.5E+07		2.0E+07		1.9E+07	
1436	DG(15:0/20:3/0:0)	$C_{38}H_{68}O_5$	[M+Na]+	627.49590	627.49580	0.16		4.4E+06				3.6E+06		
1437	Pentacosanyl oleate	$C_{41}H_{80}O_2$	[M+Na]+	627.60505	627.60510	-0.07			5.5E+06	3.6E+06				
1438	DG(14:0/20:3/0:0)	$C_{37}H_{66}O_5$	[M+K]+	629.45418	629.45423	-0.07	8.9E+06							
1439	DG(15:0/20:2/0:0)	$C_{38}H_{70}O_5$	[M+Na]+	629.51155	629.51145	0.15		6.4E+06	4.0E+06			8.6E+06	5.5E+06	4.6E+06
1440	Tetracosanyl oleate	$C_{40}H_{78}O_2$	[M+K]+	629.56334	629.56349	-0.24					4.4E+06			
1441	Methyl-tetracosanoic acid	$C_{41}H_{82}O_2$	[M+Na]+	629.62070	629.62085	-0.23						3.7E+06		
1442	PA(P-16:0/16:1)	$C_{35}H_{67}O_7P$	[M+H]+	631.46972	631.46982	-0.16	3.4E+07	3.0E+07	4.5E+07	1.0E+07	6.0E+06	2.6E+07	4.1E+07	6.8E+06
1443	DG(15:0/20:1/0:0)	$C_{38}H_{72}O_5$	[M+Na]+	631.52720	631.52716	0.05		6.7E+06	3.9E+06			7.3E+06		5.7E+06
1444	15:1 Cholesteryl ester	$C_{42}H_{72}O_2$	[M+Na]+	631.54245	631.54250	-0.08		4.2E+06	4.5E+06			7.1E+06	5.1E+06	
1445	PA(O-16:0/16:1)	$C_{35}H_{69}O_7P$	[M+H]+	633.48537	633.48548	-0.17	5.2E+07	3.2E+07	4.3E+07	8.9E+06	5.8E+06	2.8E+07	4.1E+07	6.0E+06
1446	DG(15:0/20:0/0:0)	$C_{38}H_{74}O_5$	[M+Na]+	633.54285	633.54287	-0.04		5.0E+06				8.5E+06		4.4E+06
1447	15:0 Cholesteryl ester	$C_{42}H_{74}O_2$	[M+Na]+	633.55810	633.55805	0.08			4.1E+06	2.2E+06		6.5E+06	3.8E+06	4.7E+06
1448	Cholestane-tetrol-glucuronide	$C_{33}H_{56}O_{10}$	[M+Na]+	635.37657	635.37673	-0.25	6.2E+07		4.1E+07		2.7E+07		3.0E+07	
1449	DG(14:0/22:6/0:0)	$C_{39}H_{64}O_5$	[M+Na]+	635.46460	635.46476	-0.26			4.0E+06					
1450	PA(O-16:0/16:0)	$C_{35}H_{71}O_7P$	[M+H]+	635.50102	635.50112	-0.16	1.5E+07	1.1E+07	1.6E+07	8.4E+06	5.9E+06	1.0E+07	1.5E+07	5.7E+06
1451	(D-xylopyranosyloxy)-epoxycholestan-pentol	$C_{32}H_{54}O_{11}$	[M+Na]+	637.35583	637.35587	-0.06	4.9E+06							
1452	Evasterioside D	$C_{33}H_{58}O_{10}$	[M+Na]+	637.39222	637.39234	-0.20	1.4E+07	7.1E+06	1.1E+07		8.1E+06	4.6E+06	9.1E+06	
1453	DG(14:0/22:5/0:0)	$C_{39}H_{66}O_5$	[M+Na]+	637.48025	637.48037	-0.19	4.7E+06	6.2E+06	5.0E+06			5.8E+06	4.4E+06	
1454	Avermectin A2a aglycone	$C_{35}H_{52}O_9$	[M+Na]+	639.35035	639.35054	-0.29		5.8E+06						
1455	PG(12:0/14:0)	$C_{32}H_{63}O_{10}P$	[M+H]+	639.42316	639.42325	-0.14	4.2E+06							
1456	DG(14:0/22:4/0:0)	$C_{39}H_{68}O_5$	[M+Na]+	639.49590	639.49590	0.00	5.7E+06	1.3E+07	8.7E+06	2.5E+06		9.0E+06	9.3E+06	5.1E+06
1457	Bryotoxin A	$C_{32}H_{42}O_{12}$	[M+Na]+	641.25685	641.25695	-0.16	6.8E+06							
1458	(O-D-glucopyranosyl)-keto-octacosanetriol	$C_{34}H_{66}O_9$	[M+Na]+	641.45990	641.45995	-0.07	3.6E+06				4.4E+06			
1459	DG(14:1/22:2/0:0)	$C_{39}H_{70}O_5$	[M+Na]+	641.51155	641.51151	0.05	4.9E+06	1.2E+07				9.3E+06	6.9E+06	4.7E+06
1460	Hexacosanyl oleate	$C_{42}H_{82}O_2$	[M+Na]+	641.62070	641.62054	0.25		5.8E+06						5.3E+06

				ESI(+) FT-ICR MS										
**No.**	**Plausible Compound**[a]	**Molecular formula (M)**	**Ion**	**Theor m/z**[b]	**Exp. m/z**[c]	**Δppm**[d]	**SM$_R$H**[e]	**SM$_R$O**[e]	**SM$_P$H**[e]	**SM$_P$O**[e]	**TF$_R$H**[e]	**TF$_R$O**[e]	**TF$_P$H**[e]	**TF$_P$O**[e]
1461	Myricetin 3,3'-digalactoside	$C_{27}H_{30}O_{18}$	[M+H]+	643.15049	643.15061	-0.19					4.2E+06			
1462	(Ladderane-hexanoyl)-ladderane-octanyl)-sn-glycerol	$C_{41}H_{64}O_4$	[M+Na]+	643.46968	643.46975	-0.10		4.8E+06	5.7E+06				6.6E+06	
1463	DG(14:0/22:2/0:0)	$C_{39}H_{72}O_5$	[M+Na]+	643.52720	643.52717	0.05	6.5E+06	1.5E+07	6.3E+06	3.6E+06		1.3E+07	8.6E+06	5.9E+06
1464	Pentacosanyl oleate	$C_{41}H_{80}O_2$	[M+K]+	643.57899	643.57879	0.31					4.0E+06			
1465	Galactosylceramide (d18:1/12:0)	$C_{36}H_{69}NO_8$	[M+H]+	644.50959	644.50939	0.32						3.7E+06		
1466	PA(12:0/20:2)	$C_{35}H_{65}O_8P$	[M+H]+	645.44898	645.44911	-0.19	4.0E+06					4.3E+06		
1467	PA(O-16:0/17:2)	$C_{36}H_{69}O_7P$	[M+H]+	645.48537	645.48549	-0.18	8.5E+06	7.2E+06	1.3E+07			6.8E+06	1.1E+07	
1468	DG(14:0/22:1/0:0)	$C_{39}H_{74}O_5$	[M+Na]+	645.54285	645.54285	-0.01		6.9E+06				5.9E+06		3.9E+06
1469	Vitamin D3 palmitate	$C_{43}H_{74}O_2$	[M+Na]+	645.55810	645.55807	0.05	5.1E+06	1.2E+07	6.1E+06	4.2E+06		1.8E+07	7.9E+06	8.5E+06
1470	DG(17:2/20:5/0:0)	$C_{40}H_{64}O_5$	[M+Na]+	647.46460	647.46467	-0.11	4.8E+06	4.0E+06					5.4E+06	
1471	PA(O-16:0/17:1)	$C_{36}H_{71}O_7P$	[M+H]+	647.50102	647.50113	-0.17	1.1E+07	8.7E+06	1.3E+07			7.9E+06	1.2E+07	
1472	DG(14:0/22:0/0:0)	$C_{39}H_{76}O_5$	[M+Na]+	647.55850	647.55850	-0.01		4.0E+06				5.5E+06		5.0E+06
1473	16:0 Cholesteryl ester	$C_{43}H_{76}O_2$	[M+Na]+	647.57375	647.57371	0.07						7.5E+06		4.1E+06
1474	Cer (d18:1/24:1(15Z))	$C_{42}H_{81}NO_3$	[M+H]+	648.62892	648.62860	0.50						4.1E+06		
1475	Hydroxykaempferol caffeylglucoside	$C_{30}H_{26}O_{15}$	[M+Na]+	649.11639	649.11653	-0.21	4.3E+06						7.0E+06	
1476	O-(Glcb)-spirostantriol	$C_{34}H_{58}O_{10}$	[M+Na]+	649.39222	649.39242	-0.31	2.5E+08	4.2E+06	1.6E+08	6.0E+06	1.0E+08	9.8E+06	1.1E+08	
1477	Methoxy-methyloctadecenynoyl anhydride	$C_{40}H_{66}O_5$	[M+Na]+	649.48025	649.48050	-0.38	9.8E+06	5.6E+06	5.1E+06				5.8E+06	
1478	PA(O-16:0/17:0)	$C_{36}H_{73}O_7P$	[M+H]+	649.51667	649.51673	-0.10	7.2E+06	4.7E+06	7.2E+06			6.2E+06	5.8E+06	
1479	Squalamine	$C_{34}H_{65}N_3O_5S$	[M+Na]+	650.45371	650.45322	0.76						5.5E+06		
1480	Perhydroazepino-N-carbonyl-L-Leu-D-Trp-D-Trp	$C_{35}H_{44}N_6O_5$	[M+Na]+	651.32654	651.32632	0.34			3.7E+06		3.5E+06			
1481	PI(P-20:0/0:0)	$C_{29}H_{57}O_{11}P$	[M+K]+	651.32701	651.32646	0.84					3.5E+06			
1482	PA(13:0/18:3)	$C_{34}H_{61}O_8P$	[M+Na]+	651.39963	651.39914	0.75	1.6E+07		1.5E+07		8.3E+06			
1483	DG(15:0/22:5/0:0)	$C_{40}H_{68}O_5$	[M+Na]+	651.49590	651.49617	-0.41	3.9E+06	4.4E+06	4.2E+06			4.4E+06	4.6E+06	
1484	PI(22:2/0:0)	$C_{31}H_{57}O_{12}P$	[M+H]+	653.36604	653.36622	-0.27		5.5E+06						
1485	PG(12:0/15:0)	$C_{33}H_{65}O_{10}P$	[M+H]+	653.43881	653.43879	0.03	5.4E+06	4.6E+06	3.9E+06					
1486	PA(P-16:0/18:4)	$C_{37}H_{65}O_7P$	[M+H]+	653.45407	653.45422	-0.23			4.2E+06					
1487	DG(15:0/22:4/0:0)	$C_{40}H_{70}O_5$	[M+Na]+	653.51155	653.51163	-0.12	3.7E+06	5.5E+06				6.1E+06	4.5E+06	
1488	Oleanoic acid 3-O-glucuronide	$C_{36}H_{56}O_9$	[M+Na]+	655.38165	655.38200	-0.53		7.5E+06						

				ESI(+) FT-ICR MS											
**No.**	**Plausible Compound**[a]	**Molecular formula (M)**	**Ion**	**Theor m/z**[b]	**Exp. m/z**[c]	**Δppm**[d]	**SM$_R$H**[e]	**SM$_R$O**[e]	**SM$_P$H**[e]	**SM$_P$O**[e]	**TF$_R$H**[e]	**TF$_R$O**[e]	**TF$_P$H**[e]	**TF$_P$O**[e]
1489	PA(O-16:0/18:4)	$C_{37}H_{67}O_7P$	[M+H]+	655.46972	655.46981	-0.14	9.1E+06	1.0E+07	2.2E+07	5.3E+06		7.1E+06	1.1E+07	
1490	Hexahydroxy-methoxyflavone neohesperidoside	$C_{28}H_{32}O_{18}$	[M+H]+	657.16614	657.16601	0.20					3.8E+06			
1491	Enalkiren	$C_{35}H_{56}N_6O_6$	[M+H]+	657.43341	657.43378	-0.56	6.5E+06							
1492	PA(O-16:0/18:3)	$C_{37}H_{69}O_7P$	[M+H]+	657.48537	657.48548	-0.18	1.6E+07	1.3E+07	2.3E+07	6.0E+06		9.6E+06	1.4E+07	
1493	PA(O-16:0/18:2)	$C_{37}H_{71}O_7P$	[M+H]+	659.50102	659.50116	-0.21	2.3E+07	1.5E+07	2.2E+07	8.2E+06	4.0E+06	1.2E+07	1.7E+07	4.3E+06
1494	DG(15:0/22:1/0:0)	$C_{40}H_{76}O_5$	[M+Na]+	659.55850	659.55849	0.01						3.9E+06		
1495	17:1 Cholesteryl ester	$C_{44}H_{76}O_2$	[M+Na]+	659.57375	659.57381	-0.09						7.0E+06	4.3E+06	
1496	Purpureacin-1	$C_{37}H_{66}O_8$	[M+Na]+	661.46499	661.46503	-0.07	4.6E+06		4.3E+06			3.8E+06		
1497	PA(O-16:0/18:1)	$C_{37}H_{73}O_7P$	[M+H]+	661.51667	661.51680	-0.20	1.4E+07	6.3E+06	9.5E+06			5.2E+06	8.4E+06	
1498	2-Octaprenylphenol	$C_{46}H_{70}O$	[M+Na]+	661.53189	661.53200	-0.17	8.1E+06	1.3E+07	1.3E+07	5.6E+06		1.8E+07	1.1E+07	6.9E+06
1499	Mosesin 4	$C_{35}H_{60}O_{10}$	[M+Na]+	663.40787	663.40811	-0.36	1.4E+07		1.3E+07		7.4E+06		8.1E+06	
1500	DG(16:0/22:6/0:0)	$C_{41}H_{68}O_5$	[M+Na]+	663.49590	663.49587	0.04	3.5E+06							
1501	PA(16:0e/18:0)	$C_{37}H_{75}O_7P$	[M+H]+	663.53232	663.53237	-0.08	5.9E+06	3.7E+06	5.8E+06		4.0E+06	4.6E+06	5.7E+06	
1502	16:0 Cholesteryl ester	$C_{43}H_{76}O_2$	[M+K]+	663.54769	663.54766	0.05			3.8E+06					
1503	DG(16:0/22:5/0:0)	$C_{41}H_{70}O_5$	[M+Na]+	665.51155	665.51177	-0.33		4.5E+06	4.4E+06			5.2E+06		
1504	Squalamine	$C_{34}H_{65}N_3O_5S$	[M+K]+	666.42765	666.42713	0.78	6.8E+06				4.3E+06		6.5E+06	
1505	PI(22:6/0:0)	$C_{31}H_{49}O_{12}P$	[M+Na]+	667.28538	667.28484	0.82			5.5E+06				6.3E+06	
1506	DG(16:0/22:4/0:0)	$C_{41}H_{72}O_5$	[M+Na]+	667.52720	667.52724	-0.07			3.9E+06					
1507	Finaconitine	$C_{33}H_{46}N_2O_{10}$	[M+K]+	669.27841	669.27786	0.81							4.8E+06	
1508	(O-galactopyranosyl)-keto-triacontanetriol	$C_{36}H_{70}O_9$	[M+Na]+	669.49120	669.49133	-0.19	5.5E+06							
1509	18:2 Cholesteryl ester	$C_{45}H_{76}O_2$	[M+Na]+	671.57375	671.57358	0.26						5.6E+06		
1510	Galactosylceramide	$C_{38}H_{73}NO_8$	[M+H]+	672.54089	672.54095	-0.08		4.3E+06				4.9E+06		
1511	Thapsigargin	$C_{34}H_{50}O_{12}$	[M+Na]+	673.31945	673.31965	-0.29	1.1E+07		1.1E+07		8.2E+06		9.4E+06	
1512	18:1 Cholesteryl ester	$C_{45}H_{78}O_2$	[M+Na]+	673.58940	673.58936	0.06		5.1E+06		1.7E+06		7.1E+06		4.3E+06
1513	N-Acetyl-leu-leu-leu-leu-tyr-amide	$C_{35}H_{58}N_6O_7$	[M+H]+	675.44397	675.44440	-0.63	4.2E+06							
1514	DG(17:1/22:6/0:0)	$C_{42}H_{68}O_5$	[M+Na]+	675.49590	675.49605	-0.23	5.6E+06	4.0E+06						
1515	DG(17:0/22:6/0:0)	$C_{42}H_{70}O_5$	[M+Na]+	677.51155	677.51172	-0.26	7.7E+06							
1516	PA(O-16:0/19:0)	$C_{38}H_{77}O_7P$	[M+H]+	677.54797	677.54766	0.45			3.7E+06					
1517	PG(12:0/17:1)	$C_{35}H_{67}O_{10}P$	[M+H]+	679.45446	679.45446	0.01	5.9E+06	3.9E+06			4.4E+06			

				ESI(+) FT-ICR MS									
**No.**	**Plausible Compound[a]**	**Molecular formula (M)**	**Ion**	**Theor m/z[b]**	**Exp. m/z[c]**	**Δppm[d]**	**SM_RH^e**	**SM_RO^e**	**SM_PH^e**	**SM_PO^e**	**TF_RH^e**	**TF_RO^e**	**TF_PH^e**	**TF_PO^e**
1518	PI(21:0/0:0)	$C_{30}H_{59}O_{12}P$	[M+K]+	681.33757	681.33694	0.94			1.2E+07				1.4E+07	
1519	PG(12:0/17:0)	$C_{35}H_{69}O_{10}P$	[M+H]+	681.47011	681.47028	-0.25	4.8E+06							
1520	Mannosyl-1beta-phosphomycoketide C30	$C_{36}H_{73}O_9P$	[M+H]+	681.50650	681.50650	0.00	4.0E+06	5.1E+06						
1521	PA(O-16:0/20:3)	$C_{39}H_{73}O_7P$	[M+H]+	685.51667	685.51664	0.04			3.6E+06					
1522	DG(15:0/24:1/0:0)	$C_{42}H_{80}O_5$	[M+Na]+	687.58980	687.58948	0.46						3.6E+06		
1523	PA(O-16:0/20:1)	$C_{39}H_{77}O_7P$	[M+H]+	689.54797	689.54790	0.10			3.8E+06				4.2E+06	
1524	DG(15:0/24:0/0:0)	$C_{42}H_{82}O_5$	[M+Na]+	689.60545	689.60530	0.21						3.6E+06		
1525	Peonidin 3-glucoside-5-(6''-acetylglucoside)	$C_{30}H_{35}O_{17}$	[M+Na]+	690.17665	690.17641	0.34	4.9E+06		4.6E+06		5.4E+06			
1526	PG(P-16:0/15:1)	$C_{37}H_{71}O_9P$	[M+H]+	691.49085	691.49104	-0.28						3.6E+06		
1527	PA(O-16:0/20:0)	$C_{39}H_{79}O_7P$	[M+H]+	691.56362	691.56372	-0.15			3.8E+06				4.2E+06	
1528	Monensin	$C_{36}H_{62}O_{11}$	[M+Na]+	693.41843	693.41864	-0.30	1.9E+08		1.2E+08	5.4E+06	7.8E+07		8.2E+07	
1529	PG(12:0/18:1)	$C_{36}H_{69}O_{10}P$	[M+H]+	693.47011	693.47027	-0.23		4.5E+06			6.2E+06			
1530	PG(12:0/18:0)	$C_{36}H_{71}O_{10}P$	[M+H]+	695.48576	695.48586	-0.14	3.7E+06							
1531	Mannosyl-phosphomycoketide C31	$C_{37}H_{75}O_9P$	[M+H]+	695.52215	695.52197	0.25		4.1E+06						
1532	GalCer(d18:1/16:0)	$C_{40}H_{77}NO_8$	[M+H]+	700.57219	700.57206	0.19						4.3E+06		
1533	PA(14:0/22:1)	$C_{39}H_{75}O_8P$	[M+H]+	703.52723	703.52741	-0.25	5.8E+06							
1534	DG(16:0/24:0/0:0)	$C_{43}H_{84}O_5$	[M+Na]+	703.62110	703.62106	0.05						4.0E+06		
1535	DG(19:0/22:6/0:0)	$C_{44}H_{74}O_5$	[M+Na]+	705.54285	705.54269	0.23	7.3E+06						4.0E+06	
1536	SM(d18:0/16:0)	$C_{39}H_{81}N_2O_6P$	[M+H]+	705.59050	705.59063	-0.18		3.9E+06						
1537	PG(12:0/19:1)	$C_{37}H_{71}O_{10}P$	[M+H]+	707.48576	707.48588	-0.16	7.6E+06	6.4E+06			9.8E+06	5.9E+06	4.3E+06	
1538	Mannosyl-1beta-phosphomycoketide C32	$C_{38}H_{77}O_9P$	[M+H]+	709.53780	709.53798	-0.25		5.0E+06				4.0E+06		
1539	O-alpha-D-glucopyranosyl-(tetradecanoyloxy)-eicosanol	$C_{40}H_{78}O_8$	[M+Na]+	709.55889	709.55884	0.07	6.2E+06	4.1E+06	9.5E+06		4.0E+06		1.2E+07	
1540	Dihexadecanoyl-sn-glycero-3-O-(N,N,N-trimethyl)-homoserine	$C_{42}H_{81}NO_7$	[M+H]+	712.60858	712.60865	-0.10	4.2E+06		4.7E+06					
1541	(O-D-glucopyranosyl)-keto-dotriacontanetriol	$C_{38}H_{74}O_9$	[M+K]+	713.49644	713.49639	0.07	5.8E+06							
1542	Scillaren A	$C_{36}H_{52}O_{13}$	[M+Na]+	715.33001	715.33019	-0.24	1.6E+07		1.5E+07		8.6E+06		8.7E+06	
1543	PA(O-16:0/22:1)	$C_{41}H_{81}O_7P$	[M+H]+	717.57927	717.57939	-0.17	4.2E+06		4.2E+06					
1544	PA(O-16:0/22:0)	$C_{41}H_{83}O_7P$	[M+H]+	719.59492	719.59505	-0.18	4.4E+06		4.3E+06				4.4E+06	
1545	Cyanidin 3-(6''-malonylglucoside)-5-glucoside	$C_{30}H_{33}O_{19}$	[M+Na]+	720.15082	720.15055	0.38	4.7E+06		4.9E+06		4.3E+06			

				ESI(+) FT-ICR MS									
No.	Plausible Compound[a]	Molecular formula (M)	Ion	Theor m/z[b]	Exp. m/z[c]	Δppm[d]	SM$_{R}$H[e]	SM$_{R}$O[e]	SM$_{P}$H[e]	SM$_{P}$O[e]	TF$_{R}$H[e]	TF$_{R}$O[e]	TF$_{P}$H[e]	TF$_{P}$O[e]
1546	PG(12:0/20:1)	$C_{38}H_{73}O_{10}P$	[M+H]+	721.50141	721.50152	-0.15	7.0E+06	7.3E+06	4.2E+06		8.8E+06		6.1E+06	
1547	PG(12:0/20:0)	$C_{38}H_{75}O_{10}P$	[M+H]+	723.51706	723.51726	-0.27	3.9E+06							
1548	PA(O-20:0/17:2)	$C_{40}H_{77}O_{7}P$	[M+Na]+	723.52991	723.53056	-0.90	9.5E+06	4.0E+07	2.3E+07	1.3E+07	8.7E+06	2.1E+07	2.5E+07	1.8E+07
1549	Crasseride 2a	$C_{41}H_{80}O_{8}$	[M+Na]+	723.57454	723.57455	-0.01	4.2E+06		7.7E+06			4.2E+06	1.1E+07	
1550	PC(12:0/18:2)	$C_{38}H_{72}NO_{8}P$	[M+Na]+	724.48878	724.48941	-0.88		1.4E+07						
1551	PG(13:0/18:3)	$C_{37}H_{67}O_{10}P$	[M+Na]+	725.43641	725.43582	0.81	4.4E+06							
1552	Ecdysone palmitate	$C_{43}H_{74}O_{7}$	[M+Na]+	725.53268	725.53276	-0.12		4.7E+06	4.2E+06					
1553	DG(18:0/24:1/0:0)	$C_{45}H_{86}O_{5}$	[M+Na]+	729.63675	729.63677	-0.03						3.7E+06		
1554	PA(O-18:0/20:5)	$C_{41}H_{73}O_{7}P$	[M+Na]+	731.49861	731.49813	0.66	6.5E+06							
1555	PA(16:0/22:1)	$C_{41}H_{79}O_{8}P$	[M+H]+	731.55853	731.55870	-0.23	5.4E+06							
1556	PA(O-20:0/19:1)	$C_{42}H_{83}O_{7}P$	[M+H]+	731.59492	731.59486	0.08			4.6E+06					
1557	PG(13:0/20:2)	$C_{39}H_{73}O_{10}P$	[M+H]+	733.50141	733.50132	0.12	5.1E+06							
1558	DG(21:0/22:6/0:0)	$C_{46}H_{78}O_{5}$	[M+Na]+	733.57415	733.57418	-0.04	5.3E+06	4.2E+06	4.1E+06				3.8E+06	
1559	PA(O-18:0/21:0)	$C_{42}H_{85}O_{7}P$	[M+H]+	733.61057	733.61058	-0.02			4.6E+06					
1560	Digitalin	$C_{36}H_{56}O_{14}$	[M+Na]+	735.35623	735.35639	-0.22	4.9E+06				4.2E+06			
1561	PG(13:0/20:1)	$C_{39}H_{75}O_{10}P$	[M+H]+	735.51706	735.51726	-0.26	6.9E+06	7.1E+06	4.1E+06		5.8E+06	5.0E+06	4.5E+06	
1562	PA(O-16:0/22:2)	$C_{41}H_{79}O_{7}P$	[M+Na]+	737.54556	737.54622	-0.89				6.6E+06				1.1E+07
1563	O-glucopyranosyl-(hexadecanoyloxy)-eicosanol	$C_{42}H_{82}O_{8}$	[M+Na]+	737.59019	737.59014	0.07	4.9E+06		8.8E+06			4.5E+06	9.9E+06	
1564	ADP-Ribosyl-L-arginine	$C_{21}H_{35}N_{9}O_{15}P_{2}$	[M+Na]+	738.16201	738.16164	0.50	3.8E+06				4.0E+06			
1565	Chalcomycin	$C_{35}H_{56}O_{14}$	[M+K]+	739.33017	739.33009	0.10	4.3E+06							
1566	PG(12:0/20:3)	$C_{38}H_{69}O_{10}P$	[M+Na]+	739.45206	739.45156	0.67	1.1E+07		8.9E+06					
1567	PC(P-14:0/18:1)	$C_{40}H_{79}NO_{7}P$	[M+Na]+	739.54864	739.54860	0.05							4.1E+06	
1568	Glc-Cer(d18:1/18:0)	$C_{42}H_{79}NO_{9}$	[M+H]+	742.58276	742.58271	0.07	5.4E+06							
1569	Butanoyloxyfucoxanthin	$C_{46}H_{64}O_{8}$	[M+H]+	745.46740	745.46792	-0.70		1.9E+07		5.8E+06		1.0E+07		5.1E+06
1570	DG(22:1/22:6/0:0)	$C_{47}H_{78}O_{5}$	[M+Na]+	745.57415	745.57422	-0.10	3.9E+06		4.3E+06			4.4E+06	6.3E+06	
1571	PA(O-18:0/22:1)	$C_{43}H_{85}O_{7}P$	[M+H]+	745.61057	745.61090	-0.44			3.8E+06				3.8E+06	
1572	TG(14:0/14:0/14:0)	$C_{45}H_{86}O_{6}$	[M+Na]+	745.63166	745.63150	0.22		3.7E+06				5.0E+06		
1573	PI(12:0/14:1)	$C_{35}H_{65}O_{13}P$	[M+Na]+	747.40550	747.40604	-0.72				2.6E+06		4.7E+06		8.9E+06
1574	PG(12:0/22:2)	$C_{40}H_{75}O_{10}P$	[M+H]+	747.51706	747.51705	0.02			3.6E+06					

ESI(+) FT-ICR MS															
**No.**	**Plausible Compound**[a]	**Molecular formula (M)**	**Ion**	**Theor m/z**[b]	**Exp. m/z**[c]	**Δppm**[d]	**SM$_R$H**[e]	**SM$_R$O**[e]	**SM$_P$H**[e]	**SM$_P$O**[e]	**TF$_R$H**[e]	**TF$_R$O**[e]	**TF$_P$H**[e]	**TF$_P$O**[e]
1575	DG(20:5/24:1/0:0)	$C_{47}H_{80}O_5$	[M+Na]+	747.58980	747.59001	-0.29	4.4E+06		4.6E+06				5.2E+06	
1576	Hexadecanoyl-(octadecenoyl)-sn-glycero-phospho-sn-glycerol	$C_{40}H_{77}O_{10}P$	[M+H]+	749.53271	749.53286	-0.19	6.8E+06	6.5E+06	4.6E+06			6.0E+06	5.6E+06	
1577	PG(12:0/22:0)	$C_{40}H_{79}O_{10}P$	[M+H]+	751.54836	751.54865	-0.38	3.7E+06	3.3E+06	3.9E+06				4.0E+06	
1578	PA(18:0/22:2)	$C_{43}H_{81}O_8P$	[M+H]+	757.57418	757.57411	0.10			3.6E+06					
1579	PA(O-20:0/20:5)	$C_{43}H_{77}O_7P$	[M+Na]+	759.52991	759.52940	0.67	4.9E+06							
1580	PA(18:0/22:1)	$C_{43}H_{83}O_8P$	[M+H]+	759.58983	759.58995	-0.16	7.6E+06	6.8E+06	9.9E+06			6.8E+06	9.2E+06	
1581	PA(P-20:0/21:0)	$C_{44}H_{87}O_7P$	[M+H]+	759.62622	759.62604	0.23			3.7E+06					
1582	PA(O-18:0/22:4)	$C_{43}H_{79}O_7P$	[M+Na]+	761.54556	761.54513	0.57	7.1E+06							
1583	PA(18:0/22:0)	$C_{43}H_{85}O_8P$	[M+H]+	761.60548	761.60559	-0.14	8.8E+06	6.3E+06	9.2E+06	4.0E+06		6.9E+06	9.3E+06	
1584	PG(13:0/22:1)	$C_{41}H_{79}O_{10}P$	[M+H]+	763.54836	763.54849	-0.17	6.1E+06	4.5E+06	4.1E+06		3.7E+06	4.5E+06	4.5E+06	
1585	PG(P-18:0/17:2)	$C_{41}H_{77}O_9P$	[M+Na]+	767.51974	767.52041	-0.87	6.8E+07		8.9E+07		3.3E+07			
1586	GalCer(d16:1/23:0)	$C_{45}H_{87}NO_8$	[M+H]+	770.65045	770.65021	0.31	3.9E+06							
1587	PA(19:0/22:2)	$C_{44}H_{83}O_8P$	[M+H]+	771.58983	771.58993	-0.12			5.8E+06			6.4E+06	6.0E+06	
1588	DG(22:6/24:1/0:0)	$C_{49}H_{82}O_5$	[M+Na]+	773.60545	773.60556	-0.15	1.1E+07	1.1E+07	1.6E+07	3.0E+06		1.1E+07	1.6E+07	
1589	PA(O-20:0/22:1)	$C_{45}H_{89}O_7P$	[M+H]+	773.64187	773.64195	-0.11			3.4E+06					
1590	DG(22:5/24:1/0:0)	$C_{49}H_{84}O_5$	[M+Na]+	775.62110	775.62119	-0.12	1.1E+07	1.0E+07	1.4E+07			1.0E+07	1.4E+07	
1591	Chikusetsusaponin Ia	$C_{41}H_{70}O_{12}$	[M+Na]+	777.47595	777.47592	0.04	3.8E+06							
1592	PG(14:0/22:1)	$C_{42}H_{81}O_{10}P$	[M+H]+	777.56401	777.56419	-0.22	5.1E+06	3.9E+06			3.4E+06		4.5E+06	
1593	PI(12:0/18:1)	$C_{39}H_{73}O_{13}P$	[M+H]+	781.48616	781.48618	-0.03		5.6E+06						
1594	Hexadecanoyl-(octadecenoyl)-sn-glycero-phosphocholine	$C_{42}H_{83}NO_8P$	[M+Na]+	783.57485	783.57444	0.52		4.1E+06						
1595	PA(20:1/22:2)	$C_{45}H_{83}O_8P$	[M+H]+	783.58983	783.58985	-0.02							3.7E+06	
1596	PA(20:0/22:2)	$C_{45}H_{85}O_8P$	[M+H]+	785.60548	785.60555	-0.08	1.2E+07	1.0E+07	1.6E+07	2.6E+06		1.2E+07	1.7E+07	
1597	PA(20:0/22:1)	$C_{45}H_{87}O_8P$	[M+H]+	787.62113	787.62116	-0.03	2.7E+07	2.1E+07	3.5E+07	5.7E+06	4.4E+06	2.4E+07	3.5E+07	5.3E+06
1598	PA(20:0/22:0)	$C_{45}H_{89}O_8P$	[M+H]+	789.63678	789.63685	-0.08	2.1E+07	1.5E+07	2.6E+07	7.9E+06	4.9E+06	1.8E+07	2.4E+07	4.4E+06
1599	PG(P-20:0/17:2)	$C_{43}H_{81}O_9P$	[M+Na]+	795.55104	795.55142	-0.48			4.8E+06					
1600	GalCer(d18:2/23:0)	$C_{47}H_{89}NO_8$	[M+H]+	796.66610	796.66605	0.06	3.5E+06							
1601	TG(16:1/14:0/16:1)	$C_{49}H_{90}O_6$	[M+Na]+	797.66296	797.66290	0.08	5.3E+06	1.8E+07	8.1E+06	4.3E+06		2.5E+07	1.1E+07	8.0E+06
1602	GalCer(d18:1/23:0)	$C_{47}H_{91}NO_8$	[M+H]+	798.68175	798.68169	0.07	3.3E+06							

				ESI(+) FT-ICR MS										
**No.**	**Plausible Compound[a]**	**Molecular formula (M)**	**Ion**	**Theor m/z[b]**	**Exp. m/z[c]**	**Δppm[d]**	**SM_RH[e]**	**SM_RO[e]**	**SM_PH[e]**	**SM_PO[e]**	**TF_RH[e]**	**TF_RO[e]**	**TF_PH[e]**	**TF_PO[e]**
1603	PA(21:0/22:2)	$C_{46}H_{87}O_8P$	[M+H]+	799.62113	799.62124	-0.13	1.7E+07	1.4E+07	2.7E+07	4.2E+06	3.7E+06	1.7E+07	2.5E+07	4.5E+06
1604	TG(16:0/14:0/16:1)	$C_{49}H_{92}O_6$	[M+Na]+	799.67861	799.67858	0.04	7.2E+06	2.6E+07	9.7E+06	5.0E+06			1.5E+07	1.3E+07
1605	MGDG(18:2/18:2)	$C_{45}H_{78}O_{10}$	[M+Na]+	801.54872	801.54897	-0.31	1.0E+07		8.7E+06		5.7E+06			
1606	PA(21:0/22:1)	$C_{46}H_{89}O_8P$	[M+H]+	801.63678	801.63694	-0.19	3.6E+07	2.9E+07	5.0E+07	6.6E+06	6.6E+06	3.4E+07	4.9E+07	7.5E+06
1607	Dodecanoyl-hexadecanoyl-octadecanoyl-sn-glycerol	$C_{49}H_{94}O_6$	[M+Na]+	801.69426	801.69437	-0.14	4.3E+06	1.3E+07	3.7E+06	3.8E+06		1.9E+07	8.4E+06	7.2E+06
1608	PE(16:0/22:6)	$C_{43}H_{74}NO_9P$	[M+Na]+	802.49934	802.49989	-0.69		4.3E+07	3.6E+06			4.4E+06	4.4E+06	
1609	PA(21:0/22:0)	$C_{46}H_{91}O_8P$	[M+H]+	803.65243	803.65260	-0.21	2.6E+07	1.9E+07	3.2E+07	5.9E+06	5.3E+06	2.1E+07	3.1E+07	6.5E+06
1610	PE(20:5/22:6)	$C_{47}H_{72}NO_8P$	[M+H]+	810.50683	810.50692	-0.11	3.1E+07		8.5E+06		1.4E+07		9.9E+06	
1611	PS(14:0/22:2)	$C_{42}H_{78}NO_{10}P$	[M+Na]+	810.52556	810.52628	-0.89	4.2E+07		2.6E+07		1.4E+07		1.4E+07	
1612	PA(22:1/22:2)	$C_{47}H_{87}O_8P$	[M+H]+	811.62113	811.62131	-0.22	8.3E+06	7.7E+06	1.2E+07			7.6E+06	1.2E+07	
1613	PE(20:4/22:6)	$C_{47}H_{74}NO_8P$	[M+H]+	812.52248	812.52240	0.10							3.6E+06	
1614	PA(22:0/22:2)	$C_{47}H_{89}O_8P$	[M+H]+	813.63678	813.63692	-0.17	3.4E+07	3.0E+07	4.8E+07	6.2E+06	5.6E+06	3.4E+07	4.7E+07	6.6E+06
1615	Leucomycin A4	$C_{41}H_{67}NO_{15}$	[M+H]+	814.45835	814.45855	-0.25							4.4E+06	
1616	PA(22:0/22:0)	$C_{47}H_{93}O_8P$	[M+H]+	817.66808	817.66821	-0.15	3.5E+07	2.5E+07	4.1E+07	7.8E+06	7.2E+06	2.8E+07	3.9E+07	7.3E+06
1617	Diginatin	$C_{41}H_{64}O_{15}$	[M+Na]+	819.41374	819.41403	-0.35	9.9E+06		7.2E+06		5.0E+06			
1618	PG(O-18:0/22:0)	$C_{46}H_{93}O_9P$	[M+H]+	821.66300	821.66284	0.19						4.3E+06		
1619	Spongipregnoloside D	$C_{40}H_{64}O_{16}$	[M+Na]+	823.40866	823.40889	-0.28	3.9E+06							
1620	TG(16:1/14:0/18:2)	$C_{51}H_{92}O_6$	[M+Na]+	823.67861	823.67851	0.13	4.6E+06	1.4E+07	6.5E+06	2.9E+06		1.7E+07	9.0E+06	5.9E+06
1621	TG(16:0/14:0/18:2)	$C_{51}H_{94}O_6$	[M+Na]+	825.69426	825.69422	0.05	9.7E+06	3.2E+07	1.3E+07	5.0E+06		4.4E+07	1.9E+07	1.5E+07
1622	Glucosylceramide (d18:1/25:0)	$C_{49}H_{95}NO_8$	[M+H]+	826.71305	826.71309	-0.05	3.9E+06		4.0E+06					
1623	PI(P-16:0/17:2)	$C_{42}H_{77}O_{12}P$	[M+Na]+	827.50449	827.50393	0.67	5.3E+06							
1624	TG(16:0/14:0/18:1)	$C_{51}H_{96}O_6$	[M+Na]+	827.70991	827.70987	0.05	8.2E+06	3.2E+07	1.1E+07	6.0E+06		4.6E+07	1.7E+07	1.4E+07
1625	TG(16:0/14:0/18:0)	$C_{51}H_{98}O_6$	[M+Na]+	829.72556	829.72549	0.09		1.2E+07	3.7E+06	3.0E+06		1.6E+07	5.7E+06	6.6E+06
1626	TG(16:0/16:0/18:1)	$C_{53}H_{100}O_6$	[M+H]+	833.75927	833.75998	-0.85						3.3E+06		
1627	TG(16:1/16:1/17:2)	$C_{52}H_{92}O_6$	[M+Na]+	835.67861	835.67872	-0.13		3.6E+06				4.9E+06		
1628	TG(16:0/16:1/17:2)	$C_{52}H_{94}O_6$	[M+Na]+	837.69426	837.69436	-0.11	4.8E+06	1.0E+07	5.4E+06	1.9E+06		1.3E+07	9.1E+06	5.9E+06
1629	GalCer(d18:1/26:1)	$C_{50}H_{95}NO_8$	[M+H]+	838.71305	838.71309	-0.05	4.3E+06		5.8E+06					
1630	TG(15:0/18:1/16:1)	$C_{52}H_{96}O_6$	[M+Na]+	839.70991	839.70996	-0.05	6.2E+06	2.2E+07	1.0E+07	4.1E+06		3.1E+07	1.4E+07	1.0E+07
1631	GalCer(d18:0/26:1)	$C_{50}H_{97}NO_8$	[M+H]+	840.72870	840.72879	-0.11	4.1E+06		5.6E+06					

					ESI(+) FT-ICR MS										
**No.**	**Plausible Compound[a]**	**Molecular formula (M)**	**Ion**	**Theor m/z[b]**	**Exp. m/z[c]**	**Δppm[d]**	**SM_RH^e**	**SM_RO^e**	**SM_PH^e**	**SM_PO^e**	**TF_RH^e**	**TF_RO^e**	**TF_PH^e**	**TF_PO^e**
1632	TG(15:0/18:1/16:0)	$C_{52}H_{98}O_6$	[M+Na]+	841.72556	841.72563	-0.08	5.4E+06	1.9E+07	6.8E+06	3.9E+06		2.7E+07	1.4E+07	9.0E+06
1633	TG(16:0/16:0/17:0)	$C_{52}H_{100}O_6$	[M+Na]+	843.74121	843.74131	-0.11		6.1E+06		3.4E-01		9.0E+06		4.5E+06
1634	SM(d18:1/26:0)	$C_{49}H_{100}N_2O_6P$	[M+H]+	844.73918	844.73917	0.01						3.7E+06		
1635	Glycyrrhizinate	$C_{42}H_{62}O_{16}$	[M+Na]+	845.39301	845.39337	-0.42	5.7E+06		5.6E+06					
1636	TG(16:0/14:0/18:0)	$C_{51}H_{98}O_6$	[M+K]+	845.69950	845.69959	-0.11					6.1E+06			
1637	TG(16:0/16:1/18:3)	$C_{53}H_{94}O_6$	[M+Na]+	849.69426	849.69415	0.13		5.8E+06	3.6E+06			7.1E+06		
1638	TG(16:1/16:1)/17:2)	$C_{52}H_{92}O_6$	[M+K]+	851.65255	851.65274	-0.22	4.7E+06		6.3E+06					
1639	TG(16:0/16:0/18:3)	$C_{53}H_{96}O_6$	[M+Na]+	851.70991	851.70990	0.01	5.3E+06	1.7E+07	7.3E+06	2.8E+06		1.9E+07	9.6E+06	6.4E+06
1640	TG(16:0/16:1/17:2)	$C_{52}H_{94}O_6$	[M+K]+	853.66820	853.66815	0.06					3.3E+06			
1641	TG(16:0/16:0/18:2)	$C_{53}H_{98}O_6$	[M+Na]+	853.72556	853.72549	0.09	1.1E+07	2.8E+07	9.9E+06	4.1E+06			1.4E+07	9.7E+06
1642	TG(16:0/16:0/18:1)	$C_{53}H_{100}O_6$	[M+Na]+	855.74121	855.74112	0.11		2.5E+07	5.6E+06	3.5E+06			1.2E+07	8.3E+06
1643	TG(15:0/18:1/16:0)	$C_{52}H_{98}O_6$	[M+K]+	857.69950	857.69949	0.01					8.0E+06			
1644	TG(16:0/16:0/18:0)	$C_{53}H_{102}O_6$	[M+Na]+	857.75686	857.75688	-0.02		4.4E+06				5.7E+06		3.6E+06
1645	TG(16:0/16:0/17:0)	$C_{52}H_{100}O_6$	[M+K]+	859.71515	859.71496	0.22	1.7E+07		2.0E+07		4.1E+06			
1646	TG(16:1/16:1/18:3)	$C_{53}H_{92}O_6$	[M+K]+	863.65255	863.65276	-0.24			4.2E+06				3.4E+06	
1647	TG(15:0/16:0/20:4)	$C_{54}H_{96}O_6$	[M+Na]+	863.70991	863.70987	0.05		4.8E+06	4.3E+06			5.9E+06	4.3E+06	3.1E+06
1648	LacCer(d18:0/16:0)	$C_{46}H_{89}NO_{13}$	[M+H]+	864.64067	864.64020	0.54	3.6E+06							
1649	PG(20:4/22:6)	$C_{48}H_{75}O_{10}P$	[M+Na]+	865.49901	865.49892	0.10	2.1E+07		2.4E+07		2.1E+07		2.8E+07	
1650	TG(15:0/16:0/20:3)	$C_{54}H_{98}O_6$	[M+Na]+	865.72556	865.72540	0.18	3.3E+06	1.0E+07	5.7E+06	1.8E+06		1.3E+07	7.4E+06	5.5E+06
1651	TG(15:0/16:0/20:2)	$C_{54}H_{100}O_6$	[M+Na]+	867.74121	867.74110	0.13	4.3E+06	1.3E+07	6.7E+06	2.5E+06		1.7E+07	9.1E+06	5.9E+06
1652	TG(16:0/16:1/19:0)	$C_{54}H_{102}O_6$	[M+Na]+	869.75686	869.75678	0.09		7.6E+06	4.0E+06	1.7E+06		1.1E+07	4.4E+06	5.6E+06
1653	Pheophytin a	$C_{55}H_{74}N_4O_5$	[M+H]+	871.57320	871.57339	-0.22				1.8E+06				2.8E+06
1654	TG(16:0/16:0/19:0)	$C_{54}H_{104}O_6$	[M+Na]+	871.77251	871.77223	0.32						3.5E+06		
1655	PG(O-20:0/22:6)	$C_{48}H_{85}O_9P$	[M+K]+	875.55628	875.55613	0.17						4.4E+06		
1656	TG(16:0/16:0/20:5)	$C_{55}H_{96}O_6$	[M+Na]+	875.70991	875.71004	-0.15			3.8E+06			5.5E+06		3.0E+06
1657	Ditetradecanoyl-sn-glycero-3-phospho-lyso tetradecanoylglycerol	$C_{48}H_{93}O_{11}P$	[M+H]+	877.65283	877.65309	-0.30	5.7E+06	3.5E+06						
1658	TG(16:0/17:2/18:3)	$C_{54}H_{94}O_6$	[M+K]+	877.66820	877.66819	0.01			3.8E+06					
1659	TG(16:0/16:0/20:4)	$C_{55}H_{98}O_6$	[M+Na]+	877.72556	877.72559	-0.03	4.6E+06	1.2E+07	7.7E+06	2.4E+06		1.2E+07	7.9E+06	5.5E+06

ESI(+) FT-ICR MS

No.	Plausible Compound[a]	Molecular formula (M)	Ion	Theor m/z[b]	Exp. m/z[c]	Δppm[d]	SM$_{R}$H[e]	SM$_{R}$O[e]	SM$_{P}$H[e]	SM$_{P}$O[e]	TF$_{R}$H[e]	TF$_{R}$O[e]	TF$_{P}$H[e]	TF$_{P}$O[e]
1660	TG(16:0/16:0/20:3)	$C_{55}H_{100}O_6$	[M+Na]+	879.74121	879.74115	0.07	8.5E+06	1.8E+07	8.7E+06	3.1E+06		1.9E+07	8.2E+06	6.7E+06
1661	Thevetin B	$C_{42}H_{66}O_{18}$	[M+Na]+	881.41414	881.41436	-0.25	3.6E+06							
1662	TG(15:0/16:0/20:3)	$C_{54}H_{98}O_6$	[M+K]+	881.69950	881.69960	-0.11					3.4E+06			
1663	TG(16:0/16:0/20:2)	$C_{55}H_{102}O_6$	[M+Na]+	881.75686	881.75692	-0.07	1.3E+07	2.1E+07	6.6E+06	2.8E+06		2.2E+07	9.5E+06	6.6E+06
1664	TG(15:0/16:0/20:2)	$C_{54}H_{100}O_6$	[M+K]+	883.71515	883.71506	0.10					4.4E+06			
1665	TG(16:0/16:0/20:1)	$C_{55}H_{104}O_6$	[M+Na]+	883.77251	883.77254	-0.03	1.1E+07	9.5E+06				7.4E+06		3.5E+06
1666	TG(16:0/16:1/19:0)	$C_{54}H_{102}O_6$	[M+K]+	885.73080	885.73116	-0.41					4.9E+06			
1667	TG(16:0/16:0/20:0)	$C_{55}H_{106}O_6$	[M+Na]+	885.78816	885.78809	0.08						3.3E+06		
1668	TG(16:0/16:0/19:0)	$C_{54}H_{104}O_6$	[M+K]+	887.74645	887.74642	0.04	5.1E+06		5.5E+06				5.5E+06	
1669	TG(16:0/17:0/20:5)	$C_{56}H_{98}O_6$	[M+Na]+	889.72556	889.72590	-0.38						3.4E+06		
1670	TG(16:0/16:0/20:5)	$C_{55}H_{96}O_6$	[M+K]+	891.68385	891.68415	-0.34	5.1E+06							
1671	TG(16:0/17:0/20:4)	$C_{56}H_{100}O_6$	[M+Na]+	891.74121	891.74122	-0.01		3.7E+06				4.6E+06		
1672	TG(16:0/16:0/20:4)	$C_{55}H_{98}O_6$	[M+K]+	893.69950	893.69981	-0.35					4.8E+06			
1673	TG(16:0/16:0/20:3)	$C_{55}H_{100}O_6$	[M+K]+	895.71515	895.71526	-0.12					4.6E+06			
1674	TG(16:0/17:0/20:2)	$C_{56}H_{104}O_6$	[M+Na]+	895.77251	895.77245	0.07						4.6E+06		
1675	TG(16:0/16:0/20:2)	$C_{55}H_{102}O_6$	[M+K]+	897.73080	897.73087	-0.08					4.7E+06			
1676	TG(16:0/16:1/21:0)	$C_{56}H_{106}O_6$	[M+Na]+	897.78816	897.78820	-0.04						3.7E+06		
1677	TG(16:0/16:0/20:1)	$C_{55}H_{104}O_6$	[M+K]+	899.74645	899.74642	0.03				3.1E+06				
1678	TG(18:3/18:0/18:3)	$C_{57}H_{98}O_6$	[M+Na]+	901.72556	901.72543	0.15		1.0E+07	5.8E+06			7.0E+06	4.1E+06	3.6E+06
1679	TG(16:0/16:0/20:0)	$C_{55}H_{106}O_6$	[M+K]+	901.76210	901.76215	-0.05	5.7E+06		4.3E+06				4.8E+06	
1680	LacCer(d18:0/16:0)	$C_{46}H_{89}NO_{13}$	[M+K]+	902.59655	902.59596	0.66			3.3E+06					
1681	PC(22:0/22:0)	$C_{52}H_{104}NO_8P$	[M+H]+	902.75723	902.75671	0.58	4.6E+06							
1682	TG(18:2/18:1/18:2)	$C_{57}H_{100}O_6$	[M+Na]+	903.74121	903.74118	0.03	3.5E+06	1.2E+07	6.5E+06			7.6E+06	5.0E+06	4.2E+06
1683	TG(17:2/18:1/18:2)	$C_{56}H_{98}O_6$	[M+K]+	905.69950	905.69969	-0.21			3.5E+06					
1684	TG(18:2/16:0/20:2)	$C_{57}H_{102}O_6$	[M+Na]+	905.75686	905.75680	0.07		1.2E+07	6.3E+06	1.7E+06		8.9E+06	5.4E+06	3.8E+06
1685	TG(17:2/18:1/18:1)	$C_{56}H_{100}O_6$	[M+K]+	907.71515	907.71528	-0.14	4.7E+06		7.7E+06					
1686	TG(18:1/18:1/18:1)	$C_{57}H_{104}O_6$	[M+Na]+	907.77251	907.77250	0.02	8.0E+06	1.5E+07	6.0E+06	1.8E+06		1.2E+07	5.3E+06	3.9E+06
1687	TG(17:2/18:0/18:1)	$C_{56}H_{102}O_6$	[M+K]+	909.73080	909.73080	0.00	6.8E+06		9.6E+06					
1688	TG(18:1/18:0/18:1)	$C_{57}H_{106}O_6$	[M+Na]+	909.78816	909.78808	0.09		6.5E+06				6.3E+06		3.2E+06

				ESI(+) FT-ICR MS											
**No.**	**Plausible Compound**[a]	**Molecular formula (M)**	**Ion**	**Theor m/z**[b]	**Exp. m/z**[c]	**Δppm**[d]	**SM$_R$H**[e]	**SM$_R$O**[e]	**SM$_P$H**[e]	**SM$_P$O**[e]	**TF$_R$H**[e]	**TF$_R$O**[e]	**TF$_P$H**[e]	**TF$_P$O**[e]
1689	TG(17:2/18:0/18:0)	$C_{56}H_{104}O_6$	[M+K]+	911.74645	911.74636	0.10	7.3E+06	6.2E+06	1.0E+07					
1690	TG(18:0/18:0/18:1)	$C_{57}H_{108}O_6$	[M+Na]+	911.80381	911.80355	0.29	6.7E+06	5.6E+06				4.4E+06		
1691	TG(17:1/18:0/18:0)	$C_{56}H_{106}O_6$	[M+K]+	913.76210	913.76204	0.06	6.5E+06		6.9E+06				6.2E+06	
1692	TG(18:0/18:0/18:0)	$C_{57}H_{110}O_6$	[M+Na]+	913.81946	913.81951	-0.05		3.4E+06				3.4E+06		
1693	TG(17:0/18:0/18:0)	$C_{56}H_{108}O_6$	[M+K]+	915.77775	915.77753	0.24	4.1E+06		3.8E+06				4.5E+06	
1694	Galabiosylceramide (d18:1/20:0)	$C_{50}H_{95}NO_{13}$	[M+H]+	918.68762	918.68774	-0.13	4.2E+06							
1695	TG(18:2/18:1/18:2)	$C_{57}H_{100}O_6$	[M+K]+	919.71515	919.71498	0.19					5.0E+06			
1696	TG(18:2/18:2/19:0)	$C_{58}H_{104}O_6$	[M+Na]+	919.77251	919.77250	0.01						3.0E+06		
1697	TG(18:2/16:0/20:2)	$C_{57}H_{102}O_6$	[M+K]+	921.73080	921.73074	0.07					4.1E+06			
1698	TG(18:1/16:0/20:2)	$C_{57}H_{104}O_6$	[M+K]+	923.74645	923.74630	0.16					4.2E+06			
1699	TG(18:0/18:0/18:2)	$C_{57}H_{106}O_6$	[M+K]+	925.76210	925.76225	-0.16			1.0E+07				7.4E+06	
1700	TG(16:1/18:0/21:0)	$C_{58}H_{110}O_6$	[M+Na]+	925.81946	925.81954	-0.08		3.3E+06				4.3E+06		
1701	TG(18:0/18:0/18:1)	$C_{57}H_{108}O_6$	[M+K]+	927.77775	927.77789	-0.15			8.2E+06					
1702	TG(16:0/17:0/22:0)	$C_{58}H_{112}O_6$	[M+Na]+	927.83511	927.83508	0.03						3.3E+06		
1703	TG(18:0/18:0/18:0)	$C_{57}H_{110}O_6$	[M+K]+	929.79340	929.79350	-0.11			4.3E+06					
1704	TG(17:0/17:2/21:0)	$C_{58}H_{108}O_6$	[M+K]+	939.77775	939.77802	-0.29	3.5E+06		4.1E+06				3.9E+06	
1705	TG(18:0/18:0/20:1)	$C_{59}H_{112}O_6$	[M+Na]+	939.83511	939.83521	-0.10						4.2E+06		
1706	TG(18:0/18:1/19:0)	$C_{58}H_{110}O_6$	[M+K]+	941.79340	941.79354	-0.14	4.0E+06		5.6E+06					
1707	TG(16:0/18:0/21:0)	$C_{58}H_{112}O_6$	[M+K]+	943.80905	943.80902	0.03			4.3E+06				3.9E+06	
1708	TG(18:2/18:2/20:0)	$C_{59}H_{106}O_6$	[M+K]+	949.76210	949.76176	0.36			3.4E+06					
1709	TG(18:1/18:1/20:1)	$C_{59}H_{108}O_6$	[M+K]+	951.77775	951.77772	0.03							3.8E+06	
1710	TG(18:0/18:2/21:0)	$C_{60}H_{112}O_6$	[M+Na]+	951.83511	951.83469	0.44						3.4E+06		
1711	Thyroxine glucuronide	$C_{21}H_{19}I_4NO_{10}$	[M+H]+	953.72606	953.72642	-0.37			2.8E+06					
1712	TG(18:2/19:0/19:0)	$C_{59}H_{110}O_6$	[M+K]+	953.79340	953.79320	0.21	5.0E+06		6.5E+06				5.2E+06	
1713	TG(18:1/19:0/19:0)	$C_{59}H_{112}O_6$	[M+K]+	955.80905	955.80873	0.34			8.4E+06					
1714	TG(19:0/19:0/19:0)	$C_{60}H_{116}O_6$	[M+Na]+	955.86641	955.86635	0.07						2.9E+06		
1715	TG(16:0/20:0/20:0)	$C_{59}H_{114}O_6$	[M+K]+	957.82470	957.82449	0.22	4.1E+06		5.2E+06				4.7E+06	
1716	PC(22:0/24:1)	$C_{54}H_{106}NO_8P$	[M+K]+	966.72877	966.72945	-0.71							3.9E+06	
1717	TG(18:0/18:2/21:0)	$C_{60}H_{112}O_6$	[M+K]+	967.80905	967.80881	0.25			5.2E+06				4.5E+06	

					ESI(+) FT-ICR MS									
**No.**	**Plausible Compound[a]**	**Molecular formula (M)**	**Ion**	**Theor m/z[b]**	**Exp. m/z[c]**	**Δppm[d]**	**SM$_R$H[e]**	**SM$_R$O[e]**	**SM$_P$H[e]**	**SM$_P$O[e]**	**TF$_R$H[e]**	**TF$_R$O[e]**	**TF$_P$H[e]**	**TF$_P$O[e]**
1718	PC(22:0/24:0)	$C_{54}H_{108}NO_8P$	[M+K]+	968.74442	968.74507	-0.68							3.4E+06	
1719	TG(18:0/20:1/20:1)	$C_{61}H_{114}O_6$	[M+K]+	981.82470	981.82486	-0.16			4.7E+06				4.4E+06	
1720	Tomatine	$C_{50}H_{83}NO_{21}$	[M+H]+	1034.55304	1034.55351	-0.46			9.7E+06				3.6E+06	
1721	Acetyl-sphingosine-tetraacetyl-GalCer(d18:1/h24:0)	$C_{58}H_{103}NO_{14}$	[M+H]+	1038.74513	1038.74449	0.62							3.5E+06	

[a] Cer: Ceramide; GalCer: Galactosylceramide; GlcCer: Glucosylceramide; LacCer: Lactosylceramide; MG: Monoacylglycerol; DG: Diacylglycerol; TG: Triacylglycerol; MGDG: Monoacyldiacylglycerol; PA:
Phosphatidic acid; PC: Phosphatidylcholine; PE: Phosphatidylethanolamine; PG: Glycerophospholipids; PI: Phosphatidylinositol; PS: Phosphatidylserine;; SM: Sphingomyelin

[b] Theor. stands for calculated exact mass to charge ratio

[c] Exp. stands for experimental m/z value

[d] The error expressed in parts per million (ppm)

[e] SM$_R$H and SM$_R$O stand for hydroalcoholic and organic portions of red San Marzano extracts, respectively; SM$_P$H and SM$_P$O stand for hydroalcoholic and organic portions of pink San Marzano extracts, respectively. TF$_R$H and TF$_R$O stand for hydroalcoholic and organic portions of red Torpedino di Fondi extracts, respectively; TF$_P$H and TF$_P$O stand for hydroalcoholic and organic portions of pink Torpedino di Fondi extracts, respectively.

Table S3: Comprehensive list of metabolites detected in hydroalcoholic and organic fractions of pink and red San Marzano (SM) and Torpedino di Fondi (TF) extracts using ESI(-) FT-ICR MS.

				ESI(-) FTICR-MS										
**No.**	**Plausible compound[a]**	**Molecular formula (M)**	**Ion**	**Theor. m/z[b]**	**Exp. m/z[c]**	**Δppm[d]**	**SM$_R$H[e]**	**SM$_R$O[e]**	**SM$_P$H[e]**	**SM$_P$O[e]**	**TF$_R$H[e]**	**TF$_R$O[e]**	**TF$_P$H[e]**	**TF$_P$O[e]**
1	L-Serine	$C_3H_7NO_3$	[M-H]-	104.03532	104.03540	0.77	1.0E+06	5.6E+06		1.2E+06	1.4E+06		3.2E+06	2.3E+06
2	2-Furoic acid	$C_5H_4O_3$	[M-H]-	111.00877	111.00886	0.81	1.9E+06		1.5E+06				1.4E+07	
3	Oxopentenoic acid	$C_5H_6O_3$	[M-H]-	113.02442	113.02448	0.53							1.4E+06	
4	Maleic acid	$C_4H_4O_4$	[M-H]-	115.00368	115.00376	0.70							2.4E+06	
5	Glutarate semialdehyde	$C_5H_8O_3$	[M-H]-	115.04007	115.04012	0.43		1.3E+06						
6	L-Valine	$C_5H_{11}NO_2$	[M-H]-	116.07170	116.07177	0.60		1.4E+06						
7	Succinic acid	$C_4H_6O_4$	[M-H]-	117.01933	117.01941	0.68		1.1E+06					4.0E+06	
8	D-Threonine	$C_4H_9NO_3$	[M-H]-	118.05097	118.05105	0.68		2.0E+06					1.6E+06	
9	D-Erythrose	$C_4H_8O_4$	[M-H]-	119.03498	119.03508	0.84	2.8E+06				1.3E+06		5.0E+06	
10	o-Hydroxybenzaldehyde	$C_7H_6O_2$	[M-H]-	121.02950	121.02957	0.58		1.1E+06						
11	2-Oxo-4E-hexenoic acid	$C_6H_8O_3$	[M-H]-	127.04007	127.04017	0.79							1.2E+06	
12	Pyroglutamic acid	$C_5H_7NO_3$	[M-H]-	128.03532	128.03540	0.62	1.4E+07	3.2E+07	1.5E+06	5.5E+06	1.1E+07	1.2E+07	3.9E+07	1.5E+07
13	2-Oxoglutarate semialdehyde	$C_5H_6O_4$	[M-H]-	129.01933	129.01942	0.70							1.5E+06	
14	3-Oxohexanoic acid	$C_6H_{10}O_3$	[M-H]-	129.05572	129.05580	0.62		1.4E+06						1.1E+06
15	Octanol	$C_8H_{18}O$	[M-H]-	129.12849	129.12858	0.70							1.0E+06	
16	L-Leucine	$C_6H_{13}NO_2$	[M-H]-	130.08735	130.08743	0.61		3.2E+06					1.1E+06	
17	Glutaric acid	$C_5H_8O_4$	[M-H]-	131.03498	131.03506	0.61		1.2E+06					3.4E+06	
18	L-Asparagine	$C_4H_8N_2O_3$	[M-H]-	131.04622	131.04630	0.61							3.3E+06	
19	L-Aspartic acid	$C_4H_7NO_4$	[M-H]-	132.03023	132.03032	0.68	1.0E+07	1.3E+06	3.0E+06		2.1E+06		3.0E+07	

				ESI(-) FTICR-MS										
No.	Plausible compound[a]	Molecular formula (M)	Ion	Theor. m/z[b]	Exp. m/z[c]	Δppm[d]	SM_RH[e]	SM_RO[e]	SM_PH[e]	SM_PO[e]	TF_RH[e]	TF_RO[e]	TF_PH[e]	TF_PO[e]
20	Malic acid	$C_4H_6O_5$	[M-H]-	133.01425	133.01434	0.68	3.5E+06		5.9E+06		1.4E+06		3.0E+07	
21	Threonic acid	$C_4H_8O_5$	[M-H]-	135.02990	135.03000	0.74							1.8E+06	
22	4-Nitroaniline	$C_6H_6N_2O_2$	[M-H]-	137.03565	137.03573	0.58		7.2E+06		1.9E+06	2.5E+06	2.7E+06	1.6E+07	4.2E+06
23	Dimethylmaleic acid	$C_6H_8O_4$	[M-H]-	143.03498	143.03507	0.63	2.0E+06	1.5E+06			1.3E+06		3.9E+06	1.3E+06
24	Oxoglutaric acid	$C_5H_6O_5$	[M-H]-	145.01425	145.01435	0.69							1.3E+06	
25	Methylglutaric acid	$C_6H_{10}O_4$	[M-H]-	145.05063	145.05072	0.62	2.2E+06	9.9E+06		4.8E+06	2.5E+06	5.5E+06	1.5E+07	1.0E+07
26	L-Glutamine	$C_5H_{10}N_2O_3$	[M-H]-	145.06187	145.06197	0.69	2.9E+06		2.0E+06		1.3E+06		1.9E+07	
27	L-Glutamic acid	$C_5H_9NO_4$	[M-H]-	146.04588	146.04597	0.62	2.8E+07	1.6E+06	5.7E+06		3.4E+06		6.8E+07	1.8E+06
28	Hydroxyglutarate	$C_5H_8O_5$	[M-H]-	147.02990	147.02998	0.54							2.8E+06	
29	2-Deoxyribonic acid	$C_5H_{10}O_5$	[M-H]-	149.04555	149.04565	0.67	1.4E+06						2.5E+06	
30	Diethylphosphoric acid	$C_4H_{11}O_4P$	[M-H]-	153.03222	153.03230	0.52							1.0E+06	
31	L-Histidine	$C_6H_9N_3O_2$	[M-H]-	154.06220	154.06228	0.52							1.6E+06	
32	Isopropylmaleic acid	$C_7H_{10}O_4$	[M-H]-	157.05063	157.05071	0.51		2.4E+06					2.0E+06	1.4E+06
33	Oxooctanoic acid	$C_8H_{14}O_3$	[M-H]-	157.08702	157.08709	0.45		4.0E+06		1.5E+06		1.6E+06		2.3E+06
34	Methyl-octanoic acid	$C_9H_{18}O_2$	[M-H]-	157.12340	157.12348	0.51		1.4E+06			1.1E+06	1.1E+06	1.7E+06	1.3E+06
35	Pimelic acid	$C_7H_{12}O_4$	[M-H]-	159.06628	159.06637	0.57		4.7E+06		2.0E+06	1.9E+06	2.1E+06	4.3E+06	4.4E+06
36	N-Methyl-L-glutamic acid	$C_6H_{11}NO_4$	[M-H]-	160.06153	160.06159	0.37							1.1E+06	
37	Hydroxyadipic acid	$C_6H_{10}O_5$	[M-H]-	161.04555	161.04563	0.50	4.2E+06	1.6E+06	1.5E+06		2.5E+06		1.1E+07	1.8E+06
38	L-Phenylalanine	$C_9H_{11}NO_2$	[M-H]-	164.07170	164.07178	0.49		2.1E+06		1.3E+06	1.2E+06		1.4E+06	1.7E+06
39	Formylsalicylic acid	$C_8H_6O_4$	[M-H]-	165.01933	165.01937	0.24							1.8E+06	
40	D-Ribonic acid	$C_5H_{10}O_6$	[M-H]-	165.04046	165.04054	0.48					1.2E+06		4.8E+06	

				ESI(-) FTICR-MS										
**No.**	**Plausible compound[a]**	**Molecular formula (M)**	**Ion**	**Theor. m/z[b]**	**Exp. m/z[c]**	**Δppm[d]**	**SM$_R$H[e]**	**SM$_R$O[e]**	**SM$_P$H[e]**	**SM$_P$O[e]**	**TF$_R$H[e]**	**TF$_R$O[e]**	**TF$_P$H[e]**	**TF$_P$O[e]**
41	Uric acid	$C_5H_4N_4O_3$	[M-H]-	167.02106	167.02115	0.54							1.2E+06	
42	N-Cyclopropylammelide	$C_6H_8N_4O_2$	[M-H]-	167.05745	167.05731	-0.84							1.3E+06	
43	2-Octenedioic acid	$C_8H_{12}O_4$	[M-H]-	171.06628	171.06637	0.53		1.3E+06					1.4E+06	1.4E+06
44	Oxo-nonanoic acid	$C_9H_{16}O_3$	[M-H]-	171.10267	171.10276	0.53		3.1E+06		1.4E+06		1.3E+06	1.4E+06	1.9E+06
45	Decanoic acid	$C_{10}H_{20}O_2$	[M-H]-	171.13905	171.13914	0.53		1.2E+06					1.1E+06	1.2E+06
46	N-Acetyl-L-leucine	$C_8H_{15}NO_3$	[M-H]-	172.09792	172.09799	0.41		1.0E+06						
47	Dehydroascorbic acid	$C_6H_6O_6$	[M-H]-	173.00916	173.00924	0.46	4.3E+06		1.4E+06		2.4E+06		2.6E+07	
48	Shikimic acid	$C_7H_{10}O_5$	[M-H]-	173.04555	173.04564	0.52					1.6E+06		3.2E+06	1.4E+06
49	2-Propylglutaric acid	$C_8H_{14}O_4$	[M-H]-	173.08193	173.08200	0.40	1.5E+06	5.7E+06		3.0E+06	1.8E+06	3.9E+06	4.8E+06	6.3E+06
50	Hydroxy pelargonic acid	$C_9H_{18}O_3$	[M-H]-	173.11832	173.11840	0.46		1.1E+06					1.3E+06	1.2E+06
51	N-Formyl-L-glutamic acid	$C_6H_9NO_5$	[M-H]-	174.04080	174.04084	0.23		1.0E+06					9.7E+05	
52	Argininic acid	$C_6H_{13}N_3O_3$	[M-H]-	174.08841	174.08850	0.52		7.0E+06		3.2E+06	1.6E+06	5.1E+06	2.3E+06	4.6E+06
53	Ascorbic acid	$C_6H_8O_6$	[M-H]-	175.02481	175.02488	0.40	3.0E+06		1.5E+06				1.0E+07	
54	Isopropylmalic acid	$C_7H_{12}O_5$	[M-H]-	175.06120	175.06126	0.34							1.5E+06	
55	Hexose-1,5-lactone	$C_6H_{10}O_6$	[M-H]-	177.04046	177.04053	0.40							1.6E+06	
56	Fluorenone	$C_{13}H_8O$	[M-H]-	179.05024	179.05020	-0.22	5.8E+06						7.1E+06	
57	Aldohexose	$C_6H_{12}O_6$	[M-H]-	179.05611	179.05619	0.45	2.8E+08	3.8E+06	7.5E+07	3.9E+06	1.1E+08	3.3E+06	4.4E+08	8.8E+06
58	Amino-piperidinecarboxylic acid	$C_6H_{12}N_2O_2$	[M+Cl]-	179.05928	179.05942	0.78	2.9E+06							
59	L-Tyrosine	$C_9H_{11}NO_3$	[M-H]-	180.06662	180.06669	0.39		3.1E+06		2.3E+06		3.8E+06		3.9E+06
60	Mannitol	$C_6H_{14}O_6$	[M-H]-	181.07176	181.07183	0.39		1.4E+06			1.1E+06	1.6E+06	1.1E+06	1.4E+06
61	Tartaric acid	$C_4H_6O_6$	[M+Cl]-	184.98584	184.98574	-0.54							1.0E+06	

					ESI(-) FTICR-MS										
**No.**	**Plausible compound[a]**	**Molecular formula (M)**	**Ion**	**Theor. m/z[b]**	**Exp. m/z[c]**	**Δppm[d]**	**SM$_{R}$H[e]**	**SM$_{R}$O[e]**	**SM$_{P}$H[e]**	**SM$_{P}$O[e]**	**TF$_{R}$H[e]**	**TF$_{R}$O[e]**	**TF$_{P}$H[e]**	**TF$_{P}$O[e]**	
62	Oxodecanoic acid	$C_{10}H_{18}O_3$	[M-H]-	185.11832	185.11837	0.27		1.3E+06						1.2E+06	
63	2-(Acetamidomethylene)	$C_7H_9NO_5$	[M-H]-	186.04080	186.04087	0.38							9.2E+05		
64	Oxo-amino-nonanoic acid	$C_9H_{17}NO_3$	[M-H]-	186.11357	186.11364	0.38		2.8E+06		1.8E+06	1.6E+06	1.9E+06	1.3E+06	2.1E+06	
65	Nonanedioic acid	$C_9H_{16}O_4$	[M-H]-	187.09758	187.09766	0.43		4.9E+06		2.9E+06	2.3E+06	3.5E+06	7.0E+06	4.6E+06	
66	Hydroxy-decanoic acid	$C_{10}H_{20}O_3$	[M-H]-	187.13397	187.13402	0.27								1.2E+06	
67	N-Acetyl-L-glutamic acid	$C_7H_{11}NO_5$	[M-H]-	188.05645	188.05652	0.37							1.5E+06		
68	Oxalosuccinic acid	$C_6H_6O_7$	[M-H]-	189.00408	189.00415	0.37							1.3E+06		
69	Dehydroquinic acid	$C_7H_{10}O_6$	[M-H]-	189.04046	189.04052	0.32							1.3E+06		
70	Hydroxysuberic acid	$C_8H_{14}O_5$	[M-H]-	189.07685	189.07695	0.53							1.2E+06	9.9E+05	
71	Citric acid	$C_6H_8O_7$	[M-H]-	191.01973	191.01980	0.37	1.5E+08		1.2E+08		4.8E+07		4.5E+08	1.6E+06	
72	Quinic acid	$C_7H_{12}O_6$	[M-H]-	191.05611	191.05618	0.37					2.7E+06		2.0E+07		
73	D-Glucuronic acid	$C_6H_{10}O_7$	[M-H]-	193.03538	193.03545	0.36	9.7E+06				2.9E+06		3.5E+06		
74	Galactonic acid	$C_6H_{12}O_7$	[M-H]-	195.05103	195.05108	0.26	2.6E+07	2.1E+06	1.1E+06	1.3E+06	2.1E+06	8.4E+07	8.8E+06		
75	Dodecenoic acid	$C_{12}H_{22}O_2$	[M-H]-	197.15470	197.15475	0.25							1.2E+06		
76	L-Threonine O-3-phosphate	$C_4H_{10}NO_6P$	[M-H]-	198.01730	198.01736	0.30	1.8E+06								
77	Dihydroclavaminic acid	$C_8H_{12}N_2O_4$	[M-H]-	199.07243	199.07253	0.50								9.3E+05	
78	Decenedioic acid	$C_{10}H_{16}O_4$	[M-H]-	199.09758	199.09762	0.20		1.1E+06							
79	Hydroxy-undecenoic acid	$C_{11}H_{20}O_3$	[M-H]-	199.13397	199.13405	0.40		1.1E+06						1.1E+06	
80	Lauric acid	$C_{12}H_{24}O_2$	[M-H]-	199.17035	199.17043	0.40		4.0E+06		2.2E+06	1.4E+06	4.2E+06	1.3E+07	3.9E+06	
81	Diethyl oxalpropionic acid	$C_9H_{14}O_5$	[M-H]-	201.07685	201.07692	0.35		1.9E+06		1.2E+06		1.3E+06	1.6E+06	1.8E+06	
82	Sebacic acid	$C_{10}H_{18}O_4$	[M-H]-	201.11323	201.11330	0.35		4.4E+06		2.6E+06	2.9E+06	2.5E+06	3.3E+06	6.1E+06	

					ESI(-) FTICR-MS									
**No.**	**Plausible compound[a]**	**Molecular formula (M)**	**Ion**	**Theor. m/z[b]**	**Exp. m/z[c]**	**Δppm[d]**	**SM_RH[e]**	**SM_RO[e]**	**SM_PH[e]**	**SM_PO[e]**	**TF_RH[e]**	**TF_RO[e]**	**TF_PH[e]**	**TF_PO[e]**
83	L-Tryptophan	$C_{11}H_{12}N_2O_2$	[M-H]-	203.08260	203.08266	0.30		2.0E+06		1.3E+06		3.2E+06		2.2E+06
84	Diethyl-methyl-hydroxysuccinate	$C_9H_{16}O_5$	[M-H]-	203.09250	203.09253	0.15							9.9E+05	
85	Acetyl-hydroxy-L-lysine	$C_8H_{16}N_2O_4$	[M-H]-	203.10373	203.10379	0.30		1.2E+06						
86	Methylcitric acid	$C_7H_{10}O_7$	[M-H]-	205.03538	205.03543	0.24	1.0E+07		1.5E+06		1.6E+06		1.9E+07	
87	Naphthalenesulfonic acid	$C_{10}H_8O_3S$	[M-H]-	207.01214	207.01221	0.34		1.6E+06				1.4E+06	4.4E+06	2.8E+06
88	beta-D-Sedoheptulopyranose	$C_7H_{14}O_7$	[M-H]-	209.06668	209.06672	0.19	7.7E+06				1.3E+06		2.9E+06	
89	Oxo-dodecenoic acid	$C_{12}H_{20}O_3$	[M-H]-	211.13397	211.13400	0.14		1.1E+06						
90	Methyl-dodecenoic acid	$C_{13}H_{24}O_2$	[M-H]-	211.17035	211.17041	0.28		1.6E+06					1.5E+06	1.4E+06
91	Oxododecanoic acid	$C_{12}H_{22}O_3$	[M-H]-	213.14962	213.14971	0.42		1.2E+06						1.0E+06
92	Methyl-dodecanoic acid	$C_{13}H_{26}O_2$	[M-H]-	213.18600	213.18607	0.33	1.7E+06	9.6E+06		4.9E+06	3.2E+06	1.1E+07	2.5E+07	1.0E+07
93	N-Nonanoylglycine	$C_{11}H_{21}NO_3$	[M-H]-	214.14487	214.14495	0.37		1.5E+06						1.0E+06
94	Bergapten	$C_{12}H_8O_4$	[M-H]-	215.03498	215.03507	0.42							3.3E+06	
95	Hydroxy-dodecanoic acid	$C_{12}H_{24}O_3$	[M-H]-	215.16527	215.16541	0.65								1.1E+06
96	Hydroxydecanedioic acid	$C_{10}H_{18}O_5$	[M-H]-	217.10815	217.10823	0.37		1.2E+06					1.0E+06	1.2E+06
97	O-Succinylhomoserine	$C_8H_{13}NO_6$	[M-H]-	218.06701	218.06707	0.28							1.0E+06	
98	Dihydroxykynurenate	$C_{10}H_7NO_5$	[M-H]-	220.02515	220.02516	0.05			1.7E+06			1.2E+06		
99	Acetyl-glucose	$C_8H_{14}O_7$	[M-H]-	221.06668	221.06673	0.23	1.9E+06		1.2E+06				1.4E+06	
100	Tetradecadienoic acid	$C_{14}H_{24}O_2$	[M-H]-	223.17035	223.17041	0.27							1.0E+06	
101	Myristoleic acid	$C_{14}H_{26}O_2$	[M-H]-	225.18600	225.18607	0.31	4.1E+06	3.2E+07		1.6E+07	1.1E+07	4.1E+07	4.0E+07	3.8E+07
102	Dihydroxy-diepoxy-undecadienol	$C_{11}H_{16}O_5$	[M-H]-	227.09250	227.09256	0.26		1.2E+06					1.5E+06	1.5E+06
103	Myristic acid	$C_{14}H_{28}O_2$	[M-H]-	227.20165	227.20172	0.31	1.2E+07	9.9E+07	2.0E+06	4.4E+07	3.0E+07	9.3E+07	2.0E+08	9.7E+07

					ESI(-) FTICR-MS								
**No.**	**Plausible compound**[a]	**Molecular formula (M)**	**Ion**	**Theor. m/z**[b]	**Exp. m/z**[c]	**Δppm**[d]	**SM$_R$H**[e]	**SM$_R$O**[e]	**SM$_P$H**[e]	**SM$_P$O**[e]	**TF$_R$H**[e]	**TF$_R$O**[e]	**TF$_P$H**[e]	**TF$_P$O**[e]
104	D-Arabinose 5-phosphate	$C_5H_{11}O_8P$	[M-H]-	229.01188	229.01188	0.00							1.3E+06	
105	Dodecanedioic acid	$C_{12}H_{22}O_4$	[M-H]-	229.14453	229.14461	0.35		1.6E+06					1.4E+06	1.2E+06
106	N-Acetyl-D-glucosaminate	$C_8H_{15}NO_7$	[M-H]-	236.07758	236.07760	0.08							1.1E+06	
107	Deoxy-manno-octulosonate	$C_8H_{14}O_8$	[M-H]-	237.06159	237.06164	0.21							3.0E+06	
108	Campherene-diol	$C_{15}H_{26}O_2$	[M-H]-	237.18600	237.18603	0.13		1.3E+06					1.3E+06	
109	Carboxy-methyl-propyl-furanpropanoic acid	$C_{12}H_{16}O_5$	[M-H]-	239.09250	239.09256	0.25							1.2E+06	
110	Tridecenyl acetate	$C_{15}H_{28}O_2$	[M-H]-	239.20165	239.20172	0.29	8.6E+06	6.7E+07	1.3E+06	3.2E+07	2.2E+07	8.3E+07	7.3E+07	7.7E+07
111	Myo-Inositol phosphate	$C_6H_{11}O_8P$	[M-H]-	241.01188	241.01206	0.75							4.4E+06	
112	Keto myristic acid	$C_{14}H_{26}O_3$	[M-H]-	241.18092	241.18100	0.33		1.9E+06						2.2E+06
113	Methyl myristic acid	$C_{15}H_{30}O_2$	[M-H]-	241.21730	241.21737	0.29	2.4E+07	2.1E+08	4.1E+06	8.3E+07	5.8E+07	1.8E+08	3.1E+08	1.9E+08
114	Uridine	$C_9H_{12}N_2O_6$	[M-H]-	243.06226	243.06229	0.12			1.1E+06				2.1E+06	
115	Methyl-dodecanedioic acid	$C_{13}H_{24}O_4$	[M-H]-	243.16018	243.16025	0.29		1.8E+06				1.5E+06	1.7E+06	1.5E+06
116	2-Hydroxymyristic acid	$C_{14}H_{28}O_3$	[M-H]-	243.19657	243.19664	0.29		1.8E+06			1.2E+06		1.3E+06	2.0E+06
117	Glycerophosphoglycerol	$C_6H_{15}O_8P$	[M-H]-	245.04318	245.04331	0.53	3.0E+06		1.4E+06				2.7E+06	
118	Hydroxyindoleacetylglycine	$C_{12}H_{12}N_2O_4$	[M-H]-	247.07243	247.07231	-0.49		1.2E+06						1.3E+06
119	Hexadecatrienoic acid	$C_{16}H_{26}O_2$	[M-H]-	249.18600	249.18604	0.16		1.2E+06						
120	Muramic acid	$C_9H_{17}NO_7$	[M-H]-	250.09323	250.09329	0.24			6.3E+06				1.3E+06	
121	Hexadecynoic acid	$C_{16}H_{28}O_2$	[M-H]-	251.20165	251.20171	0.24		1.4E+07		6.4E+06	4.1E+06	1.6E+07	1.1E+07	1.6E+07
122	N-(Aminobutyryl)-L-histidine	$C_{11}H_{18}N_4O_3$	[M-H]-	253.13061	253.13063	0.08		1.6E+06					1.1E+06	
123	Palmitoleic acid	$C_{16}H_{30}O_2$	[M-H]-	253.21730	253.21736	0.24	8.6E+07	7.1E+08	1.1E+07	3.2E+08	2.1E+08	7.7E+08	5.6E+08	7.6E+08
124	Oxo-pentadecanoic acid	$C_{15}H_{28}O_3$	[M-H]-	255.19657	255.19664	0.27		4.5E+06		2.2E+06		3.7E+06	3.5E+06	4.4E+06

ESI(-) FTICR-MS														
**No.**	**Plausible compound**[a]	**Molecular formula (M)**	**Ion**	**Theor. m/z**[b]	**Exp. m/z**[c]	**Δppm**[d]	**SM$_R$H**[e]	**SM$_R$O**[e]	**SM$_P$H**[e]	**SM$_P$O**[e]	**TF$_R$H**[e]	**TF$_R$O**[e]	**TF$_P$H**[e]	**TF$_P$O**[e]
125	Palmitic acid	$C_{16}H_{32}O_2$	[M-H]-	255.23295	255.23301	0.24	1.0E+08	7.9E+08	2.2E+07	3.3E+08	2.0E+08	6.7E+08	9.9E+08	7.3E+08
126	N-Acetyl-D-hexosamine	$C_8H_{15}NO_6$	[M+Cl]-	256.05934	256.05935	0.04							1.4E+06	
127	Tetradecanedioic acid	$C_{14}H_{26}O_4$	[M-H]-	257.17583	257.17587	0.16		2.4E+06				1.4E+06	1.6E+06	1.9E+06
128	Ketose 1-phosphate	$C_6H_{13}O_9P$	[M-H]-	259.02244	259.02249	0.19	2.5E+07		9.9E+06		8.2E+06		3.2E+07	
129	Hymexazol N-glucoside	$C_{10}H_{15}NO_7$	[M-H]-	260.07758	260.07760	0.08							1.2E+06	
130	Heptadecenynoic acid	$C_{17}H_{28}O_2$	[M-H]-	263.20165	263.20171	0.23		1.5E+06				1.8E+06	1.5E+06	1.5E+06
131	Dinor-dihydro-oxo-phytoenoic acid	$C_{16}H_{26}O_3$	[M-H]-	265.18092	265.18097	0.19							2.9E+06	
132	Pentadecadienyl acetate	$C_{17}H_{30}O_2$	[M-H]-	265.21730	265.21737	0.26	1.3E+06	5.0E+06		1.8E+06	2.1E+06	6.1E+06	4.9E+06	5.3E+06
133	Neuraminic acid	$C_9H_{17}NO_8$	[M-H]-	266.08814	266.08819	0.19	4.7E+06						1.3E+06	
134	MannosyL-glycerate	$C_9H_{16}O_9$	[M-H]-	267.07216	267.07220	0.15	2.2E+07				2.3E+06		1.6E+07	
135	Oxo-pentyl-cyclopentanehexanoic acid	$C_{16}H_{28}O_3$	[M-H]-	267.19657	267.19662	0.19	1.4E+06	5.6E+06		2.7E+06	1.9E+06	5.8E+06	4.0E+06	6.7E+06
136	Methyl-hexadecenoic acid	$C_{17}H_{32}O_2$	[M-H]-	267.23295	267.23301	0.22	2.8E+07	1.7E+08	3.6E+06	7.4E+07	6.6E+07	2.0E+08	1.8E+08	1.8E+08
137	Valerenic acid	$C_{15}H_{22}O_2$	[M+Cl]-	269.13138	269.13123	-0.56		2.2E+06						
138	Keto palmitic acid	$C_{16}H_{30}O_3$	[M-H]-	269.21222	269.21228	0.22	2.4E+06	1.5E+07		6.1E+06	3.9E+06	1.3E+07	1.2E+07	1.6E+07
139	Methyl palmitic acid	$C_{17}H_{34}O_2$	[M-H]-	269.24860	269.24866	0.22	2.6E+07	1.6E+08	4.3E+06	6.7E+07	5.7E+07	1.6E+08	2.2E+08	1.6E+08
140	Trihydroxyisoflavanone	$C_{15}H_{12}O_5$	[M-H]-	271.06120	271.06124	0.15	3.5E+06		6.1E+06				5.8E+07	
141	Methyl-tetradecanedioic acid	$C_{15}H_{28}O_4$	[M-H]-	271.19148	271.19156	0.29		1.2E+06					1.5E+06	1.3E+06
142	Hydroxy palmitic acid	$C_{16}H_{32}O_3$	[M-H]-	271.22787	271.22793	0.22	2.9E+06	1.3E+07	1.3E+06	5.3E+06	4.2E+06	1.5E+07	1.2E+07	1.4E+07
143	Deoxy-heptulose-phosphate	$C_7H_{15}O_9P$	[M-H]-	273.03809	273.03813	0.15	2.3E+06							
144	Hydroxytetradecanedioic acid	$C_{14}H_{26}O_5$	[M-H]-	273.17075	273.17077	0.07							1.3E+06	
145	Dodecanoyl-sn-glycerol	$C_{15}H_{30}O_4$	[M-H]-	273.20713	273.20715	0.07							1.4E+06	

ESI(-) FTICR-MS

No.	Plausible compound[a]	Molecular formula (M)	Ion	Theor. m/z[b]	Exp. m/z[c]	Δppm[d]	SM$_R$H[e]	SM$_R$O[e]	SM$_P$H[e]	SM$_P$O[e]	TF$_R$H[e]	TF$_R$O[e]	TF$_P$H[e]	TF$_P$O[e]
146	(L-Glutamyl)-glutamine	$C_{10}H_{17}N_3O_6$	[M-H]-	274.10446	274.10451	0.18	1.5E+06							
147	Gamma Glutamylglutamic acid	$C_{10}H_{16}N_2O_7$	[M-H]-	275.08847	275.08855	0.29	1.3E+06							
148	Linolenic acid	$C_{18}H_{30}O_2$	[M-H]-	277.21730	277.21736	0.22	2.3E+06	1.1E+07		4.9E+06	5.2E+06	1.3E+07	1.2E+07	1.1E+07
149	Linoleic acid	$C_{18}H_{32}O_2$	[M-H]-	279.23295	279.23301	0.21	3.2E+07	1.7E+08	4.1E+06	7.9E+07	7.6E+07	2.0E+08	1.7E+08	1.9E+08
150	Xylobiose	$C_{10}H_{18}O_9$	[M-H]-	281.08781	281.08784	0.11							1.5E+06	
151	Dihydrophaseic acid	$C_{15}H_{22}O_5$	[M-H]-	281.13945	281.13951	0.21							1.1E+06	
152	Dehydrolupanine	$C_{15}H_{22}N_2O$	[M+Cl]-	281.14261	281.14283	0.78							1.4E+06	
153	Hydroxy-heptadecadienoic acid	$C_{17}H_{30}O_3$	[M-H]-	281.21222	281.21228	0.21		2.2E+06					2.7E+06	2.4E+06
154	Oleic acid	$C_{18}H_{34}O_2$	[M-H]-	281.24860	281.24866	0.21	8.1E+07	5.0E+08	1.0E+07	2.2E+08	1.8E+08	5.5E+08	5.4E+08	5.1E+08
155	Guanosine	$C_{10}H_{13}N_5O_5$	[M-H]-	282.08439	282.08443	0.14	2.1E+06						1.3E+06	
156	Dihydrosantonin	$C_{15}H_{20}O_3$	[M+Cl]-	283.11065	283.11055	-0.35							2.5E+06	1.7E+06
157	Dihexyl maleic acid	$C_{16}H_{28}O_4$	[M-H]-	283.19148	283.19155	0.25		1.8E+06					1.6E+06	1.4E+06
158	Methoxy-hexadecenoic acid	$C_{17}H_{32}O_3$	[M-H]-	283.22787	283.22793	0.21		4.6E+06		2.0E+06	2.4E+06	4.1E+06	3.9E+06	5.2E+06
159	Stearic acid	$C_{18}H_{36}O_2$	[M-H]-	283.26425	283.26431	0.21	6.1E+07	3.3E+08	2.8E+07	1.6E+08	1.0E+08	2.5E+08	4.3E+08	3.5E+08
160	Abscisic alcohol	$C_{15}H_{22}O_3$	[M+Cl]-	285.12630	285.12618	-0.42		2.5E+06				1.5E+06		1.7E+06
161	Hydroxy-oxo-hexadecanoic acid	$C_{16}H_{30}O_4$	[M-H]-	285.20713	285.20720	0.25		3.4E+06		1.4E+06	1.6E+06	2.3E+06	3.2E+06	3.2E+06
162	Hydroxy-palmitic acid methyl ester	$C_{17}H_{34}O_3$	[M-H]-	285.24352	285.24358	0.21	1.6E+06	4.8E+06		2.1E+06	1.7E+06	5.0E+06	4.3E+06	5.1E+06
163	Dihydrokaempferol	$C_{15}H_{12}O_6$	[M-H]-	287.05611	287.05619	0.28							1.2E+06	
164	Dihydroxy-palmitic acid	$C_{16}H_{32}O_4$	[M-H]-	287.22278	287.22285	0.24		7.7E+06					3.2E+06	1.8E+06
165	Sedoheptulose-phosphate	$C_7H_{15}O_{10}P$	[M-H]-	289.03301	289.03305	0.14	2.8E+06							
166	Malonyl-D-tryptophan	$C_{14}H_{14}N_2O_5$	[M-H]-	289.08300	289.08305	0.17	1.7E+06							

ESI(-) FTICR-MS														
**No.**	**Plausible compound**[a]	**Molecular formula (M)**	**Ion**	**Theor. m/z**[b]	**Exp. m/z**[c]	**Δppm**[d]	**SM$_R$H**[e]	**SM$_R$O**[e]	**SM$_P$H**[e]	**SM$_P$O**[e]	**TF$_R$H**[e]	**TF$_R$O**[e]	**TF$_P$H**[e]	**TF$_P$O**[e]
167	Anhydro-N-acetylneuraminic acid	$C_{11}H_{17}NO_8$	[M-H]-	290.08814	290.08819	0.17	6.1E+07	1.4E+06	1.9E+07		9.2E+06		1.6E+07	
168	Hydroxysteroid	$C_{19}H_{32}O_2$	[M-H]-	291.23295	291.23301	0.21		3.6E+06			9.0E+05		1.3E+06	1.7E+06
169	N-Glycosyl-L-asparagine	$C_{10}H_{18}N_2O_8$	[M-H]-	293.09904	293.09908	0.14	1.9E+07		5.6E+06		2.8E+06		3.5E+06	
170	Hydroxylinolenic acid	$C_{18}H_{30}O_3$	[M-H]-	293.21222	293.21228	0.20		6.7E+06			1.5E+06	2.6E+06	2.1E+06	2.9E+06
171	Methyl-octadecadienoic acid	$C_{19}H_{34}O_2$	[M-H]-	293.24860	293.24866	0.20	2.6E+06	2.4E+07		5.3E+06	4.4E+06	1.3E+07	9.1E+06	1.2E+07
172	Tocopheronic acid	$C_{16}H_{24}O_5$	[M-H]-	295.15510	295.15515	0.17		1.8E+06						
173	Ropinirole	$C_{16}H_{24}N_2O$	[M+Cl]-	295.15826	295.15851	0.85		1.2E+06						
174	Methanoxyethano-isolongifol-ene	$C_{18}H_{28}O$	[M-H]-	295.18342	295.18350	0.27		6.2E+06					1.9E+06	
175	Deoxymethynolide	$C_{17}H_{28}O_4$	[M-H]-	295.19148	295.19152	0.14		1.4E+06						
176	Hydroxy-linoleic acid	$C_{18}H_{32}O_3$	[M-H]-	295.22787	295.22793	0.20	2.4E+06	2.3E+07		4.3E+06	2.9E+06	8.9E+06	7.2E+06	1.1E+07
177	Methyl-octadecenoic acid	$C_{19}H_{36}O_2$	[M-H]-	295.26425	295.26431	0.20	4.1E+06	6.3E+07		1.0E+07	8.6E+06	2.7E+07	2.5E+07	2.4E+07
178	N-oxododecanoyl-homoserine lactone	$C_{16}H_{27}NO_4$	[M-H]-	296.18673	296.18685	0.41		1.6E+06						
179	Tetranor-oxo-dihydroxy-prostenoic acid	$C_{16}H_{26}O_5$	[M-H]-	297.17075	297.17077	0.07		7.2E+06					1.6E+06	
180	Heptadecenedioic acid	$C_{17}H_{30}O_4$	[M-H]-	297.20713	297.20718	0.17		1.7E+06						
181	Keto stearic acid	$C_{18}H_{34}O_3$	[M-H]-	297.24352	297.24357	0.17	2.8E+06	4.1E+07		8.4E+06	4.8E+06	1.4E+07	1.2E+07	1.8E+07
182	Methyl stearic acid	$C_{19}H_{38}O_2$	[M-H]-	297.27990	297.27996	0.20	3.8E+06	5.6E+07		7.9E+06	6.5E+06	1.8E+07	2.2E+07	1.9E+07
183	Pentadecanoylglycine	$C_{17}H_{33}NO_3$	[M-H]-	298.23877	298.23882	0.17		4.3E+06		1.1E+06				1.6E+06
184	[(Dihydro-hydroxy-naphthalenyl)thio]- oxopropanoic acid	$C_{13}H_{12}O_4S$	[M+Cl]-	299.01503	299.01503	0.00	1.7E+06		1.4E+06					
185	Salidroside	$C_{14}H_{20}O_7$	[M-H]-	299.11363	299.11368	0.17		1.3E+06						
186	Tetranor-trihydroxy-prostenoic acid	$C_{16}H_{28}O_5$	[M-H]-	299.18640	299.18647	0.23		7.3E+06		1.7E+06		1.9E+06	2.1E+06	4.3E+06

				ESI(-) FTICR-MS										
**No.**	**Plausible compound**[a]	**Molecular formula (M)**	**Ion**	**Theor. m/z**[b]	**Exp. m/z**[c]	**Δppm**[d]	**SM_RH**[e]	**SM_RO**[e]	**SM_PH**[e]	**SM_PO**[e]	**TF_RH**[e]	**TF_RO**[e]	**TF_PH**[e]	**TF_PO**[e]
187	Retinoic acid	$C_{20}H_{28}O_2$	[M-H]-	299.20165	299.20172	0.23		1.9E+06						
188	Tetradecenoyl-glycerol	$C_{17}H_{32}O_4$	[M-H]-	299.22278	299.22283	0.17		1.6E+07		1.4E+06	1.4E+06		4.5E+06	2.5E+06
189	Hydroxystearic acid	$C_{18}H_{36}O_3$	[M-H]-	299.25917	299.25923	0.20	2.4E+06	2.1E+07		3.0E+06	3.1E+06	6.8E+06	6.5E+06	6.7E+06
190	N-Acetyl-D-Glucosamine 6-Phosphate	$C_8H_{16}NO_9P$	[M-H]-	300.04899	300.04903	0.13	2.4E+06		1.5E+06				1.7E+06	
191	Xanthoxic acid	$C_{15}H_{22}O_4$	[M+Cl]-	301.12121	301.12108	-0.43	1.2E+06	6.2E+06			1.2E+06			
192	Tributyrin	$C_{15}H_{26}O_6$	[M-H]-	301.16566	301.16573	0.23		4.7E+06					1.8E+06	1.2E+06
193	Hydroxy-hexadecan-dioic acid	$C_{16}H_{30}O_5$	[M-H]-	301.20205	301.20208	0.10		4.0E+06					1.7E+06	
194	Eicosapentaenoic acid	$C_{20}H_{30}O_2$	[M-H]-	301.21730	301.21734	0.13		1.8E+06						
195	MG(0:0/14:0/0:0)	$C_{17}H_{34}O_4$	[M-H]-	301.23843	301.23847	0.13		1.9E+07					4.8E+06	
196	Adenosine	$C_{10}H_{13}N_5O_4$	[M+Cl]-	302.06616	302.06631	0.50	1.8E+06							
197	Nopaline	$C_{11}H_{20}N_4O_6$	[M-H]-	303.13101	303.13104	0.10		2.5E+06						
198	Trihydroxy palmitic acid	$C_{16}H_{32}O_5$	[M-H]-	303.21770	303.21775	0.16		2.4E+06					1.3E+06	
199	Arachidonic acid	$C_{20}H_{32}O_2$	[M-H]-	303.23295	303.23301	0.20		5.6E+06			1.5E+06		2.5E+06	3.0E+06
200	20:3(5Z,13Z,16Z)	$C_{20}H_{34}O_2$	[M-H]-	305.24860	305.24866	0.20	3.7E+06	4.1E+07		9.0E+06	8.5E+06	2.4E+07	1.7E+07	2.1E+07
201	Glutathione	$C_{10}H_{17}N_3O_6S$	[M-H]-	306.07653	306.07655	0.07	2.2E+06						5.9E+06	
202	Amino-hexadecanoic acid	$C_{16}H_{33}NO_2$	[M+Cl]-	306.22053	306.22056	0.10		2.2E+06						
203	Obscurine	$C_{17}H_{24}N_2O$	[M+Cl]-	307.15826	307.15850	0.78		3.6E+06					2.2E+06	
204	Eicosadienoic acid	$C_{20}H_{36}O_2$	[M-H]-	307.26425	307.26431	0.20	7.9E+06	9.5E+07		1.8E+07	1.8E+07	4.7E+07	4.0E+07	4.1E+07
205	N-Acetyl-a-neuraminic acid	$C_{11}H_{19}NO_9$	[M-H]-	308.09870	308.09875	0.16	9.2E+07	6.0E+06	2.5E+07		1.2E+07		2.3E+08	
206	Sauroxine	$C_{17}H_{26}N_2O$	[M+Cl]-	309.17392	309.17418	0.84	2.7E+07	9.7E+08	1.3E+07	4.9E+06	6.8E+06	1.2E+07	2.8E+08	2.2E+07
207	Epoxy-oxo-octadecenoic acid	$C_{18}H_{30}O_4$	[M-H]-	309.20713	309.20722	0.29		2.8E+06						1.7E+06

ESI(-) FTICR-MS														
**No.**	**Plausible compound**[a]	**Molecular formula (M)**	**Ion**	**Theor. m/z**[b]	**Exp. m/z**[c]	**Δppm**[d]	**SM_RH**[e]	**SM_RO**[e]	**SM_PH**[e]	**SM_PO**[e]	**TF_RH**[e]	**TF_RO**[e]	**TF_PH**[e]	**TF_PO**[e]
208	Octadecenyl acetate	$C_{20}H_{38}O_2$	[M-H]-	309.27990	309.27996	0.19	5.9E+06	8.5E+07		1.3E+07	1.3E+07	3.4E+07	3.7E+07	3.0E+07
209	Alanyltryptophan	$C_{14}H_{17}N_3O_3$	[M+Cl]-	310.09639	310.09665	0.84	2.1E+06							
210	O-(beta-D-Xylopyranosyl)-D-glucopyranose	$C_{11}H_{20}O_{10}$	[M-H]-	311.09837	311.09841	0.13							2.9E+06	
211	Octadecadiynoic acid	$C_{18}H_{28}O_2$	[M+Cl]-	311.17833	311.17838	0.16		1.3E+07					4.0E+06	
212	Dihydroxy-octadecadienoic acid	$C_{18}H_{32}O_4$	[M-H]-	311.22278	311.22285	0.22		1.0E+07		1.4E+06			3.4E+06	2.9E+06
213	Oxo-nonadecanoic acid	$C_{19}H_{36}O_3$	[M-H]-	311.25917	311.25920	0.10		1.4E+06						
214	Arachidic acid	$C_{20}H_{40}O_2$	[M-H]-	311.29555	311.29561	0.19	6.2E+06	8.6E+07	1.4E+06	1.3E+07	1.2E+07	2.7E+07	3.8E+07	2.7E+07
215	Lycomarasmine B	$C_9H_{15}N_3O_7$	[M+Cl]-	312.06040	312.06039	-0.03	1.7E+06		1.1E+06					
216	Oxo-retinoate	$C_{20}H_{25}O_3$	[M-H]-	312.17309	312.17324	0.48							2.4E+06	
217	N-palmitoyl glycine	$C_{18}H_{35}NO_3$	[M-H]-	312.25442	312.25447	0.16		3.8E+06						
218	5'-Phosphoribosyl-N-formylglycinamide	$C_8H_{15}N_2O_9P$	[M-H]-	313.04424	313.04438	0.45	1.8E+06							
219	Dihydroxy-octadecenoic acid	$C_{18}H_{34}O_4$	[M-H]-	313.23843	313.23850	0.22	2.0E+06	1.8E+07		2.3E+06	1.6E+06	4.0E+06	7.0E+06	5.0E+06
220	Hydroxy-nonadecanoic acid	$C_{19}H_{38}O_3$	[M-H]-	313.27482	313.27485	0.10		2.1E+06					1.3E+06	
221	Belladine	$C_{19}H_{25}NO_3$	[M-H]-	314.17617	314.17630	0.41		1.7E+06						
222	Dihydroxy-octadecanoic acid	$C_{18}H_{36}O_4$	[M-H]-	315.25408	315.25416	0.25		6.6E+06					2.6E+06	1.3E+06
223	Methyladenosine	$C_{11}H_{15}N_5O_4$	[M+Cl]-	316.08181	316.08198	0.54	1.9E+06							
224	Hexahydroxyflavone	$C_{15}H_{10}O_8$	[M-H]-	317.03029	317.03037	0.25	3.3E+06		1.3E+06					
225	Methylinosine	$C_{11}H_{14}N_4O_5$	[M+Cl]-	317.06582	317.06603	0.66	1.8E+06							
226	Menthyl O-beta-D-glucoside	$C_{16}H_{30}O_6$	[M-H]-	317.19696	317.19703	0.22		1.6E+06						
227	Cucumopine	$C_{11}H_{13}N_3O_6$	[M+Cl]-	318.04984	318.05004	0.63			1.6E+06					
228	Ethyl-androstane-diol	$C_{21}H_{36}O_2$	[M-H]-	319.26425	319.26432	0.22		2.4E+06					1.5E+06	

					ESI(-) FTICR-MS									
**No.**	**Plausible compound**[a]	**Molecular formula (M)**	**Ion**	**Theor. m/z**[b]	**Exp. m/z**[c]	**Δppm**[d]	**SM$_R$H**[e]	**SM$_R$O**[e]	**SM$_P$H**[e]	**SM$_P$O**[e]	**TF$_R$H**[e]	**TF$_R$O**[e]	**TF$_P$H**[e]	**TF$_P$O**[e]
229	Gingerol	$C_{19}H_{30}O_4$	[M-H]-	321.20713	321.20717	0.12		1.6E+06						
230	Methyl-androstanetriol	$C_{20}H_{34}O_3$	[M-H]-	321.24352	321.24356	0.12		2.1E+06						
231	Methyl-eicosadienoic acid	$C_{21}H_{38}O_2$	[M-H]-	321.27990	321.27998	0.25		5.0E+06		1.5E+06			2.3E+06	2.2E+06
232	Cytidine 3'-phosphate	$C_9H_{14}N_3O_8P$	[M-H]-	322.04457	322.04462	0.16	7.2E+06		2.0E+06				2.0E+06	
233	Orotidine	$C_{10}H_{12}N_2O_8$	[M+Cl]-	323.02877	323.02862	-0.46	5.1E+07		1.4E+07		2.9E+06		1.3E+07	
234	Fructofuranose dianhydride	$C_{12}H_{20}O_{10}$	[M-H]-	323.09837	323.09841	0.12							3.4E+06	
235	Flabellidine	$C_{18}H_{28}N_2O$	[M+Cl]-	323.18957	323.18980	0.71		3.3E+06					2.3E+06	
236	Hydroxy-eicosadienoic acid	$C_{20}H_{36}O_3$	[M-H]-	323.25917	323.25921	0.12		3.0E+06					1.3E+06	
237	Heneicosenoic acid	$C_{21}H_{40}O_2$	[M-H]-	323.29555	323.29562	0.22		7.6E+06		1.9E+06	2.0E+06	4.4E+06	4.0E+06	3.1E+06
238	N-Glycoloyl-neuraminate	$C_{11}H_{19}NO_{10}$	[M-H]-	324.09362	324.09364	0.06	1.2E+06						1.7E+06	
239	O-beta-D-Glucosyl-coumarate	$C_{15}H_{18}O_8$	[M-H]-	325.09289	325.09292	0.09	4.1E+06		1.5E+06				3.5E+06	
240	2-O-Fucopyranosyl-galactose	$C_{12}H_{22}O_{10}$	[M-H]-	325.11402	325.11407	0.15							2.3E+07	
241	Oxo-eicosanoic acid	$C_{20}H_{38}O_3$	[M-H]-	325.27482	325.27488	0.18		5.2E+06					2.2E+06	2.0E+06
242	Heneicosanoic acid	$C_{21}H_{42}O_2$	[M-H]-	325.31120	325.31127	0.22	2.5E+06	2.5E+07		4.0E+06	4.4E+06	8.6E+06	1.3E+07	8.4E+06
243	N-palmitoyl alanine	$C_{19}H_{37}NO_3$	[M-H]-	326.27007	326.27011	0.12		2.2E+06						
244	Steryl sulfate	$C_{17}H_{28}O_4S$	[M-H]-	327.16355	327.16359	0.12		2.6E+06					2.4E+06	
245	Difluoro-hexadecanoic acid	$C_{16}H_{30}F_2O_2$	[M+Cl]-	327.19079	327.19106	0.83	1.8E+06	2.3E+07	2.3E+06				7.3E+06	2.1E+06
246	Trihydroxy-octadecadienoic acid	$C_{18}H_{32}O_5$	[M-H]-	327.21770	327.21777	0.21		4.7E+06		1.4E+06		1.8E+06	1.9E+06	3.1E+06
247	MG(0:0/16:1(9Z)/0:0)	$C_{19}H_{36}O_4$	[M-H]-	327.25408	327.25414	0.18	2.0E+06	2.0E+07		2.2E+06	2.0E+06	4.5E+06	7.1E+06	4.6E+06
248	Hydroxy-eicosanoic acid	$C_{20}H_{40}O_3$	[M-H]-	327.29047	327.29051	0.12		3.0E+06					1.7E+06	
249	MG(0:0/16:0/0:0)	$C_{19}H_{38}O_4$	[M-H]-	329.26973	329.26979	0.18		6.2E+06					2.3E+06	

				ESI(-) FTICR-MS										
No.	Plausible compound[a]	Molecular formula (M)	Ion	Theor. m/z[b]	Exp. m/z[c]	Δppm[d]	SM_RH^e	SM_RO^e	SM_PH^e	SM_PO^e	TF_RH^e	TF_RO^e	TF_PH^e	TF_PO^e
250	Pentahydroxy-methoxyflavone	$C_{16}H_{12}O_8$	[M-H]-	331.04594	331.04601	0.21	3.6E+07		4.0E+06		1.5E+06			
251	Hydroxytridecane-tricarboxylic acid	$C_{16}H_{28}O_7$	[M-H]-	331.17623	331.17630	0.21		1.4E+06						
252	Hydroxy-dihydroxy-stearic acid	$C_{18}H_{36}O_5$	[M-H]-	331.24900	331.24905	0.15		1.0E+07					5.2E+06	
253	Adrenic acid	$C_{22}H_{36}O_2$	[M-H]-	331.26425	331.26432	0.21	1.9E+06	1.3E+07		2.7E+06	2.1E+06	5.5E+06	5.5E+06	4.9E+06
254	Albendazole sulfone	$C_{12}H_{15}N_3O_4S$	[M+Cl]-	332.04773	332.04743	-0.90	2.6E+06						1.4E+06	
255	Galactosyl-sn-glycerol-phosphate	$C_9H_{19}O_{11}P$	[M-H]-	333.05922	333.05927	0.15	1.8E+06		1.4E+06				7.9E+06	
256	Docosatrienoic acid	$C_{22}H_{38}O_2$	[M-H]-	333.27990	333.27996	0.18	2.9E+06	2.4E+07		5.3E+06	5.9E+06	1.5E+07	1.2E+07	1.2E+07
257	Acetyl-beta-D-glucosaminyl-asparagine	$C_{12}H_{21}N_3O_8$	[M-H]-	334.12559	334.12563	0.12	2.6E+06		2.2E+06		1.9E+06		3.7E+06	
258	Palmitoylethanolamide	$C_{18}H_{37}NO_2$	[M+Cl]-	334.25183	334.25188	0.15		1.6E+06						
259	Eicosadienyl acetate	$C_{22}H_{40}O_2$	[M-H]-	335.29555	335.29560	0.15	2.1E+06	1.7E+07		3.4E+06	4.0E+06	8.7E+06	9.0E+06	7.6E+06
260	Deoxy-hydroxymethylcytidine-phosphate	$C_{10}H_{16}N_3O_8P$	[M-H]-	336.06022	336.06040	0.54	1.3E+06							
261	Shiromodiol diacetate	$C_{19}H_{30}O_5$	[M-H]-	337.20205	337.20206	0.03		1.2E+06						
262	Docosenoic acid	$C_{22}H_{42}O_2$	[M-H]-	337.31120	337.31126	0.18	2.4E+06	2.0E+07		3.7E+06	4.1E+06	9.8E+06	1.0E+07	7.6E+06
263	N-oleoyl glycine	$C_{20}H_{37}NO_3$	[M-H]-	338.27007	338.27012	0.15		1.3E+06						
264	Ketolactose	$C_{12}H_{20}O_{11}$	[M-H]-	339.09328	339.09335	0.21							1.6E+06	
265	Methyl-fucopyranosyl-galactoside	$C_{13}H_{24}O_{10}$	[M-H]-	339.12967	339.12972	0.15					1.6E+06		5.9E+06	
266	Hydroperoxy-eicosadienoic acid	$C_{20}H_{36}O_4$	[M-H]-	339.25408	339.25412	0.12		4.8E+06					2.0E+06	
267	Oxo-heneicosanoic acid	$C_{21}H_{40}O_3$	[M-H]-	339.29047	339.29055	0.24		3.4E+06						
268	Methyl-heneicosanoic acid	$C_{22}H_{44}O_2$	[M-H]-	339.32685	339.32691	0.18	5.8E+06	6.3E+07	1.2E+06	9.9E+06	1.7E+07	2.4E+07	3.2E+07	2.1E+07
269	Glucosaminyl-myo-inositol	$C_{12}H_{23}NO_{10}$	[M-H]-	340.12492	340.12493	0.03							2.0E+06	
270	Stearoylglycine	$C_{20}H_{39}NO_3$	[M-H]-	340.28572	340.28577	0.15		1.9E+06						

				ESI(-) FTICR-MS										
**No.**	**Plausible compound**[a]	**Molecular formula (M)**	**Ion**	**Theor . m/z**[b]	**Exp. m/z**[c]	**Δppm**[d]	**SM$_R$H**[e]	**SM$_R$O**[e]	**SM$_P$H**[e]	**SM$_P$O**[e]	**TF$_R$H**[e]	**TF$_R$O**[e]	**TF$_P$H**[e]	**TF$_P$O**[e]
271	Caffeic acid 3-glucoside	$C_{15}H_{18}O_9$	[M-H]-	341.08781	341.08784	0.09	1.2E+07		3.7E+06		1.8E+06		4.4E+06	
272	Glucosyl-glucose	$C_{12}H_{22}O_{11}$	[M-H]-	341.10894	341.10899	0.15	3.7E+06	2.3E+07	2.9E+06	3.6E+06	4.4E+06	5.7E+06	1.1E+07	6.6E+06
273	Methyl-dihydroxy-oxo-octadecenoate	$C_{19}H_{34}O_5$	[M-H]-	341.23335	341.23342	0.21		3.1E+06			1.8E+06		1.5E+06	
274	Eicosanedioic acid	$C_{20}H_{38}O_4$	[M-H]-	341.26973	341.26979	0.18	2.1E+06	1.6E+07		2.9E+06	5.4E+06	5.8E+06	7.0E+06	7.5E+06
275	Hydroxy-heneicosanoic acid	$C_{21}H_{42}O_3$	[M-H]-	341.30612	341.30619	0.21		1.5E+06						
276	N-palmitoyl serine	$C_{19}H_{37}NO_4$	[M-H]-	342.26498	342.26502	0.12		2.5E+06						
277	Maltitol	$C_{12}H_{24}O_{11}$	[M-H]-	343.12459	343.12462	0.09							1.7E+06	
278	Affinisine	$C_{20}H_{24}N_2O$	[M+Cl]-	343.15826	343.15850	0.70					1.9E+06		2.2E+06	
279	Dihydroxy-methoxy-octadecenoic acid	$C_{19}H_{36}O_5$	[M-H]-	343.24900	343.24907	0.20		4.4E+07			1.5E+07		2.2E+07	1.3E+06
280	Sulfadoxine	$C_{12}H_{14}N_4O_4S$	[M+Cl]-	345.04298	345.04266	-0.93	4.1E+06		2.2E+06		1.5E+06			
281	Dihydroxy-Octadecanedioic acid	$C_{18}H_{34}O_6$	[M-H]-	345.22826	345.22829	0.09		2.1E+06						
282	Deoxyguanosine 5'-monophosphate	$C_{10}H_{14}N_5O_7P$	[M-H]-	346.05581	346.05585	0.12	1.3E+08	1.7E+06	3.3E+07		1.5E+07		2.6E+07	
283	Leonuridine	$C_{15}H_{24}O_9$	[M-H]-	347.13476	347.13479	0.09		6.7E+06			3.3E+06		2.1E+06	
284	Hydroxy-(methoxymethyl)-methyl-androstan- one	$C_{22}H_{36}O_3$	[M-H]-	347.25917	347.25922	0.14		2.4E+06						1.5E+06
285	Dihydropteroic acid	$C_{14}H_{14}N_6O_3$	[M+Cl]-	349.08214	349.08205	-0.26	3.0E+06							
286	MG(18:3/0:0/0:0)	$C_{21}H_{36}O_4$	[M-H]-	351.25408	351.25408	0.00		1.7E+06						
287	Tricosenoic acid	$C_{23}H_{44}O_2$	[M-H]-	351.32685	351.32692	0.20		5.9E+06			1.8E+06		3.2E+06	2.3E+06
288	N-palmitoyl proline	$C_{21}H_{39}NO_3$	[M-H]-	352.28572	352.28588	0.45							1.4E+06	
289	Chlorogenic acid	$C_{16}H_{18}O_9$	[M-H]-	353.08781	353.08786	0.14	1.1E+07	1.8E+06	1.4E+07		3.9E+06		1.1E+08	
290	MG(0:0/18:2/0:0)	$C_{21}H_{38}O_4$	[M-H]-	353.26973	353.26980	0.20		6.8E+06			1.8E+06		2.3E+06	1.6E+06

					ESI(-) FTICR-MS									
**No.**	**Plausible compound[a]**	**Molecular formula (M)**	**Ion**	**Theor. m/z[b]**	**Exp. m/z[c]**	**Δppm[d]**	**SM$_R$H[e]**	**SM$_R$O[e]**	**SM$_P$H[e]**	**SM$_P$O[e]**	**TF$_R$H[e]**	**TF$_R$O[e]**	**TF$_P$H[e]**	**TF$_P$O[e]**
291	Oxo-docosanoic acid	$C_{22}H_{42}O_3$	[M-H]-	353.30612	353.30619	0.20		3.6E+06				1.9E+06	1.6E+06	1.7E+06
292	Methyl-docosanoic acid	$C_{23}H_{46}O_2$	[M-H]-	353.34250	353.34256	0.17	6.1E+06	6.4E+07	1.4E+06	9.9E+06	1.8E+07	2.6E+07	3.3E+07	2.2E+07
293	N-palmitoyl valine	$C_{21}H_{41}NO_3$	[M-H]-	354.30137	354.30143	0.17		1.7E+06						
294	Feruloyl-glucose	$C_{16}H_{20}O_9$	[M-H]-	355.10346	355.10350	0.11	2.2E+06		1.4E+06				1.8E+06	
295	Prostaglandin F1alpha	$C_{20}H_{36}O_5$	[M-H]-	355.24900	355.24907	0.20		3.6E+06				1.8E+06		2.5E+06
296	MG(0:0/18:1/0:0)	$C_{21}H_{40}O_4$	[M-H]-	355.28538	355.28545	0.20	1.9E+06	1.1E+07		2.2E+06	3.5E+06	3.9E+06	4.4E+06	5.2E+06
297	Hydroxydocosanoic acid	$C_{22}H_{44}O_3$	[M-H]-	355.32177	355.32183	0.17		9.0E+06			3.0E+06	4.4E+06	3.9E+06	2.9E+06
298	N-palmitoyl threonine	$C_{20}H_{39}NO_4$	[M-H]-	356.28063	356.28070	0.20		1.8E+06						
299	Epideoxyloganic acid	$C_{16}H_{24}O_9$	[M-H]-	359.13476	359.13477	0.03	3.1E+06						1.8E+06	
300	Cholanediol	$C_{24}H_{42}O_2$	[M-H]-	361.31120	361.31123	0.08		1.9E+06					1.5E+06	
301	Methylthiopropyl-desulfoglucosinolate	$C_{11}H_{21}NO_6S_2$	[M+Cl]-	362.05043	362.05076	0.91	1.0E+07		2.1E+06		3.5E+06		4.0E+06	
302	Guanosine monophosphate	$C_{10}H_{14}N_5O_8P$	[M-H]-	362.05072	362.05077	0.14	1.0E+07		1.7E+06		3.5E+06		4.0E+06	
303	Docosenyl acetate	$C_{24}H_{46}O_2$	[M-H]-	365.34250	365.34256	0.16	1.1E+07	2.4E+07		4.3E+06	7.3E+06	1.0E+07	1.3E+07	8.6E+06
304	Tetracosanoic acid	$C_{24}H_{48}O_2$	[M-H]-	367.35815	367.35821	0.16	8.9E+07	2.1E+08	3.9E+06	3.5E+07	6.9E+07	8.9E+07	1.3E+08	7.4E+07
305	N-palmitoyl leucine	$C_{22}H_{43}NO_3$	[M-H]-	368.31702	368.31711	0.24		1.7E+06						
306	Heptahydroxyflavone	$C_{15}H_{10}O_9$	[M+Cl]-	369.00188	369.00187	-0.03	1.5E+06							
307	Methoxytaxifolin	$C_{16}H_{14}O_8$	[M+Cl]-	369.03827	369.03814	-0.35			1.6E+06					
308	Fluoro-hydroxyandrostene-trione	$C_{19}H_{23}FO_4$	[M+Cl]-	369.12744	369.12734	-0.27		2.8E+06	1.5E+06					
309	Amylose	$C_{14}H_{26}O_{11}$	[M-H]-	369.14024	369.14029	0.14							1.8E+06	
310	Keto-prostaglandin F1	$C_{20}H_{34}O_6$	[M-H]-	369.22826	369.22828	0.05		1.6E+06						
311	Docosanedioic acid	$C_{22}H_{42}O_4$	[M-H]-	369.30103	369.30109	0.16	1.1E+07	1.9E+07		3.9E+06	5.3E+06	7.5E+06	8.4E+06	1.1E+07

				ESI(-) FTICR-MS											
**No.**	**Plausible compound**[a]	**Molecular formula (M)**	**Ion**	**Theor. m/z**[b]	**Exp. m/z**[c]	**Δppm**[d]	**SM$_R$H**[e]	**SM$_R$O**[e]	**SM$_P$H**[e]	**SM$_P$O**[e]	**TF$_R$H**[e]	**TF$_R$O**[e]	**TF$_P$H**[e]	**TF$_P$O**[e]
312	Hydroxy-tricosanoic acid	$C_{23}H_{46}O_3$	[M-H]-	369.33742	369.33746	0.11	5.1E+06	8.8E+06			3.6E+06		4.4E+06	3.0E+06
313	N-stearoyl serine	$C_{21}H_{41}NO_4$	[M-H]-	370.29628	370.29636	0.22		2.7E+06						
314	Deoxyoleandolide	$C_{20}H_{36}O_6$	[M-H]-	371.24391	371.24397	0.16	7.9E+06	9.3E+06			9.5E+06	2.1E+06	4.8E+06	2.9E+06
315	Urate D-ribonucleotide	$C_{10}H_{13}N_4O_{10}P$	[M-H]-	379.02965	379.02937	-0.74							1.6E+06	
316	Diphyllin	$C_{21}H_{16}O_7$	[M-H]-	379.08233	379.08269	0.95		2.1E+07				2.7E+06	5.8E+06	
317	MG(0:0/20:3/0:0)	$C_{23}H_{40}O_4$	[M-H]-	379.28538	379.28545	0.18		1.6E+06						
318	Pentacosenoic acid	$C_{25}H_{48}O_2$	[M-H]-	379.35815	379.35820	0.13	5.1E+06	8.9E+06		2.4E+06	3.4E+06	4.7E+06	6.0E+06	4.1E+06
319	Amino-formylamino-(phosphoribosylamino)pyrimidinone	$C_{10}H_{16}N_5O_9P$	[M-H]-	380.06129	380.06130	0.03	3.4E+06							
320	PA(14:0/0:0)	$C_{17}H_{35}O_7P$	[M-H]-	381.20476	381.20494	0.47		3.1E+06			1.8E+06		2.1E+06	
321	Heptadecenyl-resorcinol	$C_{23}H_{38}O_2$	[M+Cl]-	381.25658	381.25673	0.39							1.5E+06	
322	MG(0:0/20:2/0:0)	$C_{23}H_{42}O_4$	[M-H]-	381.30103	381.30108	0.13		1.7E+06						
323	Hydroxy-tetracosenoic acid	$C_{24}H_{46}O_3$	[M-H]-	381.33742	381.33749	0.18		3.8E+06			2.3E+06		2.4E+06	2.2E+06
324	Isopentacosanoic acid	$C_{25}H_{50}O_2$	[M-H]-	381.37380	381.37386	0.16	6.4E+07	1.4E+08	3.2E+06	3.5E+07	5.9E+07	6.3E+07	1.0E+08	6.4E+07
325	PC(8:0/0:0)	$C_{16}H_{34}NO_7P$	[M-H]-	382.20001	382.20022	0.55		1.7E+06						1.7E+06
326	Acetyl-maltose	$C_{14}H_{24}O_{12}$	[M-H]-	383.11950	383.11952	0.05							1.7E+06	
327	Sativic acid	$C_{18}H_{36}O_6$	[M+Cl]-	383.22059	383.22064	0.13					1.6E+06		2.2E+06	
328	MG(0:0/20:1/0:0)	$C_{23}H_{44}O_4$	[M-H]-	383.31668	383.31673	0.13	9.2E+06	1.7E+07		4.6E+06	5.8E+06	7.1E+06	8.4E+06	1.3E+07
329	Hydroxy-tetracosanoic acid	$C_{24}H_{48}O_3$	[M-H]-	383.35307	383.35312	0.13	1.2E+07	2.9E+07	1.6E+06	3.8E+06	8.9E+06	1.4E+07	1.7E+07	8.3E+06
330	Carboxy-tocotrienol	$C_{24}H_{33}O_4$	[M-H]-	384.23061	384.23083	0.57		1.9E+06						
331	N-palmitoyl glutamic acid	$C_{21}H_{39}NO_5$	[M-H]-	384.27555	384.27555	0.00		2.8E+06			2.1E+06		1.7E+06	

				ESI(-) FTICR-MS											
**No.**	**Plausible compound[a]**	**Molecular formula (M)**	**Ion**	**Theor. m/z[b]**	**Exp. m/z[c]**	**Δppm[d]**	**SM$_R$H[e]**	**SM$_R$O[e]**	**SM$_P$H[e]**	**SM$_P$O[e]**	**TF$_R$H[e]**	**TF$_R$O[e]**	**TF$_P$H[e]**	**TF$_P$O[e]**
332	6-Deoxyerythronolide B	$C_{21}H_{38}O_6$	[M-H]-	385.25956	385.25962	0.16		6.0E+06			4.7E+06		3.5E+06	1.6E+06
333	Tuberonic acid glucoside	$C_{18}H_{28}O_9$	[M-H]-	387.16606	387.16611	0.13	5.7E+06		3.0E+06				7.2E+06	
334	Rehmaionoside C	$C_{19}H_{32}O_8$	[M-H]-	387.20244	387.20243	-0.03		1.6E+06						
335	Amino-tetrahydroxyeicosenoic acid	$C_{20}H_{39}NO_6$	[M-H]-	388.27046	388.27049	0.08		3.4E+06			3.2E+06		2.0E+06	
336	Hydroxy-dihydrojasmonic acid glucoside	$C_{18}H_{30}O_9$	[M-H]-	389.18171	389.18172	0.03		2.0E+06					1.9E+06	
337	Dehydrorotenone	$C_{23}H_{20}O_6$	[M-H]-	391.11871	391.11885	0.36	2.9E+06							
338	Hexacosadienoic acid	$C_{26}H_{48}O_2$	[M-H]-	391.35815	391.35822	0.18		3.0E+06			1.7E+06		2.0E+06	1.6E+06
339	Miraxanthin-I	$C_{14}H_{18}N_2O_7S$	[M+Cl]-	393.05287	393.05255	-0.81	1.0E+08		3.5E+07		2.1E+07		9.2E+07	
340	MG(0:0/18:0/0:0)	$C_{21}H_{42}O_4$	[M+Cl]-	393.27771	393.27776	0.13					3.9E+06		2.5E+06	
341	Hexacosenoic acid	$C_{26}H_{50}O_2$	[M-H]-	393.37380	393.37386	0.15	8.7E+06	1.7E+07		4.6E+06	6.9E+06	7.7E+06	1.1E+07	7.7E+06
342	PGF2alpha isopropyl ester	$C_{23}H_{40}O_5$	[M-H]-	395.28030	395.28034	0.10		2.6E+06					2.0E+06	
343	Hydroxynervonic acid	$C_{25}H_{48}O_3$	[M-H]-	395.35307	395.35306	-0.03							1.7E+06	
344	Methyl-hexacosanoic acid	$C_{26}H_{52}O_2$	[M-H]-	395.38945	395.38950	0.13	4.5E+07	9.3E+07	2.5E+06	2.5E+07	3.9E+07	4.3E+07	7.0E+07	4.4E+07
345	Trihydroxy-prostadienoyl-ethanolamine	$C_{22}H_{39}NO_5$	[M-H]-	396.27555	396.27562	0.18		1.9E+06						
346	Auramycinone	$C_{21}H_{18}O_8$	[M-H]-	397.09289	397.09325	0.91			8.3E+06				2.4E+06	
347	Tetracosanedioic acid	$C_{24}H_{46}O_4$	[M-H]-	397.33233	397.33238	0.13	2.1E+07	2.0E+07		5.4E+06	7.0E+06	9.7E+06	1.2E+07	1.6E+07
348	Hydroxy-pentacosanoic acid	$C_{25}H_{50}O_3$	[M-H]-	397.36872	397.36877	0.13	9.4E+06	1.5E+07			5.9E+06	6.3E+06	9.3E+06	5.4E+06
349	Aurachin D	$C_{25}H_{33}NO$	[M+Cl]-	398.22562	398.22598	0.90		2.3E+06						
350	Oxo-hydroxydimethylprostadienoic acid	$C_{22}H_{36}O_4$	[M+Cl]-	399.23076	399.23081	0.13							1.9E+06	
351	Didecanoylglycerol	$C_{23}H_{44}O_5$	[M-H]-	399.31160	399.31164	0.10		4.8E+06			2.7E+06		3.6E+06	
352	Hydroxyvitamin D3	$C_{27}H_{44}O_2$	[M-H]-	399.32685	399.32691	0.15		6.8E+06		2.2E+06	2.9E+06	2.9E+06	4.4E+06	2.7E+06

				ESI(-) FTICR-MS									
**No.**	**Plausible compound[a]**	**Molecular formula (M)**	**Ion**	**Theor. m/z[b]**	**Exp. m/z[c]**	**Δppm[d]**	**SM_RH[e]**	**SM_RO[e]**	**SM_PH[e]**	**SM_PO[e]**	**TF_RH[e]**	**TF_RO[e]**	**TF_PH[e]**	**TF_PO[e]**
353	Salicin 6-phosphate	$C_{13}H_{19}O_{10}P$	[M+Cl]-	401.04099	401.04131	0.80	1.1E+07		7.4E+06		3.8E+06		1.3E+07	
354	Dihydroxy-diperoxyeicosadienoic acid	$C_{20}H_{34}O_8$	[M-H]-	401.21809	401.21811	0.05							2.6E+06	
355	Erythronolide B	$C_{21}H_{38}O_7$	[M-H]-	401.25448	401.25453	0.12		2.3E+06					2.2E+06	
356	Hydroxy-mercapto-pregnenedione acetate	$C_{23}H_{32}O_4S$	[M-H]-	403.19485	403.19490	0.12	7.6E+06	1.5E+07			2.9E+06		5.7E+06	
357	Octacosaoctaenoic acid	$C_{28}H_{40}O_2$	[M-H]-	407.29555	407.29559	0.10		8.6E+06		4.1E+06			5.3E+06	
358	Methyl-hexacosenoic acid	$C_{27}H_{52}O_2$	[M-H]-	407.38945	407.38951	0.15		4.2E+06			2.9E+06		3.3E+06	2.1E+06
359	Palmitoylglycerol phosphate	$C_{19}H_{39}O_7P$	[M-H]-	409.23606	409.23619	0.32		2.7E+06						
360	Gama-Tocotrienol	$C_{28}H_{42}O_2$	[M-H]-	409.31120	409.31127	0.17		1.8E+07						
361	MG(22:2/0:0/0:0)	$C_{25}H_{46}O_4$	[M-H]-	409.33233	409.33242	0.22		2.2E+06						1.7E+06
362	Oxohexacosanoic acid	$C_{26}H_{50}O_3$	[M-H]-	409.36872	409.36877	0.12		2.3E+06					2.0E+06	
363	Methyl-hexacosanoic acid	$C_{27}H_{54}O_2$	[M-H]-	409.40510	409.40515	0.12	2.4E+07	2.5E+07		6.4E+06	1.4E+07	1.1E+07	2.6E+07	1.1E+07
364	N-oleoyl glutamic acid	$C_{23}H_{41}NO_5$	[M-H]-	410.29120	410.29124	0.10		2.8E+06					1.9E+06	
365	MG(0:0/22:1/0:0)	$C_{25}H_{48}O_4$	[M-H]-	411.34798	411.34802	0.10	1.6E+07	1.4E+07		3.5E+06	7.8E+06	5.8E+06	9.0E+06	1.0E+07
366	Hydroxyhexacosanoic acid	$C_{26}H_{52}O_3$	[M-H]-	411.38437	411.38442	0.12	1.0E+07	1.2E+07			5.8E+06	6.2E+06	1.1E+07	5.2E+06
367	N-stearoyl glutamic acid	$C_{23}H_{43}NO_5$	[M-H]-	412.30685	412.30681	-0.10		2.3E+06						
368	Methyl hydroperoxy bisepidioxy eicosadienoate	$C_{21}H_{34}O_8$	[M-H]-	413.21809	413.21812	0.07							2.2E+06	
369	MG(0:0/22:0/0:0)	$C_{25}H_{50}O_4$	[M-H]-	413.36363	413.36365	0.05		2.8E+06						
370	(Deoxy-gluc-4-enuronosyl)-N-acetyl-D- glucosamine	$C_{14}H_{21}NO_{11}$	[M+Cl]-	414.08086	414.08071	-0.36	2.9E+06							
371	Calcitriol	$C_{27}H_{44}O_3$	[M-H]-	415.32177	415.32180	0.07		3.0E+06					2.6E+06	
372	Dihydroxy-azavitamin D3	$C_{26}H_{43}NO_3$	[M-H]-	416.31702	416.31701	-0.02		3.5E+06						

				ESI(-) FTICR-MS										
**No.**	**Plausible compound[a]**	**Molecular formula (M)**	**Ion**	**Theor. m/z[b]**	**Exp. m/z[c]**	**Δppm[d]**	**SM$_R$H[e]**	**SM$_R$O[e]**	**SM$_P$H[e]**	**SM$_P$O[e]**	**TF$_R$H[e]**	**TF$_R$O[e]**	**TF$_P$H[e]**	**TF$_P$O[e]**
373	Hydroxy-mercaptoandrostenone acetate propionate	$C_{24}H_{34}O_4S$	[M-H]-	417.21050	417.21054	0.10	2.4E+07	6.8E+07	6.1E+06		8.6E+06	2.8E+06	2.8E+07	3.6E+06
374	Phyllanthin	$C_{24}H_{34}O_6$	[M-H]-	417.22826	417.22830	0.10	5.1E+06	4.0E+06						
375	Palmitoyl glucuronide	$C_{22}H_{42}O_7$	[M-H]-	417.28578	417.28583	0.12		3.2E+06			2.4E+06		3.2E+06	
376	Lamioside	$C_{18}H_{28}O_{11}$	[M-H]-	419.15589	419.15593	0.10							2.6E+06	
377	Epigallocatechin sulfate	$C_{15}H_{13}O_{10}S$	[M+Cl]-	419.99234	419.99270	0.86	3.2E+06							
378	Phospho-glucosyl-glucose	$C_{12}H_{23}O_{14}P$	[M-H]-	421.07527	421.07528	0.02					3.2E+06		3.9E+06	
379	Bis(ethylhexyl) sulfosuccinate	$C_{20}H_{38}O_7S$	[M-H]-	421.22655	421.22657	0.05		2.6E+07	5.9E+06	8.2E+06	9.3E+06	1.1E+08	9.8E+06	3.0E+07
380	Methyl-butanoyloxy-villanovanediol	$C_{25}H_{42}O_5$	[M-H]-	421.29595	421.29600	0.12								2.3E+06
381	Octacosenoic acid	$C_{28}H_{54}O_2$	[M-H]-	421.40510	421.40514	0.09		8.8E+06		5.5E+06	7.4E+06	9.3E+06	7.5E+06	9.9E+06
382	Dopaxanthin quinone	$C_{18}H_{16}N_2O_8$	[M+Cl]-	423.06007	423.06027	0.47							1.9E+06	
383	Montanic acid	$C_{28}H_{56}O_2$	[M-H]-	423.42075	423.42079	0.09	1.8E+07	2.1E+07		1.6E+07	2.3E+07	2.9E+07	2.3E+07	3.1E+07
384	Hexacosanedioic acid	$C_{26}H_{50}O_4$	[M-H]-	425.36363	425.36367	0.09	2.4E+07	2.1E+07	4.1E+06	1.7E+07	1.2E+07	2.6E+07	1.3E+07	4.7E+07
385	Mycolipanolic acid	$C_{27}H_{54}O_3$	[M-H]-	425.40002	425.40006	0.09		4.5E+06		3.1E+06	3.8E+06	7.6E+06	3.8E+06	6.1E+06
386	Stearoylcarnitine	$C_{25}H_{49}NO_4$	[M-H]-	426.35888	426.35893	0.12						7.0E+06		
387	Glyceryl-PGF2alpha	$C_{23}H_{40}O_7$	[M-H]-	427.27013	427.27016	0.07								2.3E+06
388	Calcitetrol	$C_{27}H_{44}O_4$	[M-H]-	431.31668	431.31674	0.14								2.4E+06
389	Oleoylglycerone phosphate	$C_{21}H_{39}O_7P$	[M-H]-	433.23606	433.23609	0.07	7.3E+06						6.6E+06	
390	Trihydroxy-dihydrovitamin D3	$C_{27}H_{46}O_4$	[M-H]-	433.33233	433.33239	0.14		2.9E+06		4.0E+06	3.1E+06	3.9E+06		4.2E+06
391	Phe-Phe-CHN2	$C_{25}H_{28}N_4O$	[M+Cl]-	435.19571	435.19597	0.60								5.8E+06
392	Varanic acid	$C_{26}H_{44}O_5$	[M-H]-	435.31160	435.31164	0.09								2.2E+06

				ESI(-) FTICR-MS											
**No.**	**Plausible compound[a]**	**Molecular formula (M)**	**Ion**	**Theor. m/z[b]**	**Exp. m/z[c]**	**Δppm[d]**	**SM$_R$H[e]**	**SM$_R$O[e]**	**SM$_P$H[e]**	**SM$_P$O[e]**	**TF$_R$H[e]**	**TF$_R$O[e]**	**TF$_P$H[e]**	**TF$_P$O[e]**
393	Tridecyl oleate	$C_{29}H_{56}O_2$	[M-H]-	435.42075	435.42079	0.09		3.8E+06			4.5E+06	3.6E+06	2.2E+06	3.4E+06
394	Deoxythymidine 5'-diphosphate	$C_{10}H_{16}N_2O_{11}P_2$	[M+Cl]-	436.99233	436.99232	-0.02					3.7E+06		1.9E+07	
395	(Methyl-butanoyloxy)-villanovanediol	$C_{25}H_{42}O_6$	[M-H]-	437.29086	437.29093	0.16						2.2E+06		3.0E+06
396	Norcholestanepentol	$C_{26}H_{46}O_5$	[M-H]-	437.32725	437.32733	0.18								2.5E+06
397	Nonacosanoic acid	$C_{29}H_{58}O_2$	[M-H]-	437.43640	437.43646	0.14		6.0E+06		4.5E+06	7.6E+06	7.6E+06	7.2E+06	8.2E+06
398	MG(24:1/0:0/0:0)	$C_{27}H_{52}O_4$	[M-H]-	439.37928	439.37933	0.11	1.9E+07	1.4E+07		1.3E+07	9.5E+06	2.1E+07	1.1E+07	3.4E+07
399	Hydroxy-octacosanoic acid	$C_{28}H_{56}O_3$	[M-H]-	439.41567	439.41573	0.14		2.3E+06			3.2E+06		2.0E+06	3.2E+06
400	N-linolenoyl-glutamine	$C_{23}H_{38}N_2O_4$	[M+Cl]-	441.25256	441.25280	0.54	4.7E+07		1.1E+07	6.9E+07	3.7E+07	1.3E+08	6.9E+07	2.2E+08
401	MG(24:0/0:0/0:0)	$C_{27}H_{54}O_4$	[M-H]-	441.39493	441.39497	0.09		6.5E+06		4.4E+06	6.2E+06	8.4E+06	5.4E+06	9.9E+06
402	Dihydrophaseic acid 4-O-beta-D-glucoside	$C_{21}H_{32}O_{10}$	[M-H]-	443.19227	443.19229	0.05	1.5E+07		5.2E+06				4.9E+06	
403	Geranylgeranyl-glycerol phosphate	$C_{23}H_{41}O_6P$	[M-H]-	443.25680	443.25707	0.61		2.5E+06				2.8E+06		
404	Trihydroxycholanoic acid	$C_{24}H_{40}O_5$	[M+Cl]-	443.25698	443.25707	0.20		2.5E+06				2.8E+06		
405	Glyceryl-keto-PGF1	$C_{23}H_{40}O_8$	[M-H]-	443.26504	443.26509	0.11	5.4E+06				3.1E+06	2.2E+06	4.8E+06	3.1E+06
406	Dynorphin A	$C_{18}H_{39}N_9O_4$	[M-H]-	444.30522	444.30485	-0.83		3.1E+06				2.7E+06		4.5E+06
407	Trihydroxy-oxovitamin D3	$C_{27}H_{42}O_5$	[M-H]-	445.29595	445.29600	0.11						2.8E+06		4.3E+06
408	Grayanotoxin I	$C_{22}H_{36}O_7$	[M+Cl]-	447.21551	447.21557	0.13							2.4E+06	
409	Methyl-octacosadienoic acid	$C_{30}H_{56}O_2$	[M-H]-	447.42075	447.42079	0.09		3.2E+06			3.9E+06		2.5E+06	2.7E+06
410	Tetrahydroxyflavanone glucoside	$C_{21}H_{22}O_{11}$	[M-H]-	449.10894	449.10902	0.18	4.2E+06							
411	Estratrienetriol triacetate	$C_{24}H_{30}O_6$	[M+Cl]-	449.17364	449.17367	0.07		5.4E+06		4.2E+06		5.3E+06		
412	Coprocholic acid	$C_{27}H_{46}O_5$	[M-H]-	449.32725	449.32728	0.07						2.7E+06		2.9E+06
413	Myristyl oleate	$C_{30}H_{58}O_2$	[M-H]-	449.43640	449.43644	0.09	1.5E+07	1.4E+07		8.5E+06	1.5E+07	1.6E+07	1.3E+07	1.6E+07

ESI(-) FTICR-MS

No.	Plausible compound[a]	Molecular formula (M)	Ion	Theor. m/z[b]	Exp. m/z[c]	Δppm[d]	SM_RH[e]	SM_RO[e]	SM_PH[e]	SM_PO[e]	TF_RH[e]	TF_RO[e]	TF_PH[e]	TF_PO[e]
414	6-C-Glucopyranosylcatechin	$C_{21}H_{24}O_{11}$	[M-H]-	451.12459	451.12466	0.16			4.8E+06				8.0E+06	
415	Cyprinol	$C_{27}H_{48}O_5$	[M-H]-	451.34290	451.34294	0.09								2.6E+06
416	Tetradecyl-hexadecanoate	$C_{30}H_{60}O_2$	[M-H]-	451.45205	451.45208	0.07	9.2E+06	7.8E+06		6.0E+06	9.4E+06	1.1E+07	9.8E+06	1.1E+07
417	Glucoerysolin	$C_{12}H_{23}NO_{11}S_3$	[M-H]-	452.03605	452.03641	0.80	2.7E+06		3.6E+06				2.8E+06	
418	Pentamethoxy-prenyloxyflavone	$C_{25}H_{28}O_8$	[M-H]-	455.17114	455.17125	0.24	3.4E+06							
419	Volicitin	$C_{23}H_{38}N_2O_5$	[M+Cl]-	457.24747	457.24773	0.57								2.4E+06
420	Glucosyl sphingosine	$C_{24}H_{47}NO_7$	[M-H]-	460.32798	460.32797	-0.02								3.3E+06
421	Tephrorin A	$C_{24}H_{26}O_7$	[M+Cl]-	461.13725	461.13756	0.67	2.7E+06							
422	Hydroxypropyl-dihydroxy-norvitamin D3	$C_{29}H_{50}O_4$	[M-H]-	461.36363	461.36371	0.17	7.4E+06				2.7E+06			
423	D-Glucosyldihydrosphingosine	$C_{24}H_{49}NO_7$	[M-H]-	462.34363	462.34371	0.17								2.3E+06
424	Pentadecyl oleate	$C_{31}H_{60}O_2$	[M-H]-	463.45205	463.45210	0.11	6.6E+06	5.2E+06	3.0E+06	3.5E+06	5.3E+06	5.0E+06	5.5E+06	5.6E+06
425	Cholesterol sulfate	$C_{27}H_{46}O_4S$	[M-H]-	465.30440	465.30443	0.06	1.9E+08	1.4E+08	5.9E+07	9.4E+07	1.3E+08	2.6E+08	1.3E+08	2.5E+08
426	Methyl-triacontanoic acid	$C_{31}H_{62}O_2$	[M-H]-	465.46770	465.46778	0.17							2.6E+06	2.4E+06
427	Oxethazaine	$C_{28}H_{41}N_3O_3$	[M-H]-	466.30752	466.30779	0.58	5.7E+07	4.1E+07	1.8E+07	2.8E+07	3.9E+07	7.7E+07	4.0E+07	7.1E+07
428	Catechinol O-galactopyranoside	$C_{21}H_{24}O_{12}$	[M-H]-	467.11950	467.11956	0.13	2.8E+06							
429	Hydroxy-triacontanoic acid	$C_{30}H_{60}O_3$	[M-H]-	467.44697	467.44706	0.19	2.7E+06					3.6E+06		3.3E+06
430	TG(8:0/8:0/8:0)	$C_{27}H_{50}O_6$	[M-H]-	469.35346	469.35352	0.13	4.1E+06	2.8E+06			3.2E+06	4.3E+06		4.7E+06
431	Proteacin	$C_{20}H_{27}NO_{12}$	[M-H]-	472.14605	472.14607	0.04	5.2E+06							
432	Formyltetrahydrofolate	$C_{20}H_{23}N_7O_7$	[M-H]-	472.15862	472.15829	-0.70	2.7E+06							
433	Dihydroxy-dimethyl-hexadehydrovitamin D3	$C_{29}H_{42}O_3$	[M+Cl]-	473.28280	473.28266	-0.30		1.5E+08	1.2E+07	7.4E+07	2.8E+07	5.1E+07	3.4E+07	5.1E+07
434	Glucocheirolin	$C_{11}H_{21}NO_{11}S_3$	[M+Cl]-	473.99708	473.99669	-0.82	4.0E+06							

					ESI(-) FTICR-MS										
**No.**	**Plausible compound**[a]	**Molecular formula (M)**	**Ion**	**Theor. m/z**[b]	**Exp. m/z**[c]	**Δppm**[d]	**SM$_R$H**[e]	**SM$_R$O**[e]	**SM$_P$H**[e]	**SM$_P$O**[e]	**TF$_R$H**[e]	**TF$_R$O**[e]	**TF$_P$H**[e]	**TF$_P$O**[e]
435	Punaglandin 8	$C_{23}H_{33}ClO_6$	[M+Cl]-	475.16597	475.16591	-0.13			2.5E+06					
436	Kanokoside A	$C_{21}H_{32}O_{12}$	[M-H]-	475.18210	475.18217	0.15	3.6E+06	2.6E+06				3.0E+06		4.7E+06
437	Trihydroxy-dimethoxychalcone-O-glucoside	$C_{23}H_{26}O_{11}$	[M-H]-	477.14024	477.14051	0.57	2.7E+06							
438	Lusitanicoside	$C_{21}H_{30}O_{10}$	[M+Cl]-	477.15330	477.15320	-0.21	3.0E+06							
439	Palmityl oleate	$C_{32}H_{62}O_2$	[M-H]-	477.46770	477.46774	0.08	1.8E+07	1.3E+07	5.0E+06	8.7E+06	2.0E+07	1.6E+07	1.8E+07	1.6E+07
440	Hexahydroxyflavone 3-glucoside	$C_{21}H_{20}O_{13}$	[M-H]-	479.08311	479.08314	0.06	5.0E+06							
441	Dihydroxyvitamin D3 sulfur dioxide adduct	$C_{27}H_{44}O_5S$	[M-H]-	479.28367	479.28369	0.04	5.3E+06	2.7E+06	3.2E+06		2.9E+06	3.9E+06	2.8E+06	7.6E+06
442	Hydroxyecdysone	$C_{27}H_{44}O_7$	[M-H]-	479.30143	479.30146	0.06	3.9E+06		3.7E+06			2.6E+06		3.6E+06
443	Hexadecyl hexadecanoate	$C_{32}H_{64}O_2$	[M-H]-	479.48335	479.48339	0.08					3.5E+06		3.0E+06	
444	Fusicoccin H	$C_{26}H_{42}O_8$	[M-H]-	481.28069	481.28074	0.10	4.1E+06						2.6E+06	
445	Hydroxycholesterol sulfate	$C_{27}H_{46}O_5S$	[M-H]-	481.29932	481.29935	0.06	7.8E+06	4.9E+06	3.7E+06	3.6E+06	4.2E+06	6.6E+06	4.1E+06	1.1E+07
446	Triacontanedioic acid	$C_{30}H_{58}O_4$	[M-H]-	481.42623	481.42626	0.06	5.7E+07	8.6E+07	8.4E+06	4.1E+07	8.9E+07	1.2E+08	8.4E+07	1.3E+08
447	Digalloyl-glucose	$C_{20}H_{20}O_{14}$	[M-H]-	483.07803	483.07801	-0.04	3.0E+06							
448	Diospyrin	$C_{21}H_{24}O_{13}$	[M-H]-	483.11441	483.11441	0.00	3.3E+06							
449	Cholestan-heptol	$C_{27}H_{48}O_7$	[M-H]-	483.33273	483.33280	0.14		2.7E+06						2.9E+06
450	Cabergoline	$C_{26}H_{37}N_5O_2$	[M+Cl]-	486.26413	486.26437	0.49								3.1E+06
451	Fucosyllactose	$C_{18}H_{32}O_{15}$	[M-H]-	487.16684	487.16688	0.08		3.3E+06						4.0E+06
452	Ubiquinone Q4	$C_{29}H_{42}O_4$	[M+Cl]-	489.27771	489.27756	-0.31	5.3E+07	3.6E+07	2.5E+07	3.0E+07	4.8E+07	6.5E+07	3.0E+07	6.7E+07
453	Heptadecyl oleate	$C_{33}H_{64}O_2$	[M-H]-	491.48335	491.48340	0.10	4.1E+06	3.4E+06			5.7E+06	3.6E+06	3.7E+06	2.9E+06
454	Pentahydroxy-dimethoxyflavone L-arabinopyranoside	$C_{22}H_{22}O_{13}$	[M-H]-	493.09876	493.09879	0.06	1.8E+07		4.4E+06		3.6E+06			

ESI(-) FTICR-MS															
**No.**	**Plausible compound**[a]	**Molecular formula (M)**	**Ion**	**Theor. m/z**[b]	**Exp. m/z**[c]	**Δppm**[d]	**SM$_R$H**[e]	**SM$_R$O**[e]	**SM$_P$H**[e]	**SM$_P$O**[e]	**TF$_R$H**[e]	**TF$_R$O**[e]	**TF$_P$H**[e]	**TF$_P$O**[e]
455	N-(benzenesulfonamide) arachidonoyl amine	$C_{26}H_{38}N_2O_3S$	[M+Cl]-	493.22972	493.22933	-0.79					4.0E+06		4.6E+06	
456	Prednisolone tebutate	$C_{27}H_{38}O_6$	[M+Cl]-	493.23624	493.23626	0.04	3.3E+06		3.9E+06				2.9E+06	
457	Hydroxy-dimethoxyflavone glucoside	$C_{23}H_{24}O_{10}$	[M+Cl]-	495.10635	495.10590	-0.91	3.1E+06							
458	Arachidonoyl Serotonin	$C_{30}H_{42}N_2O_2$	[M+Cl]-	497.29403	497.29427	0.48								3.4E+06
459	Cholestanoctol	$C_{27}H_{48}O_8$	[M-H]-	499.32764	499.32772	0.16	3.8E+06							3.2E+06
460	Tetratriacontatetraenoic acid	$C_{34}H_{60}O_2$	[M-H]-	499.45205	499.45210	0.10	4.4E+06	4.3E+06	2.6E+06		5.4E+06	5.3E+06	4.8E+06	3.6E+06
461	Fabatriose	$C_{18}H_{30}O_{16}$	[M-H]-	501.14611	501.14611	0.00	3.6E+07		1.4E+07		2.8E+06		1.0E+07	
462	Trihydroxy-methoxy-phenylcoumarin O-acetylgalactoside	$C_{24}H_{24}O_{12}$	[M-H]-	503.11950	503.11952	0.04	3.4E+06							
463	(Benzodioxolyl)-tetramethoxy-[(methyl- butenyl)oxy]-benzopyranone	$C_{25}H_{26}O_9$	[M+Cl]-	505.12708	505.12741	0.65	1.1E+07		5.4E+06					
464	Stearyl oleate	$C_{34}H_{66}O_2$	[M-H]-	505.49900	505.49903	0.06	6.7E+06	5.0E+06			8.0E+06	4.2E+06	7.2E+06	4.8E+06
465	Tetrahydroxy-dimethoxyflavone glucoside	$C_{23}H_{24}O_{13}$	[M-H]-	507.11441	507.11470	0.57	4.5E+06							
466	DG(14:0/14:0/0:0)	$C_{31}H_{60}O_5$	[M-H]-	511.43680	511.43682	0.04					3.3E+06			4.7E+06
467	Apigenin-(diacetylglucoside)	$C_{25}H_{24}O_{12}$	[M-H]-	515.11950	515.11949	-0.02	1.1E+07		8.0E+06				1.1E+07	
468	Glucosyloxydocosanoate	$C_{28}H_{54}O_8$	[M-H]-	517.37459	517.37461	0.04	1.1E+07		3.9E+06		4.1E+06		9.8E+06	
469	Hydrocortisone cypionate	$C_{29}H_{42}O_6$	[M+Cl]-	521.26754	521.26763	0.17			4.0E+06					
470	DG(14:1/15:0/0:0)	$C_{32}H_{60}O_5$	[M-H]-	523.43680	523.43681	0.02					3.6E+06			
471	Hexatriacontatetraenoic acid	$C_{36}H_{64}O_2$	[M-H]-	527.48335	527.48339	0.08	5.6E+06	3.4E+06			5.6E+06		4.9E+06	3.5E+06
472	Luteolin glucoside-4'-(methyl-butenoate)	$C_{26}H_{26}O_{12}$	[M-H]-	529.13515	529.13518	0.06	3.2E+06							
473	Epoxymurin-A	$C_{35}H_{62}O_3$	[M-H]-	529.46262	529.46267	0.09					4.3E+07		1.8E+07	
474	Cefsulodin	$C_{22}H_{20}N_4O_8S_2$	[M-H]-	531.06498	531.06479	-0.36	1.0E+07							

				ESI(-) FTICR-MS											
No.	Plausible compound[a]	Molecular formula (M)	Ion	Theor. m/z[b]	Exp. m/z[c]	Δppm[d]	SM_RH[e]	SM_RO[e]	SM_PH[e]	SM_PO[e]	TF_RH[e]	TF_RO[e]	TF_PH[e]	TF_PO[e]
475	Mayolene	$C_{34}H_{60}O_4$	[M-H]-	531.44188	531.44191	0.06	6.8E+06	5.2E+06		4.3E+06	6.7E+06	7.6E+06	6.3E+06	5.1E+06
476	Nocardicin B	$C_{23}H_{24}N_4O_9$	[M+Cl]-	535.12373	535.12326	-0.88			4.7E+06					5.3E+06
477	D-Glucan	$C_{18}H_{32}O_{18}$	[M-H]-	535.15159	535.15160	0.02	4.1E+06							
478	Cellotriose	$C_{18}H_{32}O_{16}$	[M+Cl]-	539.13844	539.13856	0.22			2.4E+06					
479	DG(14:0/16:0/0:0)	$C_{33}H_{64}O_5$	[M-H]-	539.46810	539.46812	0.04	5.3E+06	4.2E+06			6.6E+06	1.1E+07	5.6E+06	7.5E+06
480	CMP-3-deoxy-manno-octulosonate	$C_{17}H_{26}N_3O_{15}P$	[M-H]-	542.10288	542.10329	0.76	7.4E+06							
481	Polyoxin B	$C_{17}H_{25}N_5O_{13}$	[M+Cl]-	542.11429	542.11417	-0.22	5.7E+06							
482	Estriol 3-sulfate 16-glucuronide	$C_{24}H_{32}O_{12}S$	[M-H]-	543.15417	543.15432	0.28						8.5E+06		5.2E+06
483	Bacteriohopane-tetrol	$C_{35}H_{62}O_4$	[M-H]-	545.45753	545.45755	0.04		4.0E+06					4.0E+06	
484	DG(15:0/16:1/0:0)	$C_{34}H_{64}O_5$	[M-H]-	551.46810	551.46810	0.00		4.7E+06			4.5E+06		5.9E+06	6.8E+06
485	TG(10:0/10:0/10:0)	$C_{33}H_{62}O_6$	[M-H]-	553.44736	553.44739	0.05		4.0E+06			3.6E+06			4.2E+06
486	DG(15:0/16:0/0:0)	$C_{34}H_{66}O_5$	[M-H]-	553.48375	553.48375	0.00	4.5E+06	5.1E+06			5.4E+06	9.3E+06	5.2E+06	6.7E+06
488	Methylbacteriohopane-tetrol	$C_{36}H_{64}O_4$	[M-H]-	559.47318	559.47318	0.00	9.4E+06	9.6E+06		5.9E+06	1.3E+07	1.4E+07	1.3E+07	1.2E+07
489	Cholesterol glucuronide	$C_{33}H_{54}O_7$	[M-H]-	561.37968	561.37964	-0.07	6.8E+06				4.6E+06			4.3E+06
490	DG(18:2/14:0/0:0)	$C_{35}H_{64}O_5$	[M-H]-	563.46810	563.46825	0.27								3.7E+06
491	N-Stearoylsphingosine	$C_{36}H_{71}NO_3$	[M-H]-	564.53612	564.53616	0.07						6.5E+06		
492	Uridine diphosphategalactose	$C_{15}H_{24}N_2O_{17}P_2$	[M-H]-	565.04774	565.04770	-0.07	2.4E+07		1.1E+07		7.5E+06		1.0E+07	
493	DG(14:0/18:0/0:0)	$C_{35}H_{68}O_5$	[M-H]-	567.49940	567.49939	-0.02	5.8E+06	4.8E+06			6.5E+06	1.1E+07	6.2E+06	9.4E+06
494	DG(14:1/16:1/0:0)	$C_{33}H_{60}O_5$	[M+Cl]-	571.41348	571.41352	0.07	4.1E+06							
495	N-Palmitoylsphingosine	$C_{34}H_{67}NO_3$	[M+Cl]-	572.48150	572.48146	-0.07	4.6E+06							
496	DG(16:1/14:0/0:0)	$C_{33}H_{62}O_5$	[M+Cl]-	573.42913	573.42912	-0.02	9.9E+06	6.3E+06	3.2E+06	5.6E+06	7.2E+06			9.1E+06

ESI(-) FTICR-MS

No.	Plausible compound[a]	Molecular formula (M)	Ion	Theor. m/z[b]	Exp. m/z[c]	Δppm[d]	SM_RH[e]	SM_RO[e]	SM_PH[e]	SM_PO[e]	TF_RH[e]	TF_RO[e]	TF_PH[e]	TF_PO[e]
497	(O-glucopyranosyl)-keto-hexacosanediol	$C_{32}H_{62}O_8$	[M-H]-	573.43719	573.43719	0.00	1.1E+07		3.5E+06				9.7E+06	
498	Proanthocyanidin A2	$C_{30}H_{24}O_{12}$	[M-H]-	575.11950	575.12006	0.97	3.1E+07		1.2E+07				1.6E+07	
499	(Glucopyranosyl)-hexacosanetriol	$C_{32}H_{64}O_8$	[M-H]-	575.45284	575.45286	0.03	6.5E+07		2.1E+07		1.4E+07		5.4E+07	
500	Isochamaejasmin	$C_{30}H_{22}O_{10}$	[M+Cl]-	577.09070	577.09015	-0.95			5.1E+06					
501	DG(15:0/18:2/0:0)	$C_{36}H_{66}O_5$	[M-H]-	577.48375	577.48382	0.12					4.0E+06			3.8E+06
502	Cer(d18:1/19:0)	$C_{37}H_{73}NO_3$	[M-H]-	578.55177	578.55176	-0.02						7.8E+06		4.1E+06
503	DG(15:0/18:1/0:0)	$C_{36}H_{68}O_5$	[M-H]-	579.49940	579.49942	0.03		4.0E+06			4.7E+06		4.5E+06	5.6E+06
504	DG(O-16:0/18:1)	$C_{37}H_{72}O_4$	[M-H]-	579.53578	579.53579	0.02	1.2E+07	1.5E+07		7.6E+06	1.8E+07	2.1E+07	1.9E+07	1.9E+07
506	Coixenolide	$C_{38}H_{70}O_4$	[M-H]-	589.52013	589.52013	0.00	8.1E+06	1.2E+07		6.9E+06	1.4E+07	1.7E+07	1.5E+07	1.5E+07
507	Cer(d18:2/20:0)	$C_{38}H_{73}NO_3$	[M-H]-	590.55177	590.55172	-0.08					3.8E+06	8.8E+06		
508	DG(16:0/18:2/0:0)	$C_{37}H_{68}O_5$	[M-H]-	591.49940	591.49937	-0.05	4.5E+06				4.6E+06	8.3E+06	3.9E+06	5.1E+06
509	Cer(d16:1/22:0)	$C_{38}H_{75}NO_3$	[M-H]-	592.56742	592.56743	0.02			3.0E+06		5.5E+06	1.7E+07		
510	DG(16:0/18:1/0:0)	$C_{37}H_{70}O_5$	[M-H]-	593.51505	593.51507	0.03		3.8E+06					3.8E+06	4.5E+06
511	PI(18:2/0:0)	$C_{27}H_{49}O_{12}P$	[M-H]-	595.28889	595.28877	-0.20			3.7E+06					
512	[D-glucopyranosyl]-furostantriol	$C_{33}H_{56}O_9$	[M-H]-	595.38516	595.38519	0.05	6.8E+06						3.8E+06	
513	DG(16:0/18:0/0:0)	$C_{37}H_{72}O_5$	[M-H]-	595.53070	595.53080	0.17	3.8E+06				4.3E+06			
514	Catechin 3-O-rutinoside	$C_{27}H_{34}O_{15}$	[M-H]-	597.18249	597.18253	0.07	1.1E+07						4.1E+06	
515	Cyanidin 3-(6''-malylglucoside)	$C_{25}H_{25}O_{15}$	[M+Cl]-	600.08875	600.08879	0.07	4.1E+06		8.6E+06				9.8E+06	
516	DG(16:0/16:1/0:0)	$C_{35}H_{66}O_5$	[M+Cl]-	601.46043	601.46041	-0.03			4.1E+06	9.6E+06				
517	(O-glucopyranosyl)-keto-octacosanediol	$C_{34}H_{66}O_8$	[M-H]-	601.46849	601.46848	-0.02	8.4E+06						6.2E+06	
518	(O-galactopyranosyl)-octaconsanetriol	$C_{34}H_{68}O_8$	[M-H]-	603.48414	603.48413	-0.02	1.6E+07		6.0E+06		6.2E+06		1.4E+07	

ESI(-) FTICR-MS															
No.	Plausible compound[a]	Molecular formula (M)	Ion	Theor. m/z[b]	Exp. m/z[c]	Δppm[d]	SM$_R$H[e]	SM$_R$O[e]	SM$_P$H[e]	SM$_P$O[e]	TF$_R$H[e]	TF$_R$O[e]	TF$_P$H[e]	TF$_P$O[e]
519	Kinetensin	$C_{26}H_{38}N_9O_6$	[M+Cl]-	607.26391	607.26411	0.33		4.6E+06				5.2E+06	4.7E+06	
520	Pentahydroxyflavone neohesperidoside	$C_{27}H_{30}O_{16}$	[M-H]-	609.14611	609.14609	-0.03	1.5E+07		6.1E+06		5.4E+06		3.2E+07	
521	Glutathione disulfide	$C_{20}H_{32}N_6O_{12}S_2$	[M-H]-	611.14469	611.14472	0.05	5.2E+06		3.7E+06					
522	Evasterioside D	$C_{33}H_{58}O_{10}$	[M-H]-	613.39572	613.39571	-0.02	1.4E+07	9.4E+06	5.7E+06	7.1E+06	5.8E+06	7.9E+06	6.7E+06	1.4E+07
523	Dimethyl-pentadecanoyl-(ladderane-octanyl)-sn- glycerol	$C_{40}H_{72}O_4$	[M-H]-	615.53578	615.53578	0.00		4.1E+06					4.3E+06	
524	DG(15:0/18:0/0:0)	$C_{36}H_{70}O_5$	[M+Cl]-	617.49173	617.49178	0.08				4.1E+06				
525	Cer(d18:1/22:1)	$C_{40}H_{77}NO_3$	[M-H]-	618.58307	618.58313	0.10						4.9E+06		
526	Kanokoside D	$C_{27}H_{44}O_{16}$	[M-H]-	623.25566	623.25564	-0.03	1.0E+07		5.0E+06					
527	DG(16:0/18:3/0:0)	$C_{37}H_{66}O_5$	[M+Cl]-	625.46043	625.46037	-0.10	5.8E+06							
528	Kurilensoside F	$C_{33}H_{58}O_{11}$	[M-H]-	629.39064	629.39063	-0.02	6.5E+06							
529	TG(12:0/12:0/12:0)	$C_{39}H_{74}O_6$	[M-H]-	637.54126	637.54126	0.00	6.3E+06				5.2E+06	6.1E+06	5.5E+06	6.3E+06
530	Myricetin 3,3'-digalactoside	$C_{27}H_{30}O_{18}$	[M-H]-	641.13594	641.13592	-0.03	4.2E+06							
531	Galactosylceramide (d18:1/12:0)	$C_{36}H_{69}NO_8$	[M-H]-	642.49504	642.49500	-0.06	6.2E+06	4.1E+06			7.6E+06	6.1E+06	6.5E+06	6.9E+06
532	DG(15:0/20:1/0:0)	$C_{38}H_{72}O_5$	[M+Cl]-	643.50738	643.50743	0.08	4.5E+06							
533	Didecanoyl-docosanediol	$C_{42}H_{82}O_4$	[M-H]-	649.61403	649.61400	-0.05	2.1E+07	2.3E+07		1.1E+07	3.5E+07	3.5E+07	3.5E+07	2.8E+07
534	DG(16:1/20:3/0:0)	$C_{39}H_{68}O_5$	[M+Cl]-	651.47608	651.47605	-0.05	3.6E+06							
535	Tetraacetoxy-cholestenol	$C_{35}H_{54}O_9$	[M+Cl]-	653.34618	653.34602	-0.24	8.9E+06		3.8E+06		3.5E+06		8.7E+06	
536	DG(16:0/20:3/0:0)	$C_{39}H_{70}O_5$	[M+Cl]-	653.49173	653.49176	0.05	4.9E+06							
537	DG(14:0/22:2/0:0)	$C_{39}H_{72}O_5$	[M+Cl]-	655.50738	655.50719	-0.29		5.3E+06		3.6E+06				
538	DG(16:0/20:1/0:0)	$C_{39}H_{74}O_5$	[M+Cl]-	657.52303	657.52281	-0.33		4.4E+06		3.4E+06				

				ESI(-) FTICR-MS										
**No.**	**Plausible compound**[a]	**Molecular formula (M)**	**Ion**	**Theor. m/z**[b]	**Exp. m/z**[c]	**Δppm**[d]	**SM$_R$H**[e]	**SM$_R$O**[e]	**SM$_P$H**[e]	**SM$_P$O**[e]	**TF$_R$H**[e]	**TF$_R$O**[e]	**TF$_P$H**[e]	**TF$_P$O**[e]
539	Maltotetraose	$C_{24}H_{42}O_{21}$	[M-H]-	665.21458	665.21419	-0.59	3.9E+06							
540	Ikarisoside F	$C_{31}H_{36}O_{14}$	[M+Cl]-	667.17991	667.18025	0.51	3.6E+06							
541	Pandaroside B	$C_{35}H_{54}O_{10}$	[M+Cl]-	669.34110	669.34104	-0.09	4.4E+06		4.2E+06					
542	PA(16:0/18:3)	$C_{37}H_{67}O_8P$	[M-H]-	669.45008	669.45000	-0.12						3.8E+06		
543	GlcCer(d18:1/14:0)	$C_{38}H_{73}NO_8$	[M-H]-	670.52634	670.52624	-0.15							3.7E+06	
544	PA(16:0/18:2)	$C_{37}H_{69}O_8P$	[M-H]-	671.46573	671.46567	-0.09		4.7E+06		1.1E+07		7.6E+06		9.7E+06
545	Quercetin glucoside-glucuronide	$C_{27}H_{28}O_{18}$	[M+Cl]-	675.09697	675.09701	0.06	5.9E+06		5.2E+06				5.8E+06	
546	Sophorosyloxydocosanoic acid	$C_{34}H_{64}O_{13}$	[M-H]-	679.42742	679.42733	-0.13	7.0E+06							
547	Cer(d18:1/24:0)	$C_{42}H_{83}NO_3$	[M+Cl]-	684.60670	684.60692	0.32	4.0E+06							
548	Glucopyranosyl-(tetradecanoyloxy)-eicosanol	$C_{40}H_{78}O_8$	[M-H]-	685.56239	685.56235	-0.06	4.8E+06				5.2E+06		5.1E+06	
549	PA(18:2/18:3)	$C_{39}H_{67}O_8P$	[M-H]-	693.45008	693.45016	0.12						3.6E+06		
550	Phylloflavanine	$C_{35}H_{32}O_{13}$	[M+Cl]-	695.15369	695.15333	-0.52	4.8E+06							
551	PA(18:1/18:3)	$C_{39}H_{69}O_8P$	[M-H]-	695.46573	695.46570	-0.04				7.2E+06		4.2E+06		
552	Cer(t18:0/24:0)	$C_{42}H_{85}NO_4$	[M+Cl]-	702.61726	702.61727	0.01	3.8E+06							
553	Ceramide (d18:1/26:0)	$C_{44}H_{87}NO_3$	[M+Cl]-	712.63800	712.63799	-0.01	4.1E+06							
554	PC(O-16:0/O-18:1)	$C_{42}H_{86}NO_6P$	[M-H]-	730.61200	730.61202	0.03	4.3E+06							
555	Quercetin glucosyl-xylosyl-rhamnoside	$C_{32}H_{38}O_{20}$	[M-H]-	741.18837	741.18828	-0.12	7.3E+06		4.4E+06				8.8E+06	
556	TG(16:0/14:0/16:1)	$C_{49}H_{92}O_6$	[M-H]-	775.68211	775.68172	-0.50					3.9E+06			
557	Quercetin-(di-p-coumarylglucoside)	$C_{39}H_{32}O_{16}$	[M+Cl]-	791.13844	791.13897	0.67			3.3E+06					
558	PI(16:0/18:3)	$C_{43}H_{77}O_{13}P$	[M-H]-	831.50290	831.50270	-0.24			3.9E+06					
559	PI(16:0/18:2)	$C_{43}H_{79}O_{13}P$	[M-H]-	833.51855	833.51838	-0.20	5.9E+06		5.3E+06	4.0E+06			3.1E+06	

					ESI(-) FTICR-MS								
**No.**	**Plausible compound[a]**	**Molecular formula (M)**	**Ion**	**Theor. m/z[b]**	**Exp. m/z[c]**	**Δppm[d]**	**SM_RH[e]**	**SM_RO[e]**	**SM_PH[e]**	**SM_PO[e]**	**TF_RH[e]**	**TF_RO[e]**	**TF_PH[e]**	**TF_PO[e]**
560	Diguanosine tetraphosphate	$C_{20}H_{28}N_{10}O_{21}P_4$	[M-H]-	867.03082	867.03164	0.95			2.9E+06					
561	Malonyl-CoA semialdehyde	$C_{24}H_{38}N_7O_{18}P_3S$	[M+Cl]-	872.09009	872.08952	-0.65	3.6E+06		3.8E+06					

[a] Cer: Ceramide; GalCer: Galactosylceramide; GlcCer: Glucosylceramide; LacCer: Lactosylceramide; MG: Monoacylglycerol; DG: Diacylglycerol; TG: Triacylglycerol; MGDG: Monoacyldiacylglycerol; PA:
Phosphatidic acid; PC: Phosphatidylcholine; PE: Phosphatidylethanolamine; PG: Glycerophospholipids; PI: Phosphatidylinositol; PS: Phosphatidylserine;; SM: Sphingomyelin

[b] Theor. stands for calculated exact mass to charge ratio

[c] Exp. stands for experimental m/z value

[d] The error expressed in parts per million (ppm)

[e] SM_RH and SM_RO stand for hydroalcoholic and organic portions of red San Marzano extracts, respectively; SM_PH and SM_PO stand for hydroalcoholic and organic portions of pink San Marzano extracts, respectively. TF_RH and TF_RO stand for hydroalcoholic and organic portions of red Torpedino di Fondi extracts, respectively; TF_PH and TF_PO stand for hydroalcoholic and organic portions of pink Torpedino di Fondi extracts, respectively.

Table 4. Overview of the relative abundancies of specific classes of saturated, mono-, di- and poly-unsaturated fatty acids (FA) obtained by ESI(-) FT-ICR MS analyses of SM_P, SM_R, TF_P, TF_R organic extracts. .

	FA	Formula	Accurate m/z ratio	(Δppm)[a]	SM_P	SM_R	TF_P	TF_R
Saturated Fatty Acids	12:0	$[C_{12}H_{23}O_2]^-$	*m/z* 199.17040	(0.2)	0.4	0.3	0.3	0.4
	14:0	$[C_{14}H_{27}O_2]^-$	*m/z* 227.20166	(0.04)	7.8	7.5	7.8	8.3
	16:0	$[C_{16}H_{31}O_2]^-$	*m/z* 255.23299	(0.2)	59.6	60.2	59.5	61.3
	18:0	$[C_{18}H_{35}O_2]^-$	*m/z* 283.26430	(0.2)	29.9	25.6	30.2	27.5
	20:0	$[C_{20}H_{39}O_2]^-$	*m/z* 311.29560	(0.2)	2.3	6.4	2.2	2.4
Mono-unsaturated	12:1	$[C_{12}H_{21}O_2]^-$	/	/				
	14:1	$[C_{14}H_{25}O_2]^-$	*m/z* 225.18601	(0.04)	2.8	5.5	2.9	3.0
	16:1	$[C_{16}H_{29}O_2]^-$	*m/z* 253.21730	(0.0)	55.7	57.5	58.1	55.4
	18:1	$[C_{18}H_{33}O_2]^-$	*m/z* 281.24864	(0.1)	39.2	39.8	38.8	39.1
	20:1	$[C_{20}H_{37}O_2]^-$	*m/z* 309.27995	(0.2)	2.3	0.2	0.1	2.4
Di-unsaturated	12:2	$[C_{12}H_{21}O_2]^-$	/	/				
	14:2	$[C_{14}H_{23}O_2]^-$	/	/				
	16:2	$[C_{16}H_{27}O_2]^-$	*m/z* 251.20172	(0.0)	6.1	5.0	6.4	6.7
	18:2	$[C_{18}H_{31}O_2]^-$	*m/z* 279.23299	(0.1)	76.7	62.0	77.0	76.1
	20:2	$[C_{20}H_{35}O_2]^-$	*m/z* 307.26425	(0.0)	17.1	32.9	16.6	17.2
Poly-unsaturated	16:3	$[C_{16}H_{25}O_2]^-$	*m/z* 249.18603	(0.1)	0.0	1.2	0.0	0.0
	18:3	$[C_{18}H_{29}O_2]^-$	*m/z* 277.21735	(0.0)	37.0	20.4	34.7	37.2
	20:3	$[C_{20}H_{33}O_2]^-$	*m/z* 305.24860	(0.2)	63.0	78.4	65.3	62.8
	18:4	$[C_{18}H_{27}O_2]^-$	/	/				
	20:4	$[C_{20}H_{31}O_2]^-$	*m/z* 303.23295	(0.2)	0.0	100	100	0.0

[a)] Mass accuracy expressed in part per million

Table 5. Antifungal activity of tomato fruit extracts against 4 *C. albicans* strains (ATCC10231, ATCC24433, 3153A, PMC1033), 3 *C. glabrata* strains (PMC0822, PMC0851, PMC0807) and 2 *C. krusei* strains (PMC0631, PMC0624). [a, b.]

Candida	**MIC$_{50}$ (μg /mL)**															
	**ORGANIC**				**HYDROALCOHOLIC**				**ORGANIC**				**HYDROALCOHOLIC**			
	**SM_R**		**SM_P**		**SM_R**		**SM_P**		**TF_R**		**TF_P**		**TF_R**		**TF_P**	
	**Range**	**GM**	**range**	**GM**	**range**	**GM**	**range**	**GM**	**range**	**GM**	**range**	**GM**	**range**	**GM**	**range**	**GM**
***albicans*[a]**	1000->1000	1834	250->1000	771	1000->1000	1682	1000->1000	1682	250->1000	841	500->1000	1000	1000->1000	1682	500->1000	1297
***glabrata*[b]**	500->1000	944	250-1000	630	500->1000	944	250->1000	891	250-500	281	250-1000	375	500-1000	794	250-1000	595
***krusei*[c]**	500->1000	841	>1000	>1000	>1000	>1000	>1000	>1000	>1000	>1000	>1000	>1000	>1000	>1000	>1000	>1000

	**MIC$_{90}$ (μg /mL)**															
	ORGANIC				HYDROALCOHOLIC				ORGANIC				HYDROALCOHOLIC			
	SM_R		SM_P		SM_R		SM_P		TF_R		TF_P		TF_R		TF_P	
	Range	GM	range	GM	range	GM	range	GM	range	GM	range	GM	range	GM	range	GM
***albicans*[c]**	>1000	>1000	500->1000	1297	>1000	>1000	>1000	>1000	500->1000	1190	500->1000	1297	>1000	>1000	500->1000	1682
***glabrata*[d]**	1000->1000	1498	1000->1000	1587	1000->1000	1498	500->1000	1059	500-1000	561	500-1000	707	1000->1000	1260	1000	1000
***krusei*[e]**	1000>1000	1414	>1000	>1000	>1000	>1000	>1000	>1000	>1000	>1000	>1000	>1000	>1000	>1000	>1000	>1000

	**MIC$_{100}$ (μg /mL)**															
	ORGANIC				HYDROALCOHOLIC				ORGANIC				HYDROALCOHOLIC			
	SM_R		SM_P		SM_R		SM_P		TF_R		TF_P		TF_R		TF_P	
	range	GM	range	GM	range	GM	range	GM	range	GM	range	GM	range	GM	range	GM
***albicans*[c]**	>1000	>1000	>1000	>1000	>1000	>1000	>1000	>1000	1000->1000	1834	1000->1000	1834	>1000	>1000	>1000	>1000
***glabrata*[d]**	>1000	>1000	>1000	>1000	>1000	>1000	>1000	>1000	>1000	>1000	>1000	>1000	>1000	>1000	>1000	>1000
***krusei*[e]**	>1000	>1000	>1000	>1000	>1000	>1000	>1000	>1000	>1000	>1000	>1000	>1000	>1000	>1000	>1000	>1000

[a)] MIC=minimal inhibitory concentration. [b)] GM= geometric mean MIC$_{50}$, MIC$_{80}$, MIC$_{90}$, and MIC$_{100}$ are the lowest concentration of extracts that caused growth inhibition ≥ 50%, ≥ 80%, ≥ 90% and 100%, respectively. [c)] 4 strains (ATCC10231, ATCC24433, 3153A, PMC1033); [d)] 3 strains (PMC0822, PMC0851, PMC0807); [e)] and 2 strains (PMC0631, PMC0624).
